# 12-month neurological and psychiatric outcomes of semaglutide use for type 2 diabetes: a propensity-score matched cohort study

**DOI:** 10.1016/j.eclinm.2024.102726

**Published:** 2024-07-10

**Authors:** Riccardo De Giorgi, Ivan Koychev, Amanda I. Adler, Philip J. Cowen, Catherine J. Harmer, Paul J. Harrison, Maxime Taquet

**Affiliations:** a Department of Psychiatry, University of Oxford, Warneford Hospital, Warneford Lane, Oxford, OX3 7JX, United Kingdom; b Oxford Health NHS Foundation Trust, Warneford Hospital, Warneford Lane, Oxford, OX3 7JX, United Kingdom; c Department of Psychological Medicine, John Radcliffe Hospital, Headley Way, Oxford, OX3 9DU, United Kingdom; d Diabetes Trials Unit, Oxford Centre for Diabetes, Endocrinology and Metabolism, University of Oxford, Churchill Hospital, Oxford, OX3 7LE, United Kingdom

**Keywords:** Semaglutide, Cognition, Neurological outcomes, Psychiatric outcomes, Pharmacoepidemiology

## Abstract

**Background:**

While semaglutide, approved for type-2 diabetes mellitus (T2DM), is being investigated as a treatment for brain disorders, concerns over adverse neuropsychiatric events have emerged. More data are therefore needed to assess the effects of semaglutide on brain health. This study provides robust estimates of the risk of neurological and psychiatric outcomes following semaglutide use compared to three other antidiabetic medications.

**Methods:**

This retrospective cohort study used electronic health records from TriNetX US Collaborative Network, covering >100 million patients in the USA. Due to the exploratory nature of this study, we did not use a pre-registered protocol or statistical analysis plan. Three cohorts with T2DM prescribed semaglutide between 1st December 2017 and 31st May 2021 were propensity-score matched (1:1 using a greedy nearest-neighbour algorithm with calliper distance of 0.1) with cohorts receiving sitagliptin, empagliflozin, and glipizide. Using Cox regression analysis, we compared the risks of 22 neurological and psychiatric outcomes within one year since the index prescription: encephalitis, parkinsonism, cognitive deficit, dementia, epilepsy/seizure, migraine, insomnia, nerve disorder, myoneural junction/muscle disease, intracranial haemorrhage, ischaemic stroke, alcohol misuse, opioid misuse, cannabis misuse, stimulants misuse, nicotine misuse, psychosis, bipolar disorder, depression, anxiety, obsessive-compulsive disorder, and suicidality. Negative control outcomes (NCOs) were used to assess unmeasured confounding.

**Findings:**

Each matched cohort included 23,386 (semaglutide *vs* sitagliptin), 22,584 (*vs* empagliflozin), and 19,206 (*vs* glipizide) patients. Semaglutide was not associated with an increased risk of neurological and psychiatric outcomes. Instead, after multiple-testing correction, semaglutide was associated with reduced risk for several such outcomes, notably cognitive deficit compared to sitagliptin (HR 0.72, 95% CI 0.64–0.80) and glipizide (HR 0.72, 95% CI 0.63–0.81), dementia compared to sitagliptin (HR 0.52, 95% CI 0.40–0.68), and nicotine misuse across most comparisons (HR 0.72, 95% CI 0.61–0.85 against glipizide; HR 0.77, 95% CI 0.65–0.90 against empagliflozin; HR 0.82, 95% CI 0.70–0.95 against sitagliptin, though the latter was no longer statistically significant after adjustment for multiple comparisons). Empagliflozin showed fewest differences from semaglutide. No differences in NCOs were observed between cohorts.

**Interpretation:**

Semaglutide is not associated with higher 12-month risk of adverse neuropsychiatric outcomes compared to other antidiabetic medications. Potential beneficial associations with some outcomes, especially cognitive deficit and nicotine misuse, should stimulate validation in clinical trials.

**Funding:**

10.13039/501100000272National Institute for Health Research (NIHR) Oxford Health Biomedical Research Centre, Medical Research Council.

**Research in context**

**Evidence before this study**

We searched Embase, PsycInfo, and PubMed/MEDLINE via Ovid SP on 1st March 2024 for relevant clinical studies using the terms “(semaglutide OR ‘glp-1 receptor agonist’) AND (neurolog∗ OR cognit∗ OR psychiatr∗ OR mental) AND (incidence OR epidemiolog∗ OR pharmacovigilance OR ‘systematic review’ OR ‘meta-analysis’)”. For cognitive and depression outcomes, two meta-analyses based on both interventional and observational data had shown a promising effect of GLP1-RAs, but the number of studies and participants was low, data for the recently-approved semaglutide was scant, and heterogeneity was high. A recent disproportionality analysis of the US Food and Drugs Administration (FDA) Adverse Event Reporting System (FAERS) database has seen many psychiatric adverse events with higher incidence in GLP1-RAs users. Following the opening of the European Medicines Agency (EMA) inquiry in July 2023, numerous epidemiological studies have been examining the risk of depression, suicide, or self-harm associated with GLP1-RAs, leading to conflicting results over short follow-ups. Earlier studies have focussed on physical health outcomes associated with GLP1-RAs in people with neurological and psychiatric disorders, especially in relation to antipsychotic use. Lastly, a search on Clinicaltrials.gov retrieved 13 ongoing trials of semaglutide for cognitive disorders, substance misuse, psychosis, and depression. We did not identify any large-scale study including a control group investigating neuropsychiatric outcomes (except for suicidality).

**Added value of this study**

To our knowledge, we provide the first robust estimates of the risks for an array of common or serious neurological and psychiatric conditions in the 12 months following a first prescription of semaglutide compared to three other antidiabetic drugs, using the electronic health records of 23,386 (semaglutide *vs* sitagliptin), 22,584 (*vs* empagliflozin), and 19,206 (*vs* glipizide) patients with T2DM in each cohort. We show that semaglutide use is not associated with higher risk for any of the outcomes measured. To the contrary, semaglutide shows a protective association against cognitive deficit and nicotine misuse. Evidence regarding a putative protective effect in depression remains uncertain.

**Implications of all the available evidence**

Concerns regarding potential neuropsychiatric adverse outcomes associated with semaglutide are not supported by our analyses, which is informative to regulatory bodies, patients, and clinicians. Our findings instead prompt further investigations into the role of semaglutide for the treatment or prevention of cognitive deficits and nicotine misuse.

## Introduction

Semaglutide is a glucagon-like peptide 1 receptor agonist (GLP1-RA) licensed for type 2 diabetes mellitus (T2DM) and obesity.^1^ GLP1-RAs have been hailed as the scientific breakthrough of the year 2023.^2^ Healthcare spending on these medications is predicted to expand further as novel indications,^3^ formulations,^4^ and related molecules^5^ become available.

Randomised controlled trials (RCTs) have confirmed the efficacy of semaglutide on metabolic measures^6^ and cardiovascular morbidity and mortality in the general population.^7^ Further studies are investigating the effects of GLP1-RAs in patients with other chronic diseases.^8^ Pre-clinical evidence suggests that these medications have neurobiological activity, including protection against neuronal degeneration and inflammation, as well as modulation of dopamine-related reward mechanisms.^9^, ^10^, ^11^, ^12^ Semaglutide is therefore under consideration for use in neurological, psychiatric, and substance use disorders.^13^, ^14^, ^15^, ^16^, ^17^ However, in July 2023, the European Medicines Agency (EMA) and the UK Medicines and Healthcare products Regulatory Agency (MHRA) began a review of these medications’ safety, following reports of worsening mood and suicidal behaviour in GLP1-RAs users.^18^ The US Food and Drug Administration (FDA) has issued a preliminary evaluation suggesting no causal link,^19^ but concerns over adverse neuropsychiatric events still resonate within the media and with the public.^20^ The safety profile of semaglutide, and GLP-1 RAs more broadly, remains under assessment; a better clarification of it is necessary to inform patients, prescribers, and policymakers.^21^

Whether semaglutide has positive or negative effects on brain health therefore still needs to be determined. In this regard, studies using electronic health records (EHR) are providing valuable information about the comparative effectiveness^22^ and safety^23^ of semaglutide for outcomes other than glycaemic control, including suicidality.^24^

In this propensity-score matched cohort study based on EHR data from 100 million people in the USA, we estimate the risks of neurological and psychiatric outcomes within one year of a first prescription of semaglutide in patients with T2DM, and compare them with those arising after a first prescription of three other common antidiabetic medications.

## Methods

This study follows the Reporting of studies Conducted using Observational Routinely-collected health Data (RECORD) guidelines.

### Study design and data collection

In this retrospective cohort study, we used TriNetX US Collaborative Network, a large-scale federated database which, at the time of study, holds anonymised EHRs of >100 million insured and uninsured patients over 62 healthcare organisations (primary care, hospitals, and specialist providers) in the USA. This platform includes information about patients’ demographics, diagnoses (encoded as ICD-10-CM codes), medications, and procedures.

### Ethics

Data de-identification formally meets standards defined by section §164.514(b) (1) of the Health Insurance Portability and Accountability Act (HIPAA) privacy rule, superseding TriNetX's waiver from the Western Institutional Review Board; no further ethical approval was thus needed. As the data are routinely collected and fully anonymised, patient consent is not required. Further description of TriNetX US Collaborative Network database, including legal and ethical status, is in Appendix, pp 3–5.

### Cohorts

We identified people 18 years old and above with a diagnosis of T2DM (ICD-10-CM E11) within one month prior to a first prescription of semaglutide, and used the latter as the index event. This had to have occurred between 1st December 2017 (date at which semaglutide was licensed for T2DM in the USA) and 31st May 2021 (date at which it started being licensed for obesity too), in line with a prior study.^24^ Patients were included if they had had at least one healthcare encounter recorded at least one year before the index event to increase coverage of baseline characteristics. Comparator cohorts were created similarly after substituting semaglutide with three other common drugs approved by the US FDA for T2DM: the dipeptidyl peptidase 4 inhibitor (DPP4I) sitagliptin; the sodium-glucose transporter 2 inhibitor (SGLT2I) empagliflozin; and the sulphonylurea glipizide—as per a previous study.^22^ For each comparison, individuals exposed to either class of drugs being compared before their index events were excluded. Details of the cohorts’ construction are in Appendix, pp 6–8.

### Covariates

The study cohorts were matched for 179 covariates selected based on expert opinion and prior literature^24^ (i.e., suspected risk factors for both differences in choice of antidiabetic drug and outcomes), as well as statistical differences between cohorts before matching (i.e., variables whose baseline incidence was greater than 5% in both cohorts and the difference between cohorts was greater than 2% before matching). These included demographics, socioeconomic determinants of health, lifestyle factors, measures of healthcare utilisation, comorbidities, and concurrent or historical use of other medications. The full list of covariates with individual codes and further details regarding the choice of covariates are in Appendix, pp 9–14.

### Outcomes

In a time-to-event analysis, we assessed the risk of a diagnosis of 22 neurological and psychiatric outcomes occurring 1–365 days after the index event using ICD-10-CM codes (see detailed list with specific codes in Appendix, pp 15 and 16): encephalitis; parkinsonism; cognitive deficit; dementia; epilepsy/seizure; migraine; insomnia; nerve disorder; myoneural junction (MNJ)/muscle disease; intracranial haemorrhage (ICH); ischaemic stroke; alcohol misuse; opioid misuse; cannabis misuse; stimulants misuse; nicotine misuse; psychosis; bipolar disorder; depression; anxiety disorders; obsessive-compulsive disorder (OCD); suicidality. To assess for possible unmeasured confounding, we also estimated the risk of 15 negative control outcomes^25^ (NCOs, i.e., outcomes such as dog bite or wrist/hand fracture that are not expected to be affected by differences between choices of antidiabetic medications, but which might be affected by differences in healthcare use and other unmeasured confounders), both individually and as a composite outcome. We separately estimated the incidence of first diagnoses (i.e., excluding those who had received a specific diagnosis before the index event—primary analysis) and the incidence of any diagnosis (i.e., including individuals who had a diagnosis at any point before the index event and who had the diagnosis rerecorded again during the outcome period—secondary analysis), except for diagnoses that tend to be chronic (i.e., dementia, parkinsonism, and myoneural junction/muscle disease). All-cause mortality within the year since first prescription was also investigated as an exploratory analysis.

### Statistics

Propensity-score matching was achieved via the TriNetX interface to produce three pairs of 1:1 matched cohorts (one for each comparison) using a greedy nearest-neighbour algorithm with calliper distance of 0.1. For each characteristic, matching was considered successful if the standardised mean difference (SMD) between the cohorts was <0.1.^26^ All other analyses were conducted using R (version 4.2.1) and the survival package (version 3.3.1). The incidence of each outcome was estimated using Kaplan–Meier estimator. Comparisons between cohorts were made with a log-rank test. Hazard ratios (HRs) and 95% confidence intervals (CIs) were computed with a Cox proportional hazards model (HRs <1 correspond to a reduced risk after semaglutide than after the comparator drug, HRs >1 correspond to a higher risk after semaglutide than after the comparator drug). The proportional-hazard assumption was tested via the generalised Schoenfeld approach; if violated, time-varying HRs were assessed by fitting natural cubic splines to the log-cumulative hazard. Statistical significance was set at two-sided p-value <0.05; for the main analyses of the 22 neurological and psychiatric outcomes, we corrected for multiple testing within each drug comparison using Bonferroni correction (p_adj_), corresponding to a threshold of 0.0023 applied to unadjusted p-values. NCOs’ p-values were not adjusted to avoid spurious lacks of associations due to multiple comparisons adjustment.

We also conducted four further secondary analyses. First, we assessed results for study participants according to two age subgroups (<65 years old; ≥65 years old). Second, we stratified participants by year of index prescription (between 1st December 2017 and 31st December 2018; between 1st January 2019 and 31st December 2019; between 1st January 2020 and 31st May 2021). This robustness analysis also allowed us to assess for differences between those who had their index prescription before the COVID-19 pandemic (corresponding to the first two study periods) and after the beginning of the COVID-19 pandemic (corresponding to the last period). We reported HRs and p-values for all outcomes after pooling results from all time windows as well as in the pre-pandemic and in-pandemic periods. To assess whether results remained consistent between the pre- and in-pandemic periods, correlations in HRs were calculated across periods for outcomes that are significantly associated with semaglutide (at a nominal p < 0.05) in the pooled analysis. Third, to account for death as a competing risk and thus address survivorship bias, each outcome was analysed as part of a composite outcome with death as the other component. Fourth, we explored outcomes at a longer follow-up of 2 years.

We did not have a pre-specified statistical analysis plan, but followed that of prior similar work—further details of the statistical analyses are in Appendix, p 17.

### Role of the funding source

The funder(s) of the study had no role in study design, data collection, data analysis, data interpretation, or writing of the manuscript.

## Results

Following application of inclusion/exclusion criteria (see Appendix, pp 6–8), a total of 23,386 patients were included in each cohort (after matching) for the semaglutide *vs* sitagliptin comparison (mean ± SD age 56.6 ± 12.8 years, 48.6% female), 22,584 patients in each cohort for the empagliflozin comparison (mean ± SD age 57.6 ± 12.4 years, 48.9% female), and 19,206 patients in each cohort for the glipizide comparison (mean ± SD age 56.3 ± 13.0 years, 49.3% female)—see Table 1 for a summary of baseline characteristics and Appendix, pp 18–37 for all baseline characteristics before and after matching. Adequate propensity-score matching (SMD <0.1) was achieved for every comparison and covariate.

**Table 1:** Baseline characteristics of the study cohorts.

Characteristics	Semaglutide *vs* Sitagliptin		Semaglutide *vs* Empagliflozin		Semaglutide *vs* Glipizide	
**Cohort size**	23,386	23,386	22,584	22,584	19,206	19,206
**Age** (at index event)						
Years [mean (SD)]	56.7 (12.2)	56.6 (13.3)	57.6 (12.3)	57.6 (12.4)	56.4 (12.4)	56.2 (13.6)
**Sex**						
Female	11,411 (48.8%)	11,317 (48.4%)	11,067 (49.0%)	11,012 (48.8%)	9440 (49.2%)	9497 (49.4%)
Male	10,183 (43.5%)	10,269 (43.9%)	9638 (42.7%)	9690 (42.9%)	8150 (42.4%)	8104 (42.2%)
Unknown	1792 (7.7%)	1800 (7.7%)	1879 (8.3%)	1882 (8.3%)	1616 (8.4%)	1605 (8.4%)
**Ethnic group**						
Black/African-American	3461 (14.8%)	3503 (15.0%)	3546 (15.7%)	3544 (15.7%)	2938 (15.3%)	2963 (15.4%)
White	14,137 (60.5%)	14,117 (60.4%)	13,480 (59.7%)	13,533 (59.9%)	11,526 (60.0%)	11,486 (59.8%)
Unknown	3684 (15.8%)	3658 (15.6%)	3585 (15.9%)	3569 (15.8%)	2966 (15.4%)	2941 (15.3%)
**Comorbidities** (any time before the index event)						
Overweight/obesity	13,865 (59.3%)	13,920 (59.5%)	13,850 (61.3%)	13,895 (61.5%)	11,245 (58.5%)	11,287 (58.8%)
Thyroid disorders	5541 (23.7%)	5501 (23.5%)	5697 (25.2%)	5610 (24.8%)	4480 (23.3%)	4518 (23.5%)
CV disorders	19,858 (84.9%)	19,837 (84.8%)	19,511 (86.4%)	19,592 (86.8%)	16,161 (84.1%)	16,147 (84.1%)
Renal disorders	4919 (21.0%)	4854 (20.8%)	5267 (23.3%)	5321 (23.6%)	4014 (20.9%)	3957 (20.6%)
Neurological disorders	14,950 (63.9%)	14,944 (63.9%)	14,856 (65.8%)	14,947 (66.2%)	12,156 (63.3%)	12,141 (63.2%)
Psychiatric disorders	12,206 (52.2%)	12,188 (52.1%)	12,027 (53.3%)	12,092 (53.5%)	9935 (51.7%)	9981 (52.0%)
Chronic pain	5642 (24.1%)	5743 (24.6%)	5745 (25.4%)	5809 (25.7%)	4492 (23.4%)	4457 (23.2%)
Infectious diseases	9439 (40.4%)	9409 (40.2%)	9549 (42.3%)	9586 (42.4%)	7573 (39.4%)	7607 (39.6%)
Neoplasms	8167 (34.9%)	8118 (34.7%)	8402 (37.2%)	8489 (37.6%)	6816 (35.5%)	6810 (35.5%)
Blood disorders	7703 (32.9%)	7691 (32.9%)	7865 (34.8%)	7936 (35.1%)	6075 (31.6%)	6056 (31.5%)
**Other medications** (any time before the index event)						
Insulin	12,270 (52.5%)	12,328 (52.7%)	12,195 (54.0%)	12,240 (54.2%)	9788 (51.0%)	9828 (51.2%)
Metformin	17,433 (74.5%)	17,638 (75.4%)	17,025 (75.4%)	17,154 (76.0%)	13,767 (71.7%)	13,943 (72.6%)
Beta-blockers	10,152 (43.4%)	10,107 (43.2%)	10,227 (45.3%)	10,247 (45.4%)	8052 (41.9%)	8028 (41.8%)
CCBs	7504 (32.1%)	7509 (32.1%)	7667 (33.9%)	7765 (34.4%)	5926 (30.9%)	5904 (30.7%)
RAAS inhibitors	15,166 (64.9%)	15,135 (64.7%)	15,118 (66.9%)	15,203 (67.3%)	11,927 (62.1%)	11,980 (62.4%)
Statins	16,549 (70.8%)	16,620 (71.1%)	16,442 (72.8%)	16,520 (73.1%)	13,209 (68.8%)	13,292 (69.2%)
Corticosteroids	13,570 (58.0%)	13,647 (58.4%)	13,570 (60.1%)	13,669 (60.5%)	10,999 (57.3%)	11,010 (57.3%)
Thyroid agents	3457 (14.8%)	3440 (14.7%)	3467 (15.4%)	3451 (15.3%)	2847 (14.8%)	2842 (14.8%)
Anti-infectives	18,415 (78.7%)	18,398 (78.7%)	18,146 (80.3%)	18,207 (80.6%)	14,974 (78.0%)	14,937 (77.8%)
Antineoplastics	2537 (10.8%)	2588 (11.1%)	2614 (11.6%)	2619 (11.6%)	2022 (10.5%)	2047 (10.7%)
Anti-inflammatories	12,410 (53.1%)	12,432 (53.2%)	12,294 (54.4%)	12,410 (55.0%)	9803 (51.0%)	9896 (51.5%)
Opioids	12,628 (54.0%)	12,668 (54.2%)	12,631 (55.9%)	12,685 (56.2%)	10,103 (52.6%)	10,179 (53.0%)
Antimigraine	1888 (8.1%)	1873 (8.0%)	1890 (8.4%)	1953 (8.6%)	1506 (7.8%)	1543 (8.0%)
Antiepileptics	7809 (33.4%)	7896 (33.8%)	7803 (34.6%)	7894 (35.0%)	6155 (32.0%)	6207 (32.3%)
Antipsychotics	3399 (14.5%)	3404 (14.6%)	3445 (15.3%)	3471 (15.4%)	2674 (13.9%)	2703 (14.1%)
Hypnotics/sedatives	9686 (41.4%)	9742 (41.7%)	9770 (43.3%)	9859 (43.7%)	7751 (40.4%)	7779 (40.5%)
SSRIs	5465 (23.4%)	5435 (23.2%)	5397 (23.9%)	5427 (24.0%)	3776 (19.7%)	3851 (20.1%)
Other antidepressants	5736 (24.5%)	5758 (24.6%)	5696 (25.2%)	5769 (25.5%)	4316 (22.5%)	4374 (22.8%)

For each comparison, the left column corresponds to the semaglutide cohort, and the right column corresponds to the comparator cohort. Values are N (%) unless stated otherwise. Only main characteristics with a prevalence higher than 5% in the whole population are displayed—full baseline characteristics are provided in the Appendix, pp 18–37. CCBs, calcium-channel blockers; CV, cardiovascular; RAAS, renin-angiotensin-aldosterone system; SD, standard deviation; SMD, standardised mean difference; SSRIs, selective-serotonin reuptake inhibitors.

**Fig. 1:**
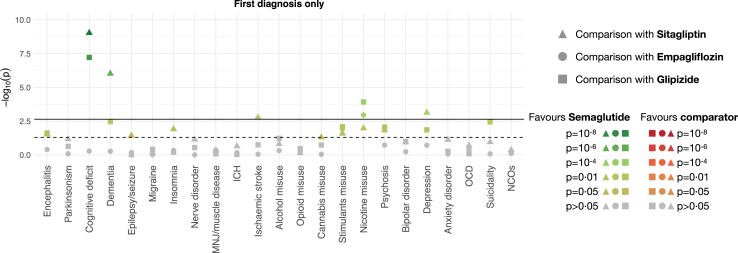
Negative logarithm of the p-values for the risks of neurological, psychiatric, and negative control outcomes (first diagnosis) in the year after semaglutide compared to three other antidiabetic medications. The horizontal solid line represents the threshold (p < 0.0023) above which associations are statistically significant after Bonferroni correction. The dashed line represents the threshold above which associations are statistically significant at the nominal p < 0.05 level. Colour shades encode the p-values with green shades favouring semaglutide and red shades favouring the comparator. The same figure for any diagnosis can be found in the Appendix, p 95. ICH, intracranial haemorrhage; MNJ, myoneural junction; NCOs, composite negative control outcomes; OCD, obsessive-compulsive disorder.

The risks of neurological and psychiatric outcomes for first diagnoses are summarised in Fig. 1 and Table 2 (cumulative incidences are in Appendix, pp 38–42).

**Table 2:** Hazard ratios (95% confidence interval) of first diagnosis for all outcomes in the year after semaglutide compared to sitagliptin, empagliflozin, and glipizide.

Outcomes	Semaglutide *vs* Sitagliptin	Semaglutide *vs* Empagliflozin	Semaglutide *vs* Glipizide
Encephalitis	0.35 (0.13–0.96)	0.62 (0.20–1.89)	0.26 (0.074–0.95)
Parkinsonism	0.63 (0.39–1.04)	1.06 (0.64–1.76)	0.71 (0.41–1.24)
Cognitive deficit	0.72 (0.64–0.80)	0.96 (0.86–1.08)	0.72 (0.63–0.81)
Dementia	0.52 (0.40–0.68)	0.91 (0.69–1.21)	0.63 (0.46–0.86)
Epilepsy/seizure	0.77 (0.61–0.98)	0.94 (0.73–1.22)	1.00 (0.76–1.31)
Migraine	0.96 (0.80–1.15)	1.01 (0.83–1.22)	1.10 (0.89–1.36)
Insomnia	0.86 (0.77–0.97)	0.96 (0.85–1.08)	0.97 (0.85–1.10)
Nerve disorder	0.89 (0.79–1.01)	1.00 (0.88–1.14)	0.93 (0.81–1.06)
MNJ/muscle disease	0.87 (0.61–1.25)	0.87 (0.61–1.24)	0.96 (0.65–1.42)
ICH	0.80 (0.56–1.15)	0.93 (0.65–1.33)	1.02 (0.67–1.55)
Ischaemic stroke	0.75 (0.62–0.90)	0.99 (0.81–1.19)	0.87 (0.70–1.07)
Alcohol misuse	0.82 (0.62–1.08)	0.90 (0.67–1.20)	0.74 (0.54–1.01)
Opioid misuse	0.95 (0.71–1.27)	0.94 (0.70–1.26)	0.86 (0.63–1.18)
Cannabis misuse	0.73 (0.53–1.00)	1.03 (0.72–1.45)	0.78 (0.54–1.13)
Stimulants misuse	0.59 (0.36–0.95)	0.51 (0.30–0.87)	0.52 (0.32–0.85)
Nicotine misuse	0.82 (0.70–0.95)	0.77 (0.65–0.90)	0.72 (0.61–0.85)
Psychosis	0.63 (0.44–0.92)	0.77 (0.52–1.14)	0.59 (0.39–0.88)
Bipolar disorder	0.79 (0.59–1.06)	0.91 (0.66–1.26)	0.76 (0.55–1.06)
Depression	0.84 (0.75–0.93)	0.93 (0.83–1.04)	0.86 (0.77–0.97)
Anxiety disorder	0.92 (0.84–1.01)	0.99 (0.90–1.09)	0.97 (0.88–1.07)
OCD	0.68 (0.37–1.24)	0.76 (0.39–1.45)	0.92 (0.47–1.79)
Suicidality	0.75 (0.52–1.07)	0.96 (0.66–1.40)	0.54 (0.36–0.83)
NCOs	0.97 (0.88–1.05)	0.99 (0.90–1.08)	1.03 (0.93–1.13)
All–cause mortality	0.58 (0.50–0.66)	0.86 (0.75–0.99)	0.55 (0.47–0.64)

Hazard ratios (HRs) <1 indicate that risk is lower after semaglutide than after the comparator drug, HRs >1 indicate that risk is higher after semaglutide than after the comparator drug. HRs for any diagnosis, as well as p–values and cumulative incidences for all outcomes are provided in the Appendix, pp 38–42. ICH, intracranial haemorrhage; MNJ, myoneural junction; NCOs, negative control outcomes; OCD, obsessive-compulsive disorder.

**Fig. 2:**
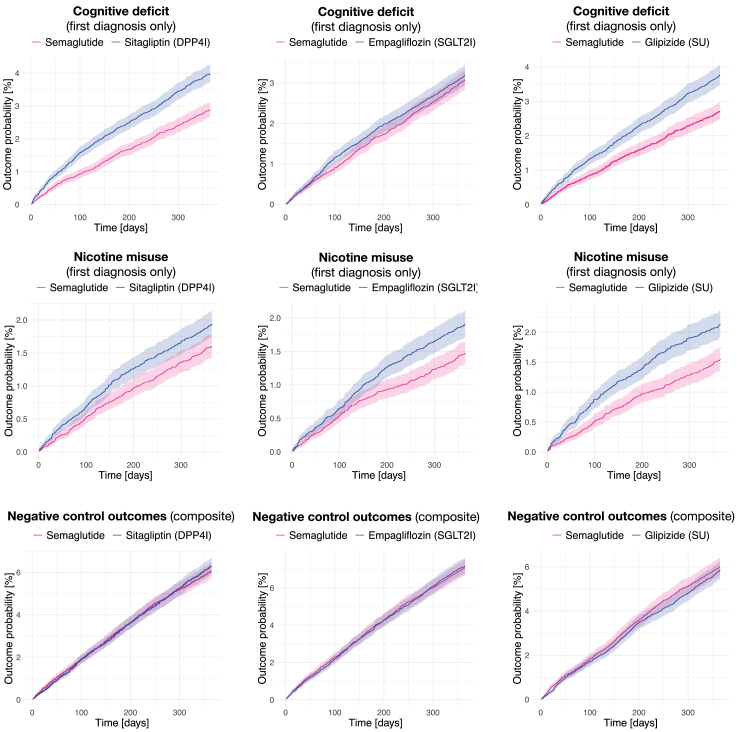
Kaplan–Meier estimates for the incidence of cognitive deficit, nicotine misuse, and negative control outcomes across comparisons. Shaded areas are 95% confidence intervals. For diagnostic subcategories of cognitive deficit, see the Appendix, pp 43 and 44. DPP4I, dipeptidyl peptidase 4 inhibitor; SGLT2I, sodium-glucose transporter 2 inhibitor; SU, sulphonylurea.

Semaglutide was not associated with any increased risk of a first diagnosis of neurological or psychiatric condition. Conversely, semaglutide was associated with a lower risk of several neurological and psychiatric outcomes. Two outcomes are noteworthy (Fig. 2). First, the risk of cognitive deficits was significantly lower after semaglutide compared to sitagliptin (HR 0.72, 95% CI 0.64–0.80, p_adj_ < 0.0001) and glipizide (HR 0.72, 95% CI 0.63–0.81, p_adj_ < 0.0001), but similar between semaglutide and empagliflozin (HR 0.96, 95% CI 0.86–1.08, p_adj_ 0.51). Similarly, the risk of dementia was lower after semaglutide compared to sitagliptin (HR 0.52, 95% CI 0.40–0.68, p_adj_ < 0.0001) and glipizide (HR 0.63, 95% CI 0.46–0.86, although this did not reach significance after correction, p_adj_ 0.075), but similar between semaglutide and empagliflozin (HR 0.91, 95% CI 0.69–1.21, p_adj_ 0.53). Second, the risk of nicotine dependence was found to be significantly lower after semaglutide compared to glipizide (HR 0.72, 95% CI 0.61–0.85, p_adj_ 0.0027) and empagliflozin (HR 0.77, 95% CI 0.65–0.90, p_adj_ 0.024), and lower compared to sitagliptin, although not after correction for multiple testing (HR 0.82, 95% CI 0.70–0.95, p_adj_ 0.23). Other significant results include a lower risk of a first diagnosis of depression and ischaemic stroke compared to sitagliptin.

Analyses of individual and composite NCOs consistently showed no difference for any of the comparisons made (HRs for composite NCOs between 0.97 and 1.03, all uncorrected p-values >0.4—see Fig. 2).

The risk of all-cause mortality was lower after semaglutide compared to sitagliptin (HR 0.58, 95% CI 0.50–0.66, p < 0.0001), glipizide (HR 0.55, 95% CI 0.47–0.64, p < 0.0001), and empagliflozin (HR 0.86, 95% CI 0.75–0.99, p 0.035).

Results were similar when the outcomes included both first and recurrent diagnoses (see Appendix, pp 38–42 and p 95). In particular, the association of semaglutide with significantly lower risk of cognitive deficits (compared to sitagliptin and glipizide) and nicotine misuse (compared to all three comparator drugs) held true. One exception was an increased risk of any diagnosis of migraine for semaglutide compared to glipizide (HR 1.20, 95% CI 1.08–1.33, p_adj_ 0.015).

Other secondary analyses supported the robustness of the findings above. Results according to age group closely mirrored those of the primary analysis (see Appendix, pp 45–61 and p 96), though with stronger associations between semaglutide and lower cognitive deficit and dementia in people ≥65 years old, and between semaglutide and lower substance use disorders in those <65 years old. As semaglutide was associated with a lower risk of both death and several outcomes, the risks of the composite of death with each outcome were also significantly lower in those receiving semaglutide (see Appendix, pp 62–63 and p 106). Results were very similar when the analysis was stratified by time of the index prescription (see Appendix, pp 64–90 and p 98). In addition, for outcomes that were found to be significantly associated with semaglutide, the HRs in the period before the COVID-19 pandemic strongly correlated with those in the pooled analysis (Pearson's r 0.84, p-value <0.0001), and so did the HRs measured during the pandemic (Pearson's r 0.93, p-value <0.0001). Finally, results remained broadly consistent at 2-year follow-up, with an additional potential association with lower risk of psychosis for semaglutide compared to sitagliptin and glipizide (see Appendix, pp 91 and 92 and p 99).

There was no evidence of violation of the proportional-hazard assumption for most comparisons with a few exceptions (see Appendix, pp 93 and 94 for all p-values related to tests of the proportionality assumption, and pp 140–142 for time-varying hazard ratios when the assumption was violated). For instance, the HR for cognitive deficit in the comparison between semaglutide and sitagliptin appears more pronounced earlier in the follow-up and progressively wanes (but remains <1) towards the 12-month endpoint.

## Discussion

Various beneficial and harmful neuropsychiatric effects of semaglutide have been described or are under investigation.^13^, ^14^, ^15^, ^16^, ^17^, ^18^^,^^24^ Here, we provide the first large-scale real-world data on the neurological and psychiatric outcomes within 12-months of a first dose of semaglutide in patients with T2DM. We found no evidence of an increased risk of 22 neurological and psychiatric outcomes following a first prescription of semaglutide compared to other antidiabetic agents (except for an increased risk of migraine compared to glipizide), and a possible protective association for cognition and nicotine misuse.

The observation that semaglutide use was associated with lower risks of cognitive deficits and, to some extent, dementia than the DPP4I sitagliptin and the sulphonylurea glipizide, but a similar risk compared to the SGLT2I empagliflozin aligns with a recent network meta-analysis showing that SGLT2Is and GLP1-RAs rank better than DPP4Is and sulphonylureas in terms of cognitive outcomes and dementia.^17^ In this meta-analysis, however, only a few studies (i.e., one RCTs and small case–control studies) were available for GLP1-RAs, and none involved semaglutide.^17^ Data from the present study thus confirm these preliminary observations of a possible class effect of GLP1-RAs on cognition, and show that they also apply to semaglutide. The underlying mechanisms remain to be determined. One possibility is that any protective effect of GLP1-RAs on cognition is mediated by their effect on cardiovascular morbidity.^7^ However, the lack of consistent association with ischaemic strokes suggests that other mechanisms might also be at play. These might include other neuroprotective and anti-inflammatory mechanisms,^9^, ^10^, ^11^ supported by measurements of systemic inflammation in people exposed to semaglutide.^27^ As these are partly shared with SGLT2I,^28^ this hypothesis would also explain why no difference in cognitive outcomes were observed when semaglutide was compared with the SGLT2I empagliflozin. Clinical trials including biomarkers of disease progression and longer follow-ups can help to further define these key potential mechanisms of action.

The association of semaglutide with lower risks of nicotine use disorder was robust and consistent across comparisons. This might reflect that people prescribed semaglutide are fundamentally different in their risk of nicotine misuse that was not captured in the propensity-score matching. Alternatively, it is possible that, unlike other antidiabetic agents, GLP1-RAs regulate dopaminergic pathways underlying reward sensitivity, which are at least partly responsible for their weight-loss action and their putative activity against addictive behaviour.^9^^,^^12^ The same reasoning could apply to other substance use disorders (e.g., stimulant misuse), for which however we only observed trend associations that did not hold against adjustment for multiple comparisons. Further research is therefore needed to investigate the potential role of semaglutide in the treatment of addictions.^15^

That semaglutide was not associated with an increased risk for any psychiatric outcomes is important. The lack of psychiatric adverse outcomes (and corresponding safety signals) is highly relevant to the care of patients with T2DM, who have a high burden of mental illness,^29^ while also providing reassurance at a general population level.^20^ Specifically, the absence of negative associations between semaglutide use and depression or suicidality can inform current investigations by the EMA, MHRA, and FDA,^18^^,^^19^ although ongoing pharmacovigilance as well as studies in other at-risk subgroups (e.g., obesity) are recommended. Our findings for suicidality corroborate those from previous studies based on EHR data.^24^ Also, an antidepressant effect of GLP1-RAs had been suggested by a recent meta-analysis,^13^ but this was not confirmed by our data. While this might be due to the small sample sizes and lack of tight control on covariates in the studies included in the meta-analysis, it is also possible that the antidepressant effect is only observed in those with established depression (with no effect on incident depression), or that it only applies to other GLP1-RAs (since semaglutide was not included in the meta-analysis).

The lower all-cause mortality for semaglutide than for all three comparators is striking, but must be interpreted cautiously since linkage between the TriNetX US Collaborative Network and death registry is incomplete, and previous studies had suggested similar mortality rates between GLP1-RAs and SGLT2I.^30^ If confirmed, a protective effect of semaglutide on mortality would be highly relevant for public health. It might in part be mediated by the association of semaglutide with lower cognitive and addictive burden, as well as with protective effect of semaglutide on other bodily systems.^7^^,^^8^

The large sample size, comparisons across a range of other antidiabetic agents, and extensive propensity-score matching contribute to the strength of our study. Further, results remained robust to extensive secondary analyses. However, this study also has several limitations. As for all analyses of EHR, it is subject to under-coding/coding errors (including potential misdiagnosis) and unknown completeness of data (see also Appendix, pp 3–5). The same illness episode may have been coded multiple times in different settings so that ‘recurrent’ diagnoses might not reflect relapses; as such, we consider incidences of first diagnoses to be more reliable and easier to interpret. We could not assess patients' medication adherence nor the duration of exposure as this information is not available in their records. For privacy reasons, the identity of healthcare organisations could not be used in the analyses so that within-hospital correlation could not be assessed. Because most individuals receiving semaglutide were matched to individuals taking a comparator drug but not vice versa, the hazard ratios reported in this paper should be seen as estimates of the average treatment effect on the treated, rather than the average treatment effect. Residual and unmeasured confounding remains a possibility, especially due to the paucity of information on socioeconomic determinants of health and lifestyle factors. However, the consistent lack of any association for the numerous NCOs measured across all comparisons (see Fig. 2 and Appendix, pp 38–42) provides reassurance in this regard. Additionally, the observed associations might be partly mediated by better diabetes control as measured by HbA1c, but this was not tested here. Because this study is observational, it cannot be demonstrated that the observed outcomes are adverse events of medications. We could not differentiate between formulations (i.e., oral *vs* subcutaneous)^4^ and doses of semaglutide, as such information is not well recorded in the data. Finally, our results do not necessarily extend to other GLP-1RAs (e.g., liraglutide, dulaglutide) nor to the upcoming dual- and triple-agonists (e.g., tirzepatide, retatutride),^5^ as different brain penetrance and pathophysiological mechanisms might be involved.

In summary, semaglutide use is not associated with higher risks of onset or recurrence of neurological and psychiatric outcomes in the following 12 months compared to other commonly prescribed antidiabetic agents. In contrast, semaglutide has a potential benefit on cognition and nicotine misuse. Our findings can inform current regulatory investigations and public health, and stimulate clinical trials to test the role of semaglutide for the treatment and prevention of cognitive deficits and substance misuse.

## Contributors

RDG, MT, and IK conceived the study. RDG and MT developed the study methodology, collected the data, conducted the study analyses, and investigated its findings. IK, AA, PJC, CJH, and PJH validated the data, supported with the interpretation of the findings, and supervised the overall project. RDG drafted the manuscript and tables, MT designed the figures. All authors critically revised the manuscript and approved the final version. All authors had full access to all the data in the study and accept responsibility to submit for publication.

## Data sharing statement

The TriNetX US Collaborative Network returned the results of these analyses as csv files, which we downloaded and archived. Aggregate data, as presented in this paper, is fully provided in the Appendix. Data presented in this article were acquired from TriNetX. This study had no special privileges. Eligibility criteria specified in the Methods and Supplementary Material would allow other researchers to identify similar cohorts of patients as used for our analyses; however, TriNetX is a live platform with new data being added daily so exact counts will vary. To gain access to the data, a request can be made to TriNetX (join@trinetx.com), but costs might be incurred, and a data sharing agreement would be necessary.

## Declaration of interests

RDG, IK, CJH, PJC, PJH are supported by the NIHR Oxford Health Biomedical Research Centre (NIHR203316). MT is an NIHR clinical lecturer. AIA is supported by the NIHR Oxford Biomedical Research Centre. IK declares additional funding for this work through the Medical Research Council (MR/T033371/1), and NIHR Development and Skills Enhancement Award (NIHR301616). IK is in receipt of grant funding from Novo Nordisk for an investigator-initiated study of semaglutide in Alzheimer's disease; he is paid a medical advisor for digital healthcare companies in the dementia space (Five Lives SAS, Cognetivity Ltd, Cognes Ltd). PJC declares additional funding for this work through the Medical Research Council (MR/K022202). CJH has received consultancy fees from P1vital, Lundbeck, Servier, UCB, Zogenix, J&J, and Syndesi outside of the current work. The other authors declare that they have no conflict of interest.
