# Supplementary material for: 12-month neurological and psychiatric outcomes of semaglutide use for type 2 diabetes: a propensity-score matched cohort study
Source: eClinicalMedicine. 2024 Jul 10;74:102726. doi: 10.1016/j.eclinm.2024.102726 (PMC11701436; doi:10.1016/j.eclinm.2024.102726)
Supplement: Appendix [file mmc1.pdf]

APPENDIX – 12-month neurological and psychiatric outcomes of semaglutide use for type 2 diabetes: a propensity-score matched cohort study

Riccardo De Giorgi[1,2,*] (DPhil), Ivan Koychev[1,3] (PhD), Prof Amanda I Adler[4] (PhD), Prof Philip J Cowen[1,2] (MD), Prof Catherine J Harmer[1,2] (PhD), Prof Paul J Harrison[1,2] (FRCPsych), Maxime Taquet[1,2] (PhD)

[1] Department of Psychiatry, University of Oxford, Warneford Hospital, Warneford Lane, Oxford, OX3 7JX, United Kingdom

[2] Oxford Health NHS Foundation Trust, Warneford Hospital, Warneford Lane, Oxford, OX3 7JX, United Kingdom

[3] Department of Psychological Medicine, John Radcliffe Hospital, Headley Way, Oxford, OX3 9DU, United Kingdom

[4] Diabetes Trials Unit, Oxford Centre for Diabetes, Endocrinology and Metabolism, University of Oxford, Churchill Hospital, Oxford, OX3 7LE, United Kingdom

[*] Correspondence to:
Riccardo De Giorgi
Address: University of Oxford, Department of Psychiatry, Warneford Hospital, Warneford Lane, Oxford, OX3 7JX, United Kingdom.
Email: riccardo.degiorgi@psych.ox.ac.uk
Phone: +44 (0) 1865 618238

Table of Contents

SUPPLEMENTARY METHODS *3*

Database **3**

Cohorts' construction **6**

Semaglutide vs Sitagliptin (DPP4I) comparison 6
Semaglutide vs Empagliflozin (SGLT2I) comparison 6
Semaglutide vs Glipizide (SU) comparison 7
Summary table of the study flow chart 7

List of covariates (including TriNetX codes) **9**

List of outcomes (including ICD-10-CM codes) **15**

Neurological and psychiatric outcomes 15
Negative control outcomes (NCO) 16
Other outcomes 16

Further details of statistical analyses **17**

Statistical analysis plan 17
Implementation details of propensity score matching 17
Censoring 17
Estimating time-varying hazard ratios 17

SUPPLEMENTARY TABLES *18*

Supplementary Table S1. Baseline characteristics before matching 18
Supplementary Table S2. Baseline characteristics after matching 29
Supplementary Table S3. Full study results 38
Supplementary Table S4. Study results for diagnostic subcategories of cognitive deficit and dementia 43
Supplementary Table S5. Secondary analysis, <65yo; ≥65yo 45
Supplementary Table S6. Secondary analysis, death as composite outcome, study results 62
Supplementary Table S7. Secondary analysis, between 1st December 2017 and 31st December 2018; between 1st January 2019 and 31st December 2019; between 1st January 2020 and 31st May 2021 64
Supplementary Table S8. Secondary analysis, 2-year follow-up 91
Supplementary Table S9. Tests of the proportionality assumption (p-values) 93

SUPPLEMENTARY FIGURES *95*

Supplementary Figure S1. Negative logarithm of the p-values for the risks of neurological, psychiatric, and negative control outcomes (any diagnosis) in the year after semaglutide compared to three other antidiabetic medications 95
Supplementary Figure S2. Negative logarithm of the p-values for the risks of neurological, psychiatric, and negative control outcomes (first diagnosis) for <65yo and ≥ 65yo in the year after semaglutide compared to three other antidiabetic medications 96
Supplementary Figure S3. Negative logarithm of the p-values for the composite risks of death in addition to neurological and psychiatric outcomes (first diagnosis) in the year after semaglutide compared to three other antidiabetic medications 97
Supplementary Figure S4. Negative logarithm of the p-values for the risks of neurological, psychiatric, and negative control outcomes (first diagnosis) in the year after semaglutide compared to three other antidiabetic medications, adjusted for time of the index event (using pooling of results stratified in three time windows: between 1st December 2017 and 31st December 2018; between 1st January 2019 and 31st December 2019; between 1st January 2020 and 31st May 2021). 98
Supplementary Figure S5. Negative logarithm of the p-values for the risks of neurological, psychiatric, and negative control outcomes (first diagnosis) in the two years after semaglutide compared to three other antidiabetic medications. 99
Supplementary Figure S6. Kaplan-Meier estimates for all outcomes across comparisons. 100
Supplementary Figure S6. Time-varying hazard ratios 140

SUPPLEMENTARY REFERENCES *143*

SUPPLEMENTARY METHODS

Database

This section largely replicates our previous descriptions of the network[1].

Legal and ethical status

TriNetX US Collaborative Network is compliant with the Health Insurance Portability and Accountability Act (HIPAA), the US federal law which protects the privacy and security of healthcare data. TriNetX is certified to the ISO 27001:2013 standard and maintains an Information Security Management System (ISMS) to ensure the protection of the healthcare data it has access to and to meet the requirements of the HIPAA Security Rule. Data are de-identified and displayed on the TriNetX Platform in aggregate form. The process by which the data is de-identified is attested to through a formal determination by a qualified expert as defined in Section §164.514(b)(1) of the HIPAA Privacy Rule. This formal determination by a qualified expert, refreshed in December 2020, supersedes TriNetX's previous waiver from the Western Institutional Review Board (IRB). The network contains data that are provided by participating Health Care Organizations (HCOs), each of which represents and warrants that it has all necessary rights, consents, approvals, and authority to provide the data to TriNetX under a Business Associate Agreement (BAA), so long as their name remains anonymous as a data source and their data are utilized for research purposes. The data shared through the TriNetX Platform are attenuated to ensure that they do not include sufficient information to facilitate the determination of which HCO contributed which specific information about a patient.

Acquisition of data, quality control, and other procedures

The data are stored onboard a TriNetX appliance – a physical server residing at the institution's data centre or a virtual hosted appliance. The TriNetX platform is a fleet of these appliances connected into a federated network able to broadcast queries to each appliance. Results are subsequently collected and aggregated.

Once the data are sent to the network, they are mapped to a standard and controlled set of clinical terminologies and undergo a data quality assessment including 'data cleaning' that rejects records which do not meet the TriNetX quality standards. Different data modalities are available in the network. They include demographics (coded to HL7 version 3 administrative standards), diagnoses (represented by ICD-10-CM codes), procedures (coded in ICD-10-PCS or CPT), and measurements (coded to LOINC). While extensive information is provided about patients' diagnoses and procedures, other variables (such as socioeconomic and lifetime factors) are not comprehensively represented.

The data from a typical HCO generally go back around 7 years, with some going back 13 years. The data are continuously updated. HCOs update their data at various times, with most refreshing every 1, 2, or 4 weeks.

Data quality assessment followed a standardised strategy wherein the data are reviewed for conformance (adherence to specified standards and formats), completeness (quantifying data presence or absence) and plausibility

(believability of the data from a clinical perspective). There are pre-defined metrics for each of the above assessment categories. Results for these metrics are visualised and reviewed for each new site that joins the network as well as on an ongoing basis. Any identified issue is communicated to the data provider and resolved before continuing data collection.

The basic formatting of contributed data is also checked (e.g. to ensure that dates are properly represented). Records are checked against a list of required fields (e.g., patient identifier) and records for which the required information is missing are rejected. Referential integrity checking is done to ensure that data spanning multiple database tables can be successfully joined together. As the data are refreshed, changes in volume of data over time is monitored to ensure data validity. At least one non-demographic fact for each patient is required for them to be counted in the dataset. Patient records with only demographics information are discarded.

The software also undergoes quality control. The engineers testing the software are independent from the engineers developing it. Each test code is checked by two independent testing engineers. Each piece of software is tested extensively against a range of synthetic data (i.e. generated for the purpose of testing) for which the expected output is established independently. If the software fails to return this output, then the software is deemed to have failed the test and is examined and modified accordingly. For statistical software (including that used for propensity score matching, for Kaplan-Meier analysis, etc), an additional quality control step is implemented. Two independent codes are written in two different programming languages (typically R and python) and the statistical results are compared. If discrepancies are identified, then the codes are deemed to have failed the test and are examined and modified accordingly. All the code is reviewed independently by another engineer.

The test strategy follows three levels of granularity:
1. Unit tests: These test specific blocks, or units, of code that perform specific actions (e.g. querying the database).
2. Integration tests: These ensure that different components are working together correctly.
3. End-to-end tests: These tests run the entire system and check the final output.

Some comments on missing data

TriNetX carries out an intensive data pre-processing to minimise missing values. As noted in other studies,[2] TriNetX maps the data to a consistent clinical data model with a consistent semantic meaning so that the data can be queried consistently regardless of the underlying data source(s). All covariates are either binary, categorical (which is expanded to a set of binary columns), or continuous but essentially guaranteed to exist. As for demographic variables, age is guaranteed to exist. Missing sex, ethnicity and/or race are coded using their own category (i.e., 'unknown sex', 'unknown ethnicity', and 'unknown race'). The decision to code these within their own category was made because they might in part represent individuals with mixed race/mixed ethnicity/who do not want a binary sex recorded, or individuals who receive their care in a place where coding is less robust, and both explanations might be relevant confounding factors that we therefore want to match cohorts for. For other variables, including medical conditions, procedures, laboratory tests and socioeconomic determinant health, the value is either present or absent, so 'missing' is not pertinent.

Some comments on advantages and disadvantages of EHR data

One advantage of EHR data, like those in TriNetX, over insurance claim data is that both insured and uninsured patients are included. However, there are also limitations inherent to research of this kind using electronic health records, including TriNetX – see Casey et al., 2016[3]; Cowie et al., 2017[4]; Jetley and Zhang, 2019[5]:

1. Despite the matching and use of various comparison cohorts, there may be residual confounding, particularly related to social and economic factors which are not well captured in EHR networks, and which might influence outcomes.
2. We do not know which diagnoses were made in primary or secondary care or specialist facilities, nor by whom.
3. A patient may be seen in different HCOs for different parts of their care, and if one HCO is not part of the federated network then part of their medical records may not be available. Using a network of HCOs (rather than a single HCO) limits this possibility but does not eliminate it.
4. The severity and persistence of diagnoses is difficult to assess using EHR data as this is not typically coded. As a result, we can comment on incidence of new cases but cannot assess the duration of illness.
5. We are measuring the date of diagnosis, not the date at which diagnostic criteria were first met. This time difference may well differ between diagnostic categories, and potentially between exposure and comparator cohorts.
6. Since the data are presented as they are recorded, we cannot be sure that there has not been mis-recording of information, adding a degree of noise to the data.
7. Historical data before the start of EHRs (or the addition of an HCO to the network) may well be incomplete. For example, a previous psychiatric or neurologic diagnosis may not have been recorded.

Cohorts' construction

All codes are RxNorm codes used on TriNetX.

Semaglutide vs Sitagliptin (DPP4I) comparison

Semaglutide cohort [N = 687,980]

All patients meeting all of the following criteria were included:

A. A first prescription of semaglutide (1991302) between Dec 01 2017 and May 31 2021. [N = 111,607]

B. No prescription of DPP4I (1100699 linagliptin, 1368001 alogliptin, 593411 sitagliptin, 596554 vildagliptin, 857974 saxagliptin) or other GLP1-RAs (1440051 lixisenatide, 1534763 albiglutide, 1551291 dulaglutide, 475968 liraglutide, 60548 exenatide) before event A. [N = 61,449]

C. A diagnosis of T2DM recorded within 1 month before and up to the same day as event A. [N = 29,722]`

D. Any healthcare visit at least one year before event A. [N = 24,626]

Sitagliptin (DPP4I) cohort [N = 707,902]

All patients meeting all of the following criteria were included:

A. A first prescription of sitagliptin (593411) between Dec 01 2017 and May 31 2021. [N = 307,781]

B. No prescription of GLP1-RAs (1440051 lixisenatide, 1534763 albiglutide, 1551291 dulaglutide, 1991302 semaglutide, 475968 liraglutide, 60548 exenatide) or other DPP4I (1100699 linagliptin, 1368001 alogliptin, 596554 vildagliptin, 857974 saxagliptin) before event A. [N = 263,013]

C. A diagnosis of T2DM recorded within 1 month before and up to the same day as event A. [N = 135,944]

D. Any healthcare visit at least one year before event A. [N = 104,541]

Semaglutide vs Empagliflozin (SGLT2I) comparison

Semaglutide cohort [N = 687,980]

All patients meeting all of the following criteria were included:

A. A first prescription of semaglutide (1991302) between Dec 01 2017 and May 31 2021. [N = 107,157]

B. No prescription of SGLT2I (1373458 canagliflozin, 1488564 dapagliflozin, 1545653 empagliflozin, 1992672 ertugliflozin, 2638675 sotagliflozin) or other GLP1-RAs (1440051 lixisenatide, 1534763 albiglutide, 1551291 dulaglutide, 475968 liraglutide, 60548 exenatide) before event A. [N = 57,539]

C. A diagnosis of T2DM recorded within 1 month before and up to the same day as event A. [N = 28,355]

D. Any healthcare visit at least one year before event A. [N = 24,006]

Empagliflozin (SGLT2I) cohort [N = 562,907]

All patients meeting all of the following criteria were included:

A. A first prescription of empagliflozin (1545653) between Dec 01 2017 and May 31 2021. [N = 188,722]

B. No prescription of GLP1-RAs (1440051 lixisenatide, 1534763 albiglutide, 1551291 dulaglutide, 1991302 semaglutide, 475968 liraglutide, 60548 exenatide) or other SGLT2I (1373458 canagliflozin, 1488564 dapagliflozin, 1992672 ertugliflozin, 2638675 sotagliflozin) before event A. [N = 117,890]

C. A diagnosis of T2DM recorded within 1 month before and up to the same day as event A. [N = 68,493]

D. Any healthcare visit at least one year before event A. [N = 56,844]

Semaglutide vs Glipizide (SU) comparison

**Semaglutide cohort** [N = 687,980]<br>All patients meeting all of the following criteria were included:<br>A. A first prescription of semaglutide (1991302) between Dec 01 2017 and May 31 2021. [N = 106,323]<br>B. No prescription of SU (10633 tolazamide, 10635 tolbutamide, 173 acetohexamide, 2404 chlorpropamide, 25789 glimepiride, 4815 glyburide, 4816 gliclazide, 4821 glipizide) or other GLP1-RAs (1440051 lixisenatide, 1534763 albiglutide, 1551291 dulaglutide, 475968 liraglutide, 60548 exenatide) before event A. [N = 53,695]<br>C. A diagnosis of T2DM recorded within 1 month before and up to the same day as event A. [N = 25,044]<br>D. Any healthcare visit at least one year before event A. [N = 20,638]
**Glipizide (SU) cohort** [N = 867,428]<br>All patients meeting all of the following criteria were included:<br>A. A first prescription of glipizide (4821) between Dec 01 2017 and May 31 2021. [N = 364,273]<br>B. No prescription of GLP1-RAs (1440051 lixisenatide, 1534763 albiglutide, 1551291 dulaglutide, 1991302 semaglutide, 475968 liraglutide, 60548 exenatide) or other SU (10633 tolazamide, 10635 tolbutamide, 173 acetohexamide, 2404 chlorpropamide, 25789 glimepiride, 4815 glyburide, 4816 gliclazide) before event A. [N = 288,393]<br>C. A diagnosis of T2DM recorded within 1 month before and up to the same day as event A. [N = 156,245]<br>D. Any healthcare visit at least one year before event A. [N = 113,152]

Summary table of the study flow chart

	Exclusion criteria	**Semaglutide vs Sitagliptin**	**Semaglutide vs Empagliflozin**	**Semaglutide vs Glipizide**
**Exposure (semaglutide)**	Participants with a prescription of exposure	687,980	687,980	687,980
	A first prescription of exposure between Dec 01 2017 and May 31 2021 (event A)	111,607	107,157	106,323
	No prescription of comparator or other GLP1-RA before event A	61,449	57,539	53,695
	A diagnosis of T2DM recorded within 1 month before and up to the same day as event A	29,722	28,355	25,044
	Any healthcare visit at least one year before event A	24,626	24,006	20,638
**Comparator (sitagliptin, empagliflozin, glipizide)**	Participants with a prescription of comparator	707,902	562,907	867,428
	A first prescription of comparator between Dec 01 2017 and May 31 2021 (event A)	307,781	188,722	364,273
	No prescription of GLP1-RA or other comparator class drugs before event A	263,013	117,890	288,393

	A diagnosis of T2DM recorded within 1 month before and up to the same day as event A	135,944	68,493	156,245
	Any healthcare visit at least one year before event A	104,541	56,844	113,152

List of covariates (including TriNetX codes)

As mentioned in the main text, the study cohorts were matched for 179 covariates selected based on expert opinion and prior literature[2] (i.e. suspected risk factors for both differences in choice of antidiabetic drug and outcomes), as well as statistical differences between cohorts before matching (i.e. variables whose baseline incidence was greater than 5% in both cohorts and the difference between cohorts was greater than 2% before matching)[6].

	Code	Characteristic
**Demographics**	AI	Age at Index
Sex	F	Female
	M	Male
	UN	Unknown Gender
Ethnicity/race	1002-5	American Indian or Alaska Native
	2028-9	Asian
	2054-5	Black or African American
	2076-8	Native Hawaiian or Other Pacific Islander
	2106-3	White
	2131-1	Other Race
	2135-2	Hispanic or Latino
	2186-5	Not Hispanic or Latino
	UN	Unknown Ethnicity
	UNK	Unknown Race
Marital status	D	Divorced
	M	Married
	S	Never Married
	W	Widowed
**Socioeconomic determinants**	Z55-Z65	Persons with potential health hazards related to socioeconomic and psychosocial circumstances
	Z55	Problems related to education and literacy
	Z56	Problems related to employment and unemployment
	Z59	Problems related to housing and economic circumstances
	Z60	Problems related to social environment
	Z62	Problems related to upbringing
	Z63	Other problems related to primary support group, including family circumstances
	Z64	Problems related to certain psychosocial circumstances
	Z65	Problems related to other psychosocial circumstances
**Lifestyle factors**	Z71.3	Dietary counseling and surveillance
	Z71.4	Alcohol abuse counseling and surveillance
	Z71.5	Drug abuse counseling and surveillance
	Z71.82	Exercise counseling
	Z72	Problems related to lifestyle
	Z72.3	Lack of physical exercise

	Z72.4	Inappropriate diet and eating habits
	Z81	Family history of mental and behavioral disorders
	Z98.84	Bariatric surgery status
**Measures of healthcare utilisation**	Z79	Long term (current) drug therapy
	Z79.4	Long term (current) use of insulin
	Z79.82	Long term (current) use of aspirin
	Z79.84	Long term (current) use of oral hypoglycemic drugs
**Comorbidities**	E00-E89	Endocrine, nutritional and metabolic diseases
	A00-B99	Certain infectious and parasitic diseases
	C00-D49	Neoplasms
	D50-D89	Diseases of the blood and blood-forming organs and certain disorders involving the immune mechanism
	D80-D89	Certain disorders involving the immune mechanism
	E00-E07	Disorders of thyroid gland
	E10	Type 1 diabetes mellitus
	E55	Vitamin D deficiency
	E65-E68	Overweight, obesity and other hyperalimentation
	E70-E88	Metabolic disorders
	E78	Disorders of lipoprotein metabolism and other lipidemias
	E87	Other disorders of fluid, electrolyte and acid-base balance
	E88.81	Metabolic syndrome and other insulin resistance
	F01	Vascular dementia
	F01-F99	Mental, Behavioral and Neurodevelopmental disorders
	F02	Dementia in other diseases classified elsewhere
	F03	Unspecified dementia
	F05	Delirium due to known physiological condition
	F06.8	Other specified mental disorders due to known physiological condition
	F10	Alcohol related disorders
	F10-F19	Mental and behavioral disorders due to psychoactive substance use
	F11	Opioid related disorders
	F12	Cannabis related disorders
	F14	Cocaine related disorders
	F15	Other stimulant related disorders
	F19	Other psychoactive substance related disorders

	F20-F29	Schizophrenia, schizotypal, delusional, and other non-mood psychotic disorders
	F30	Manic episode
	F30-F39	Mood [affective] disorders
	F31	Bipolar disorder
	F32	Depressive episode
	F33	Major depressive disorder, recurrent
	F34	Persistent mood [affective] disorders
	F39	Unspecified mood [affective] disorder
	F40-F48	Anxiety, dissociative, stress-related, somatoform and other nonpsychotic mental disorders
	F50	Eating disorders
	F50-F59	Behavioral syndromes associated with physiological disturbances and physical factors
	F51	Sleep disorders not due to a substance or known physiological condition
	F60-F69	Disorders of adult personality and behavior
	F90	Attention-deficit hyperactivity disorders
	F90-F98	Behavioral and emotional disorders with onset usually occurring in childhood and adolescence
	G00-G99	Diseases of the nervous system
	G04	Encephalitis, myelitis and encephalomyelitis
	G05	Encephalitis, myelitis and encephalomyelitis in diseases classified elsewhere
	G20-G26	Extrapyramidal and movement disorders
	G30	Alzheimer's disease
	G31.0	Frontotemporal dementia
	G31.1	Senile degeneration of brain, not elsewhere classified
	G31.2	Degeneration of nervous system due to alcohol
	G31.83	Neurocognitive disorder with Lewy bodies
	G31.84	Mild cognitive impairment of uncertain or unknown etiology
	G40	Epilepsy and recurrent seizures
	G43	Migraine
	G47	Sleep disorders
	G89.2	Chronic pain, not elsewhere classified
	G93.40	Encephalopathy, unspecified
	H00-H59	Diseases of the eye and adnexa
	H46-H47	Disorders of optic nerve and visual pathways
	H60-H95	Diseases of the ear and mastoid process

	I00-I99	Diseases of the circulatory system
	I10-I1A	Hypertensive diseases
	I12	Hypertensive chronic kidney disease
	I20-I25	Ischemic heart diseases
	I30-I5A	Other forms of heart disease
	I60-I69	Cerebrovascular diseases
	I70-I79	Diseases of arteries, arterioles and capillaries
	J00-J99	Diseases of the respiratory system
	K00-K95	Diseases of the digestive system
	K70-K77	Diseases of liver
	L00-L99	Diseases of the skin and subcutaneous tissue
	L40	Psoriasis
	M00-M99	Diseases of the musculoskeletal system and connective tissue
	M05	Rheumatoid arthritis with rheumatoid factor
	M06	Other rheumatoid arthritis
	M32	Systemic lupus erythematosus (SLE)
	N00-N99	Diseases of the genitourinary system
	N17-N19	Acute kidney failure and chronic kidney disease
	N40-N53	Diseases of male genital organs
	N80-N98	Noninflammatory disorders of female genital tract
	O00-O9A	Pregnancy, childbirth and the puerperium
	R40	Somnolence, stupor and coma
	R41	Other symptoms and signs involving cognitive functions and awareness
	R41.81	Age-related cognitive decline
	R45.851	Suicidal ideations
	R45.88	Nonsuicidal self-harm
	R56	Convulsions, not elsewhere classified
	R63.5	Abnormal weight gain
	S06	Intracranial injury
	T14.91	Suicide attempt
	U07.1	COVID-19
	U09	Post COVID-19 condition
	X71-X83	Intentional self-harm

	Z20.822	Contact with and (suspected) exposure to COVID-19
	Z72.82	Problems related to sleep
	Z86.5	Personal history of mental and behavioral disorders
	Z91.4	Personal history of psychological trauma, not elsewhere classified
	Z91.5	Personal history of self-harm
**Other medications**	1191	aspirin
	4832	glucagon
	5640	ibuprofen
	A08	ANTIOBESITY PREPARATIONS, EXCL. DIET PRODUCTS
	A10A	INSULINS AND ANALOGUES
	A10BA	Biguanides
	A10BB	Sulfonylureas
	A10BG	Thiazolidinediones
	A10BH	Dipeptidyl peptidase 4 (DPP-4) inhibitors
	A10BK	Sodium-glucose co-transporter 2 (SGLT2) inhibitors
	C01B	ANTIARRHYTHMICS, CLASS I AND III
	C02	ANTIHYPERTENSIVES
	C03	DIURETICS
	C07	BETA BLOCKING AGENTS
	C08	CALCIUM CHANNEL BLOCKERS
	C09	AGENTS ACTING ON THE RENIN-ANGIOTENSIN SYSTEM
	C10	LIPID MODIFYING AGENTS
	H02	CORTICOSTEROIDS FOR SYSTEMIC USE
	H03	THYROID THERAPY
	J	ANTIINFECTIVES FOR SYSTEMIC USE
	L01	ANTINEOPLASTIC AGENTS
	L02AA	Estrogens
	L02AB	Progestogens
	L04	IMMUNOSUPPRESSANTS
	M01	ANTIINFLAMMATORY AND ANTIRHEUMATIC PRODUCTS
	N02A	OPIOIDS
	N02B	OTHER ANALGESICS AND ANTIPYRETICS
	N02C	ANTIMIGRAINE PREPARATIONS
	N03	ANTIEPILEPTICS
	N04	ANTI-PARKINSON DRUGS
	N05A	ANTIPSYCHOTICS
	N05AN	Lithium
	N05C	HYPNOTICS AND SEDATIVES
	N06AA	Non-selective monoamine reuptake inhibitors
	N06AB	Selective serotonin reuptake inhibitors
	N06AF	Monoamine oxidase inhibitors, non-selective
	N06AX	Other antidepressants
	N06B	PSYCHOSTIMULANTS, AGENTS USED FOR ADHD AND NOOTROPICS

	N06D	ANTI-DEMENTIA DRUGS
	N07B	DRUGS USED IN ADDICTIVE DISORDERS
	P	ANTIPARASITIC PRODUCTS, INSECTICIDES AND REPELLENTS
	R06	ANTIHISTAMINES FOR SYSTEMIC USE
	V03AB	Antidotes

List of outcomes (including ICD-10-CM codes)

As mentioned in the manuscript and as in our previous studies,[1] for chronic illnesses, only first diagnoses were counted (i.e., patients with the diagnosis before the index event were excluded from the survival analysis). For diagnoses that can recur or relapse, we separately estimated the incidence of first diagnosis and the incidence of any diagnosis, but we focus on first diagnosis in the main manuscript (while presenting the results for any diagnosis in this appendix) given that any diagnosis might include re-coding of previous diagnoses.

Neurological and psychiatric outcomes

Encephalitis: G04 (Encephalitis, myelitis and encephalomyelitis), G05 (Encephalitis, myelitis and encephalomyelitis in diseases classified elsewhere), A86 (Unspecified viral encephalitis), or A85.8 (Other specified viral encephalitis).

Parkinsonism: G20 (Parkinson's disease) or G21 (Secondary parkinsonism).

Cognitive deficit: F01 (Vascular dementia), F02 (Dementia in other diseases classified elsewhere), F03 (Unspecified dementia), F05 (Delirium due to known physiological condition), F06.8 (Other specified mental disorders due to known physiological condition), G30 (Alzheimer's disease), G31.0 (Frontotemporal dementia), G31.83 (Dementia with Lewy bodies), G31.84 (Mild cognitive impairment, so stated), G93.40 (Encephalopathy, unspecified), R40 (Somnolence, stupor and coma), R41 (Other symptoms and signs involving cognitive functions and awareness), R48 (Dyslexia and other symbolic dysfunctions, not elsewhere classified).

Dementia: F01 (Vascular dementia), F02 (Dementia in other diseases classified elsewhere), F03 (Unspecified dementia), G30 (Alzheimer's disease), G31.0 (Frontotemporal dementia), G31.83 (Dementia with Lewy bodies).

Epilepsy/seizure: G40 (Epilepsy and recurrent seizures), R56 (Convulsions, not elsewhere classified).

Migraine: G43 (Migraine)

Insomnia: F51.0 (Insomnia not due to a substance or known physiological condition) or G47.0 (Insomnia).

Nerve/nerve root/plexus disorder: G50-G59 (Nerve, nerve root and plexus disorders).

Myoneural junction (MNJ)/muscle disorder: G70-G73 (Diseases of myoneural junction and muscle) – these disorders are often called neuromuscular, but we use the ICD-10-CM term.

Intracranial haemorrhage (ICH): I60 (non-traumatic subarachnoid haemorrhage), I61 (non-traumatic intracerebral haemorrhage), and I62 (other and unspecified non-traumatic intracranial haemorrhage).

Ischaemic stroke: I63 (cerebral infarction).

Alcohol misuse: F10 (Mental and behavioural disorders due to use of alcohol).

Opioid misuse: F11 (Mental and behavioural disorders due to use of opioids).

Cannabis misuse: F12 (Mental and behavioural disorders due to use of cannabinoids).

Stimulants misuse: F14 (Mental and behavioural disorders due to use of cocaine), F15 (Mental and behavioural disorders due to use of other stimulants, including caffeine), clustered together as per previous studies.[7]

Nicotine misuse: F17 (Mental and behavioural disorders due to use of tobacco)

Psychosis: F20-F29 (Schizophrenia, schizotypal, delusional, and other non-mood psychotic disorders).

Bipolar disorder: F30 (Manic episode), F31 (Bipolar affective disorder). ICD-10-CM codes representing remission (e.g., F31.7 Bipolar affective disorder, currently in remission) were excluded.

Depression: F32, F33, F34. ICD-10-CM codes representing remission (e.g., F32.4 Major depressive disorder, single episode, in partial remission) were excluded.
Anxiety disorder: F40 (Phobic anxiety disorders), F41 (Other anxiety disorders), F43 (Reaction to severe stress, and adjustment disorders), F44 (Dissociative [conversion] disorders), F45 (Somatoform disorders), F48 (Other neurotic disorders).
Obsessive-compulsive disorder (OCD): F42 (Obsessive-compulsive disorder)
Suicidality: R45.851 (Suicidal ideation)

Negative control outcomes (NCO)

Bitten by dog: W54.0 (Bitten by dog).
Ingrown nail: L60.0 (Ingrowing nail).
Ganglion: M67.4 (Ganglion).
Sprain of ankle: S93.4 (Sprain and strain of ankle).
Otitis externa: H60 (Otitis externa).
Sebaceous cyst: L72.3 (Sebaceous cyst).
Seborrheic keratosis: L82 (Seborrheic keratosis).
Trigger finger M65.3 (Trigger finger).
Viral warts: B07 (Viral warts).
Adhesive capsulitis of shoulder: M75.0 (Adhesive capsulitis of shoulder).
Synovitis and tenosynovitis: M65 (Synovitis and tenosynovitis).
Blepharitis: H01.0 (Blepharitis).
Folliculitis: L73 (Other follicular disorders).
Fracture of wrist and hand: S62 (Fracture of wrist and hand).
Tennis elbow: M77.1 (Lateral epicondylitis).
Any NCOs: (all the above) W54.0, L60.0, M67.4, S93.4, H60, L72.3, L82, M65.3, B07, M75.0, M65, H01.0, L73, S62, M77.1.

Other outcomes

All-cause mortality: (deceased). TriNetX records death from EHR which accurately record in-hospital deaths as well as some out-of-hospital deaths. For a subset of patients, linkage with third-party mortality data sources (SSA, Private Obituary Data, and Private Claims Data) is used to increase coverage of out-of-hospital deaths.

Further details of statistical analyses

Statistical analysis plan

Following cohort definition, all analyses follow a similar approach as our group has used in previous cohort studies using the same EHR network.[1,8,9]

Implementation details of propensity score matching

In propensity score matching, the propensity score was calculated using a logistic regression (implemented by the function LogisticRegression of the scikit-learn package in Python 3.7) including each of the covariates mentioned above. To eliminate the influence of ordering of records, the order of the records in the covariate matrix were randomised before matching. The matching itself was performed with NumPy 1.21.5 in Python 3.7.

Censoring

We used the Kaplan-Meier estimator which allows for censoring of individuals who did not have records beyond a certain point. If people had no record beyond a specific timepoint within the first 12 months after exposure, then they were censored at that timepoint (e.g., if their last record was 8 months after exposure, then they were censored at 8 months). If they had a record after the 12-month timepoint and had not developed the outcome within the 12 months post-exposure, then they were censored at 12 months.

Estimating time-varying hazard ratios

Time-varying HRs were assessed using natural cubic splines (in log-time) fitted to the log-cumulative hazard.[10] This was achieved using the generalized survival models of the rstpm2 package (version 1.5.1) in R.[11] As recommended by Royston and Parmar,[10] splines of 1, 2 and 3 degrees were estimated for both the baseline log-cumulative hazard and its cohort dependency and the number of degrees of freedom leading to the lowest Akaike Information Criterion (AIC) was selected. This was achieved on a per-comparison basis so that more complex time dependency (i.e., higher number of degrees of freedom) could be selected for a specific comparison if there was enough evidence in the data to support such complexity.

SUPPLEMENTARY TABLES

Supplementary Table S1. Baseline characteristics before matching

			Semaglutide 24,626		Sitagliptin 104,541			Semaglutide 24,006		Empagliflozin 56,844			Semaglutide 20,638		Glipizide 113,152		
Characteristic			N	%	N	%	SMD	N	%	N	%	SMD	N	%	N	%	SMD
Demographics		Age at index	56.2	12.5	63.6	13.1	0.579	56.9	12.7	60.6	11.9	0.305	55.8	12.5	63.0	13.4	0.552
	Sex	Female	11,990	48.7%	50,853	48.6%	0.001	12,136	50.6%	22,559	39.7%	0.220	10,129	49.1%	52,163	46.1%	0.060
		Male	10,540	42.8%	49,714	47.6%	0.096	9,828	40.9%	30,780	54.1%	0.267	8,514	41.3%	57,278	50.6%	0.189
		Unknown sex	2,096	8.5%	3,974	3.8%	0.197	2,042	8.5%	3,505	6.2%	0.090	1,995	9.7%	3,711	3.3%	0.262
	Ethnicity/race	American Indian or Alaska Native	104	0.4%	486	0.5%	0.006	105	0.4%	284	0.5%	0.009	78	0.4%	576	0.5%	0.020
		Asian	945	3.8%	6,415	6.1%	0.106	965	4.0%	3,139	5.5%	0.071	902	4.4%	5,461	4.8%	0.022
		Black or African American	3,823	15.5%	12,509	12.0%	0.103	3,773	15.7%	9,013	15.9%	0.004	3,366	16.3%	12,769	11.3%	0.146
		Native Hawaiian or Other Pacific Islander	305	1.2%	875	0.8%	0.040	297	1.2%	430	0.8%	0.048	280	1.4%	1,048	0.9%	0.041
		White	14,769	60.0%	62,953	60.2%	0.005	14,293	59.5%	34,279	60.3%	0.016	12,304	59.6%	67,910	60.0%	0.008
		Other Race	855	3.5%	3,711	3.6%	0.004	721	3.0%	1,760	3.1%	0.005	619	3.0%	3,642	3.2%	0.013
		Hispanic or Latino	1,608	6.5%	9,378	9.0%	0.091	1,426	5.9%	5,595	9.8%	0.145	1,277	6.2%	12,543	11.1%	0.175
		Not Hispanic or Latino	15,890	64.5%	70,962	67.9%	0.071	15,512	64.6%	38,032	66.9%	0.048	13,125	63.6%	76,633	67.7%	0.087
		Unknown ethnicity	7,128	28.9%	24,201	23.2%	0.132	7,068	29.4%	13,217	23.3%	0.141	6,236	30.2%	23,976	21.2%	0.208
		Unknown race	3,825	15.5%	17,592	16.8%	0.035	3,852	16.0%	7,939	14.0%	0.058	3,089	15.0%	21,746	19.2%	0.113
	Marital status	Divorced	1,337	5.4%	5,920	5.7%	0.010	1,382	5.8%	2,937	5.2%	0.026	1,177	5.7%	6,096	5.4%	0.014
		Married	7,677	31.2%	31,063	29.7%	0.032	7,654	31.9%	17,285	30.4%	0.032	6,970	33.8%	29,175	25.8%	0.175
		Never Married	2,781	11.3%	11,035	10.6%	0.024	2,600	10.8%	5,740	10.1%	0.024	2,294	11.1%	13,406	11.8%	0.023
		Widowed	1,045	4.2%	8,696	8.3%	0.169	1,134	4.7%	2,846	5.0%	0.013	888	4.3%	8,694	7.7%	0.143

**Socioeconomic determinants**		Persons with potential health hazards related to socioeconomic and psychosocial circumstances	1,091	4.4%	3,512	3.4%	0.055	1,076	4.5%	2,489	4.4%	0.005	1,031	5.0%	1,880	1.7%	0.187
		Problems related to education and literacy	41	0.2%	1,112	1.1%	0.115	48	0.2%	152	0.3%	0.014	39	0.2%	148	0.1%	0.015
		Problems related to employment and unemployment	113	0.5%	321	0.3%	0.025	113	0.5%	227	0.4%	0.011	90	0.4%	421	0.4%	0.010
		Problems related to housing and economic circumstances	278	1.1%	439	0.4%	0.081	259	1.1%	787	1.4%	0.028	208	1.0%	1,238	1.1%	0.008
		Problems related to social environment	57	0.2%	389	0.4%	0.026	63	0.3%	204	0.4%	0.017	52	0.3%	820	0.7%	0.068
		Problems related to upbringing	58	0.2%	161	0.2%	0.018	66	0.3%	106	0.2%	0.018	43	0.2%	433	0.4%	0.032
		Other problems related to primary support group, including family circumstances	316	1.3%	2,345	2.2%	0.073	337	1.4%	701	1.2%	0.015	269	1.3%	1,278	1.1%	0.016
		Problems related to certain psychosocial circumstances	27	0.1%	89	0.1%	0.008	30	0.1%	37	0.1%	0.019	19	0.1%	48	0.0%	0.019
		Problems related to other psychosocial circumstances	267	1.1%	982	0.9%	0.014	298	1.2%	585	1.0%	0.020	262	1.3%	423	0.4%	0.099
**Lifestyle factors**		Dietary counseling and surveillance	1,975	8.0%	4,165	4.0%	0.171	2,037	8.5%	2,894	5.1%	0.135	1,593	7.7%	5,929	5.2%	0.101
		Alcohol abuse counseling and surveillance	58	0.2%	816	0.8%	0.077	76	0.3%	195	0.3%	0.005	58	0.3%	192	0.2%	0.023
		Drug abuse counseling and surveillance	10	0.0%	22	0.0%	0.011	12	0.1%	20	0.0%	0.007	11	0.1%	39	0.0%	0.009
		Exercise counseling	159	0.6%	515	0.5%	0.020	160	0.7%	324	0.6%	0.012	142	0.7%	473	0.4%	0.036
		Problems related to lifestyle	2,112	8.6%	8,200	7.8%	0.027	2,120	8.8%	5,491	9.7%	0.029	1,845	8.9%	5,352	4.7%	0.167

		Lack of physical exercise	33	0.1%	61	0.1%	0.024	30	0.1%	52	0.1%	0.010	21	0.1%	102	0.1%	0.004
		Inappropriate diet and eating habits	31	0.1%	43	0.0%	0.029	31	0.1%	64	0.1%	0.005	23	0.1%	84	0.1%	0.012
		Family history of mental and behavioral disorders	164	0.7%	573	0.5%	0.015	177	0.7%	346	0.6%	0.016	137	0.7%	2,898	2.6%	0.151
**Measures of healthcare utilisation**		Bariatric surgery status	637	2.6%	1,091	1.0%	0.116	641	2.7%	723	1.3%	0.101	603	2.9%	866	0.8%	0.161
		Long term (current) drug therapy	13,893	56.4%	51,983	49.7%	0.134	13,990	58.3%	30,768	54.1%	0.084	12,146	58.9%	55,882	49.4%	0.191
		Long term (current) use of insulin	8,794	35.7%	23,516	22.5%	0.294	8,873	37.0%	16,051	28.2%	0.187	7,045	34.1%	39,377	34.8%	0.014
		Long term (current) use of aspirin	3,511	14.3%	18,756	17.9%	0.100	3,680	15.3%	10,413	18.3%	0.080	3,111	15.1%	17,504	15.5%	0.011
**Comorbidities**		Long term (current) use of oral hypoglycemic drugs	5,008	20.3%	23,484	22.5%	0.052	5,209	21.7%	13,475	23.7%	0.048	4,181	20.3%	31,302	27.7%	0.174
		Endocrine, nutritional and metabolic diseases	24,626	100.0%	104,541	100.0%	NA	24,006	100.0%	56,844	100.0%	NA	20,638	100.0%	113,152	100.0%	NA
		Certain infectious and parasitic diseases	9,985	40.5%	41,444	39.6%	0.018	10,321	43.0%	22,306	39.2%	0.076	8,137	39.4%	45,616	40.3%	0.018
		Neoplasms	8,580	34.8%	37,414	35.8%	0.020	8,953	37.3%	20,478	36.0%	0.026	7,759	37.6%	23,101	20.4%	0.386
		Diseases of the blood and blood-forming organs and certain disorders involving the immune mechanism	8,066	32.8%	38,535	36.9%	0.086	8,510	35.4%	18,780	33.0%	0.051	6,438	31.2%	42,176	37.3%	0.128
		Certain disorders involving the immune mechanism	883	3.6%	2,932	2.8%	0.044	959	4.0%	1,533	2.7%	0.072	730	3.5%	3,236	2.9%	0.038
		Disorders of thyroid gland	5,739	23.3%	29,817	28.5%	0.119	6,173	25.7%	12,296	21.6%	0.096	4,887	23.7%	23,349	20.6%	0.073
		Type 1 diabetes mellitus	2,782	11.3%	9,497	9.1%	0.073	2,744	11.4%	5,375	9.5%	0.065	2,072	10.0%	15,667	13.8%	0.118
		Vitamin D deficiency	6,617	26.9%	24,577	23.5%	0.077	6,825	28.4%	12,988	22.8%	0.128	5,789	28.1%	22,167	19.6%	0.200
		Overweight, obesity and other hyperalimentation	14,624	59.4%	61,631	59.0%	0.009	15,163	63.2%	27,061	47.6%	0.317	12,081	58.5%	65,607	58.0%	0.011
		Metabolic disorders	20,704	84.1%	89,651	85.8%	0.047	20,407	85.0%	49,645	87.3%	0.067	17,269	83.7%	94,049	83.1%	0.015

		Disorders of lipoprotein metabolism and other lipidemias	19,609	79.6%	84,746	81.1%	0.036	19,369	80.7%	47,758	84.0%	0.087	16,360	79.3%	87,380	77.2%	0.050
		Other disorders of fluid, electrolyte and acid-base balance	5,144	20.9%	16,396	15.7%	0.135	5,180	21.6%	11,800	20.8%	0.020	4,368	21.2%	17,374	15.4%	0.151
		Metabolic syndrome and other insulin resistance	1,008	4.1%	4,021	3.8%	0.013	1,145	4.8%	1,604	2.8%	0.102	811	3.9%	4,908	4.3%	0.020
		Vascular dementia	69	0.3%	1,396	1.3%	0.118	77	0.3%	224	0.4%	0.012	67	0.3%	310	0.3%	0.009
		Mental, Behavioral and Neurodevelopmental disorders	12,969	52.7%	51,063	48.8%	0.076	13,000	54.2%	28,437	50.0%	0.083	10,791	52.3%	55,822	49.3%	0.059
		Dementia in other diseases classified elsewhere	89	0.4%	313	0.3%	0.011	91	0.4%	292	0.5%	0.020	82	0.4%	362	0.3%	0.013
		Unspecified dementia	177	0.7%	477	0.5%	0.034	186	0.8%	641	1.1%	0.036	152	0.7%	540	0.5%	0.033
		Delirium due to known physiological condition	112	0.5%	1,066	1.0%	0.066	129	0.5%	330	0.6%	0.006	99	0.5%	701	0.6%	0.019
		Other specified mental disorders due to known physiological condition	132	0.5%	731	0.7%	0.021	145	0.6%	306	0.5%	0.009	99	0.5%	1,012	0.9%	0.050
		Alcohol related disorders	757	3.1%	3,707	3.5%	0.026	765	3.2%	2,389	4.2%	0.054	751	3.6%	2,081	1.8%	0.110
		Mental and behavioral disorders due to psychoactive substance use	4,850	19.7%	20,077	19.2%	0.012	4,838	20.2%	12,265	21.6%	0.035	3,955	19.2%	26,601	23.5%	0.106
		Opioid related disorders	466	1.9%	992	0.9%	0.080	424	1.8%	1,035	1.8%	0.004	362	1.8%	2,386	2.1%	0.026
		Cannabis related disorders	299	1.2%	1,119	1.1%	0.014	317	1.3%	820	1.4%	0.010	268	1.3%	1,501	1.3%	0.002
		Cocaine related disorders	206	0.8%	1,465	1.4%	0.054	211	0.9%	575	1.0%	0.014	159	0.8%	1,378	1.2%	0.045
		Other stimulant related disorders	126	0.5%	544	0.5%	0.001	139	0.6%	333	0.6%	0.001	116	0.6%	621	0.5%	0.002
		Other psychoactive substance related disorders	366	1.5%	1,616	1.5%	0.005	392	1.6%	908	1.6%	0.003	312	1.5%	2,021	1.8%	0.022

		Schizophrenia, schizotypal, delusional, and other non-mood psychotic disorders	454	1.8%	2,813	2.7%	0.057	494	2.1%	1,223	2.2%	0.007	367	1.8%	3,304	2.9%	0.075
		Manic episode	48	0.2%	187	0.2%	0.004	58	0.2%	108	0.2%	0.011	41	0.2%	1,012	0.9%	0.094
		Mood [affective] disorders	7,527	30.6%	26,591	25.4%	0.114	7,650	31.9%	14,636	25.7%	0.135	6,238	30.2%	27,682	24.5%	0.130
		Bipolar disorder	854	3.5%	1,925	1.8%	0.101	820	3.4%	1,543	2.7%	0.041	592	2.9%	5,246	4.6%	0.093
		Depressive episode	6,515	26.5%	23,022	22.0%	0.104	6,675	27.8%	12,618	22.2%	0.130	5,419	26.3%	23,730	21.0%	0.125
		Major depressive disorder, recurrent	1,966	8.0%	5,780	5.5%	0.098	2,034	8.5%	3,605	6.3%	0.081	1,567	7.6%	10,937	9.7%	0.074
		Persistent mood [affective] disorders	923	3.7%	3,020	2.9%	0.048	1,037	4.3%	1,770	3.1%	0.064	779	3.8%	2,843	2.5%	0.072
		Unspecified mood [affective] disorder	637	2.6%	2,006	1.9%	0.045	696	2.9%	1,181	2.1%	0.053	544	2.6%	2,541	2.2%	0.025
		Anxiety, dissociative, stress-related, somatoform and other nonpsychotic mental disorders	7,668	31.1%	26,393	25.2%	0.131	7,870	32.8%	15,509	27.3%	0.120	6,483	31.4%	27,781	24.6%	0.153
		Eating disorders	241	1.0%	805	0.8%	0.022	298	1.2%	295	0.5%	0.077	182	0.9%	1,302	1.2%	0.027
		Behavioral syndromes associated with physiological disturbances and physical factors	1,748	7.1%	5,235	5.0%	0.088	1,778	7.4%	3,326	5.9%	0.063	1,470	7.1%	5,249	4.6%	0.106
		Sleep disorders not due to a substance or known physiological condition	1,181	4.8%	2,098	2.0%	0.154	1,101	4.6%	2,166	3.8%	0.039	959	4.6%	2,752	2.4%	0.120
		Disorders of adult personality and behavior	347	1.4%	1,213	1.2%	0.022	369	1.5%	696	1.2%	0.027	273	1.3%	2,361	2.1%	0.059
		Attention-deficit hyperactivity disorders	424	1.7%	1,666	1.6%	0.010	460	1.9%	647	1.1%	0.063	330	1.6%	2,030	1.8%	0.015
		Behavioral and emotional disorders with onset usually	599	2.4%	2,815	2.7%	0.016	651	2.7%	976	1.7%	0.068	522	2.5%	2,135	1.9%	0.044

		occurring in childhood and adolescence															
		Diseases of the nervous system	15,872	64.5%	62,400	59.7%	0.098	15,940	66.4%	35,203	61.9%	0.093	13,125	63.6%	71,645	63.3%	0.006
		Encephalitis, myelitis and encephalomyelitis	44	0.2%	169	0.2%	0.004	47	0.2%	71	0.1%	0.018	35	0.2%	222	0.2%	0.006
		Encephalitis, myelitis and encephalomyelitis in diseases classified elsewhere	10	0.0%	10	0.0%	0.020	10	0.0%	10	0.0%	0.014	10	0.0%	10	0.0%	0.023
		Extrapyramidal and movement disorders	1,366	5.5%	5,807	5.6%	0.000	1,447	6.0%	3,014	5.3%	0.031	1,370	6.6%	2,593	2.3%	0.212
		Alzheimer's disease	59	0.2%	150	0.1%	0.022	59	0.2%	228	0.4%	0.027	49	0.2%	155	0.1%	0.023
		Frontotemporal dementia	10	0.0%	59	0.1%	0.007	10	0.0%	19	0.0%	0.004	10	0.0%	54	0.0%	0.000
		Senile degeneration of brain, not elsewhere classified	10	0.0%	10	0.0%	0.020	10	0.0%	14	0.0%	0.009	10	0.0%	15	0.0%	0.020
		Degeneration of nervous system due to alcohol	0	0.0%	15	0.0%	0.017	0	0.0%	10	0.0%	0.019	0	0.0%	0	0.0%	NA
		Neurocognitive disorder with Lewy bodies	10	0.0%	68	0.1%	0.011	10	0.0%	21	0.0%	0.002	10	0.0%	75	0.1%	0.007
		Mild cognitive impairment of uncertain or unknown etiology	215	0.9%	897	0.9%	0.002	228	1.0%	621	1.1%	0.014	180	0.9%	1,744	1.5%	0.061
		Epilepsy and recurrent seizures	449	1.8%	2,311	2.2%	0.028	447	1.9%	1,029	1.8%	0.004	391	1.9%	982	0.9%	0.088
		Migraine	2,118	8.6%	5,499	5.3%	0.132	2,139	8.9%	3,416	6.0%	0.111	1,726	8.4%	9,226	8.2%	0.008
		Sleep disorders	9,713	39.4%	30,111	28.8%	0.226	9,686	40.3%	18,931	33.3%	0.146	8,120	39.3%	31,123	27.5%	0.253
		Chronic pain, not elsewhere classified	6,020	24.4%	21,362	20.4%	0.096	6,218	25.9%	13,060	23.0%	0.068	4,777	23.1%	31,056	27.4%	0.099
		Encephalopathy, unspecified	326	1.3%	1,314	1.3%	0.006	338	1.4%	825	1.5%	0.004	271	1.3%	1,692	1.5%	0.015
		Diseases of the eye and adnexa	8,118	33.0%	37,703	36.1%	0.065	8,595	35.8%	20,787	36.6%	0.016	6,689	32.4%	40,605	35.9%	0.073

		Disorders of optic nerve and visual pathways	365	1.5%	1,523	1.5%	0.002	384	1.6%	1,074	1.9%	0.022	295	1.4%	1,728	1.5%	0.008
		Diseases of the ear and mastoid process	6,075	24.7%	24,606	23.5%	0.026	6,254	26.1%	14,059	24.7%	0.030	5,323	25.8%	22,928	20.3%	0.132
		Diseases of the circulatory system	20,831	84.6%	93,248	89.2%	0.137	20,577	85.7%	50,840	89.4%	0.113	17,327	84.0%	100,375	88.7%	0.139
		Hypertensive diseases	19,687	79.9%	88,539	84.7%	0.125	19,477	81.1%	48,232	84.9%	0.099	16,319	79.1%	95,347	84.3%	0.134
		Hypertensive chronic kidney disease	2,104	8.5%	12,587	12.0%	0.115	2,479	10.3%	4,560	8.0%	0.080	1,757	8.5%	13,277	11.7%	0.107
		Ischemic heart diseases	5,822	23.6%	24,849	23.8%	0.003	5,773	24.0%	18,842	33.1%	0.202	4,751	23.0%	26,538	23.5%	0.010
		Other forms of heart disease	7,839	31.8%	38,868	37.2%	0.113	8,061	33.6%	21,416	37.7%	0.086	6,395	31.0%	41,840	37.0%	0.127
		Cerebrovascular diseases	2,503	10.2%	15,114	14.5%	0.131	2,606	10.9%	7,476	13.2%	0.071	2,102	10.2%	9,412	8.3%	0.064
		Diseases of arteries, arterioles and capillaries	3,488	14.2%	17,001	16.3%	0.058	3,687	15.4%	9,887	17.4%	0.055	2,889	14.0%	19,160	16.9%	0.081
		Diseases of the respiratory system	15,505	63.0%	42,049	40.2%	0.467	14,860	61.9%	33,657	59.2%	0.055	12,884	62.4%	46,196	40.8%	0.443
		Diseases of the digestive system	15,766	64.0%	67,087	64.2%	0.003	15,971	66.5%	37,189	65.4%	0.023	13,219	64.1%	66,885	59.1%	0.102
		Diseases of liver	4,394	17.8%	14,742	14.1%	0.102	4,428	18.4%	9,464	16.6%	0.047	3,475	16.8%	22,280	19.7%	0.074
		Diseases of the skin and subcutaneous tissue	11,816	48.0%	47,927	45.8%	0.043	12,280	51.2%	26,620	46.8%	0.087	9,672	46.9%	51,996	46.0%	0.018
		Psoriasis	755	3.1%	2,442	2.3%	0.045	754	3.1%	1,504	2.6%	0.030	610	3.0%	2,555	2.3%	0.044
		Diseases of the musculoskeletal system and connective tissue	18,260	74.1%	78,655	75.2%	0.025	18,374	76.5%	42,856	75.4%	0.027	15,158	73.4%	84,649	74.8%	0.031
		Rheumatoid arthritis with rheumatoid factor	129	0.5%	679	0.7%	0.016	146	0.6%	328	0.6%	0.004	117	0.6%	719	0.6%	0.009
		Other rheumatoid arthritis	613	2.5%	2,656	2.5%	0.003	640	2.7%	1,295	2.3%	0.025	556	2.7%	1,690	1.5%	0.084
		Systemic lupus erythematosus (SLE)	149	0.6%	591	0.6%	0.005	167	0.7%	282	0.5%	0.026	125	0.6%	580	0.5%	0.012

		Diseases of the genitourinary system	15,213	61.8%	67,376	64.4%	0.055	15,657	65.2%	34,654	61.0%	0.088	12,661	61.3%	72,537	64.1%	0.057
		Acute kidney failure and chronic kidney disease	5,071	20.6%	28,459	27.2%	0.156	5,662	23.6%	12,021	21.1%	0.059	4,275	20.7%	26,419	23.3%	0.064
		Diseases of male genital organs	4,316	17.5%	20,466	19.6%	0.053	4,335	18.1%	12,811	22.5%	0.112	3,726	18.1%	15,449	13.7%	0.121
		Noninflammatory disorders of female genital tract	4,871	19.8%	24,054	23.0%	0.079	5,437	22.6%	8,579	15.1%	0.194	3,942	19.1%	24,571	21.7%	0.065
		Pregnancy, childbirth and the puerperium	976	4.0%	3,876	3.7%	0.013	1,187	4.9%	1,359	2.4%	0.136	814	3.9%	4,395	3.9%	0.003
		Somnolence, stupor and coma	1,212	4.9%	1,995	1.9%	0.166	1,095	4.6%	2,163	3.8%	0.038	900	4.4%	3,335	2.9%	0.075
		Other symptoms and signs involving cognitive functions and awareness	1,882	7.6%	11,177	10.7%	0.106	2,003	8.3%	4,812	8.5%	0.004	1,734	8.4%	5,271	4.7%	0.152
		Age-related cognitive decline	16	0.1%	127	0.1%	0.019	21	0.1%	55	0.1%	0.003	15	0.1%	153	0.1%	0.019
		Suicidal ideations	353	1.4%	1,358	1.3%	0.012	381	1.6%	731	1.3%	0.025	289	1.4%	1,688	1.5%	0.008
		Nonsuicidal self-harm	0	0.0%	10	0.0%	0.014	0	0.0%	0	0.0%	NA	10	0.0%	10	0.0%	0.023
		Convulsions, not elsewhere classified	647	2.6%	1,587	1.5%	0.078	632	2.6%	1,358	2.4%	0.016	555	2.7%	1,653	1.5%	0.086
		Abnormal weight gain	1,512	6.1%	2,693	2.6%	0.175	1,580	6.6%	1,853	3.3%	0.154	1,108	5.4%	5,712	5.0%	0.014
		Intracranial injury	531	2.2%	2,467	2.4%	0.014	528	2.2%	1,238	2.2%	0.001	397	1.9%	2,638	2.3%	0.028
		Suicide attempt	36	0.1%	119	0.1%	0.009	41	0.2%	71	0.1%	0.012	35	0.2%	128	0.1%	0.015
		COVID-19	667	2.7%	1,445	1.4%	0.094	613	2.6%	1,235	2.2%	0.025	515	2.5%	2,903	2.6%	0.004
		Post COVID-19 condition	0	0.0%	26	0.0%	0.022	10	0.0%	10	0.0%	0.014	0	0.0%	29	0.0%	0.023
		Intentional self-harm	40	0.2%	116	0.1%	0.014	47	0.2%	74	0.1%	0.016	39	0.2%	140	0.1%	0.017
		Contact with and (suspected) exposure to COVID-19	793	3.2%	2,936	2.8%	0.024	850	3.5%	1,798	3.2%	0.021	694	3.4%	3,135	2.8%	0.034
		Problems related to sleep	162	0.7%	2,964	2.8%	0.167	191	0.8%	296	0.5%	0.034	146	0.7%	640	0.6%	0.018

		Personal history of mental and behavioral disorders	232	0.9%	1,152	1.1%	0.016	244	1.0%	487	0.9%	0.017	201	1.0%	919	0.8%	0.017
		Personal history of psychological trauma, not elsewhere classified	43	0.2%	204	0.2%	0.005	54	0.2%	91	0.2%	0.015	46	0.2%	219	0.2%	0.006
		Personal history of self-harm	80	0.3%	250	0.2%	0.016	97	0.4%	164	0.3%	0.020	70	0.3%	1,253	1.1%	0.091
**Other medications**		aspirin	9,159	37.2%	47,150	45.1%	0.161	9,278	38.6%	25,804	45.4%	0.137	7,214	35.0%	49,559	43.8%	0.182
		glucagon	4,454	18.1%	16,623	15.9%	0.058	4,481	18.7%	9,854	17.3%	0.035	3,613	17.5%	19,623	17.3%	0.004
		ibuprofen	6,613	26.9%	24,380	23.3%	0.082	6,667	27.8%	13,989	24.6%	0.072	5,029	24.4%	22,540	19.9%	0.107
		ANTIOBESITY PREPARATIONS, EXCL. DIET PRODUCTS	745	3.0%	2,264	2.2%	0.054	860	3.6%	792	1.4%	0.141	609	3.0%	2,528	2.2%	0.045
		INSULINS AND ANALOGUES	13,122	53.3%	49,926	47.8%	0.111	13,141	54.7%	27,228	47.9%	0.137	10,725	52.0%	51,800	45.8%	0.124
		Biguanides	18,262	74.2%	79,123	75.7%	0.035	17,915	74.6%	45,010	79.2%	0.108	14,695	71.2%	82,666	73.1%	0.041
		Thiazolidinediones	1,559	6.3%	9,284	8.9%	0.096	1,716	7.1%	4,753	8.4%	0.045	1,217	5.9%	5,319	4.7%	0.053
		Dipeptidyl peptidase 4 (DPP-4) inhibitors	-	-	-	-	-	5,310	22.1%	16,609	29.2%	0.163	3,804	18.4%	24,280	21.5%	0.076
		Sodium-glucose co-transporter 2 (SGLT2) inhibitors	5,226	21.2%	11,480	11.0%	0.281	-	-	-	-	-	5,186	25.1%	27,760	24.5%	0.014
		Sulfonylureas	7,130	29.0%	45,436	43.5%	0.305	7,883	32.8%	22,905	40.3%	0.155	-	-	-	-	-
		ANTIARRHYTHMICS, CLASS I AND III	11,843	48.1%	46,229	44.2%	0.078	12,053	50.2%	26,720	47.0%	0.064	9,786	47.4%	49,053	43.4%	0.082
		ANTIHYPERTENSIVES	4,174	17.0%	20,439	19.6%	0.067	4,356	18.1%	9,782	17.2%	0.025	3,395	16.5%	21,446	19.0%	0.066
		DIURETICS	11,469	46.6%	52,049	49.8%	0.064	11,765	49.0%	27,724	48.8%	0.005	9,399	45.5%	48,739	43.1%	0.050
		BETA BLOCKING AGENTS	10,584	43.0%	50,884	48.7%	0.114	10,770	44.9%	27,911	49.1%	0.085	8,583	41.6%	54,309	48.0%	0.129
		CALCIUM CHANNEL BLOCKERS	7,796	31.7%	38,565	36.9%	0.110	8,082	33.7%	20,340	35.8%	0.044	6,276	30.4%	41,358	36.6%	0.130

		AGENTS ACTING ON THE RENIN-ANGIOTENSIN SYSTEM	15,781	64.1%	74,137	70.9%	0.146	15,727	65.5%	41,479	73.0%	0.162	12,660	61.3%	78,678	69.5%	0.173
		LIPID MODIFYING AGENTS	17,236	70.0%	81,485	77.9%	0.182	17,108	71.3%	45,354	79.8%	0.199	14,075	68.2%	84,669	74.8%	0.147
		CORTICOSTEROIDS FOR SYSTEMIC USE	14,368	58.3%	58,178	55.7%	0.054	14,622	60.9%	32,193	56.6%	0.087	11,912	57.7%	61,146	54.0%	0.074
		THYROID THERAPY	3,642	14.8%	16,534	15.8%	0.029	3,744	15.6%	7,646	13.5%	0.061	3,102	15.0%	14,828	13.1%	0.055
		ANTIINFECTIVES FOR SYSTEMIC USE	19,362	78.6%	82,774	79.2%	0.014	19,377	80.7%	44,654	78.6%	0.054	16,060	77.8%	88,946	78.6%	0.019
		ANTINEOPLASTIC AGENTS	2,686	10.9%	10,822	10.4%	0.018	2,834	11.8%	5,855	10.3%	0.048	2,209	10.7%	10,557	9.3%	0.046
		Estrogens	773	3.1%	1,334	1.3%	0.127	787	3.3%	754	1.3%	0.130	642	3.1%	1,419	1.3%	0.127
		Progestogens	871	3.5%	1,105	1.1%	0.166	796	3.3%	1,190	2.1%	0.075	745	3.6%	1,077	1.0%	0.179
		IMMUNOSUPPRESSANTS	1,331	5.4%	5,196	5.0%	0.020	1,423	5.9%	2,478	4.4%	0.071	1,014	4.9%	8,110	7.2%	0.095
		ANTIINFLAMMATORY AND ANTIRHEUMATIC PRODUCTS	13,117	53.3%	51,941	49.7%	0.072	13,231	55.1%	29,274	51.5%	0.073	10,588	51.3%	56,020	49.5%	0.036
		OPIOIDS	13,263	53.9%	57,480	55.0%	0.023	13,516	56.3%	31,363	55.2%	0.023	10,851	52.6%	61,565	54.4%	0.037
		OTHER ANALGESICS AND ANTIPYRETICS	16,817	68.3%	76,636	73.3%	0.111	16,849	70.2%	40,717	71.6%	0.032	13,658	66.2%	83,167	73.5%	0.160
		ANTIMIGRAINE PREPARATIONS	1,986	8.1%	8,125	7.8%	0.011	2,090	8.7%	3,815	6.7%	0.075	1,631	7.9%	8,447	7.5%	0.016
		ANTIEPILEPTICS	8,223	33.4%	34,503	33.0%	0.008	8,401	35.0%	18,544	32.6%	0.050	6,668	32.3%	36,523	32.3%	0.001
		ANTI-PARKINSON DRUGS	1,164	4.7%	5,327	5.1%	0.017	1,189	5.0%	2,407	4.2%	0.034	911	4.4%	5,086	4.5%	0.004
		ANTIPSYCHOTICS	3,618	14.7%	14,640	14.0%	0.020	3,785	15.8%	7,691	13.5%	0.063	2,927	14.2%	15,955	14.1%	0.002
		Lithium	26	0.1%	128	0.1%	0.005	29	0.1%	75	0.1%	0.003	19	0.1%	153	0.1%	0.013
		HYPNOTICS AND SEDATIVES	10,213	41.5%	42,707	40.9%	0.013	10,475	43.6%	24,109	42.4%	0.025	8,392	40.7%	43,085	38.1%	0.053

		Non-selective monoamine reuptake inhibitors	1,867	7.6%	7,125	6.8%	0.030	1,955	8.1%	4,083	7.2%	0.036	1,425	6.9%	7,583	6.7%	0.008
		Selective serotonin reuptake inhibitors	5,782	23.5%	22,094	21.1%	0.056	5,893	24.5%	11,755	20.7%	0.093	4,515	21.9%	8,193	7.2%	0.424
		Monoamine oxidase inhibitors, non-selective	10	0.0%	11	0.0%	0.019	10	0.0%	10	0.0%	0.014	10	0.0%	17	0.0%	0.019
		Other antidepressants	6,114	24.8%	22,277	21.3%	0.084	6,227	25.9%	12,299	21.6%	0.101	4,684	22.7%	22,608	20.0%	0.066
		PSYCHOSTIMULANTS, AGENTS USED FOR ADHD AND NOOTROPICS	1,579	6.4%	4,855	4.6%	0.077	1,634	6.8%	3,017	5.3%	0.063	1,154	5.6%	4,493	4.0%	0.076
		ANTI-DEMENTIA DRUGS	163	0.7%	2,447	2.3%	0.138	174	0.7%	616	1.1%	0.038	124	0.6%	2,197	1.9%	0.120
		DRUGS USED IN ADDICTIVE DISORDERS	2,105	8.5%	7,638	7.3%	0.046	2,148	8.9%	4,771	8.4%	0.020	1,663	8.1%	9,122	8.1%	0.000
		ANTIPARASITIC PRODUCTS, INSECTICIDES AND REPELLENTS	3,813	15.5%	14,315	13.7%	0.051	4,050	16.9%	7,555	13.3%	0.100	3,183	15.4%	15,262	13.5%	0.055
		ANTIHISTAMINES FOR SYSTEMIC USE	11,444	46.5%	47,026	45.0%	0.030	11,766	49.0%	25,361	44.6%	0.088	9,204	44.6%	56,407	49.9%	0.105
		Antidotes	4,562	18.5%	17,420	16.7%	0.049	4,649	19.4%	9,926	17.5%	0.049	3,813	18.5%	17,544	15.5%	0.079

Supplementary Table S2. Baseline characteristics after matching

			Semaglutide 23,386		Sitagliptin 23,386			Semaglutide 22,584		Empagliflozin 22,584			Semaglutide 19,206		Glipizide 19,205		
**Characteristic**			N	%	N	%	SMD	N	%	N	%	SMD	N	%	N	%	SMD
**Demographics**		Age at index	56.7	12.2	56.6	13.3	0.010	57.6	12.3	57.6	12.4	0.001	56.4	12.4	56.2	13.6	0.018
	Sex	Female	11,411	48.8%	11,317	48.4%	0.008	11,067	49.0%	11,012	48.8%	0.005	9,440	49.2%	9,497	49.4%	0.006
		Male	10,183	43.5%	10,269	43.9%	0.007	9,638	42.7%	9,690	42.9%	0.005	8,150	42.4%	8,104	42.2%	0.005
		Unknown sex	1,792	7.7%	1,800	7.7%	0.001	1,879	8.3%	1,882	8.3%	0.000	1,616	8.4%	1,605	8.4%	0.002
	Ethnicity/race	American Indian or Alaska Native	99	0.4%	98	0.4%	0.001	101	0.4%	100	0.4%	0.001	74	0.4%	77	0.4%	0.002
		Asian	923	3.9%	902	3.9%	0.005	944	4.2%	909	4.0%	0.008	862	4.5%	884	4.6%	0.005
		Black or African American	3,461	14.8%	3,503	15.0%	0.005	3,546	15.7%	3,544	15.7%	0.000	2,938	15.3%	2,963	15.4%	0.004
		Native Hawaiian or Other Pacific Islander	282	1.2%	276	1.2%	0.002	248	1.1%	260	1.2%	0.005	261	1.4%	271	1.4%	0.004
		White	14,137	60.5%	14,117	60.4%	0.002	13,480	59.7%	13,533	59.9%	0.005	11,526	60.0%	11,486	59.8%	0.004
		Other Race	800	3.4%	832	3.6%	0.007	680	3.0%	669	3.0%	0.003	579	3.0%	584	3.0%	0.002
		Hispanic or Latino	1,558	6.7%	1,617	6.9%	0.010	1,397	6.2%	1,322	5.9%	0.014	1,239	6.5%	1,278	6.7%	0.008
		Not Hispanic or Latino	15,219	65.1%	15,196	65.0%	0.002	14,683	65.0%	14,788	65.5%	0.010	12,355	64.3%	12,297	64.0%	0.006
		Unknown ethnicity	6,609	28.3%	6,573	28.1%	0.003	6,504	28.8%	6,474	28.7%	0.003	5,612	29.2%	5,631	29.3%	0.002
		Unknown race	3,684	15.8%	3,658	15.6%	0.003	3,585	15.9%	3,569	15.8%	0.002	2,966	15.4%	2,941	15.3%	0.004
	Marital status	Divorced	1,289	5.5%	1,304	5.6%	0.003	1,306	5.8%	1,299	5.8%	0.001	1,102	5.7%	1,121	5.8%	0.004
		Married	7,317	31.3%	7,414	31.7%	0.009	7,131	31.6%	7,127	31.6%	0.000	6,431	33.5%	6,354	33.1%	0.009
		Never Married	2,650	11.3%	2,589	11.1%	0.008	2,377	10.5%	2,330	10.3%	0.007	2,163	11.3%	2,208	11.5%	0.007
		Widowed	1,031	4.4%	965	4.1%	0.014	1,087	4.8%	1,146	5.1%	0.012	870	4.5%	844	4.4%	0.007
**Socioeconomic determinants**		Persons with potential health hazards related to socioeconomic and psychosocial circumstances	1,007	4.3%	1,008	4.3%	0.000	994	4.4%	1,023	4.5%	0.006	813	4.2%	748	3.9%	0.017
		Problems related to education and literacy	41	0.2%	29	0.1%	0.013	46	0.2%	47	0.2%	0.001	39	0.2%	32	0.2%	0.008
		Problems related to employment and unemployment	104	0.4%	110	0.5%	0.004	101	0.4%	117	0.5%	0.010	82	0.4%	91	0.5%	0.007

		Problems related to housing and economic circumstances	221	0.9%	212	0.9%	0.004	247	1.1%	259	1.1%	0.005	194	1.0%	193	1.0%	0.001
		Problems related to social environment	56	0.2%	56	0.2%	0.000	61	0.3%	60	0.3%	0.001	50	0.3%	54	0.3%	0.004
		Problems related to upbringing	52	0.2%	57	0.2%	0.004	56	0.2%	58	0.3%	0.002	43	0.2%	45	0.2%	0.002
		Other problems related to primary support group, including family circumstances	304	1.3%	297	1.3%	0.003	313	1.4%	315	1.4%	0.001	247	1.3%	270	1.4%	0.010
		Problems related to certain psychosocial circumstances	23	0.1%	24	0.1%	0.001	21	0.1%	19	0.1%	0.003	16	0.1%	17	0.1%	0.002
		Problems related to other psychosocial circumstances	249	1.1%	249	1.1%	0.000	271	1.2%	285	1.3%	0.006	199	1.0%	191	1.0%	0.004
**Lifestyle factors**		Dietary counseling and surveillance	1,672	7.2%	1,558	6.7%	0.019	1,690	7.5%	1,676	7.4%	0.002	1,406	7.3%	1,433	7.5%	0.005
		Alcohol abuse counseling and surveillance	56	0.2%	51	0.2%	0.004	73	0.3%	86	0.4%	0.010	53	0.3%	50	0.3%	0.003
		Drug abuse counseling and surveillance	10	0.0%	10	0.0%	0.000	11	0.0%	13	0.1%	0.004	10	0.1%	10	0.1%	0.000
		Exercise counseling	149	0.6%	151	0.6%	0.001	147	0.7%	132	0.6%	0.008	127	0.7%	136	0.7%	0.006
		Problems related to lifestyle	1,990	8.5%	1,981	8.5%	0.001	2,007	8.9%	2,052	9.1%	0.007	1,619	8.4%	1,590	8.3%	0.005
		Lack of physical exercise	28	0.1%	25	0.1%	0.004	28	0.1%	24	0.1%	0.005	21	0.1%	19	0.1%	0.003
		Inappropriate diet and eating habits	25	0.1%	24	0.1%	0.001	27	0.1%	24	0.1%	0.004	21	0.1%	20	0.1%	0.002
		Family history of mental and behavioral disorders	153	0.7%	155	0.7%	0.001	159	0.7%	161	0.7%	0.001	135	0.7%	140	0.7%	0.003
**Measures of healthcare**		Bariatric surgery status	547	2.3%	538	2.3%	0.003	513	2.3%	504	2.2%	0.003	454	2.4%	429	2.2%	0.009
		Long term (current) drug therapy	12,925	55.3%	12,897	55.1%	0.002	12,997	57.6%	13,068	57.9%	0.006	11,019	57.4%	10,959	57.1%	0.006
		Long term (current) use of insulin	7,976	34.1%	7,979	34.1%	0.000	8,120	36.0%	8,151	36.1%	0.003	6,548	34.1%	6,454	33.6%	0.010
		Long term (current) use of aspirin	3,384	14.5%	3,375	14.4%	0.001	3,531	15.6%	3,582	15.9%	0.006	2,894	15.1%	2,884	15.0%	0.001
**Comorbidities**		Long term (current) use of oral hypoglycemic drugs	4,775	20.4%	4,772	20.4%	0.000	4,937	21.9%	4,969	22.0%	0.003	3,956	20.6%	3,820	19.9%	0.018
		Endocrine, nutritional and metabolic diseases	23,386	100.0%	23,386	100.0%	NA	22,584	100.0%	22,584	100.0%	NA	19,206	100.0%	19,206	100.0%	NA
		Certain infectious and parasitic diseases	9,439	40.4%	9,409	40.2%	0.003	9,549	42.3%	9,586	42.4%	0.003	7,573	39.4%	7,607	39.6%	0.004

		Neoplasms	8,167	34.9%	8,118	34.7%	0.004	8,402	37.2%	8,489	37.6%	0.008	6,816	35.5%	6,810	35.5%	0.001
		Diseases of the blood and blood-forming organs and certain disorders involving the immune mechanism	7,703	32.9%	7,691	32.9%	0.001	7,865	34.8%	7,936	35.1%	0.007	6,075	31.6%	6,056	31.5%	0.002
		Certain disorders involving the immune mechanism	820	3.5%	781	3.3%	0.009	836	3.7%	832	3.7%	0.001	662	3.4%	691	3.6%	0.008
		Disorders of thyroid gland	5,541	23.7%	5,501	23.5%	0.004	5,697	25.2%	5,610	24.8%	0.009	4,480	23.3%	4,518	23.5%	0.005
		Type 1 diabetes mellitus	2,520	10.8%	2,515	10.8%	0.001	2,520	11.2%	2,616	11.6%	0.013	1,967	10.2%	1,936	10.1%	0.005
		Vitamin D deficiency	6,198	26.5%	6,144	26.3%	0.005	6,208	27.5%	6,271	27.8%	0.006	5,163	26.9%	5,241	27.3%	0.009
		Overweight, obesity and other hyperalimentation	13,865	59.3%	13,920	59.5%	0.005	13,850	61.3%	13,895	61.5%	0.004	11,245	58.5%	11,287	58.8%	0.004
		Metabolic disorders	19,668	84.1%	19,662	84.1%	0.001	19,279	85.4%	19,385	85.8%	0.013	16,032	83.5%	16,035	83.5%	0.000
		Disorders of lipoprotein metabolism and other lipidemias	18,650	79.7%	18,637	79.7%	0.001	18,372	81.4%	18,492	81.9%	0.014	15,185	79.1%	15,220	79.2%	0.004
		Other disorders of fluid, electrolyte and acid-base balance	4,748	20.3%	4,638	19.8%	0.012	4,839	21.4%	4,883	21.6%	0.005	3,953	20.6%	4,009	20.9%	0.007
		Metabolic syndrome and other insulin resistance	945	4.0%	904	3.9%	0.009	961	4.3%	944	4.2%	0.004	752	3.9%	781	4.1%	0.008
		Vascular dementia	69	0.3%	55	0.2%	0.012	76	0.3%	71	0.3%	0.004	62	0.3%	77	0.4%	0.013
		Mental, Behavioral and Neurodevelopmental disorders	12,206	52.2%	12,188	52.1%	0.002	12,027	53.3%	12,092	53.5%	0.006	9,935	51.7%	9,981	52.0%	0.005
		Dementia in other diseases classified elsewhere	80	0.3%	91	0.4%	0.008	90	0.4%	91	0.4%	0.001	72	0.4%	69	0.4%	0.003
		Unspecified dementia	164	0.7%	164	0.7%	0.000	182	0.8%	178	0.8%	0.002	136	0.7%	135	0.7%	0.001
		Delirium due to known physiological condition	111	0.5%	112	0.5%	0.001	121	0.5%	124	0.5%	0.002	87	0.5%	89	0.5%	0.002
		Other specified mental disorders due to known physiological condition	120	0.5%	122	0.5%	0.001	131	0.6%	135	0.6%	0.002	98	0.5%	100	0.5%	0.001
		Alcohol related disorders	731	3.1%	733	3.1%	0.000	747	3.3%	769	3.4%	0.005	623	3.2%	587	3.1%	0.011
		Mental and behavioral disorders due to psychoactive substance use	4,623	19.8%	4,629	19.8%	0.001	4,587	20.3%	4,688	20.8%	0.011	3,706	19.3%	3,660	19.1%	0.006

		Opioid related disorders	416	1.8%	450	1.9%	0.011	401	1.8%	417	1.8%	0.005	338	1.8%	347	1.8%	0.004
		Cannabis related disorders	273	1.2%	304	1.3%	0.012	298	1.3%	314	1.4%	0.006	241	1.3%	246	1.3%	0.002
		Cocaine related disorders	195	0.8%	195	0.8%	0.000	200	0.9%	197	0.9%	0.001	152	0.8%	148	0.8%	0.002
		Other stimulant related disorders	122	0.5%	129	0.6%	0.004	135	0.6%	120	0.5%	0.009	107	0.6%	107	0.6%	0.000
		Other psychoactive substance related disorders	347	1.5%	368	1.6%	0.007	367	1.6%	385	1.7%	0.006	292	1.5%	295	1.5%	0.001
		Schizophrenia, schizotypal, delusional, and other non-mood psychotic disorders	444	1.9%	447	1.9%	0.001	471	2.1%	472	2.1%	0.000	357	1.9%	356	1.9%	0.000
		Manic episode	46	0.2%	49	0.2%	0.003	50	0.2%	58	0.3%	0.007	41	0.2%	56	0.3%	0.016
		Mood [affective] disorders	7,010	30.0%	7,034	30.1%	0.002	6,934	30.7%	7,029	31.1%	0.009	5,661	29.5%	5,795	30.2%	0.015
		Bipolar disorder	730	3.1%	704	3.0%	0.006	739	3.3%	773	3.4%	0.008	572	3.0%	580	3.0%	0.002
		Depressive episode	6,075	26.0%	6,115	26.1%	0.004	6,047	26.8%	6,079	26.9%	0.003	4,908	25.6%	5,004	26.1%	0.011
		Major depressive disorder, recurrent	1,781	7.6%	1,789	7.7%	0.001	1,818	8.1%	1,816	8.0%	0.000	1,460	7.6%	1,423	7.4%	0.007
		Persistent mood [affective] disorders	859	3.7%	869	3.7%	0.002	924	4.1%	919	4.1%	0.001	686	3.6%	693	3.6%	0.002
		Unspecified mood [affective] disorder	586	2.5%	572	2.4%	0.004	619	2.7%	609	2.7%	0.003	491	2.6%	477	2.5%	0.005
		Anxiety, dissociative, stress-related, somatoform and other nonpsychotic mental disorders	7,142	30.5%	7,234	30.9%	0.009	7,172	31.8%	7,302	32.3%	0.012	5,877	30.6%	5,898	30.7%	0.002
		Eating disorders	213	0.9%	210	0.9%	0.001	216	1.0%	219	1.0%	0.001	172	0.9%	160	0.8%	0.007
		Behavioral syndromes associated with physiological disturbances and physical factors	1,577	6.7%	1,552	6.6%	0.004	1,560	6.9%	1,580	7.0%	0.003	1,284	6.7%	1,264	6.6%	0.004
		Sleep disorders not due to a substance or known physiological condition	1,034	4.4%	1,004	4.3%	0.006	1,004	4.4%	1,022	4.5%	0.004	847	4.4%	861	4.5%	0.004
		Disorders of adult personality and behavior	317	1.4%	330	1.4%	0.005	334	1.5%	325	1.4%	0.003	261	1.4%	270	1.4%	0.004
		Attention-deficit hyperactivity disorders	401	1.7%	379	1.6%	0.007	396	1.8%	389	1.7%	0.002	308	1.6%	290	1.5%	0.008

		Behavioral and emotional disorders with onset usually occurring in childhood and adolescence	565	2.4%	583	2.5%	0.005	553	2.4%	540	2.4%	0.004	469	2.4%	482	2.5%	0.004
		Diseases of the nervous system	14,950	63.9%	14,944	63.9%	0.001	14,856	65.8%	14,947	66.2%	0.009	12,156	63.3%	12,141	63.2%	0.002
		Encephalitis, myelitis and encephalomyelitis	40	0.2%	39	0.2%	0.001	43	0.2%	44	0.2%	0.001	32	0.2%	38	0.2%	0.007
		Encephalitis, myelitis and encephalomyelitis in diseases classified elsewhere	10	0.0%	10	0.0%	0.000	10	0.0%	10	0.0%	0.000	10	0.1%	0	0.0%	0.032
		Extrapyramidal and movement disorders	1,297	5.5%	1,340	5.7%	0.008	1,353	6.0%	1,368	6.1%	0.003	1,089	5.7%	1,042	5.4%	0.011
		Alzheimer's disease	52	0.2%	61	0.3%	0.008	59	0.3%	61	0.3%	0.002	43	0.2%	45	0.2%	0.002
		Frontotemporal dementia	10	0.0%	10	0.0%	0.000	10	0.0%	10	0.0%	0.000	10	0.1%	10	0.1%	0.000
		Senile degeneration of brain, not elsewhere classified	10	0.0%	10	0.0%	0.000	10	0.0%	10	0.0%	0.000	10	0.1%	0	0.0%	0.032
		Degeneration of nervous system due to alcohol	0	0.0%	10	0.0%	0.029	0	0.0%	10	0.0%	0.030	0	0.0%	0	0.0%	NA
		Neurocognitive disorder with Lewy bodies	10	0.0%	10	0.0%	0.000	10	0.0%	10	0.0%	0.000	10	0.1%	10	0.1%	0.000
		Mild cognitive impairment of uncertain or unknown etiology	207	0.9%	205	0.9%	0.001	213	0.9%	224	1.0%	0.005	167	0.9%	176	0.9%	0.005
		Epilepsy and recurrent seizures	428	1.8%	446	1.9%	0.006	421	1.9%	403	1.8%	0.006	341	1.8%	341	1.8%	0.000
		Migraine	1,933	8.3%	1,955	8.4%	0.003	1,869	8.3%	1,863	8.2%	0.001	1,605	8.4%	1,646	8.6%	0.008
		Sleep disorders	9,025	38.6%	9,007	38.5%	0.002	8,873	39.3%	9,049	40.1%	0.016	7,338	38.2%	7,316	38.1%	0.002
		Chronic pain, not elsewhere classified	5,642	24.1%	5,743	24.6%	0.010	5,745	25.4%	5,809	25.7%	0.006	4,492	23.4%	4,457	23.2%	0.004
		Encephalopathy, unspecified	307	1.3%	314	1.3%	0.003	320	1.4%	347	1.5%	0.010	251	1.3%	250	1.3%	0.000
		Diseases of the eye and adnexa	7,758	33.2%	7,791	33.3%	0.003	8,079	35.8%	8,080	35.8%	0.000	6,210	32.3%	6,170	32.1%	0.004
		Disorders of optic nerve and visual pathways	346	1.5%	355	1.5%	0.003	359	1.6%	346	1.5%	0.005	272	1.4%	263	1.4%	0.004
		Diseases of the ear and mastoid process	5,724	24.5%	5,683	24.3%	0.004	5,809	25.7%	5,795	25.7%	0.001	4,780	24.9%	4,705	24.5%	0.009
		Diseases of the circulatory system	19,858	84.9%	19,837	84.8%	0.003	19,511	86.4%	19,592	86.8%	0.011	16,161	84.1%	16,147	84.1%	0.002

		Hypertensive diseases	18,785	80.3%	18,776	80.3%	0.001	18,503	81.9%	18,576	82.3%	0.008	15,236	79.3%	15,247	79.4%	0.001
		Hypertensive chronic kidney disease	2,050	8.8%	1,990	8.5%	0.009	2,264	10.0%	2,279	10.1%	0.002	1,665	8.7%	1,626	8.5%	0.007
		Ischemic heart diseases	5,538	23.7%	5,495	23.5%	0.004	5,646	25.0%	5,753	25.5%	0.011	4,392	22.9%	4,363	22.7%	0.004
		Other forms of heart disease	7,524	32.2%	7,467	31.9%	0.005	7,660	33.9%	7,806	34.6%	0.014	5,996	31.2%	5,916	30.8%	0.009
		Cerebrovascular diseases	2,433	10.4%	2,423	10.4%	0.001	2,503	11.1%	2,548	11.3%	0.006	1,866	9.7%	1,897	9.9%	0.005
		Diseases of arteries, arterioles and capillaries	3,341	14.3%	3,313	14.2%	0.003	3,515	15.6%	3,575	15.8%	0.007	2,699	14.1%	2,677	13.9%	0.003
		Diseases of the respiratory system	14,365	61.4%	14,377	61.5%	0.001	13,855	61.3%	13,867	61.4%	0.001	11,653	60.7%	11,731	61.1%	0.008
		Diseases of the digestive system	14,953	63.9%	14,959	64.0%	0.001	14,956	66.2%	15,053	66.7%	0.009	12,181	63.4%	12,178	63.4%	0.000
		Diseases of liver	4,083	17.5%	4,095	17.5%	0.001	4,116	18.2%	4,065	18.0%	0.006	3,284	17.1%	3,278	17.1%	0.001
		Diseases of the skin and subcutaneous tissue	11,158	47.7%	11,167	47.8%	0.001	11,383	50.4%	11,523	51.0%	0.012	8,938	46.5%	8,966	46.7%	0.003
		Psoriasis	693	3.0%	693	3.0%	0.000	697	3.1%	728	3.2%	0.008	536	2.8%	509	2.7%	0.009
		Diseases of the musculoskeletal system and connective tissue	17,376	74.3%	17,272	73.9%	0.010	17,242	76.3%	17,270	76.5%	0.003	14,114	73.5%	14,096	73.4%	0.002
		Rheumatoid arthritis with rheumatoid factor	126	0.5%	128	0.5%	0.001	138	0.6%	143	0.6%	0.003	111	0.6%	119	0.6%	0.005
		Other rheumatoid arthritis	582	2.5%	561	2.4%	0.006	590	2.6%	592	2.6%	0.001	482	2.5%	478	2.5%	0.001
		Systemic lupus erythematosus (SLE)	140	0.6%	166	0.7%	0.014	145	0.6%	160	0.7%	0.008	114	0.6%	109	0.6%	0.003
		Diseases of the genitourinary system	14,450	61.8%	14,352	61.4%	0.009	14,543	64.4%	14,563	64.5%	0.002	11,779	61.3%	11,754	61.2%	0.003
		Acute kidney failure and chronic kidney disease	4,919	21.0%	4,854	20.8%	0.007	5,267	23.3%	5,321	23.6%	0.006	4,014	20.9%	3,957	20.6%	0.007
		Diseases of male genital organs	4,152	17.8%	4,126	17.6%	0.003	4,237	18.8%	4,293	19.0%	0.006	3,364	17.5%	3,377	17.6%	0.002
		Noninflammatory disorders of female genital tract	4,654	19.9%	4,629	19.8%	0.003	4,774	21.1%	4,730	20.9%	0.005	3,737	19.5%	3,794	19.8%	0.007
		Pregnancy, childbirth and the puerperium	905	3.9%	925	4.0%	0.004	944	4.2%	919	4.1%	0.006	748	3.9%	708	3.7%	0.011
		Somnolence, stupor and coma	1,000	4.3%	942	4.0%	0.012	993	4.4%	1,051	4.7%	0.012	813	4.2%	820	4.3%	0.002

		Other symptoms and signs involving cognitive functions and awareness	1,806	7.7%	1,753	7.5%	0.009	1,869	8.3%	1,889	8.4%	0.003	1,473	7.7%	1,424	7.4%	0.010
		Age-related cognitive decline	15	0.1%	19	0.1%	0.006	20	0.1%	16	0.1%	0.006	14	0.1%	12	0.1%	0.004
		Suicidal ideations	334	1.4%	341	1.5%	0.003	344	1.5%	322	1.4%	0.008	266	1.4%	268	1.4%	0.001
		Nonsuicidal self-harm	0	0.0%	10	0.0%	0.029	0	0.0%	0	0.0%	NA	10	0.1%	10	0.1%	0.000
		Convulsions, not elsewhere classified	568	2.4%	604	2.6%	0.010	581	2.6%	567	2.5%	0.004	476	2.5%	493	2.6%	0.006
		Abnormal weight gain	1,266	5.4%	1,223	5.2%	0.008	1,244	5.5%	1,267	5.6%	0.004	1,022	5.3%	1,002	5.2%	0.005
		Intracranial injury	506	2.2%	534	2.3%	0.008	484	2.1%	476	2.1%	0.002	370	1.9%	393	2.0%	0.009
		Suicide attempt	31	0.1%	39	0.2%	0.009	35	0.2%	36	0.2%	0.001	31	0.2%	25	0.1%	0.008
		COVID-19	568	2.4%	564	2.4%	0.001	565	2.5%	528	2.3%	0.011	472	2.5%	478	2.5%	0.002
		Post COVID-19 condition	0	0.0%	10	0.0%	0.029	0	0.0%	0	0.0%	NA	0	0.0%	10	0.1%	0.032
		Intentional self-harm	34	0.1%	44	0.2%	0.010	40	0.2%	41	0.2%	0.001	32	0.2%	25	0.1%	0.009
		Contact with and (suspected) exposure to COVID-19	746	3.2%	767	3.3%	0.005	775	3.4%	773	3.4%	0.000	625	3.3%	627	3.3%	0.001
		Problems related to sleep	160	0.7%	143	0.6%	0.009	166	0.7%	171	0.8%	0.003	135	0.7%	131	0.7%	0.003
		Personal history of mental and behavioral disorders	219	0.9%	234	1.0%	0.007	218	1.0%	216	1.0%	0.001	181	0.9%	180	0.9%	0.001
		Personal history of psychological trauma, not elsewhere classified	42	0.2%	32	0.1%	0.011	49	0.2%	49	0.2%	0.000	40	0.2%	40	0.2%	0.000
		Personal history of self-harm	73	0.3%	76	0.3%	0.002	83	0.4%	90	0.4%	0.005	69	0.4%	70	0.4%	0.001
**Other medications**		aspirin	8,818	37.7%	8,740	37.4%	0.007	8,932	39.6%	8,981	39.8%	0.004	6,806	35.4%	6,899	35.9%	0.010
		glucagon	4,109	17.6%	4,056	17.3%	0.006	4,167	18.5%	4,132	18.3%	0.004	3,263	17.0%	3,136	16.3%	0.018
		ibuprofen	6,220	26.6%	6,238	26.7%	0.002	6,117	27.1%	6,091	27.0%	0.003	4,567	23.8%	4,606	24.0%	0.005
		ANTIOBESITY PREPARATIONS, EXCL. DIET PRODUCTS	703	3.0%	676	2.9%	0.007	649	2.9%	641	2.8%	0.002	555	2.9%	583	3.0%	0.009
		INSULINS AND ANALOGUES	12,270	52.5%	12,328	52.7%	0.005	12,195	54.0%	12,240	54.2%	0.004	9,788	51.0%	9,828	51.2%	0.004
		Biguanides	17,433	74.5%	17,638	75.4%	0.020	17,025	75.4%	17,154	76.0%	0.013	13,767	71.7%	13,943	72.6%	0.020
		Thiazolidinediones	1,525	6.5%	1,582	6.8%	0.010	1,658	7.3%	1,683	7.5%	0.004	1,108	5.8%	1,138	5.9%	0.007

		Dipeptidyl peptidase 4 (DPP-4) inhibitors	-	-	-	-	-	5,191	23.0%	5,204	23.0%	0.001	3,626	18.9%	3,764	19.6%	0.018
		Sodium-glucose co-transporter 2 (SGLT2) inhibitors	4,711	20.1%	4,806	20.6%	0.010	-	-	-	-	-	4,803	25.0%	4,916	25.6%	0.014
		Sulfonylureas	7,024	30.0%	7,057	30.2%	0.003	7,666	33.9%	7,815	34.6%	0.014	-	-	-	-	-
		ANTIARRHYTHMICS, CLASS I AND III	11,137	47.6%	11,091	47.4%	0.004	11,186	49.5%	11,258	49.8%	0.006	8,984	46.8%	8,972	46.7%	0.001
		ANTIHYPERTENSIVES	3,995	17.1%	3,982	17.0%	0.001	4,079	18.1%	4,114	18.2%	0.004	3,155	16.4%	3,072	16.0%	0.012
		DIURETICS	10,983	47.0%	10,995	47.0%	0.001	11,108	49.2%	11,226	49.7%	0.010	8,685	45.2%	8,748	45.5%	0.007
		BETA BLOCKING AGENTS	10,152	43.4%	10,107	43.2%	0.004	10,227	45.3%	10,247	45.4%	0.002	8,052	41.9%	8,028	41.8%	0.003
		CALCIUM CHANNEL BLOCKERS	7,504	32.1%	7,509	32.1%	0.000	7,667	33.9%	7,765	34.4%	0.009	5,926	30.9%	5,904	30.7%	0.002
		AGENTS ACTING ON THE RENIN-ANGIOTENSIN SYSTEM	15,166	64.9%	15,135	64.7%	0.003	15,118	66.9%	15,203	67.3%	0.008	11,927	62.1%	11,980	62.4%	0.006
		LIPID MODIFYING AGENTS	16,549	70.8%	16,620	71.1%	0.007	16,442	72.8%	16,520	73.1%	0.008	13,209	68.8%	13,292	69.2%	0.009
		CORTICOSTEROIDS FOR SYSTEMIC USE	13,570	58.0%	13,647	58.4%	0.007	13,570	60.1%	13,669	60.5%	0.009	10,999	57.3%	11,010	57.3%	0.001
		THYROID THERAPY	3,457	14.8%	3,440	14.7%	0.002	3,467	15.4%	3,451	15.3%	0.002	2,847	14.8%	2,842	14.8%	0.001
		ANTIINFECTIVES FOR SYSTEMIC USE	18,415	78.7%	18,398	78.7%	0.002	18,146	80.3%	18,207	80.6%	0.007	14,974	78.0%	14,937	77.8%	0.005
		ANTINEOPLASTIC AGENTS	2,537	10.8%	2,588	11.1%	0.007	2,614	11.6%	2,619	11.6%	0.001	2,022	10.5%	2,047	10.7%	0.004
		Estrogens	678	2.9%	664	2.8%	0.004	595	2.6%	573	2.5%	0.006	549	2.9%	549	2.9%	0.000
		Progestogens	705	3.0%	665	2.8%	0.010	701	3.1%	685	3.0%	0.004	585	3.0%	589	3.1%	0.001
		IMMUNOSUPPRESSANTS	1,246	5.3%	1,239	5.3%	0.001	1,258	5.6%	1,286	5.7%	0.005	970	5.1%	1,002	5.2%	0.008
		ANTIINFLAMMATORY AND ANTIRHEUMATIC PRODUCTS	12,410	53.1%	12,432	53.2%	0.002	12,294	54.4%	12,410	55.0%	0.010	9,803	51.0%	9,896	51.5%	0.010
		OPIOIDS	12,628	54.0%	12,668	54.2%	0.003	12,631	55.9%	12,685	56.2%	0.005	10,103	52.6%	10,179	53.0%	0.008
		OTHER ANALGESICS AND ANTIPYRETICS	16,045	68.6%	16,039	68.6%	0.001	15,840	70.1%	15,847	70.2%	0.001	12,782	66.6%	12,868	67.0%	0.010
		ANTIMIGRAINE PREPARATIONS	1,888	8.1%	1,873	8.0%	0.002	1,890	8.4%	1,953	8.6%	0.010	1,506	7.8%	1,543	8.0%	0.007
		ANTIEPILEPTICS	7,809	33.4%	7,896	33.8%	0.008	7,803	34.6%	7,894	35.0%	0.008	6,155	32.0%	6,207	32.3%	0.006

		ANTI-PARKINSON DRUGS	1,118	4.8%	1,138	4.9%	0.004	1,100	4.9%	1,119	5.0%	0.004	845	4.4%	843	4.4%	0.001
		ANTIPSYCHOTICS	3,399	14.5%	3,404	14.6%	0.001	3,445	15.3%	3,471	15.4%	0.003	2,674	13.9%	2,703	14.1%	0.004
		Lithium	25	0.1%	21	0.1%	0.005	29	0.1%	31	0.1%	0.002	17	0.1%	25	0.1%	0.013
		HYPNOTICS AND SEDATIVES	9,686	41.4%	9,742	41.7%	0.005	9,770	43.3%	9,859	43.7%	0.008	7,751	40.4%	7,779	40.5%	0.003
		Non-selective monoamine reuptake inhibitors	1,788	7.6%	1,785	7.6%	0.000	1,807	8.0%	1,831	8.1%	0.004	1,326	6.9%	1,370	7.1%	0.009
		Selective serotonin reuptake inhibitors	5,465	23.4%	5,435	23.2%	0.003	5,397	23.9%	5,427	24.0%	0.003	3,776	19.7%	3,851	20.1%	0.010
		Monoamine oxidase inhibitors, non-selective	10	0.0%	10	0.0%	0.000	10	0.0%	10	0.0%	0.000	10	0.1%	10	0.1%	0.000
		Other antidepressants	5,736	24.5%	5,758	24.6%	0.002	5,696	25.2%	5,769	25.5%	0.007	4,316	22.5%	4,374	22.8%	0.007
		PSYCHOSTIMULANTS, AGENTS USED FOR ADHD AND NOOTROPICS	1,479	6.3%	1,525	6.5%	0.008	1,467	6.5%	1,467	6.5%	0.000	1,025	5.3%	1,036	5.4%	0.003
		ANTI-DEMENTIA DRUGS	161	0.7%	144	0.6%	0.009	171	0.8%	164	0.7%	0.004	122	0.6%	127	0.7%	0.003
		DRUGS USED IN ADDICTIVE DISORDERS	1,982	8.5%	2,008	8.6%	0.004	1,978	8.8%	2,048	9.1%	0.011	1,545	8.0%	1,570	8.2%	0.005
		ANTIPARASITIC PRODUCTS, INSECTICIDES AND REPELLENTS	3,579	15.3%	3,600	15.4%	0.002	3,643	16.1%	3,684	16.3%	0.005	2,894	15.1%	2,858	14.9%	0.005
		ANTIHISTAMINES FOR SYSTEMIC USE	10,857	46.4%	10,846	46.4%	0.001	10,894	48.2%	10,940	48.4%	0.004	8,650	45.0%	8,610	44.8%	0.004
		Antidotes	4,291	18.3%	4,284	18.3%	0.001	4,314	19.1%	4,347	19.2%	0.004	3,450	18.0%	3,400	17.7%	0.007

Values are N (%) unless stated otherwise. SMD: standardised mean difference.

Supplementary Table S3. Full study results

Outcome		Comparator	HR (95% CI)	p-value	p_{adj}	Inc Semaglutide	Inc Comparator
**Alcohol misuse**	Any	Empagliflozin	0.84 (0.71-0.99)	0.033	0.62	1.24 (1.10-1.39)	1.47 (1.32-1.64)
		Glipizide	0.73 (0.61-0.88)	0.00068	0.013	1.06 (0.92-1.22)	1.45 (1.29-1.64)
		Sitagliptin	0.81 (0.69-0.95)	0.0095	0.18	1.16 (1.03-1.30)	1.43 (1.28-1.60)
	First	Empagliflozin	0.90 (0.67-1.20)	0.47	1	0.41 (0.34-0.51)	0.46 (0.37-0.56)
		Glipizide	0.74 (0.54-1.01)	0.058	1	0.38 (0.30-0.48)	0.52 (0.43-0.65)
		Sitagliptin	0.82 (0.62-1.08)	0.16	1	0.42 (0.34-0.51)	0.51 (0.42-0.62)
**All-cause mortality**		Empagliflozin	0.86 (0.75-0.99)	0.035	0.77	1.76 (1.59-1.94)	2.04 (1.86-2.24)
		Glipizide	0.55 (0.47-0.64)	9.90E-15	2.20E-13	1.38 (1.23-1.56)	2.48 (2.26-2.71)
		Sitagliptin	0.58 (0.50-0.66)	4.30E-16	9.50E-15	1.48 (1.33-1.65)	2.55 (2.35-2.76)
**Anxiety disorders**	Any	Empagliflozin	0.98 (0.94-1.02)	0.29	1	19.08 (18.56-19.60)	19.48 (18.96-20.01)
		Glipizide	1.02 (0.98-1.07)	0.32	1	19.03 (18.48-19.60)	18.63 (18.08-19.20)
		Sitagliptin	0.96 (0.92-1.00)	0.056	1	18.63 (18.13-19.15)	19.34 (18.83-19.87)
	First	Empagliflozin	0.99 (0.90-1.09)	0.84	1	5.94 (5.57-6.33)	5.96 (5.59-6.36)
		Glipizide	0.97 (0.88-1.07)	0.53	1	5.95 (5.56-6.37)	6.10 (5.70-6.53)
		Sitagliptin	0.92 (0.84-1.01)	0.075	1	5.86 (5.50-6.24)	6.32 (5.94-6.71)
**Any NCOs**	Any	Empagliflozin	1.02 (0.97-1.08)	0.42	1	10.82 (10.41-11.23)	10.59 (10.18-11.00)
		Glipizide	1.05 (0.99-1.12)	0.11	1	10.31 (9.88-10.75)	9.82 (9.39-10.26)
		Sitagliptin	0.96 (0.91-1.02)	0.19	1	10.42 (10.03-10.82)	10.83 (10.43-11.24)
	First	Empagliflozin	0.99 (0.90-1.08)	0.76	1	7.08 (6.67-7.51)	7.19 (6.78-7.62)
		Glipizide	1.03 (0.93-1.13)	0.6	1	6.00 (5.61-6.41)	5.86 (5.48-6.28)
		Sitagliptin	0.97 (0.88-1.05)	0.43	1	6.08 (5.72-6.46)	6.30 (5.94-6.69)
**Bipolar disorder**	Any	Empagliflozin	0.92 (0.81-1.05)	0.21	1	1.99 (1.82-2.19)	2.16 (1.98-2.36)
		Glipizide	0.91 (0.79-1.05)	0.21	1	1.90 (1.71-2.10)	2.08 (1.89-2.30)
		Sitagliptin	0.95 (0.84-1.08)	0.48	1	2.02 (1.84-2.21)	2.11 (1.93-2.31)
	First	Empagliflozin	0.91 (0.66-1.26)	0.58	1	0.32 (0.26-0.41)	0.36 (0.28-0.45)

		Glipizide	0.76 (0.55-1.06)	0.1	1	0.35 (0.28-0.45)	0.46 (0.37-0.58)
		Sitagliptin	0.79 (0.59-1.06)	0.12	1	0.36 (0.29-0.45)	0.46 (0.38-0.56)
**Cannabis misuse**	Any	Empagliflozin	0.91 (0.70-1.17)	0.45	1	0.52 (0.44-0.63)	0.57 (0.48-0.68)
		Glipizide	0.81 (0.62-1.05)	0.12	1	0.54 (0.45-0.66)	0.67 (0.56-0.80)
		Sitagliptin	0.69 (0.55-0.88)	0.0024	0.045	0.51 (0.43-0.62)	0.74 (0.63-0.86)
	First	Empagliflozin	1.03 (0.72-1.45)	0.89	1	0.30 (0.23-0.38)	0.28 (0.22-0.37)
		Glipizide	0.78 (0.54-1.13)	0.18	1	0.28 (0.22-0.37)	0.36 (0.29-0.47)
		Sitagliptin	0.73 (0.53-1.00)	0.05	1	0.30 (0.24-0.38)	0.41 (0.34-0.51)
**Cognitive deficit**	Any	Empagliflozin	0.96 (0.86-1.08)	0.51	1	3.08 (2.85-3.33)	3.20 (2.96-3.46)
		Glipizide	0.72 (0.63-0.81)	6.00E-08	0.00012	2.71 (2.48-2.97)	3.77 (3.48-4.07)
		Sitagliptin	0.72 (0.64-0.80)	9.30E-10	1.70E-07	2.88 (2.66-3.12)	3.98 (3.72-4.26)
	First	Empagliflozin	1.02 (0.94-1.10)	0.61	1	5.93 (5.63-6.25)	5.81 (5.51-6.12)
		Glipizide	0.82 (0.76-0.90)	6.50E-06	0.00014	5.34 (5.03-5.67)	6.43 (6.08-6.79)
		Sitagliptin	0.80 (0.75-0.87)	9.20E-09	2.00E-07	5.46 (5.17-5.76)	6.73 (6.41-7.06)
**Dementia**	First	Empagliflozin	0.91 (0.69-1.21)	0.53	1	0.43 (0.35-0.53)	0.47 (0.39-0.58)
		Glipizide	0.63 (0.46-0.86)	0.0034	0.075	0.34 (0.27-0.44)	0.54 (0.44-0.66)
		Sitagliptin	0.52 (0.40-0.68)	9.60E-07	2.10E-05	0.37 (0.30-0.46)	0.71 (0.61-0.83)
**Depression**	Any	Empagliflozin	1.02 (0.97-1.06)	0.48	1	17.06 (16.57-17.56)	16.82 (16.33-17.32)
		Glipizide	0.99 (0.95-1.04)	0.79	1	16.58 (16.06-17.12)	16.71 (16.18-17.26)
		Sitagliptin	0.93 (0.89-0.98)	0.0027	0.052	16.52 (16.05-17.01)	17.61 (17.12-18.12)
	First	Empagliflozin	0.93 (0.83-1.04)	0.19	1	4.03 (3.76-4.37)	4.33 (4.02-4.66)
		Glipizide	0.86 (0.77-0.97)	0.014	0.3	3.94 (3.67-4.33)	4.53 (4.19-4.90)
		Sitagliptin	0.84 (0.75-0.93)	0.00074	0.016	3.99 (3.72-4.31)	4.74 (4.42-5.08)
**Encephalitis**	Any	Empagliflozin	0.50 (0.20-1.23)	0.12	1	0.032 (0.015-0.067)	0.064 (0.038-0.11)
		Glipizide	0.40 (0.17-0.97)	0.033	0.63	0.037 (0.018-0.078)	0.092 (0.057-0.15)
		Sitagliptin	0.49 (0.23-1.05)	0.057	1	0.044 (0.024-0.082)	0.089 (0.058-0.14)
	First	Empagliflozin	0.62 (0.20-1.89)	0.39	1	0.023 (0.0095-0.055)	0.037 (0.018-0.073)
		Glipizide	0.26 (0.074-0.95)	0.024	0.52	0.016 (0.0052-0.05)	0.06 (0.033-0.11)
		Sitagliptin	0.35 (0.13-0.96)	0.03	0.67	0.022 (0.0092-0.053)	0.063 (0.037-0.11)

**Epilepsy/seizure**	Any	Empagliflozin	1.16 (1.00-1.34)	0.043	0.81	1.82 (1.65-2.01)	1.57 (1.42-1.75)
		Glipizide	0.95 (0.81-1.10)	0.46	1	1.81 (1.63-2.01)	1.91 (1.72-2.11)
		Sitagliptin	0.88 (0.77-1.01)	0.064	1	1.78 (1.62-1.96)	2.01 (1.83-2.20)
	First	Empagliflozin	0.94 (0.73-1.22)	0.66	1	0.52 (0.44-0.63)	0.56 (0.46-0.67)
		Glipizide	1.00 (0.76-1.31)	0.98	1	0.57 (0.48-0.70)	0.57 (0.46-0.69)
		Sitagliptin	0.77 (0.61-0.98)	0.036	0.79	0.53 (0.44-0.64)	0.69 (0.58-0.81)
**Insomnia**	Any	Empagliflozin	0.93 (0.86-0.99)	0.034	0.65	6.57 (6.25-6.91)	7.10 (6.77-7.45)
		Glipizide	0.92 (0.85-1.00)	0.044	0.84	6.47 (6.12-6.83)	6.99 (6.63-7.37)
		Sitagliptin	0.86 (0.80-0.92)	9.50E-06	0.00018	6.63 (6.32-6.96)	7.70 (7.36-8.05)
	First	Empagliflozin	0.96 (0.85-1.08)	0.46	1	2.80 (2.57-3.04)	2.93 (2.70-3.18)
		Glipizide	0.97 (0.85-1.10)	0.62	1	2.83 (2.59-3.10)	2.92 (2.67-3.20)
		Sitagliptin	0.86 (0.77-0.97)	0.012	0.27	2.83 (2.61-3.07)	3.26 (3.02-3.52)
**Intracranial haemorrhage**	Any	Empagliflozin	0.89 (0.67-1.19)	0.44	1	0.40 (0.33-0.50)	0.45 (0.37-0.55)
		Glipizide	0.94 (0.68-1.30)	0.71	1	0.39 (0.31-0.50)	0.42 (0.33-0.52)
		Sitagliptin	0.74 (0.57-0.97)	0.027	0.50	0.42 (0.34-0.51)	0.57 (0.48-0.67)
	First	Empagliflozin	0.93 (0.65-1.33)	0.68	1	0.26 (0.20-0.34)	0.28 (0.22-0.36)
		Glipizide	1.02 (0.67-1.55)	0.92	1	0.24 (0.18-0.33)	0.24 (0.18-0.32)
		Sitagliptin	0.80 (0.56-1.15)	0.23	1	0.24 (0.19-0.32)	0.30 (0.24-0.39)
**Ischaemic stroke**	Any	Empagliflozin	0.98 (0.88-1.09)	0.74	1	3.02 (2.80-3.26)	3.07 (2.85-3.31)
		Glipizide	1.02 (0.91-1.15)	0.74	1	2.94 (2.71-3.19)	2.86 (2.63-3.11)
		Sitagliptin	0.87 (0.79-0.97)	0.012	0.23	2.86 (2.65-3.09)	3.26 (3.04-3.50)
	First	Empagliflozin	0.99 (0.81-1.19)	0.88	1	1.00 (0.87-1.14)	1.01 (0.89-1.16)
		Glipizide	0.87 (0.70-1.07)	0.18	1	0.95 (0.81-1.10)	1.09 (0.95-1.26)
		Sitagliptin	0.75 (0.62-0.90)	0.0017	0.037	0.95 (0.84-1.10)	1.26 (1.12-1.42)
**Migraine**	Any	Empagliflozin	1.03 (0.93-1.13)	0.57	1	3.86 (3.62-4.12)	3.76 (3.52-4.02)
		Glipizide	1.20 (1.08-1.33)	7.00E-04	0.013	4.15 (3.88-4.45)	3.49 (3.23-3.77)
		Sitagliptin	1.00 (0.91-1.09)	0.95	1	3.96 (3.71-4.22)	3.99 (3.74-4.25)
	First	Empagliflozin	1.01 (0.83-1.22)	0.94	1	1.04 (0.91-1.19)	1.03 (0.90-1.18)
		Glipizide	1.10 (0.89-1.36)	0.39	1	1.06 (0.92-1.23)	0.96 (0.83-1.13)

		Sitagliptin	0.96 (0.80-1.15)	0.66	1	1.07 (0.94-1.22)	1.11 (0.97-1.26)
**Myoneural junction/muscle disorder**	First	Empagliflozin	0.87 (0.61-1.24)	0.44	1	0.26 (0.20-0.34)	0.30 (0.24-0.39)
		Glipizide	0.96 (0.65-1.42)	0.83	1	0.27 (0.22-0.37)	0.28 (0.22-0.37)
		Sitagliptin	0.87 (0.61-1.25)	0.46	1	0.26 (0.20-0.34)	0.30 (0.23-0.38)
**Nerve/nerve root/plexus disorder**	Any	Empagliflozin	1.04 (0.96-1.14)	0.31	1	5.05 (4.77-5.35)	4.83 (4.56-5.13)
		Glipizide	0.99 (0.90-1.09)	0.87	1	4.70 (4.40-5.01)	4.73 (4.44-5.05)
		Sitagliptin	0.99 (0.91-1.07)	0.76	1	4.91 (4.64-5.20)	4.98 (4.70-5.27)
	First	Empagliflozin	1.00 (0.88-1.14)	0.98	1	2.53 (2.31-2.77)	2.52 (2.30-2.76)
		Glipizide	0.93 (0.81-1.06)	0.29	1	2.49 (2.26-2.74)	2.67 (2.43-2.94)
		Sitagliptin	0.89 (0.79-1.01)	0.072	1	2.48 (2.27-2.71)	2.77 (2.56-3.03)
**Nicotine misuse**	Any	Empagliflozin	0.85 (0.80-0.91)	1.10E-06	2.10E-05	7.91 (7.56-8.28)	9.23 (8.85-9.62)
		Glipizide	0.86 (0.80-0.92)	2.20E-05	0.00042	8.02 (7.64-8.42)	9.25 (8.84-9.68)
		Sitagliptin	0.86 (0.81-0.92)	3.30E-06	6.40E-05	7.96 (7.62-8.32)	9.18 (8.81-9.57)
	First	Empagliflozin	0.77 (0.65-0.90)	0.0011	0.024	1.48 (1.31-1.66)	1.91 (1.72-2.12)
		Glipizide	0.72 (0.61-0.85)	0.00012	0.0027	1.55 (1.37-1.76)	2.13 (1.91-2.37)
		Sitagliptin	0.82 (0.70-0.95)	0.011	0.23	1.60 (1.43-1.79)	1.94 (1.75-2.15)
**Obsessive-compulsive disorder**	Any	Empagliflozin	0.73 (0.49-1.09)	0.12	1	0.19 (0.14-0.25)	0.26 (0.20-0.33)
		Glipizide	0.60 (0.39-0.92)	0.018	0.34	0.18 (0.13-0.25)	0.29 (0.23-0.38)
		Sitagliptin	0.80 (0.55-1.17)	0.25	1	0.21 (0.16-0.28)	0.26 (0.20-0.34)
	First	Empagliflozin	0.76 (0.39-1.45)	0.4	1	0.073 (0.045-0.12)	0.097 (0.068-0.15)
		Glipizide	0.92 (0.47-1.79)	0.81	1	0.092 (0.057-0.15)	0.10 (0.074-0.17)
		Sitagliptin	0.68 (0.37-1.24)	0.2	1	0.079 (0.05-0.13)	0.12 (0.079-0.17)
**Opioid misuse**	Any	Empagliflozin	0.93 (0.77-1.12)	0.46	1	0.95 (0.83-1.09)	1.02 (0.89-1.16)
		Glipizide	0.93 (0.75-1.16)	0.53	1	0.86 (0.74-1.01)	0.93 (0.80-1.08)
		Sitagliptin	1.02 (0.84-1.24)	0.84	1	0.94 (0.82-1.07)	0.92 (0.80-1.05)
	First	Empagliflozin	0.94 (0.70-1.26)	0.67	1	0.39 (0.32-0.48)	0.41 (0.34-0.51)
		Glipizide	0.86 (0.63-1.18)	0.34	1	0.40 (0.32-0.51)	0.47 (0.38-0.58)
		Sitagliptin	0.95 (0.71-1.27)	0.72	1	0.41 (0.33-0.50)	0.43 (0.35-0.53)
**Parkinsonism**	First	Empagliflozin	1.06 (0.64-1.76)	0.82	1	0.14 (0.11-0.21)	0.13 (0.093-0.19)

		Glipizide	0.71 (0.41-1.24)	0.23	1	0.12 (0.084-0.19)	0.17 (0.12-0.24)
		Sitagliptin	0.63 (0.39-1.04)	0.068	1	0.12 (0.092-0.18)	0.18 (0.13-0.25)
**Psychosis**	Any	Empagliflozin	0.92 (0.77-1.10)	0.35	1	1.02 (0.90-1.17)	1.12 (0.99-1.27)
		Glipizide	0.76 (0.63-0.93)	0.0071	0.13	0.93 (0.81-1.08)	1.22 (1.07-1.39)
		Sitagliptin	0.89 (0.74-1.06)	0.2	1	0.96 (0.84-1.09)	1.08 (0.96-1.23)
	First	Empagliflozin	0.77 (0.52-1.14)	0.19	1	0.21 (0.16-0.29)	0.27 (0.22-0.36)
		Glipizide	0.59 (0.39-0.88)	0.0087	0.19	0.21 (0.16-0.29)	0.35 (0.27-0.45)
		Sitagliptin	0.63 (0.44-0.92)	0.015	0.33	0.21 (0.16-0.28)	0.32 (0.26-0.41)
**Stimulants misuse**	Any	Empagliflozin	0.70 (0.53-0.92)	0.01	0.19	0.38 (0.31-0.47)	0.55 (0.46-0.65)
		Glipizide	0.69 (0.52-0.91)	0.0084	0.16	0.44 (0.35-0.54)	0.64 (0.53-0.76)
		Sitagliptin	0.76 (0.58-0.99)	0.042	0.8	0.42 (0.34-0.51)	0.55 (0.46-0.65)
	First	Empagliflozin	0.51 (0.30-0.87)	0.012	0.26	0.092 (0.059-0.14)	0.18 (0.13-0.25)
		Glipizide	0.52 (0.32-0.85)	0.0081	0.18	0.13 (0.087-0.19)	0.25 (0.19-0.33)
		Sitagliptin	0.59 (0.36-0.95)	0.026	0.58	0.12 (0.082-0.18)	0.21 (0.15-0.27)
**Suicidal ideation**	Any	Empagliflozin	1.06 (0.80-1.40)	0.7	1	0.46 (0.38-0.56)	0.44 (0.36-0.54)
		Glipizide	0.52 (0.38-0.70)	1.20E-05	0.00022	0.34 (0.27-0.44)	0.66 (0.55-0.79)
		Sitagliptin	0.71 (0.54-0.93)	0.012	0.24	0.39 (0.32-0.48)	0.55 (0.46-0.65)
	First	Empagliflozin	0.96 (0.66-1.40)	0.83	1	0.24 (0.18-0.31)	0.26 (0.20-0.33)
		Glipizide	0.54 (0.36-0.83)	0.0036	0.08	0.18 (0.13-0.26)	0.34 (0.26-0.44)
		Sitagliptin	0.75 (0.52-1.07)	0.11	1	0.23 (0.18-0.30)	0.31 (0.24-0.39)

HR: hazard ratio (95% confidence interval), ICH: intracranial haemorrhage, Inc: incidence (95% confidence interval), MNJ: myoneural junction, NCOs: negative control outcomes, OCD: obsessive-compulsive disorder, p_{adj}: p-value adjusted for multiple testing via Bonferroni correction.

Supplementary Table S4. Study results for diagnostic subcategories of cognitive deficit and dementia

**Outcome**		**Semaglutide vs Sitagliptin**		**Semaglutide vs Empagliflozin**		**Semaglutide vs Glipizide**	
		HR (95% CI)	p-value	HR (95% CI)	p-value	HR (95% CI)	p-value
Cognitive deficit	First	0.80 (0.75-0.87)	9.20E-09	1.02 (0.94-1.10)	0.61	0.82 (0.76-0.90)	6.50E-06
	Any	0.72 (0.64-0.80)	9.30E-10	0.96 (0.86-1.08)	0.51	0.72 (0.63-0.81)	6.00E-08
Dementia	First	0.52 (0.40-0.68)	9.60E-07	0.91 (0.69-1.21)	0.53	0.63 (0.46-0.86)	0.0034
F01 Vascular dementia	First	0.56 (0.33-0.95)	0.029	0.59 (0.35-1.00)	0.046	0.67 (0.40-1.12)	0.12
F02 Dementia in other diseases classified elsewhere	First	0.72 (0.46-1.15)	0.17	1.07 (0.69-1.65)	0.76	0.48 (0.29-0.78)	0.0021
F03 Unspecified dementia	First	0.42 (0.31-0.58)	1.30E-08	0.98 (0.69-1.38)	0.9	0.47 (0.32-0.67)	2.00E-05
F05 Delirium due to known physiological condition	First	0.63 (0.43-0.92)	0.017	1.20 (0.80-1.80)	0.37	0.57 (0.37-0.87)	0.0082
	Any	0.60 (0.42-0.86)	0.0045	0.98 (0.68-1.40)	0.9	0.57 (0.39-0.85)	0.0045
F06.8 Other specified mental disorders due to known physiological condition	First	0.77 (0.35-1.70)	0.52	0.85 (0.39-1.84)	0.68	0.88 (0.36-2.16)	0.78
	Any	0.89 (0.49-1.64)	0.71	0.82 (0.47-1.42)	0.47	1.23 (0.64-2.37)	0.54
G30 Alzheimer's disease	First	0.55 (0.29-1.03)	0.054	0.84 (0.44-1.61)	0.61	0.43 (0.23-0.79)	0.0045
G31.0 Frontotemporal dementia	First	3.00 (0.31-28.87)	0.31	3.54 (0.74-17.05)	0.083	0.64 (0.11-3.84)	0.62
G31.83 Dementia with Lewy bodies	First	4.03 (0.45-36.09)	0.16	1.66 (0.40-6.96)	0.48	0.59 (0.14-2.47)	0.46
G31.84 Mild cognitive impairment, so stated	First	0.66 (0.48-0.91)	0.01	0.93 (0.66-1.32)	0.7	0.73 (0.49-1.09)	0.12
	Any	0.71 (0.55-0.91)	0.0072	0.96 (0.74-1.25)	0.75	0.85 (0.63-1.13)	0.26
G93.40 Encephalopathy, unspecified	First	0.63 (0.50-0.79)	5.70E-05	1.08 (0.85-1.37)	0.54	0.58 (0.45-0.74)	6.60E-06
	Any	0.61 (0.50-0.75)	1.00E-06	1.04 (0.84-1.28)	0.71	0.59 (0.48-0.74)	1.20E-06
R40 Somnolence, stupor and coma	First	0.90 (0.74-1.10)	0.32	0.90 (0.73-1.10)	0.3	0.71 (0.57-0.87)	0.00094
	Any	0.83 (0.70-0.98)	0.031	0.86 (0.73-1.02)	0.089	0.73 (0.61-0.87)	0.00062
R41 Other symptoms and signs involving cognitive functions and awareness	First	0.71 (0.63-0.81)	9.10E-08	1.03 (0.91-1.17)	0.61	0.68 (0.59-0.78)	1.90E-08
	Any	0.81 (0.74-0.88)	3.10E-06	1.07 (0.98-1.17)	0.15	0.85 (0.77-0.93)	0.00089
R48 Dyslexia and other symbolic dysfunctions, not elsewhere classified	First	1.22 (0.57-2.62)	0.6	0.88 (0.45-1.73)	0.71	1.05 (0.49-2.23)	0.9
	Any	0.88 (0.46-1.69)	0.7	0.89 (0.47-1.69)	0.72	0.97 (0.46-2.04)	0.95

HR: hazard ratio (95% confidence interval, CI)

Supplementary Table S5. Secondary analysis, <65yo; ≥65yo

a) Baseline characteristics after matching, <65yo

	**Semaglutide**	**Sitagliptin**		**Semaglutide**	**Empagliflozin**		**Semaglutide**	**Glipizide**	
Number	16358	16358	*SMD*	16950	16950	*SMD*	13683	13683	*SMD*
Age; mean (SD); y	51.46 (9.48)	51.46 (9.79)	0.00072	51.59 (9.36)	51.58 (9.33)	0.0013	50.98 (9.72)	50.99 (9.82)	0.0013
Sex									
Female	51.52	51.77	0.005	49.91	49.71	0.004	50.47	50.92	0.0091
Male	41.01	40.57	0.009	41.66	41.65	0.00036	40.72	40.64	0.0016
Other	7.47	7.66	0.0072	8.43	8.64	0.0078	8.81	8.43	0.013
Race									
White	56.06	56.03	0.00062	56.6	56.7	0.002	57.17	57.12	0.0012
Black or African American	18.18	18.08	0.0025	17.67	17.38	0.0078	18.67	18.71	0.00094
Asian	4.18	4.21	0.0015	4.03	3.91	0.006	4.43	4.51	0.0039
American Indian or Alaska Native	0.42	0.48	0.0091	0.42	0.4	0.0037	0.33	0.35	0.0038
Native Hawaiian or Other Pacific Islander	1.47	1.43	0.0031	1.29	1.27	0.0016	1.62	1.76	0.011
Unknown	15.81	15.87	0.0015	16.24	16.43	0.0051	13.62	13.52	0.003
Ethnicity									
Hispanic or Latino	7.95	8.13	0.0067	7.91	7.89	0.00044	8.73	8.46	0.0099
Not Hispanic of Latino	65.58	65.46	0.0026	66.15	66.18	0.00075	68.78	69.72	0.02
Unknown	26.47	26.41	0.0014	25.95	25.92	0.00054	22.49	21.82	0.016
Certain infectious and parasitic diseases	42.1	41.94	0.0031	41.37	41.07	0.0061	38.46	38.65	0.0039
ANTIOBESITY PREPARATIONS; EXCL. DIET PRODUCTS	3.26	3.23	0.0017	3.41	3.32	0.0049	3.54	3.46	0.0044
INSULINS AND ANALOGUES	49.91	49.73	0.0037	50.01	49.97	0.00083	47.54	48.23	0.014
Biguanides	77.33	77.62	0.007	77.55	77.55	0.00014	74.07	73.75	0.0073
Thiazolidinediones	5.81	5.48	0.015	5.88	5.93	0.0023	4.41	4.55	0.0064

Dipeptidyl peptidase 4 (DPP-4) inhibitors	NA	NA	NA	20.05	20.44	0.0097	17.04	17.79	0.02
Sodium-glucose co-transporter 2 (SGLT2) inhibitors	0	0	NA	NA	NA	NA	19.34	19.45	0.0026
Sulfonylureas	30.21	29.91	0.0067	30.18	30.79	0.013	NA	NA	NA
Neoplasms	32.11	32.22	0.0022	31.69	31.33	0.0076	29.2	29.77	0.013
ANTIARRHYTHMICS; CLASS I AND III	46.64	46.93	0.0058	46.64	46.47	0.0034	44.01	45.14	0.023
ANTIHYPERTENSIVES	16.18	16.41	0.0063	15.79	16.15	0.0098	15	15.03	0.00061
DIURETICS	43.75	44.03	0.0057	43.82	43.93	0.0023	39.98	40.39	0.0085
BETA BLOCKING AGENTS	38.74	39.05	0.0064	38.9	39.04	0.0029	35.97	36.71	0.015
CALCIUM CHANNEL BLOCKERS	29.36	29.89	0.012	29.25	29.49	0.0052	26.63	27.08	0.01
AGENTS ACTING ON THE RENIN-ANGIOTENSIN SYSTEM	62.17	61.96	0.0044	62.99	63.2	0.0043	58.58	58.95	0.0076
LIPID MODIFYING AGENTS	67.63	67.76	0.0027	68.45	68.81	0.0076	64.62	64.58	0.00092
Divorced	5.62	5.66	0.0016	5.82	5.83	5.00E-04	5.96	6.07	0.0046
Diseases of the blood and blood-forming organs and certain disorders involving the immune mechanism	31.91	31.89	0.00026	31.04	30.77	0.0059	29.36	30.01	0.014
Certain disorders involving the immune mechanism	3.63	3.54	0.0049	3.48	3.48	0	3.26	3.58	0.018
Disorders of thyroid gland	22.42	23.04	0.015	22.27	22.35	0.002	22.24	22.31	0.0018
Endocrine; nutritional and metabolic diseases	100	100	NA	100	100	NA	100	100	NA
Type 1 diabetes mellitus	9.57	9.66	0.0031	9.89	9.79	0.0034	8.97	8.81	0.0057
Vitamin D deficiency	26.44	26.38	0.0014	26.11	26.3	0.0043	25.85	25.75	0.0022
Overweight; obesity and other hyperalimentation	64.17	63.99	0.0038	63.8	64.12	0.0066	63.12	63.92	0.017
Metabolic disorders	81.6	81.57	0.00079	81.99	82.12	0.0034	80.3	80.47	0.0042
Disorders of lipoprotein metabolism and other lipidemias	76.62	76.64	0.00029	77.31	77.5	0.0045	75.28	75.65	0.0087
Other disorders of fluid; electrolyte and acid-base balance	18.47	18.54	0.0019	18.16	18.31	0.0038	16.71	16.82	0.0029
Metabolic syndrome and other insulin resistance	4.03	4.02	0.00062	4.35	4.41	0.0029	4.22	4.15	0.0036
Vascular dementia	0.092	0.079	0.0042	0.094	0.11	0.0037	0.073	0.095	0.0076
Mental; Behavioral and Neurodevelopmental disorders	54.63	54.41	0.0043	54.54	54.57	0.00071	52.36	52.6	0.0048
Dementia in other diseases classified elsewhere	0.092	0.1	0.0039	0.088	0.071	0.0063	0.073	0.088	0.0052
Unspecified dementia	0.24	0.28	0.0072	0.24	0.27	0.0058	0.22	0.15	0.017

Delirium due to known physiological condition	0.35	0.43	0.013	0.33	0.34	0.002	0.34	0.32	0.0038
Other specified mental disorders due to known physiological condition	0.4	0.49	0.014	0.38	0.4	0.0029	0.36	0.33	0.0062
Alcohol related disorders	3.53	3.42	0.0057	3.47	3.33	0.0078	3	2.77	0.014
Mental and behavioral disorders due to psychoactive substance use	22.3	22.36	0.0016	22.35	22.34	0.00028	20.89	20.81	0.002
Opioid related disorders	2.04	2.17	0.0094	2.02	1.99	0.0021	1.8	1.63	0.013
Cannabis related disorders	1.71	1.81	0.0074	1.67	1.86	0.015	1.69	1.66	0.0023
Cocaine related disorders	1.09	1.14	0.0047	1.04	1.04	0	0.87	0.9	0.0031
Other stimulant related disorders	0.78	0.89	0.011	0.73	0.73	0	0.74	0.72	0.0026
Other psychoactive substance related disorders	1.94	1.91	0.0022	1.82	1.88	0.0044	1.7	1.66	0.0034
Schizophrenia; schizotypal; delusional; and other non-mood psychotic disorders	2.28	2.39	0.0073	2.17	2.2	0.0024	2	2.12	0.0082
Manic episode	0.23	0.26	0.005	0.22	0.22	0.0013	0.2	0.16	0.0086
Mood [affective] disorders	32.09	32	0.0018	31.78	31.86	0.0018	30.29	30.28	0.00016
Bipolar disorder	4.14	4.16	0.00092	4.05	4.05	3.00E-04	3.85	3.94	0.0045
Depressive episode	27.71	27.75	0.00096	27.4	27.47	0.0015	25.97	25.87	0.0023
Major depressive disorder; recurrent	8.48	8.37	0.004	8.37	8.49	0.0045	7.83	7.97	0.0049
Persistent mood [affective] disorders	4.11	4.11	0	3.96	3.94	0.00091	3.36	3.55	0.01
Unspecified mood [affective] disorder	2.87	2.97	0.0058	2.79	2.87	0.0053	2.52	2.56	0.0023
Anxiety; dissociative; stress-related; somatoform and other nonpsychotic mental disorders	32.94	32.49	0.0096	32.82	32.89	0.0015	31.24	31.84	0.013
Eating disorders	1.06	1.11	0.0047	1.11	1.15	0.0033	1.21	1.2	0.00067
Behavioral syndromes associated with physiological disturbances and physical factors	6.69	6.82	0.0054	6.76	7.03	0.011	6.7	6.69	0.00029
Sleep disorders not due to a substance or known physiological condition	4.22	4.21	3.00E-04	4.2	4.31	0.0058	4.05	4.17	0.0059
Disorders of adult personality and behavior	1.63	1.63	0	1.56	1.61	0.0038	1.48	1.45	0.0024
Attention-deficit hyperactivity disorders	2.25	2.21	0.0025	2.18	2.18	4.00E-04	2.17	2.26	0.006
Behavioral and emotional disorders with onset usually occurring in childhood and adolescence	3.12	3.15	0.0018	3	3	0.00035	3.03	3.06	0.0021
Diseases of the nervous system	63.98	64.18	0.0043	63.96	63.64	0.0066	61.63	61.76	0.0027
Encephalitis; myelitis and encephalomyelitis	0.17	0.18	0.0029	0.17	0.17	0	0.17	0.16	0.0018

Encephalitis; myelitis and encephalomyelitis in diseases classified elsewhere	0.061	0.061	0	0.059	0.059	0	0.073	0.073	0
Extrapyramidal and movement disorders	4.74	4.86	0.0054	4.8	4.96	0.0074	4.44	4.74	0.015
Alzheimer disease	0.061	0.061	0	0.059	0.059	0	0.073	0.073	0
Frontotemporal dementia	0.061	0.061	0	0.059	0.059	0	0.073	0.073	0
Senile degeneration of brain; not elsewhere classified	0	0	NA	0.059	0.059	0	0.073	0	0.038
Degeneration of nervous system due to alcohol	0	0.061	0.035	0	0.059	0.034	0	0.073	0.038
Neurocognitive disorder with Lewy bodies	0.061	0.061	0	0.059	0	0.034	0.073	0.073	0
Mild cognitive impairment of uncertain or unknown etiology	0.5	0.5	0	0.5	0.53	0.0041	0.43	0.4	0.0045
Epilepsy and recurrent seizures	2.02	2.13	0.0073	1.97	2.01	0.003	1.96	1.92	0.0032
Migraine	9.82	9.76	0.0019	9.6	9.55	0.0018	9.36	9.63	0.0095
Sleep disorders	38.55	38.95	0.0082	38.74	38.58	0.0033	37.76	37.89	0.0027
Chronic pain; not elsewhere classified	24.34	24.28	0.0016	24.18	24.36	0.0043	22.45	22.73	0.0066
Encephalopathy; unspecified	1.21	1.37	0.014	1.15	1.15	0	1.18	1.14	0.0034
Diseases of the eye and adnexa	32.11	32.37	0.0055	31.59	31.52	0.0014	27.98	28.12	0.0033
CORTICOSTEROIDS FOR SYSTEMIC USE	58.23	58.57	0.0069	58.27	58.62	0.0071	55.23	56.26	0.021
THYROID THERAPY	13.51	13.88	0.011	13.26	13.15	0.0031	13.26	13.36	0.003
Disorders of optic nerve and visual pathways	1.58	1.53	0.0039	1.59	1.66	0.0056	1.56	1.51	0.0042
Diseases of the ear and mastoid process	23.19	23.04	0.0036	22.99	22.9	0.0022	20.27	21.07	0.02
Diseases of the circulatory system	82.1	81.81	0.0076	82.4	82.32	0.0022	80.39	80.65	0.0065
Hypertensive diseases	76.95	76.75	0.0048	77.34	77.2	0.0034	75.16	75.4	0.0056
Hypertensive chronic kidney disease	7.24	7.17	0.0026	6.58	6.57	0.00048	6.22	6.55	0.013
Ischemic heart diseases	17.27	17.15	0.0031	18.04	18.05	0.00015	16.79	17.28	0.013
Other forms of heart disease	26.57	26.79	0.005	26.64	26.61	0.00067	24.71	24.91	0.0046
Cerebrovascular diseases	7.66	7.56	0.0039	7.7	7.74	0.0013	7.04	6.75	0.012
Diseases of arteries; arterioles and capillaries	10.88	10.88	2.00E-04	10.76	10.55	0.0069	10.05	10.28	0.0075
ANTIINFECTIVES FOR SYSTEMIC USE	79.55	79.55	0	79.29	79.42	0.0034	77.23	78.26	0.025
Diseases of the respiratory system	60.28	60.26	5.00E-04	60.03	59.72	0.0063	56.46	57.43	0.02

Diseases of the digestive system	63.8	63.81	0.00013	63.56	63.63	0.0013	60.78	61.54	0.016
Diseases of liver	18.46	18.27	0.0049	18.67	18.98	0.0078	18.02	18.42	0.01
Diseases of the skin and subcutaneous tissue	48.94	48.47	0.0094	48.44	48.08	0.0072	44.67	45.19	0.01
ANTINEOPLASTIC AGENTS	9.81	9.83	0.00062	9.65	9.87	0.0076	8.58	8.98	0.014
Estrogens	3.99	4.08	0.0043	3.57	3.63	0.0035	3.51	3.61	0.0055
Progestogens	3.96	4.07	0.0059	3.75	3.68	0.0041	3.29	3.26	0.0016
IMMUNOSUPPRESSANTS	5.47	5.32	0.0068	5.32	5.43	0.0047	4.93	5.15	0.01
Psoriasis	2.87	2.76	0.007	2.88	2.88	0.00035	2.62	2.7	0.005
Married	30.16	30.27	0.0024	30.66	30.73	0.0015	33.65	33.68	0.00077
Diseases of the musculoskeletal system and connective tissue	73.37	73.21	0.0036	73.02	73.19	0.0039	70.07	70.66	0.013
ANTIINFLAMMATORY AND ANTIRHEUMATIC PRODUCTS	55.13	55.1	0.00049	55.05	55.13	0.0015	51.24	51.33	0.0018
Rheumatoid arthritis with rheumatoid factor	0.48	0.43	0.0082	0.5	0.5	0.00084	0.43	0.49	0.0086
Other rheumatoid arthritis	2.18	2.18	0	2.14	2.11	0.0025	2.13	2.2	0.0045
Systemic lupus erythematosus (SLE)	0.65	0.75	0.012	0.66	0.71	0.0072	0.65	0.69	0.0045
Diseases of the genitourinary system	60.92	61.17	0.005	59.69	60.15	0.0094	57.52	58.16	0.013
OPIOIDS	52.92	53.13	0.0043	52.69	52.73	0.00071	49.7	50.31	0.012
OTHER ANALGESICS AND ANTIPYRETICS	67.33	66.98	0.0074	66.99	67.25	0.0055	63.2	63.5	0.0062
ANTIMIGRAINE PREPARATIONS	9.13	8.98	0.0053	8.74	8.65	0.0031	8.13	8.24	0.0037
ANTIEPILEPTICS	33.32	33.35	0.00078	33.14	33.23	0.0019	30.5	30.85	0.0076
ANTI-PARKINSON DRUGS	4.58	4.57	0.00029	4.71	4.9	0.0088	4.15	4.25	0.0051
ANTIPSYCHOTICS	16.03	16.22	0.0052	15.62	15.88	0.007	14.62	14.35	0.0079
Lithium	0.18	0.15	0.006	0.16	0.21	0.012	0.11	0.12	0.0022
HYPNOTICS AND SEDATIVES	39.83	39.6	0.0046	39.7	39.47	0.0047	37.24	38.13	0.018
Non-selective monoamine reuptake inhibitors	8.26	8.48	0.0079	8.17	8.21	0.0015	6.88	7.1	0.0083
Selective serotonin reuptake inhibitors	24.32	24.14	0.0043	24.21	24.58	0.0088	22.43	22.74	0.0075
Monoamine oxidase inhibitors; non-selective	0.061	0.061	0	0.059	0.059	0	0.073	0.073	0
Other antidepressants	25.57	25.71	0.0032	25.48	25.76	0.0064	23.72	24.15	0.01

PSYCHOSTIMULANTS; AGENTS USED FOR ADHD AND NOOTROPICS	7.15	7	0.006	6.9	7.09	0.0074	6	6.1	0.0043
ANTI-DEMENTIA DRUGS	0.26	0.29	0.0047	0.24	0.27	0.0058	0.19	0.18	0.0017
DRUGS USED IN ADDICTIVE DISORDERS	10.05	10.01	0.0012	10.04	9.99	0.0014	8.62	8.4	0.0081
Acute kidney failure and chronic kidney disease	17.18	17.42	0.0063	16.52	16.28	0.0065	15.41	15.82	0.011
Diseases of male genital organs	15.31	15.21	0.0029	15.55	15.97	0.012	14.22	14.2	0.00063
Noninflammatory disorders of female genital tract	23.78	24.12	0.0079	22.74	22.7	0.0011	22.2	22.54	0.0081
Pregnancy; childbirth and the puerperium	5.6	5.8	0.0087	5.27	5.27	0.00026	5.24	5.37	0.0059
ANTIPARASITIC PRODUCTS; INSECTICIDES AND REPELLENTS	16.66	17.13	0.012	16.31	16.38	0.0019	14.5	14.83	0.0093
ANTIHISTAMINES FOR SYSTEMIC USE	47.04	47.31	0.0054	46.3	46.97	0.013	43.16	43.97	0.016
Somnolence; stupor and coma	4.14	4.33	0.0094	4.04	3.98	0.0033	3.49	3.37	0.0068
Other symptoms and signs involving cognitive functions and awareness	6.6	6.81	0.0086	6.38	6.39	0.00024	5.99	5.89	0.0043
Age-related cognitive decline	0.061	0.061	0	0.059	0.059	0	0.073	0.073	0
Suicidal ideations	1.92	1.91	0.00089	1.83	1.78	0.004	1.71	1.75	0.0034
Nonsuicidal self-harm	0	0.061	0.035	0	0	NA	0	0	NA
Convulsions; not elsewhere classified	2.6	2.75	0.0087	2.54	2.64	0.0059	2.51	2.42	0.0061
Abnormal weight gain	6.15	6.03	0.0049	5.76	5.95	0.0078	6.32	6.04	0.012
Never Married	13.57	13.24	0.0097	13.46	13.54	0.0024	14.63	14.54	0.0025
Intracranial injury	2.01	2.12	0.0073	2.02	2.07	0.0033	1.73	1.61	0.0086
Suicide attempt	0.21	0.22	0.0026	0.19	0.21	0.004	0.2	0.21	0.0032
COVID-19	2.48	2.53	0.0031	2.71	2.58	0.0085	2.58	2.54	0.0028
Post COVID-19 condition	0.061	0.061	0	0.059	0.059	0	0.073	0	0.038
Antidotes	16.96	17.1	0.0036	16.76	17.24	0.013	16.36	16.88	0.014
Widowed	2.67	2.66	0.00076	2.63	2.7	0.004	2.65	2.88	0.014
Intentional self-harm	0.18	0.21	0.0083	0.18	0.2	0.0041	0.17	0.13	0.0094
Contact with and (suspected) exposure to COVID-19	2.93	2.96	0.0014	3.47	3.49	0.00097	3.43	3.49	0.0036
Problems related to education and literacy	0.18	0.18	0	0.18	0.18	0.0014	0.17	0.15	0.0055
Persons with potential health hazards related to socioeconomic and psychosocial circumstances	4.52	4.52	0	4.37	4.42	0.0026	4.03	4	0.0011

Problems related to employment and unemployment	0.57	0.55	0.0025	0.53	0.58	0.0072	0.51	0.56	0.006
Problems related to housing and economic circumstances	1.25	1.26	0.0011	1.24	1.32	0.0073	1.08	0.96	0.012
Problems related to social environment	0.26	0.32	0.012	0.25	0.24	0.0024	0.2	0.22	0.0048
Problems related to upbringing	0.31	0.3	0.0022	0.28	0.26	0.0045	0.32	0.26	0.011
Other problems related to primary support group; including family circumstances	1.24	1.27	0.0033	1.22	1.13	0.0082	1.23	1.24	0.00066
Problems related to certain psychosocial circumstances	0.14	0.13	0.0033	0.15	0.14	0.0016	0.1	0.12	0.0065
Problems related to other psychosocial circumstances	1.17	1.16	0.0011	1.09	1.12	0.0028	0.99	1.02	0.0029
Dietary counseling and surveillance	7.8	7.53	0.01	7.53	7.72	0.0069	8.51	7.62	0.033
Alcohol abuse counseling and surveillance	0.29	0.3	0.0023	0.29	0.29	0.0011	0.34	0.28	0.01
Drug abuse counseling and surveillance	0.061	0.061	0	0.059	0.059	0	0.073	0.073	0
Exercise counseling	0.59	0.58	0.0016	0.57	0.53	0.0048	0.65	0.44	0.029
Problems related to lifestyle	10.11	10.43	0.011	10.39	10.53	0.0046	9.73	9.49	0.0084
Lack of physical exercise	0.092	0.15	0.016	0.1	0.12	0.0054	0.12	0.073	0.014
Inappropriate diet and eating habits	0.25	0.2	0.012	0.41	0.41	0.00092	0.41	0.26	0.025
Problems related to sleep	0.76	0.8	0.0035	0.71	0.75	0.0049	0.69	0.64	0.0072
Long term (current) drug therapy	51.76	51.96	0.0039	52.54	52.84	0.0061	55.48	54.93	0.011
Long term (current) use of insulin	31.21	31.34	0.0028	32.03	32.25	0.0045	33.47	33.6	0.0029
Long term (current) use of aspirin	11.29	11.52	0.0073	11.92	12.13	0.0063	11.83	12.2	0.011
Long term (current) use of oral hypoglycemic drugs	20.22	20	0.0055	20.36	20.04	0.0079	20.81	20.65	0.0038
Family history of mental and behavioral disorders	0.73	0.74	0.0014	0.76	0.78	0.0027	0.77	0.71	0.0068
Personal history of mental and behavioral disorders	1.04	0.93	0.011	1.03	1.04	0.00058	1.07	0.96	0.012
Personal history of psychological trauma; not elsewhere classified	0.28	0.27	0.0023	0.24	0.31	0.012	0.33	0.32	0.0013
Personal history of self-harm	0.42	0.49	0.01	0.42	0.41	0.00092	0.45	0.4	0.0067
Bariatric surgery status	2.4	2.42	0.0016	2.42	2.5	0.0046	2.57	2.28	0.019
Other Race	3.88	3.89	0.00095	3.75	3.91	0.0086	4.15	4.03	0.0059
aspirin	33.82	33.39	0.0091	34.18	34.34	0.0032	29.89	30.31	0.0091
ibuprofen	29.98	30.05	0.0016	29.85	29.7	0.0032	26.6	26.38	0.005

glucagon	15.79	15.61	0.005	16.75	16.62	0.0036	16.09	16.14	0.0016

b) Baseline characteristics after matching, ≥65yo

	Semaglutide	Sitagliptin		Semaglutide	Empagliflozin		Semaglutide	Glipizide	
Number	6227	6227	SMD	7337	7337	SMD	5312	5312	SMD
Age; mean (SD); y	70.75 (4.72)	70.66 (4.85)	0.02	71.07 (4.87)	71.08 (4.93)	0.0023	70.75 (4.73)	70.69 (4.72)	0.013
Sex									
Female	44.31	44.87	0.011	46.53	46.49	0.00082	45.16	45.12	0.00076
Male	45.38	45.11	0.0055	44.05	43.86	0.0038	43.81	43.88	0.0015
Other	10.31	10.02	0.0096	9.42	9.65	0.0079	11.03	10.99	0.0012
Race									
White	65.42	65.6	0.0037	65.73	65.65	0.0017	65.04	64.93	0.0024
Black or African American	11.19	11.66	0.015	11.39	11.18	0.0069	11.54	11.16	0.012
Asian	4.17	4.26	0.004	4.24	4.35	0.0054	4.58	4.58	0
American Indian or Alaska Native	0.19	0.18	0.0037	0.29	0.22	0.014	0.23	0.32	0.018
Native Hawaiian or Other Pacific Islander	0.74	0.67	0.0077	0.65	0.61	0.0052	0.89	1.22	0.033
Unknown	15.35	14.86	0.014	15.14	15.44	0.0083	14.53	14.66	0.0037
Ethnicity									
Hispanic or Latino	4.67	4.79	0.0053	4.51	4.09	0.021	4.8	5.06	0.012
Not Hispanic of Latino	65.2	64.97	0.0047	67.41	67.64	0.0049	70.07	70.07	0
Unknown	30.13	30.24	0.0024	28.08	28.27	0.0042	25.13	24.87	0.0061
Certain infectious and parasitic diseases	40.89	40.82	0.0013	42.69	43.08	0.008	39.91	40.06	0.0031
ANTIOBESITY PREPARATIONS; EXCL. DIET PRODUCTS	1.81	1.61	0.016	1.88	1.78	0.0071	1.9	1.71	0.014
INSULINS AND ANALOGUES	59.19	58.7	0.01	60.19	59.52	0.014	58.43	58.21	0.0046
Biguanides	70.03	70.76	0.016	71.62	72.16	0.012	65.74	66.79	0.022
Thiazolidinediones	9.72	9.57	0.0049	10.84	10.89	0.0018	6.96	7.25	0.011
Dipeptidyl peptidase 4 (DPP-4) inhibitors	NA	NA	NA	29.32	28.7	0.014	23.53	25.19	0.039
Sodium-glucose co-transporter 2 (SGLT2) inhibitors	20.56	21.38	0.02	NA	NA	NA	22.01	21.88	0.0032

Sulfonylureas	35.97	36.95	0.02	41.12	41.07	0.0011	NA	NA	NA
Neoplasms	46.12	45.32	0.016	47.47	47.7	0.0046	44.03	44.58	0.011
ANTIARRHYTHMICS; CLASS I AND III	53.12	53.7	0.012	53.77	53.71	0.0011	51.09	51.45	0.0072
ANTIHYPERTENSIVES	21.09	21.13	0.0012	21.9	21.82	0.002	20.27	20.67	0.0098
DIURETICS	58.2	58.74	0.011	59.91	60.38	0.0095	54.63	53.99	0.013
BETA BLOCKING AGENTS	56.58	57.8	0.025	57.75	58.08	0.0066	54.42	53.84	0.012
CALCIUM CHANNEL BLOCKERS	41.61	41.18	0.0088	43.26	43.68	0.0085	39.1	39.02	0.0015
AGENTS ACTING ON THE RENIN-ANGIOTENSIN SYSTEM	73.63	73.68	0.0011	75.28	75.03	0.0057	70.5	70.01	0.011
LIPID MODIFYING AGENTS	80.81	80.65	0.0041	82.47	82.43	0.0011	78.41	78.37	0.00091
Divorced	5.89	6.05	0.0068	6.11	6.13	0.0011	6.57	6.55	0.00076
Diseases of the blood and blood-forming organs and certain disorders involving the immune mechanism	40.29	39.91	0.0079	42.36	42.4	0.00083	38.99	38.69	0.0062
Certain disorders involving the immune mechanism	4.09	3.84	0.013	4.43	4.27	0.008	3.95	3.56	0.021
Disorders of thyroid gland	30.56	30.72	0.0035	30.97	30.83	0.0029	31.76	32.21	0.0097
Endocrine; nutritional and metabolic diseases	100	100	NA	100	100	NA	100	100	NA
Type 1 diabetes mellitus	13.7	13.46	0.007	13.96	14.39	0.013	12.65	12.09	0.017
Vitamin D deficiency	28.6	29.71	0.024	30.61	31.05	0.0094	31.29	31.46	0.0037
Overweight; obesity and other hyperalimentation	56.5	57.36	0.018	56.18	56.88	0.014	55.61	55.65	0.00076
Metabolic disorders	92.93	93.08	0.0057	93.43	93.61	0.0072	92.3	92.7	0.015
Disorders of lipoprotein metabolism and other lipidemias	90.3	90.61	0.01	90.94	91.32	0.013	89.91	90.34	0.015
Other disorders of fluid; electrolyte and acid-base balance	25.57	26.21	0.015	27.04	27.82	0.017	24.11	24.45	0.0079
Metabolic syndrome and other insulin resistance	3.85	3.52	0.018	3.82	3.84	0.0014	3.54	3.16	0.021
Vascular dementia	0.53	0.63	0.013	0.68	0.64	0.005	0.6	0.7	0.012
Mental; Behavioral and Neurodevelopmental disorders	48.1	48.72	0.013	48.63	49.05	0.0085	46.8	46.69	0.0023
Dementia in other diseases classified elsewhere	0.82	0.76	0.0073	1.01	1.02	0.0014	1	1.24	0.023
Unspecified dementia	1.72	1.56	0.013	1.81	1.9	0.0061	1.71	1.94	0.017
Delirium due to known physiological condition	0.71	0.77	0.0075	0.86	0.94	0.0087	0.77	0.73	0.0044
Other specified mental disorders due to known physiological condition	0.8	1.01	0.022	0.82	0.95	0.015	0.62	0.71	0.012

Alcohol related disorders	2.57	2.84	0.017	2.55	2.59	0.0026	2.13	2.32	0.013
Mental and behavioral disorders due to psychoactive substance use	14.6	14.69	0.0027	14.39	14.39	0	13.74	13.05	0.02
Opioid related disorders	1.25	1.3	0.0043	1.27	1.31	0.0036	1.28	1.32	0.0033
Cannabis related disorders	0.55	0.66	0.015	0.53	0.61	0.011	0.45	0.49	0.0055
Cocaine related disorders	0.34	0.4	0.011	0.29	0.26	0.0052	0.26	0.26	0
Other stimulant related disorders	0.16	0.16	0	0.15	0.14	0.0036	0.19	0.19	0
Other psychoactive substance related disorders	0.82	0.79	0.0036	0.76	0.63	0.016	0.87	0.71	0.017
Schizophrenia; schizotypal; delusional; and other non-mood psychotic disorders	1.57	1.65	0.0064	1.57	1.55	0.0011	1.28	1.32	0.0033
Manic episode	0.16	0.16	0	0.2	0.19	0.0031	0.19	0.19	0
Mood [affective] disorders	28.26	28.71	0.01	28.68	28.98	0.0066	27.73	27.37	0.008
Bipolar disorder	1.65	1.81	0.012	1.75	1.83	0.0062	1.83	1.88	0.0042
Depressive episode	24.46	25.16	0.016	25.23	25.42	0.0044	24.49	24.17	0.0075
Major depressive disorder; recurrent	7.39	7.72	0.013	7.54	7.81	0.01	6.59	6.7	0.0045
Persistent mood [affective] disorders	3.9	3.89	0.00083	4.09	3.91	0.009	3.29	3.77	0.026
Unspecified mood [affective] disorder	2.46	2.47	0.001	2.28	2.34	0.0045	2	2.24	0.017
Anxiety; dissociative; stress-related; somatoform and other nonpsychotic mental disorders	26.9	27.56	0.015	27.39	27.41	0.00031	25.89	27.09	0.027
Eating disorders	0.59	0.53	0.0086	0.57	0.55	0.0037	0.66	0.71	0.0068
Behavioral syndromes associated with physiological disturbances and physical factors	6.36	6.67	0.012	6.56	6.76	0.0082	6.5	6.25	0.01
Sleep disorders not due to a substance or known physiological condition	4.69	5.11	0.019	4.72	4.77	0.0026	4.72	4.52	0.0099
Disorders of adult personality and behavior	0.95	0.96	0.0017	1.01	0.98	0.0027	0.73	0.77	0.0044
Attention-deficit hyperactivity disorders	0.74	0.87	0.014	0.63	0.65	0.0034	0.66	0.87	0.024
Behavioral and emotional disorders with onset usually occurring in childhood and adolescence	1.08	1.19	0.011	0.99	1.02	0.0027	1.03	1.22	0.018
Diseases of the nervous system	67.87	68.93	0.023	68.3	69.03	0.016	66.47	66.51	8.00E-04
Encephalitis; myelitis and encephalomyelitis	0.16	0.16	0	0.2	0.18	0.0062	0.19	0.19	0
Encephalitis; myelitis and encephalomyelitis in diseases classified elsewhere	0	0	NA	0	0	NA	0	0	NA
Extrapyramidal and movement disorders	8.48	8.83	0.013	8.63	9.02	0.014	8.06	7.79	0.0098

Alzheimer disease	0.5	0.42	0.012	0.67	0.65	0.0017	0.62	0.62	0
Frontotemporal dementia	0.16	0	0.057	0.14	0.14	0	0.19	0.19	0
Senile degeneration of brain; not elsewhere classified	0	0.16	0.057	0.14	0.14	0	0.19	0.19	0
Degeneration of nervous system due to alcohol	0	0	NA	0	0	NA	0	0.19	0.061
Neurocognitive disorder with Lewy bodies	0.16	0.16	0	0.14	0.14	0	0.19	0.19	0
Mild cognitive impairment of uncertain or unknown etiology	1.72	1.78	0.0049	1.83	1.75	0.0062	1.9	2.05	0.011
Epilepsy and recurrent seizures	1.51	1.67	0.013	1.57	1.66	0.0076	1.51	1.35	0.013
Migraine	4.95	5.03	0.0037	4.93	5.1	0.0075	4.86	5.16	0.014
Sleep disorders	40.81	41.03	0.0046	40.73	41.53	0.016	39.72	39.57	0.0031
Chronic pain; not elsewhere classified	28.12	28.73	0.014	28.2	27.9	0.0067	26.66	26.47	0.0043
Encephalopathy; unspecified	1.78	2.02	0.018	2.02	2	0.00097	1.96	1.79	0.012
Diseases of the eye and adnexa	40.66	40.42	0.0049	42.29	42.48	0.0039	38.35	39.85	0.031
CORTICOSTEROIDS FOR SYSTEMIC USE	60.72	61.19	0.0095	62.67	63.13	0.0096	60.41	60.18	0.0046
THYROID THERAPY	19.01	19.01	0	19.6	19.79	0.0048	19.62	19.13	0.012
Disorders of optic nerve and visual pathways	1.83	1.91	0.0059	2.17	2.21	0.0028	2.33	2.52	0.012
Diseases of the ear and mastoid process	28.62	28.47	0.0032	30.43	30.75	0.0068	27.99	28.12	0.0029
Diseases of the circulatory system	94.96	94.96	0	94.98	95.3	0.015	94.14	93.86	0.012
Hypertensive diseases	91.67	91.7	0.0012	91.8	91.92	0.0045	90.47	90.19	0.0096
Hypertensive chronic kidney disease	15.46	16.65	0.032	17.7	17.38	0.0086	15.42	15.3	0.0031
Ischemic heart diseases	39.68	39.42	0.0053	39.35	39.05	0.0061	38.78	39.83	0.022
Other forms of heart disease	47.44	48.02	0.012	47.96	48.38	0.0085	45.71	45.78	0.0015
Cerebrovascular diseases	18.16	18.18	0.00042	18.73	18.74	0.00035	18.32	17.7	0.016
Diseases of arteries; arterioles and capillaries	25.53	25.86	0.0074	26.39	26.84	0.01	25.23	24.68	0.013
ANTIINFECTIVES FOR SYSTEMIC USE	79.94	80.63	0.017	81.71	82.12	0.011	79.16	79.18	0.00046
Diseases of the respiratory system	62.15	62.42	0.0056	62.79	62.87	0.0017	60.32	60	0.0065
Diseases of the digestive system	69.09	69.47	0.0084	70.94	71.54	0.013	68.47	69.03	0.012
Diseases of liver	16.77	16.81	0.0013	17.53	17.95	0.011	17.06	16.81	0.0065

Diseases of the skin and subcutaneous tissue	50.94	51.34	0.008	52.6	53.07	0.0096	48.59	48.29	0.006
ANTINEOPLASTIC AGENTS	14.02	13.88	0.0042	14.8	14.81	0.00038	13.57	13.82	0.0071
Estrogens	0.16	0.16	0	0.14	0.14	0	0.19	0.19	0
Progestogens	1.01	0.95	0.0065	1.12	0.95	0.016	0.77	0.83	0.0063
IMMUNOSUPPRESSANTS	5.54	5.83	0.012	6.39	6.02	0.015	6.08	6.23	0.0063
Psoriasis	3.21	3.55	0.019	3.26	3.08	0.01	3.03	3.01	0.0011
Married	31.03	31.04	0.00035	31.17	31.62	0.0097	34.64	34.58	0.0012
Diseases of the musculoskeletal system and connective tissue	80.54	80.25	0.0073	81.94	82.28	0.0089	79.48	79.37	0.0028
ANTIINFLAMMATORY AND ANTIRHEUMATIC PRODUCTS	49.56	49.48	0.0016	50.84	51.38	0.011	46.82	47.33	0.01
Rheumatoid arthritis with rheumatoid factor	0.93	1.03	0.0098	0.93	0.98	0.0056	0.96	1.02	0.0057
Other rheumatoid arthritis	3.45	3.55	0.0052	3.37	3.42	0.003	3.6	3.73	0.007
Systemic lupus erythematosus (SLE)	0.5	0.59	0.013	0.59	0.61	0.0035	0.43	0.43	0
Diseases of the genitourinary system	70.98	71.11	0.0028	73.35	73.52	0.0037	69.86	69.67	0.0041
OPIOIDS	58.3	58.42	0.0026	59.94	59.32	0.013	56.36	56.78	0.0084
OTHER ANALGESICS AND ANTIPYRETICS	73.65	73.04	0.014	75.18	75.26	0.0019	71.2	71.31	0.0025
ANTIMIGRAINE PREPARATIONS	6.44	6.17	0.011	6.76	6.98	0.0086	5.87	5.87	0
ANTIEPILEPTICS	36.9	37.1	0.004	37.48	38	0.011	35.47	36.35	0.018
ANTI-PARKINSON DRUGS	6.1	5.89	0.0088	6.2	6.61	0.017	5.5	5.25	0.011
ANTIPSYCHOTICS	14.16	14.55	0.011	14.39	14.39	0	12.61	12.59	0.00057
Lithium	0	0.16	0.057	0	0.14	0.052	0.19	0.19	0
HYPNOTICS AND SEDATIVES	46.8	47.26	0.0093	48.04	47.62	0.0085	45.2	45.35	0.003
Non-selective monoamine reuptake inhibitors	7.19	7.18	0.00062	7.48	7.39	0.0036	6.25	6.06	0.0078
Selective serotonin reuptake inhibitors	21.81	22.63	0.02	22.58	22.77	0.0046	20.46	20.67	0.0051
Monoamine oxidase inhibitors; non-selective	0.16	0.16	0	0.14	0.14	0	0.19	0.19	0
Other antidepressants	24.06	24.96	0.021	24.67	25.04	0.0085	23.57	23.63	0.0013
PSYCHOSTIMULANTS; AGENTS USED FOR ADHD AND NOOTROPICS	5.27	5.54	0.012	5.42	5.46	0.0018	4.16	4.52	0.018
ANTI-DEMENTIA DRUGS	1.69	1.88	0.015	1.76	1.77	0.001	1.71	2	0.021

DRUGS USED IN ADDICTIVE DISORDERS	5.54	5.62	0.0035	5.56	5.66	0.0041	5.1	4.69	0.019
Acute kidney failure and chronic kidney disease	35.02	34.95	0.0017	38.45	38.18	0.0056	34.43	33.43	0.021
Diseases of male genital organs	24.75	24.01	0.017	24.97	25.42	0.01	24.06	24.06	0
Noninflammatory disorders of female genital tract	14.58	14.73	0.0041	15.87	15.8	0.0019	14.36	14.83	0.013
Pregnancy; childbirth and the puerperium	0.88	0.87	0.0017	0.83	0.89	0.0059	0.71	0.64	0.0092
ANTIPARASITIC PRODUCTS; INSECTICIDES AND REPELLENTS	13.23	13.57	0.0099	14.28	14.28	0	12.56	12.74	0.0057
ANTIHISTAMINES FOR SYSTEMIC USE	47.58	47.42	0.0032	49.16	49.39	0.0046	45.93	46.78	0.017
Somnolence; stupor and coma	4.5	4.35	0.007	4.73	4.81	0.0038	3.92	3.97	0.0029
Other symptoms and signs involving cognitive functions and awareness	11.55	11.55	0	11.88	12.47	0.018	11.18	11.28	0.003
Age-related cognitive decline	0.18	0.21	0.0073	0.23	0.23	0	0.21	0.23	0.0041
Suicidal ideations	0.63	0.74	0.014	0.67	0.67	0	0.53	0.47	0.008
Nonsuicidal self-harm	0	0	NA	0	0	NA	0	0	NA
Convulsions; not elsewhere classified	2.2	2.31	0.0076	2.34	2.4	0.0036	2.41	2.22	0.013
Abnormal weight gain	4.08	3.92	0.0082	4.12	4.02	0.0048	4.39	4.24	0.0074
Never Married	5.91	6.04	0.0054	5.64	5.44	0.0089	6.06	5.78	0.012
Intracranial injury	2.63	2.3	0.022	2.44	2.54	0.0061	2.54	2.3	0.016
Suicide attempt	0.16	0.16	0	0.14	0.14	0	0.19	0.19	0
COVID-19	1.93	1.99	0.0046	2	1.94	0.0049	2.07	2.15	0.0052
Post COVID-19 condition	0	0.16	0.057	0	0	NA	0	0	NA
Antidotes	20.3	20.33	8.00E-04	20.39	20.05	0.0085	19.62	19.9	0.0071
Widowed	8.88	9.38	0.017	9.36	8.94	0.015	9.83	9.45	0.013
Intentional self-harm	0.16	0.16	0	0.14	0.14	0	0.19	0.19	0
Contact with and (suspected) exposure to COVID-19	3.15	3.23	0.0046	3.07	2.96	0.0064	3.07	2.99	0.0044
Problems related to education and literacy	0.16	0.16	0	0.16	0.15	0.0034	0.19	0.19	0
Persons with potential health hazards related to socioeconomic and psychosocial circumstances	3.53	3.71	0.0095	3.58	3.44	0.0081	3.22	3.31	0.0053
Problems related to employment and unemployment	0.19	0.23	0.007	0.15	0.15	0	0.19	0.19	0
Problems related to housing and economic circumstances	0.8	0.83	0.0036	0.79	0.65	0.016	0.58	0.51	0.01

Problems related to social environment	0.34	0.29	0.0086	0.31	0.29	0.005	0.3	0.28	0.0035
Problems related to upbringing	0.16	0.16	0	0.14	0.2	0.017	0.19	0.19	0
Other problems related to primary support group; including family circumstances	1.12	1.19	0.006	1.25	1.34	0.0072	1.32	1.54	0.019
Problems related to certain psychosocial circumstances	0	0	NA	0	0.14	0.052	0	0.19	0.061
Problems related to other psychosocial circumstances	1.06	1.04	0.0016	1.05	0.93	0.012	0.75	0.77	0.0022
Dietary counseling and surveillance	7.05	6.79	0.01	6.67	6.3	0.015	7.34	6.48	0.034
Alcohol abuse counseling and surveillance	0.27	0.24	0.0063	0.27	0.26	0.0026	0.24	0.3	0.011
Drug abuse counseling and surveillance	0.16	0.16	0	0.14	0.14	0	0.19	0	0.061
Exercise counseling	0.56	0.58	0.0021	0.61	0.55	0.009	0.7	0.68	0.0023
Problems related to lifestyle	7.08	7.16	0.0031	6.84	6.72	0.0049	6.76	6.66	0.0038
Lack of physical exercise	0.16	0.16	0	0.14	0.15	0.0036	0.19	0.19	0
Inappropriate diet and eating habits	0.29	0.26	0.0062	0.26	0.16	0.021	0.45	0.28	0.028
Problems related to sleep	0.71	0.69	0.0019	0.68	0.61	0.0085	0.66	0.7	0.0046
Long term (current) drug therapy	62.81	62.53	0.0056	60.68	60.35	0.0067	65.44	64.95	0.01
Long term (current) use of insulin	41.34	41.13	0.0042	39.84	39.53	0.0064	44.26	44.11	0.003
Long term (current) use of aspirin	20.78	21.41	0.015	21.15	21.07	0.002	22.48	22.59	0.0027
Long term (current) use of oral hypoglycemic drugs	21.73	22.26	0.013	22.15	23.07	0.022	21.57	20.71	0.021
Family history of mental and behavioral disorders	0.55	0.51	0.0044	0.53	0.49	0.0057	0.53	0.55	0.0026
Personal history of mental and behavioral disorders	0.63	0.58	0.0062	0.68	0.71	0.0033	0.6	0.75	0.018
Personal history of psychological trauma; not elsewhere classified	0.16	0.16	0	0.14	0.14	0	0.19	0.19	0
Personal history of self-harm	0.23	0.18	0.011	0.22	0.2	0.003	0.21	0.26	0.012
Bariatric surgery status	1.83	1.83	0	1.64	1.58	0.0043	2.17	1.92	0.017
Other Race	2.92	2.78	0.0087	2.55	2.55	0	3.2	3.12	0.0043
aspirin	49.77	48.96	0.016	50.98	50.47	0.01	46.07	46.86	0.016
ibuprofen	21.91	21.95	0.0012	22.42	22.02	0.0095	22.06	21.4	0.016
glucagon	18.61	18.55	0.0017	19.25	19.26	0.00035	16.27	16.27	0

c) Study results, <65yo; ≥65yo

	Outcome	<65yo		≥65yo	
**Semaglutide *vs***		*HR*	*p-value*	*HR*	*p-value*
**Empagliflozin (SGLT2I)**	Encephalitis	0.49	0.55035122	2.02	0.40247421
	Parkinsonism	1.85	0.18007288	0.91	0.7531736
	Cognitive deficit	0.92	0.23614961	1.00	0.99723872
	Dementia	0.71	0.30575987	1.00	0.97548765
	Epilepsy/seizure	0.79	0.15366797	0.99	0.97612202
	Migraine	0.91	0.3439359	0.82	0.32981421
	Insomnia	0.97	0.62903513	0.96	0.70167761
	Nerve disorder	0.96	0.56286744	1.09	0.47197542
	MNJ/muscle disease	0.94	0.79994515	0.67	0.13510314
	ICH	0.86	0.57260049	0.81	0.38792077
	Ischaemic stroke	0.89	0.37313925	1.01	0.93995414
	Alcohol misuse	0.84	0.28610377	1.29	0.38748254
	Opioid misuse	0.88	0.49791136	0.99	0.97949567
	Cannabis misuse	1.03	0.88074681	1.80	0.28006268
	Stimulants misuse	0.60	0.05628303	1.25	0.74153631
	Nicotine misuse	0.82	0.021832	0.70	0.02274894
	Psychosis	0.77	0.30268143	0.74	0.38352186
	Bipolar disorder	0.90	0.49778956	1.81	0.27748457
	Depression	0.95	0.43384809	0.90	0.3015503
	Anxiety disorder	1.07	0.22379488	0.83	0.04030693
	OCD	0.68	0.27802155	0.80	0.73716877
	Suicidality	0.89	0.58878866	0.92	0.82937466
	NCOs	1.05	0.34869384	0.95	0.49943939
**Sitagliptin (DPP4I)**	Encephalitis	0.19	0.08042827	0.59	0.45904967
	Parkinsonism	1.68	0.26435623	0.64	0.14239535
	Cognitive deficit	0.79	0.00095438	0.67	6.76E-06
	Dementia	0.40	0.00269568	0.60	0.0009199
	Epilepsy/seizure	0.60	0.00079076	0.88	0.53133574
	Migraine	0.93	0.4681725	1.87	0.01474388
	Insomnia	0.88	0.07205165	0.98	0.87219375
	Nerve disorder	1.02	0.82421179	1.06	0.62303269
	MNJ/muscle disease	0.81	0.33290485	1.12	0.70186007
	ICH	0.79	0.37825646	0.79	0.3331381
	Ischaemic stroke	0.77	0.04442967	0.84	0.2033747
	Alcohol misuse	0.84	0.30286474	0.90	0.70445398
	Opioid misuse	0.66	0.01525717	1.27	0.41439674
	Cannabis misuse	0.66	0.01487948	2.63	0.1296305
	Stimulants misuse	0.43	0.00085663	4.11	0.1563485

	Nicotine misuse	0.76	0.00162165	0.62	0.00586291
	Psychosis	0.60	0.0347041	0.49	0.03689575
	Bipolar disorder	0.70	0.02282138	0.73	0.47615041
	Depression	0.82	0.00165103	0.75	0.00562308
	Anxiety disorder	0.92	0.1070765	0.87	0.14377647
	OCD	0.98	0.96205423	0.49	0.17524118
	Suicidality	0.71	0.09920642	0.90	0.79108218
	NCOs	1.09	0.1151691	1.06	0.53024683
**Glipizide (SU)**	Encephalitis	0.00	0.00014716	1.48	0.66621151
	Parkinsonism	0.69	0.37207163	0.98	0.95252812
	Cognitive deficit	0.76	0.00083725	0.65	4.69E-06
	Dementia	0.51	0.0634984	0.68	0.02983814
	Epilepsy/seizure	0.77	0.11743783	0.85	0.44536662
	Migraine	0.82	0.07172271	1.26	0.36382492
	Insomnia	1.10	0.2176249	0.87	0.27443512
	Nerve disorder	0.89	0.14615763	1.04	0.78940994
	MNJ/muscle disease	0.78	0.32951492	1.03	0.91894143
	ICH	0.93	0.81265575	0.76	0.31310738
	Ischaemic stroke	1.16	0.32606178	0.83	0.21437959
	Alcohol misuse	0.56	0.00036413	0.54	0.05065349
	Opioid misuse	0.60	0.00856466	1.73	0.10544634
	Cannabis misuse	0.54	0.00127138	0.57	0.2264673
	Stimulants misuse	0.49	0.00987973	2.02	0.40461993
	Nicotine misuse	0.72	0.000389	0.60	0.00322003
	Psychosis	0.46	0.00271212	0.65	0.24442584
	Bipolar disorder	0.79	0.15925522	0.88	0.78266906
	Depression	0.80	0.00103516	0.79	0.03484866
	Anxiety disorder	0.97	0.66662952	0.88	0.21292071
	OCD	1.11	0.77137136	0.36	0.11153352
	Suicidality	0.49	0.00161508	0.73	0.47924912
	NCOs	1.03	0.57363325	0.92	0.38253829

Supplementary Table S6. Secondary analysis, death as composite outcome, study results

	Outcome (+ death)		
**Semaglutide** ***vs***		*HR*	*p-value*
**Empagliflozin (SGLT2I)**	Encephalitis	0.91	0.18697364
	Parkinsonism	0.90	0.13463082
	Cognitive deficit	1.01	0.76709351
	Dementia	0.91	0.13833183
	Epilepsy/seizure	0.91	0.18494713
	Migraine	0.91	0.11496881
	Insomnia	0.99	0.79053747
	Nerve disorder	0.97	0.5008048
	MNJ/muscle disease	0.88	0.06623094
	ICH	0.91	0.19161458
	Ischaemic stroke	0.99	0.83601457
	Alcohol misuse	0.90	0.13305984
	Opioid misuse	0.93	0.26704373
	Cannabis misuse	0.94	0.36557049
	Stimulants misuse	0.89	0.09585377
	Nicotine misuse	0.87	0.0136942
	Psychosis	0.91	0.16695949
	Bipolar disorder	0.93	0.26152281
	Depression	0.92	0.09455663
	Anxiety disorder	0.95	0.18879364
	OCD	0.89	0.10226758
	Suicidality	0.91	0.16417062
**Sitagliptin (DPP4I)**	Encephalitis	0.63	6.08E-11
	Parkinsonism	0.65	5.31E-10
	Cognitive deficit	0.75	1.64E-09
	Dementia	0.62	7.27E-13
	Epilepsy/seizure	0.65	1.22E-11
	Migraine	0.72	1.64E-08
	Insomnia	0.80	4.98E-06
	Nerve disorder	0.80	9.53E-06
	MNJ/muscle disease	0.66	1.59E-09
	ICH	0.64	1.53E-10
	Ischaemic stroke	0.71	1.10E-08
	Alcohol misuse	0.66	3.22E-10
	Opioid misuse	0.67	1.77E-09
	Cannabis misuse	0.65	8.42E-11
	Stimulants misuse	0.62	2.74E-12
	Nicotine misuse	0.68	2.93E-12

	Psychosis	0.64	3.76E-11
	Bipolar disorder	0.67	2.19E-09
	Depression	0.72	3.53E-13
	Anxiety disorder	0.83	2.64E-06
	OCD	0.64	3.22E-10
	Suicidality	0.64	1.48E-10
**Glipizide (SU)**	Encephalitis	0.54	9.35E-16
	Parkinsonism	0.56	8.53E-15
	Cognitive deficit	0.67	1.85E-15
	Dementia	0.56	9.93E-16
	Epilepsy/seizure	0.61	3.50E-12
	Migraine	0.66	2.36E-11
	Insomnia	0.83	0.0003349
	Nerve disorder	0.74	8.95E-09
	MNJ/muscle disease	0.60	2.72E-12
	ICH	0.57	5.98E-14
	Ischaemic stroke	0.69	2.27E-08
	Alcohol misuse	0.55	9.48E-18
	Opioid misuse	0.58	1.10E-14
	Cannabis misuse	0.54	1.19E-17
	Stimulants misuse	0.54	8.72E-17
	Nicotine misuse	0.61	4.79E-17
	Psychosis	0.52	3.65E-19
	Bipolar disorder	0.58	4.50E-15
	Depression	0.71	7.47E-12
	Anxiety disorder	0.83	3.72E-05
	OCD	0.54	4.75E-16
	Suicidality	0.52	5.31E-19

Supplementary Table S7. Secondary analysis, between 1st December 2017 and 31st December 2018; between 1st January 2019 and 31st December 2019; between 1st January 2020 and 31st May 2021

a) Baseline characteristics after matching, between 1st December 2017 and 31st December 2018

	**Semaglutide**	**Sitagliptin**		**Semaglutide**	**Empagliflozin**		**Semaglutide**	**Glipizide**	
Number	1527	1527	*SMD*	1511	1511	*SMD*	1261	1261	*SMD*
Age; mean (SD); y	56.15 (12.43)	55.79 (14.09)	0.027	56.32 (12.35)	56.36 (12.00)	0.0035	55.22 (12.41)	54.89 (13.54)	0.026
Sex									
Female	48.79	49.12	0.0066	48.38	48.58	0.004	48.45	46.71	0.035
Male	39.82	39.42	0.008	40.11	39.64	0.0095	38.3	40.21	0.039
Other	11.39	11.46	0.0021	11.52	11.78	0.0083	13.24	13.09	0.0047
Race									
White	59.33	60.12	0.016	59.5	59.1	0.0081	59.95	59.4	0.011
Black or African American	13.56	13.69	0.0038	13.3	13.04	0.0078	12.13	12.13	0
Asian	2.88	2.75	0.0079	2.91	3.38	0.027	2.78	2.22	0.036
American Indian or Alaska Native	0.66	0.66	0	0.66	0.66	0	0.79	0.79	0
Native Hawaiian or Other Pacific Islander	0.66	0.66	0	0.66	0.66	0	0.79	1.43	0.061
Unknown	19.91	18.99	0.023	19.99	20.45	0.012	20.78	21.33	0.014
Ethnicity									
Hispanic or Latino	7.99	7.27	0.027	8.21	7.61	0.022	8.25	6.5	0.067
Not Hispanic of Latino	61.17	61.69	0.011	60.89	61.81	0.019	60.35	61.78	0.029
Unknown	30.84	31.04	0.0043	30.91	30.58	0.0072	31.4	31.72	0.0068
Certain infectious and parasitic diseases	40.86	39.16	0.035	40.57	39.97	0.012	38.38	38.94	0.011
ANTIOBESITY PREPARATIONS; EXCL. DIET PRODUCTS	3.86	4.19	0.017	3.77	3.18	0.033	4.36	4.04	0.016
INSULINS AND ANALOGUES	53.31	54.16	0.017	53.08	52.42	0.013	49.41	49.48	0.0016
Biguanides	75.97	75.44	0.012	75.71	76.97	0.03	71.53	71.61	0.0018

Thiazolidinediones	8.38	8.38	0	8.4	8.8	0.014	5.95	5.95	0
Dipeptidyl peptidase 4 (DPP-4) inhibitors	NA	NA	NA	23.76	24.49	0.017	17.92	20.06	0.055
Sodium-glucose co-transporter 2 (SGLT2) inhibitors	0	0	NA	NA	NA	NA	19.35	19.83	0.012
Sulfonylureas	33.4	35.89	0.052	33.49	33.29	0.0042	NA	NA	NA
Neoplasms	38.24	38.83	0.012	37.99	39.58	0.033	34.18	32.75	0.03
ANTIARRHYTHMICS; CLASS I AND III	45.58	45.91	0.0066	45.27	43.15	0.043	41.71	41.16	0.011
ANTIHYPERTENSIVES	16.18	16.18	0	15.69	15.55	0.0036	14.67	14.12	0.016
DIURETICS	46.56	46.3	0.0053	46.26	47.12	0.017	41.55	40.6	0.019
BETA BLOCKING AGENTS	44.14	46.17	0.041	44.01	42.95	0.021	40.13	38.62	0.031
CALCIUM CHANNEL BLOCKERS	29.73	30.06	0.0072	29.32	29.25	0.0015	25.77	24.58	0.027
AGENTS ACTING ON THE RENIN-ANGIOTENSIN SYSTEM	64.51	63.65	0.018	64.26	63.14	0.023	59.48	60.03	0.011
LIPID MODIFYING AGENTS	69.88	69.88	0	70.09	69.89	0.0043	65.03	67.01	0.042
Divorced	5.57	5.63	0.0028	5.62	5.82	0.0085	5.87	6.42	0.023
Diseases of the blood and blood-forming organs and certain disorders involving the immune mechanism	34.12	34.51	0.0083	33.62	33.62	0	31.4	32.12	0.015
Certain disorders involving the immune mechanism	3.21	4.13	0.049	3.18	3.84	0.036	3.17	3.41	0.013
Disorders of thyroid gland	27.57	28.36	0.018	27.4	28.46	0.024	27.2	25.93	0.029
Endocrine; nutritional and metabolic diseases	100	100	NA	100	100	NA	100	100	NA
Type 1 diabetes mellitus	14.21	14.34	0.0037	14.16	12.57	0.047	12.69	13.88	0.035
Vitamin D deficiency	31.37	30.98	0.0085	31.04	31.44	0.0086	29.5	29.74	0.0052
Overweight; obesity and other hyperalimentation	62.61	62.8	0.0041	62.41	60.36	0.042	63.05	62.09	0.02
Metabolic disorders	85.53	85.99	0.013	85.37	85.11	0.0075	83.82	84.46	0.017
Disorders of lipoprotein metabolism and other lipidemias	82.25	81.79	0.012	82.2	81.67	0.014	79.86	81.28	0.036
Other disorders of fluid; electrolyte and acid-base balance	17.68	17.68	0	17.41	16.94	0.012	16.65	17.13	0.013
Metabolic syndrome and other insulin resistance	4.52	4.39	0.0063	4.57	3.77	0.04	4.84	4.52	0.015
Vascular dementia	0.66	0.66	0	0.66	0	0.12	0	0.79	0.13
Mental; Behavioral and Neurodevelopmental disorders	51.73	51.34	0.0079	51.29	49.37	0.038	49.48	49.01	0.0095
Dementia in other diseases classified elsewhere	0.66	0.66	0	0.66	0.66	0	0	0	NA

Unspecified dementia	0.66	0.66	0	0.66	0.66	0	0.79	0.79	0
Delirium due to known physiological condition	0.66	0.66	0	0.66	0.66	0	0.79	0.79	0
Other specified mental disorders due to known physiological condition	0.66	0.79	0.015	0.66	0.66	0	0.79	0.79	0
Alcohol related disorders	2.23	2.42	0.013	2.25	2.18	0.0045	1.9	1.67	0.018
Mental and behavioral disorders due to psychoactive substance use	18.07	17.29	0.021	18	17.21	0.021	15.78	16.81	0.028
Opioid related disorders	1.64	1.24	0.033	1.66	1.39	0.022	1.51	1.59	0.0064
Cannabis related disorders	0.79	0.85	0.0073	0.73	0.86	0.015	0.79	1.11	0.033
Cocaine related disorders	0.66	0.66	0	0.66	0.79	0.016	0.79	1.11	0.033
Other stimulant related disorders	0.66	0.66	0	0.66	0.66	0	0.79	0.79	0
Other psychoactive substance related disorders	1.24	1.11	0.012	1.19	1.39	0.018	1.43	1.51	0.0066
Schizophrenia; schizotypal; delusional; and other non-mood psychotic disorders	2.03	2.16	0.0091	1.99	1.85	0.0096	1.98	1.98	0
Manic episode	0.66	0.66	0	0.66	0.66	0	0.79	0.79	0
Mood [affective] disorders	29.73	29.14	0.013	29.39	27.53	0.041	29.18	29.5	0.007
Bipolar disorder	2.36	2.55	0.013	2.32	2.05	0.018	2.46	3.09	0.039
Depressive episode	25.61	25.28	0.0075	25.28	24.22	0.025	25.22	24.82	0.0092
Major depressive disorder; recurrent	7.99	8.05	0.0024	7.74	7.94	0.0074	7.53	7.38	0.006
Persistent mood [affective] disorders	5.11	5.11	0	4.9	4.17	0.035	5	6.19	0.052
Unspecified mood [affective] disorder	2.42	2.36	0.0043	2.38	2.05	0.022	2.54	2.54	0
Anxiety; dissociative; stress-related; somatoform and other nonpsychotic mental disorders	29.4	30.25	0.019	29.12	27.13	0.044	29.58	28.23	0.03
Eating disorders	1.64	1.64	0	1.19	1.26	0.006	1.75	1.82	0.006
Behavioral syndromes associated with physiological disturbances and physical factors	7.53	7.2	0.013	7.08	7.01	0.0026	7.53	8.17	0.024
Sleep disorders not due to a substance or known physiological condition	4.26	4.13	0.0065	4.24	4.04	0.01	4.52	5.39	0.04
Disorders of adult personality and behavior	1.24	1.51	0.022	1.19	1.46	0.023	1.19	1.9	0.058
Attention-deficit hyperactivity disorders	1.7	1.7	0	1.66	1.52	0.011	1.9	2.85	0.062
Behavioral and emotional disorders with onset usually occurring in childhood and adolescence	2.1	2.23	0.009	2.05	1.99	0.0047	2.38	3.09	0.044
Diseases of the nervous system	66.14	65.29	0.018	65.92	65.78	0.0028	63.92	62.81	0.023

Encephalitis; myelitis and encephalomyelitis	0.66	0.66	0	0.66	0.66	0	0.79	0.79	0
Encephalitis; myelitis and encephalomyelitis in diseases classified elsewhere	0	0	NA	0	0	NA	0	0	NA
Extrapyramidal and movement disorders	5.11	5.57	0.02	5.16	5.82	0.029	4.28	4.12	0.0079
Alzheimer disease	0.66	0.66	0	0.66	0.66	0	0	0	NA
Frontotemporal dementia	0	0	NA	0	0	NA	0	0	NA
Senile degeneration of brain; not elsewhere classified	0	0	NA	0	0	NA	0	0	NA
Degeneration of nervous system due to alcohol	0	0.66	0.11	0	0	NA	0	0	NA
Neurocognitive disorder with Lewy bodies	0	0	NA	0	0	NA	0	0	NA
Mild cognitive impairment of uncertain or unknown etiology	0.79	0.92	0.014	0.79	0.79	0	0.79	0.79	0
Epilepsy and recurrent seizures	1.64	1.7	0.0051	1.59	1.66	0.0052	1.51	1.82	0.025
Migraine	8.64	8.45	0.007	8.14	7.88	0.0098	8.72	8.41	0.011
Sleep disorders	39.36	38.7	0.013	39.25	38.52	0.015	40.44	41.16	0.015
Chronic pain; not elsewhere classified	21.81	20.89	0.022	21.57	21.31	0.0065	19.67	17.76	0.049
Encephalopathy; unspecified	0.92	0.85	0.007	0.93	0.66	0.03	1.03	1.27	0.022
Diseases of the eye and adnexa	36.94	37.79	0.018	36.33	35.87	0.0096	32.75	33.86	0.024
CORTICOSTEROIDS FOR SYSTEMIC USE	59.46	61.17	0.035	59.17	59.7	0.011	55.83	56.7	0.018
THYROID THERAPY	17.03	18.47	0.038	16.74	16.28	0.012	16.18	15.62	0.015
Disorders of optic nerve and visual pathways	1.7	1.57	0.01	1.72	1.59	0.01	1.27	1.27	0
Diseases of the ear and mastoid process	25.15	25.34	0.0045	24.88	25.15	0.0061	21.65	21.41	0.0058
Diseases of the circulatory system	84.87	85.07	0.0055	84.78	84.12	0.018	82.71	82.47	0.0063
Hypertensive diseases	80.09	80.09	0	80.01	80.01	0	77.72	77.16	0.013
Hypertensive chronic kidney disease	9.37	9.43	0.0022	9.13	8.07	0.038	7.3	6.5	0.031
Ischemic heart diseases	23.45	24.03	0.014	23.43	22.24	0.028	22.76	22.05	0.017
Other forms of heart disease	32.61	32.94	0.007	32.43	32.1	0.0071	28.39	28.95	0.012
Cerebrovascular diseases	9.63	10.09	0.015	9.53	9.07	0.016	8.33	8.72	0.014
Diseases of arteries; arterioles and capillaries	14.93	15.59	0.018	14.56	14.23	0.0094	13.56	13.88	0.0092
ANTIINFECTIVES FOR SYSTEMIC USE	78.45	78.32	0.0032	78.36	77.17	0.029	75.18	74.07	0.026

Diseases of the respiratory system	61.62	61.3	0.0067	61.15	60.29	0.018	56.94	57.65	0.014
Diseases of the digestive system	65.88	67.26	0.029	65.65	65.92	0.0056	61.86	61.86	0
Diseases of liver	17.94	19.52	0.04	17.8	17.54	0.0069	16.81	15.86	0.026
Diseases of the skin and subcutaneous tissue	52.13	51.67	0.0092	51.75	52.42	0.013	47.03	47.34	0.0064
ANTINEOPLASTIC AGENTS	11.85	12.77	0.028	11.45	11.85	0.012	10.55	10.31	0.0078
Estrogens	3.8	3.01	0.043	3.44	3.24	0.011	3.33	2.93	0.023
Progestogens	3.21	3.34	0.0074	3.11	3.04	0.0038	2.62	1.98	0.042
IMMUNOSUPPRESSANTS	5.24	5.63	0.017	5.16	5.82	0.029	5.16	4.52	0.03
Psoriasis	2.49	3.21	0.043	2.58	2.52	0.0042	1.98	2.14	0.011
Married	31.04	30.06	0.021	31.24	31.9	0.014	33.23	33.15	0.0017
Diseases of the musculoskeletal system and connective tissue	76.42	76.29	0.0031	76.11	76.9	0.019	73.59	72.48	0.025
ANTIINFLAMMATORY AND ANTIRHEUMATIC PRODUCTS	53.77	55.21	0.029	53.67	53.21	0.0093	48.93	49.48	0.011
Rheumatoid arthritis with rheumatoid factor	0.72	0.66	0.0079	0.73	0.79	0.0076	0.79	0.95	0.017
Other rheumatoid arthritis	3.14	2.75	0.023	3.04	3.18	0.0076	3.25	3.73	0.026
Systemic lupus erythematosus (SLE)	0.66	0.66	0	0.66	0.66	0	0.79	0.79	0
Diseases of the genitourinary system	62.8	61.43	0.028	62.61	62.34	0.0055	57.57	55.51	0.042
OPIOIDS	54.55	54.62	0.0013	54.4	54.34	0.0013	50.28	49.33	0.019
OTHER ANALGESICS AND ANTIPYRETICS	70.07	70.33	0.0057	69.75	68.1	0.036	65.03	64.16	0.018
ANTIMIGRAINE PREPARATIONS	8.25	9.17	0.033	7.88	8.07	0.0073	7.77	7.93	0.0059
ANTIEPILEPTICS	32.35	32.68	0.007	32.03	31.64	0.0085	31.32	30.77	0.012
ANTI-PARKINSON DRUGS	4.39	4.58	0.0095	4.43	4.83	0.019	3.89	3.65	0.012
ANTIPSYCHOTICS	10.15	9.43	0.024	9.93	10.59	0.022	9.75	11.18	0.047
Lithium	0.66	0.66	0	0.66	0.66	0	0.79	0.79	0
HYPNOTICS AND SEDATIVES	38.9	39.62	0.015	38.45	37.06	0.029	36.48	37.83	0.028
Non-selective monoamine reuptake inhibitors	8.78	8.78	0	8.6	8.07	0.019	7.22	7.45	0.0091
Selective serotonin reuptake inhibitors	23.64	25.61	0.046	23.16	21.64	0.037	23.47	23.39	0.0019
Monoamine oxidase inhibitors; non-selective	0	0	NA	0	0	NA	0	0	NA

Other antidepressants	24.23	23.51	0.017	24.09	23.63	0.011	23.79	25.22	0.033
PSYCHOSTIMULANTS; AGENTS USED FOR ADHD AND NOOTROPICS	5.89	5.37	0.023	5.69	5.36	0.014	5.31	6.26	0.041
ANTI-DEMENTIA DRUGS	0.72	0.66	0.0079	0.73	0.66	0.008	0.79	0.79	0
DRUGS USED IN ADDICTIVE DISORDERS	9.3	8.58	0.025	9.27	9.53	0.0091	7.93	7.3	0.024
Acute kidney failure and chronic kidney disease	20.63	21.02	0.0097	20.45	19.26	0.03	16.89	16.18	0.019
Diseases of male genital organs	17.42	16.9	0.014	17.54	17.67	0.0035	15.07	13.24	0.052
Noninflammatory disorders of female genital tract	21.94	21.35	0.014	21.51	20.58	0.023	19.59	19.59	0
Pregnancy; childbirth and the puerperium	4.65	4.65	0	4.43	4.7	0.013	3.73	3.33	0.021
ANTIPARASITIC PRODUCTS; INSECTICIDES AND REPELLENTS	16.11	15.72	0.011	15.69	14.56	0.031	14.83	14.75	0.0022
ANTIHISTAMINES FOR SYSTEMIC USE	44.47	42.04	0.049	43.88	42.75	0.023	40.84	41.08	0.0048
Somnolence; stupor and coma	4.19	4.13	0.0033	4.1	3.97	0.0067	3.57	3.65	0.0043
Other symptoms and signs involving cognitive functions and awareness	7.4	6.61	0.031	7.28	5.96	0.053	6.5	5.87	0.026
Age-related cognitive decline	0.66	0.66	0	0.66	0.66	0	0.79	0.79	0
Suicidal ideations	0.79	0.72	0.0076	0.73	0.79	0.0076	0.87	1.27	0.039
Nonsuicidal self-harm	0	0	NA	0	0	NA	0	0	NA
Convulsions; not elsewhere classified	2.29	2.55	0.017	2.32	1.92	0.028	1.9	2.22	0.022
Abnormal weight gain	7.79	5.96	0.073	7.21	8.54	0.049	8.33	7.85	0.017
Never Married	10.87	11.2	0.01	10.59	9.66	0.031	11.18	10.31	0.028
Intracranial injury	2.03	2.16	0.0091	1.99	1.59	0.03	1.82	1.43	0.031
Suicide attempt	0	0	NA	0	0	NA	0.79	0.79	0
COVID-19	0	0	NA	0	0	NA	0	0	NA
Post COVID-19 condition	0	0	NA	0	0	NA	0	0	NA
Antidotes	13.49	14.54	0.03	13.17	12.97	0.0059	12.29	11.26	0.032
Widowed	4.32	4.26	0.0032	4.3	4.37	0.0032	4.36	5	0.03
Intentional self-harm	0.66	0.66	0	0.66	0.66	0	0.79	0.79	0
Contact with and (suspected) exposure to COVID-19	0	0	NA	0	0	NA	0	0	NA
Problems related to education and literacy	0.66	0.66	0	0.66	0.66	0	0.79	0.79	0

Persons with potential health hazards related to socioeconomic and psychosocial circumstances	3.54	3.54	0	3.44	2.85	0.034	3.17	3.41	0.013
Problems related to employment and unemployment	0.66	0.66	0	0.66	0.66	0	0.79	0.79	0
Problems related to housing and economic circumstances	0.66	0.72	0.0079	0.66	0.66	0	0.79	0.79	0
Problems related to social environment	0.66	0.66	0	0.66	0	0.12	0	0	NA
Problems related to upbringing	0.66	0.66	0	0.66	0.66	0	0.79	0.79	0
Other problems related to primary support group; including family circumstances	0.98	0.98	0	0.93	0.66	0.03	0.87	1.03	0.016
Problems related to certain psychosocial circumstances	0.66	0.66	0	0.66	0.66	0	0.79	0	0.13
Problems related to other psychosocial circumstances	1.44	1.44	0	1.39	1.59	0.016	1.03	1.59	0.049
Dietary counseling and surveillance	6.16	6.09	0.0027	5.96	6.29	0.014	5.79	5.55	0.01
Alcohol abuse counseling and surveillance	0.66	0.66	0	0.66	0.66	0	0.79	0.79	0
Drug abuse counseling and surveillance	0.66	0	0.11	0.66	0.66	0	0.79	0.79	0
Exercise counseling	0.66	0.66	0	0.66	0.66	0	0.79	0.79	0
Problems related to lifestyle	8.51	7.27	0.046	8.67	8.74	0.0023	8.48	8.48	0
Lack of physical exercise	0.66	0	0.11	0.66	0.66	0	0	0	NA
Inappropriate diet and eating habits	0.66	0.66	0	0.73	0.99	0.029	0.87	0.79	0.0087
Problems related to sleep	0.66	0.72	0.0079	0.66	0.66	0	0.79	0.79	0
Long term (current) drug therapy	50.1	48.66	0.029	49.97	50.16	0.004	52.81	53.53	0.014
Long term (current) use of insulin	33.4	32.35	0.022	33.55	33.16	0.0084	35.92	36.32	0.0083
Long term (current) use of aspirin	11.59	11.53	0.002	11.65	12.04	0.012	12.13	12.45	0.0097
Long term (current) use of oral hypoglycemic drugs	14.28	13.88	0.011	14.1	14.03	0.0019	14.35	13.8	0.016
Family history of mental and behavioral disorders	0.66	0.66	0	0.66	0.66	0	0.79	0.79	0
Personal history of mental and behavioral disorders	0.66	0.66	0	0.66	0.66	0	0.79	0.79	0
Personal history of psychological trauma; not elsewhere classified	0.66	0.66	0	0.66	0.66	0	0.79	0	0.13
Personal history of self-harm	0.66	0.66	0	0.66	0.66	0	0.79	0.79	0
Bariatric surgery status	2.62	2.03	0.039	2.52	2.38	0.0086	2.62	2.62	0
Other Race	3.27	3.34	0.0037	3.24	3.11	0.0075	3.09	3.41	0.018
aspirin	39.16	40.14	0.02	39.25	38.65	0.012	32.51	32.75	0.0051

ibuprofen	27.24	26.92	0.0074	27	26.27	0.016	24.5	25.3	0.018
glucagon	16.11	17.29	0.032	15.88	16.28	0.011	13.96	13.24	0.021

b) Baseline characteristics after matching, between 1st January 2019 and 31st December 2019

	**Semaglutide**	**Sitagliptin**		**Semaglutide**	**Empagliflozin**		**Semaglutide**	**Glipizide**	
Number	5804	5804	*SMD*	6130	6130	*SMD*	5151	5151	*SMD*
Age; mean (SD); y	56.84 (11.69)	56.65 (13.36)	0.015	57.64 (11.74)	57.67 (12.05)	0.0029	56.60 (11.93)	56.38 (13.66)	0.017
Sex									
Female	48.4	49.43	0.021	48.55	48.86	0.0062	48.9	49.6	0.014
Male	42.01	40.87	0.023	41.66	41.21	0.0093	41.45	40.79	0.013
Other	9.6	9.7	0.0035	9.79	9.94	0.0049	9.65	9.61	0.0013
Race									
White	58.92	59.22	0.006	59.07	58.96	0.0023	59.39	58.88	0.01
Black or African American	14.27	14.16	0.003	14.78	14.85	0.0018	14.29	14.19	0.0028
Asian	4.38	3.96	0.021	4.34	4.08	0.013	4.6	5.05	0.021
American Indian or Alaska Native	0.24	0.36	0.022	0.33	0.36	0.0056	0.19	0.19	0
Native Hawaiian or Other Pacific Islander	1.53	1.47	0.0057	1.22	1.27	0.0044	1.57	1.51	0.0047
Unknown	17.32	17.49	0.0045	17.16	17.5	0.0091	16.83	17.05	0.0057
Ethnicity									
Hispanic or Latino	7.37	7.1	0.011	7.7	8.27	0.021	7.63	7.38	0.0096
Not Hispanic of Latino	63.77	64.06	0.0061	64.72	63.69	0.021	64.72	65.52	0.017
Unknown	28.86	28.84	0.00038	27.59	28.04	0.01	27.64	27.1	0.012
Certain infectious and parasitic diseases	37.16	36.75	0.0086	39.48	39.17	0.0063	36.32	36.58	0.0052
ANTIOBESITY PREPARATIONS; EXCL. DIET PRODUCTS	2.95	2.67	0.017	2.81	2.64	0.01	3.24	2.97	0.016
INSULINS AND ANALOGUES	54	54.08	0.0017	55.58	56.22	0.013	52.57	52.22	0.007
Biguanides	73.92	75.57	0.038	75.32	75.2	0.0026	70.69	71.05	0.0081
Thiazolidinediones	6.84	6.79	0.0021	8.06	8.27	0.0077	5.4	5.12	0.012
Dipeptidyl peptidase 4 (DPP-4) inhibitors	NA	NA	NA	24.67	25.38	0.017	19.55	20.58	0.026
Sodium-glucose co-transporter 2 (SGLT2) inhibitors	18.54	19.07	0.014	NA	NA	NA	18.66	18.52	0.0035

Sulfonylureas	28.7	28.55	0.0034	34	34.37	0.0079	NA	NA	NA
Neoplasms	33.87	34.06	0.004	35.76	36.4	0.013	32.62	32.93	0.0066
ANTIARRHYTHMICS; CLASS I AND III	45.14	45.3	0.0031	47.29	47.44	0.0029	43.7	44.55	0.017
ANTIHYPERTENSIVES	16.78	16.66	0.0032	17.77	17.24	0.014	16.41	16.48	0.0021
DIURETICS	45.62	45.47	0.0031	48.7	48.29	0.0082	44.01	44.59	0.012
BETA BLOCKING AGENTS	42.02	42.35	0.0066	44.09	43.9	0.0039	41.43	41.27	0.0032
CALCIUM CHANNEL BLOCKERS	31.32	31.36	0.00074	33.18	33.18	0	29.92	30.07	0.0034
AGENTS ACTING ON THE RENIN-ANGIOTENSIN SYSTEM	64.04	64.21	0.0036	67.06	67.19	0.0028	61.29	62.96	0.034
LIPID MODIFYING AGENTS	69.66	69.54	0.0026	72.61	72.67	0.0015	67.89	68.47	0.013
Divorced	5.69	5.89	0.0089	5.91	6.13	0.0096	6.12	6.37	0.01
Diseases of the blood and blood-forming organs and certain disorders involving the immune mechanism	31.31	31.05	0.0056	33.28	33.08	0.0042	31.16	31.18	0.00042
Certain disorders involving the immune mechanism	3.45	3.31	0.0076	3.54	3.28	0.014	3.61	3.22	0.021
Disorders of thyroid gland	25.17	25.09	0.002	25.77	25.17	0.014	25.8	25.86	0.0013
Endocrine; nutritional and metabolic diseases	100	100	NA	100	100	NA	100	100	NA
Type 1 diabetes mellitus	11.96	11.53	0.013	12.94	13.25	0.0092	11.18	10.79	0.012
Vitamin D deficiency	25.48	25.71	0.0051	27.08	27.34	0.0059	26.81	26.07	0.017
Overweight; obesity and other hyperalimentation	60.79	61.72	0.019	60.91	61.71	0.016	60.59	60.98	0.008
Metabolic disorders	84.6	84.48	0.0033	86.26	86.57	0.009	84.06	84.82	0.021
Disorders of lipoprotein metabolism and other lipidemias	80.5	80.43	0.0017	82.64	82.82	0.0047	79.95	80.86	0.023
Other disorders of fluid; electrolyte and acid-base balance	19.25	19.09	0.0039	20.7	20.75	0.0012	18.99	19.05	0.0015
Metabolic syndrome and other insulin resistance	4.19	4.03	0.0078	4.54	4.45	0.0039	4.43	4.19	0.011
Vascular dementia	0.17	0.17	0	0.23	0.21	0.0035	0.21	0.23	0.0041
Mental; Behavioral and Neurodevelopmental disorders	49.6	50.52	0.018	51.21	51.65	0.0088	49.35	49.74	0.0078
Dementia in other diseases classified elsewhere	0.34	0.41	0.011	0.44	0.47	0.0048	0.37	0.48	0.018
Unspecified dementia	0.71	0.69	0.0021	0.8	0.8	0	0.7	0.83	0.016
Delirium due to known physiological condition	0.43	0.34	0.014	0.41	0.42	0.0025	0.43	0.27	0.026
Other specified mental disorders due to known physiological condition	0.45	0.53	0.012	0.46	0.59	0.018	0.21	0.29	0.015

Alcohol related disorders	3.03	3.48	0.025	3.31	3.36	0.0027	2.74	2.78	0.0024
Mental and behavioral disorders due to psychoactive substance use	19.14	19.9	0.019	19.62	19.93	0.0078	18.93	18.89	0.00099
Opioid related disorders	1.47	1.71	0.019	1.63	1.65	0.0013	1.32	0.99	0.031
Cannabis related disorders	1.34	1.17	0.015	1.29	1.13	0.015	1.2	1.15	0.0054
Cocaine related disorders	0.72	0.9	0.019	0.64	0.49	0.02	0.58	0.6	0.0025
Other stimulant related disorders	0.48	0.48	0	0.47	0.46	0.0024	0.45	0.51	0.0085
Other psychoactive substance related disorders	1.29	1.52	0.019	1.49	1.42	0.0055	1.3	1.2	0.0087
Schizophrenia; schizotypal; delusional; and other non-mood psychotic disorders	1.6	1.74	0.011	1.88	1.84	0.0024	1.73	1.92	0.015
Manic episode	0.17	0.17	0	0.21	0.2	0.0036	0.19	0.21	0.0043
Mood [affective] disorders	28.86	29.5	0.014	30.15	30.46	0.0067	28.15	28.34	0.0043
Bipolar disorder	3.15	3.41	0.015	3.2	3.41	0.012	3.24	2.95	0.017
Depressive episode	25.22	25.5	0.0063	26.23	26.56	0.0074	24.48	24.42	0.0014
Major depressive disorder; recurrent	7.01	6.94	0.0027	7.68	7.33	0.014	6.7	6.64	0.0023
Persistent mood [affective] disorders	3.46	3.45	0.00094	3.72	3.75	0.0017	3.05	3.4	0.02
Unspecified mood [affective] disorder	1.9	2.12	0.016	2.43	2.35	0.0053	2.02	1.98	0.0028
Anxiety; dissociative; stress-related; somatoform and other nonpsychotic mental disorders	27.91	28.26	0.0077	29.41	29.85	0.0096	27.41	27.9	0.011
Eating disorders	0.88	0.74	0.015	0.96	0.99	0.0033	1.13	1.05	0.0075
Behavioral syndromes associated with physiological disturbances and physical factors	6	6.31	0.013	6.39	6.46	0.0027	5.75	5.67	0.0033
Sleep disorders not due to a substance or known physiological condition	3.72	4.2	0.025	3.88	4.18	0.015	3.46	3.51	0.0032
Disorders of adult personality and behavior	1.08	1.05	0.0034	1.14	1.21	0.0061	1.03	1.03	0
Attention-deficit hyperactivity disorders	1.67	1.88	0.016	1.68	1.52	0.013	1.63	1.69	0.0046
Behavioral and emotional disorders with onset usually occurring in childhood and adolescence	2.22	2.52	0.019	2.27	2.14	0.0089	2.21	2.35	0.0091
Diseases of the nervous system	61.89	61.7	0.0039	63.65	63.8	0.0031	60.38	61.08	0.014
Encephalitis; myelitis and encephalomyelitis	0.19	0.17	0.0041	0.16	0.16	0	0.19	0.19	0
Encephalitis; myelitis and encephalomyelitis in diseases classified elsewhere	0.17	0	0.059	0	0	NA	0	0	NA
Extrapyramidal and movement disorders	5.34	5.39	0.0023	5.53	5.91	0.016	5.09	5.18	0.0044

Alzheimer disease	0.17	0.17	0	0.26	0.28	0.0031	0.19	0.19	0
Frontotemporal dementia	0.17	0.17	0	0.16	0	0.057	0	0.19	0.062
Senile degeneration of brain; not elsewhere classified	0	0	NA	0.16	0.16	0	0.19	0	0.062
Degeneration of nervous system due to alcohol	0	0	NA	0	0	NA	0	0.19	0.062
Neurocognitive disorder with Lewy bodies	0.17	0.17	0	0.16	0.16	0	0.19	0.19	0
Mild cognitive impairment of uncertain or unknown etiology	0.76	0.71	0.0061	0.78	0.72	0.0076	0.72	0.72	0
Epilepsy and recurrent seizures	1.95	1.95	0	1.78	1.96	0.013	1.8	1.94	0.01
Migraine	7.67	7.87	0.0077	7.52	7.47	0.0019	7.32	7.49	0.0067
Sleep disorders	37.58	37.77	0.0039	38.58	38.66	0.0017	37.31	37.97	0.014
Chronic pain; not elsewhere classified	22.48	22.83	0.0082	23.82	24.24	0.0099	21.51	21.49	0.00047
Encephalopathy; unspecified	1.08	1.17	0.0082	1.22	1.26	0.0029	1.07	1.2	0.013
Diseases of the eye and adnexa	33.05	33.08	0.00073	35.45	35.69	0.0051	31.59	32.36	0.017
CORTICOSTEROIDS FOR SYSTEMIC USE	55.2	55.81	0.012	58.04	58.29	0.005	54.38	55.21	0.017
THYROID THERAPY	14.52	14.66	0.0039	15.37	14.93	0.012	14.93	15.45	0.015
Disorders of optic nerve and visual pathways	1.71	1.48	0.018	1.83	1.73	0.0074	2.02	2.17	0.011
Diseases of the ear and mastoid process	22.76	22.55	0.0049	24.54	24.52	0.00038	22.09	22.77	0.016
Diseases of the circulatory system	85.75	85.42	0.0093	87.03	87.37	0.01	84.7	85.61	0.026
Hypertensive diseases	81.58	81.48	0.0027	82.82	83.18	0.0096	80.1	81.01	0.023
Hypertensive chronic kidney disease	8.8	8.91	0.0036	9.87	9.3	0.019	8.91	8.66	0.0089
Ischemic heart diseases	23.76	23.09	0.016	25.02	25.25	0.0053	23.39	23.18	0.0051
Other forms of heart disease	31.22	30.57	0.014	33.28	33.12	0.0035	30.44	30.54	0.0021
Cerebrovascular diseases	10.3	9.92	0.013	11.08	11.21	0.0041	10.17	10.43	0.0083
Diseases of arteries; arterioles and capillaries	14.35	14.03	0.0094	15.86	15.84	0.00045	14.09	14.48	0.011
ANTIINFECTIVES FOR SYSTEMIC USE	77.02	76.79	0.0053	79.02	78.76	0.0064	76.14	76.55	0.0096
Diseases of the respiratory system	57.56	57.63	0.0014	59.54	59.98	0.009	56.14	56.84	0.014
Diseases of the digestive system	63.13	63.61	0.01	65.11	65.4	0.0062	62.09	62.22	0.0028
Diseases of liver	16.99	16.92	0.0018	17.83	17.96	0.0034	16.68	16.73	0.0016

Diseases of the skin and subcutaneous tissue	46.64	46.66	0.00035	48.84	49.13	0.0059	44.77	45.02	0.0051
ANTINEOPLASTIC AGENTS	10.13	9.89	0.008	10.34	10.33	0.00054	9.45	9.61	0.0053
Estrogens	2.6	2.55	0.0033	2.37	2.15	0.014	2.52	2.45	0.005
Progestogens	2.74	3	0.015	2.71	2.45	0.016	2.74	2.97	0.014
IMMUNOSUPPRESSANTS	5.38	5.32	0.0023	5.32	5.33	0.00073	5.61	5.46	0.0068
Psoriasis	2.98	2.79	0.011	3.12	2.97	0.0085	2.93	2.62	0.019
Married	31	29.82	0.025	30.93	30.52	0.0088	32.34	32.46	0.0025
Diseases of the musculoskeletal system and connective tissue	72.16	71.8	0.0081	74.73	74.6	0.003	71.09	71.27	0.0039
ANTIINFLAMMATORY AND ANTIRHEUMATIC PRODUCTS	49.83	50.24	0.0083	51.53	51.58	0.00098	48.73	49.43	0.014
Rheumatoid arthritis with rheumatoid factor	0.76	0.79	0.0039	0.69	0.73	0.0058	0.7	0.76	0.0069
Other rheumatoid arthritis	2.86	2.55	0.019	2.63	2.69	0.0041	2.64	2.72	0.0048
Systemic lupus erythematosus (SLE)	0.6	0.59	0.0022	0.65	0.7	0.006	0.47	0.51	0.0056
Diseases of the genitourinary system	60.18	59.6	0.012	63.28	63.36	0.0017	60.01	60.05	0.00079
OPIOIDS	52.91	53.05	0.0028	54.8	54.53	0.0052	51.02	51.54	0.01
OTHER ANALGESICS AND ANTIPYRETICS	67.83	68.07	0.0052	69.82	69.82	0	65.6	66.1	0.011
ANTIMIGRAINE PREPARATIONS	7.94	7.86	0.0032	7.99	8.19	0.0072	7.18	7.43	0.0097
ANTIEPILEPTICS	32.72	32.67	0.0011	33.7	34.14	0.0093	30.81	30.5	0.0067
ANTI-PARKINSON DRUGS	4.43	4.79	0.017	4.93	5.25	0.015	4.5	4.54	0.0019
ANTIPSYCHOTICS	13.15	13.15	0	13.61	13.87	0.0076	12.58	12.39	0.0059
Lithium	0.17	0.17	0	0.16	0.16	0	0.19	0.19	0
HYPNOTICS AND SEDATIVES	39.68	39.08	0.012	41.66	41.52	0.003	38.42	39.2	0.016
Non-selective monoamine reuptake inhibitors	7.6	7.58	0.00065	7.98	7.95	0.0012	7.03	7.4	0.014
Selective serotonin reuptake inhibitors	21.38	21.55	0.0042	22.72	22.87	0.0035	20.29	20.23	0.0014
Monoamine oxidase inhibitors; non-selective	0.17	0	0.059	0.16	0.16	0	0.19	0.19	0
Other antidepressants	23.64	23.98	0.0081	24.37	24.44	0.0015	22.77	22.36	0.0098
PSYCHOSTIMULANTS; AGENTS USED FOR ADHD AND NOOTROPICS	5.96	5.94	0.00073	5.64	5.86	0.0091	5.4	5.77	0.016
ANTI-DEMENTIA DRUGS	0.78	0.88	0.011	0.82	0.67	0.017	0.74	0.81	0.0088

DRUGS USED IN ADDICTIVE DISORDERS	8.34	8.58	0.0087	8.45	8.55	0.0035	7.98	7.98	0
Acute kidney failure and chronic kidney disease	20.5	20.35	0.0038	23.08	22.15	0.022	20.66	20.5	0.0038
Diseases of male genital organs	16.85	16.07	0.021	18.19	18.3	0.003	16.11	16.39	0.0074
Noninflammatory disorders of female genital tract	19.4	20.02	0.016	20.18	20.47	0.0073	19.55	19.2	0.0088
Pregnancy; childbirth and the puerperium	4.19	4.08	0.0052	4.14	3.74	0.021	4.17	4.02	0.0078
ANTIPARASITIC PRODUCTS; INSECTICIDES AND REPELLENTS	14.04	13.89	0.0045	14.93	14.78	0.0041	13.32	13.65	0.0097
ANTIHISTAMINES FOR SYSTEMIC USE	44.44	44.78	0.0069	46.1	46.31	0.0043	43.02	43.66	0.013
Somnolence; stupor and coma	3.41	3.53	0.0066	3.74	3.98	0.013	3.2	2.97	0.013
Other symptoms and signs involving cognitive functions and awareness	6.79	7	0.0082	7.47	7.63	0.0062	6.66	7.05	0.015
Age-related cognitive decline	0.17	0	0.059	0.16	0.16	0	0.19	0.19	0
Suicidal ideations	1.41	1.48	0.0058	1.4	1.45	0.0041	1.3	1.34	0.0034
Nonsuicidal self-harm	0	0	NA	0	0	NA	0	0	NA
Convulsions; not elsewhere classified	2.31	2.43	0.0079	2.28	2.51	0.015	2.12	2.17	0.004
Abnormal weight gain	5.13	5.01	0.0055	5.46	5.43	0.0014	5.51	5.44	0.0034
Never Married	10.7	10.61	0.0028	10.51	10.55	0.0016	11.49	11.49	0
Intracranial injury	2.26	2.5	0.016	2.25	2.17	0.0055	1.94	1.8	0.01
Suicide attempt	0.17	0.17	0	0.16	0.16	0	0.19	0.19	0
COVID-19	0	0	NA	0.16	0.16	0	0	0.19	0.062
Post COVID-19 condition	0	0	NA	0	0	NA	0	0	NA
Antidotes	14.78	15.01	0.0063	15.55	15.35	0.0054	15.14	14.72	0.012
Widowed	4.5	4.5	0	4.84	4.89	0.0023	4.41	4.91	0.024
Intentional self-harm	0.17	0.17	0	0.16	0.2	0.0077	0.19	0.19	0
Contact with and (suspected) exposure to COVID-19	0	0	NA	0.16	0	0.057	0.19	0	0.062
Problems related to education and literacy	0.17	0.17	0	0.16	0.18	0.0039	0.19	0.19	0
Persons with potential health hazards related to socioeconomic and psychosocial circumstances	3.31	3.43	0.0067	3.9	3.75	0.0077	3.28	2.99	0.017
Problems related to employment and unemployment	0.43	0.4	0.0054	0.41	0.39	0.0026	0.45	0.48	0.0057
Problems related to housing and economic circumstances	0.76	0.88	0.013	1.03	0.99	0.0033	0.66	0.54	0.015

Problems related to social environment	0.21	0.17	0.0079	0.28	0.28	0	0.19	0.19	0
Problems related to upbringing	0.31	0.29	0.0031	0.23	0.2	0.0071	0.25	0.25	0
Other problems related to primary support group; including family circumstances	0.95	1.03	0.0087	1.14	1.04	0.0094	1.07	0.91	0.016
Problems related to certain psychosocial circumstances	0.17	0.17	0	0.16	0.16	0	0.19	0.19	0
Problems related to other psychosocial circumstances	0.81	0.88	0.0075	0.96	1.01	0.005	0.76	0.74	0.0023
Dietary counseling and surveillance	6.89	6.44	0.018	7.44	7.34	0.0037	7.79	7.47	0.012
Alcohol abuse counseling and surveillance	0.28	0.26	0.0033	0.26	0.26	0	0.25	0.19	0.012
Drug abuse counseling and surveillance	0.17	0.17	0	0.16	0.16	0	0.19	0	0.062
Exercise counseling	0.5	0.53	0.0048	0.54	0.49	0.0068	0.62	0.72	0.012
Problems related to lifestyle	8.03	8.41	0.014	9.02	8.73	0.01	8.13	8.33	0.0071
Lack of physical exercise	0.17	0.17	0	0.16	0.16	0	0.19	0.19	0
Inappropriate diet and eating habits	0.4	0.29	0.018	0.59	0.44	0.021	0.58	0.39	0.028
Problems related to sleep	0.52	0.6	0.012	0.72	0.62	0.012	0.6	0.64	0.0049
Long term (current) drug therapy	54.95	54.81	0.0028	55.94	56.3	0.0072	55.19	54.57	0.012
Long term (current) use of insulin	35.7	35.1	0.013	37.18	37.9	0.015	36.15	36.5	0.0073
Long term (current) use of aspirin	14.09	14.23	0.004	15.32	15.12	0.0054	14.29	14.42	0.0039
Long term (current) use of oral hypoglycemic drugs	19.62	19.87	0.0061	20.16	19.82	0.0086	19.05	18.52	0.013
Family history of mental and behavioral disorders	0.67	0.81	0.016	0.7	0.73	0.0039	0.76	0.76	0
Personal history of mental and behavioral disorders	0.81	0.86	0.0057	0.85	0.82	0.0036	0.7	0.58	0.015
Personal history of psychological trauma; not elsewhere classified	0.21	0.17	0.0079	0.16	0.18	0.0039	0.19	0.19	0
Personal history of self-harm	0.29	0.29	0	0.29	0.28	0.0031	0.25	0.29	0.0075
Bariatric surgery status	2.34	2.38	0.0023	2.23	2.25	0.0011	2.43	2.12	0.021
Other Race	3.34	3.34	0	3.1	2.98	0.0066	3.13	3.13	0
aspirin	37.51	37.56	0.0011	39.62	39.98	0.0073	35.88	36.5	0.013
ibuprofen	25	25.26	0.006	25.76	25.97	0.0048	23.92	24.02	0.0023
glucagon	17.23	17.01	0.0059	18.24	18.27	0.00084	17.24	16.66	0.016

c) Baseline characteristics after matching, between 1st January 2020 and 31st May 2021

	Semaglutide	Sitagliptin		Semaglutide	Empagliflozin		Semaglutide	Glipizide	
Number	14367	14367	*SMD*	15718	15718	*SMD*	12552	12552	*SMD*
Age; mean (SD); y	57.95 (11.99)	57.68 (13.04)	0.022	58.11 (12.39)	58.09 (12.50)	0.0024	57.13 (12.24)	56.99 (13.35)	0.011
Sex									
Female	48.3	48.07	0.0046	48.36	47.97	0.0078	48.71	48.2	0.01
Male	45.37	45.88	0.01	43.76	44.01	0.005	44.29	44.93	0.013
Other	6.33	6.05	0.012	7.88	8.02	0.0052	7	6.87	0.0053
Race									
White	60.68	61.27	0.012	60.1	60.13	0.00078	59.8	59.58	0.0044
Black or African American	16.06	15.84	0.0061	15.64	15.7	0.0016	16.1	16.16	0.0017
Asian	4.18	4.21	0.0017	4.27	4.29	0.00094	4.53	4.59	0.0027
American Indian or Alaska Native	0.37	0.31	0.0096	0.38	0.36	0.0042	0.36	0.36	0
Native Hawaiian or Other Pacific Islander	1.14	1.17	0.0033	0.99	1.05	0.0057	1.2	1.31	0.011
Unknown	13.54	13.26	0.0082	14.89	14.82	0.0021	13.93	13.86	0.0018
Ethnicity									
Hispanic or Latino	7.06	7.36	0.012	6.65	6.74	0.0036	7.46	7.59	0.0051
Not Hispanic of Latino	67.07	67.21	0.003	67.34	67.3	0.00081	66.68	66.97	0.0061
Unknown	25.87	25.43	0.01	26.02	25.96	0.0012	25.86	25.44	0.0097
Certain infectious and parasitic diseases	40.97	40.88	0.0017	42.87	43.46	0.012	39.81	39.71	0.0021
ANTIOBESITY PREPARATIONS; EXCL. DIET PRODUCTS	2.48	2.27	0.014	2.7	2.54	0.01	2.56	2.21	0.023
INSULINS AND ANALOGUES	48.72	48.49	0.0047	51.49	51.96	0.0094	47.24	47.15	0.0019
Biguanides	75.38	76.27	0.021	76.33	76.79	0.011	73.07	73.78	0.016
Thiazolidinediones	6.26	6.27	0.00057	7.16	7.12	0.0017	4.96	5.22	0.012
Dipeptidyl peptidase 4 (DPP-4) inhibitors	NA	NA	NA	22.41	22.68	0.0064	18.74	19.01	0.0069
Sodium-glucose co-transporter 2 (SGLT2) inhibitors	19.41	19.47	0.0016	NA	NA	NA	18.79	19.14	0.0089

Sulfonylureas	31.05	31.79	0.016	33.67	34.81	0.024	NA	NA	NA
Neoplasms	34.43	34.5	0.0013	36.89	36.69	0.0042	33.21	33.17	0.00085
ANTIARRHYTHMICS; CLASS I AND III	47.53	47.71	0.0038	50	50.08	0.0015	46.57	46.46	0.0021
ANTIHYPERTENSIVES	17.21	16.96	0.0067	18.02	18.16	0.0036	16.59	16.53	0.0017
DIURETICS	46.93	46.72	0.0042	49.29	49.64	0.0071	44.9	44.84	0.0011
BETA BLOCKING AGENTS	43.86	43.93	0.0014	45.36	45.26	0.002	41.75	41.75	0.00016
CALCIUM CHANNEL BLOCKERS	32.92	32.99	0.0015	34.55	34.81	0.0053	31.38	31.77	0.0084
AGENTS ACTING ON THE RENIN-ANGIOTENSIN SYSTEM	65.09	65.18	0.0018	67.7	67.94	0.0052	62.33	62.92	0.012
LIPID MODIFYING AGENTS	72.11	72.55	0.0098	73.97	74.51	0.012	69.57	69.73	0.0036
Divorced	5.86	5.97	0.0047	5.73	5.64	0.0038	5.85	5.5	0.015
Diseases of the blood and blood-forming organs and certain disorders involving the immune mechanism	33.7	33.57	0.0028	35.23	35.54	0.0064	32.55	31.72	0.018
Certain disorders involving the immune mechanism	3.39	3.45	0.0034	3.84	3.75	0.0047	3.42	3.33	0.0049
Disorders of thyroid gland	23.54	23.12	0.0099	24.47	24.27	0.0047	23.59	23.31	0.0066
Endocrine; nutritional and metabolic diseases	100	100	NA	100	100	NA	100	100	NA
Type 1 diabetes mellitus	8.16	8.11	0.002	9.8	9.98	0.006	7.95	7.58	0.014
Vitamin D deficiency	26.02	25.59	0.0097	27.22	27.69	0.01	25.33	25.05	0.0064
Overweight; obesity and other hyperalimentation	58.39	58.74	0.0071	60.67	61.16	0.01	58.42	58.94	0.011
Metabolic disorders	84.21	84.15	0.0015	85.56	85.77	0.006	82.99	83.01	0.00042
Disorders of lipoprotein metabolism and other lipidemias	79.81	79.68	0.0035	81.43	81.71	0.0072	78.35	78.41	0.0015
Other disorders of fluid; electrolyte and acid-base balance	20.16	20.24	0.0019	21.35	21.74	0.0094	19.48	19.07	0.011
Metabolic syndrome and other insulin resistance	3.34	3.08	0.015	3.92	3.95	0.0016	3.42	3.15	0.015
Vascular dementia	0.31	0.28	0.0052	0.34	0.34	0	0.28	0.29	0.0015
Mental; Behavioral and Neurodevelopmental disorders	51.49	51.32	0.0033	53.32	53.59	0.0054	50.92	50.53	0.008
Dementia in other diseases classified elsewhere	0.33	0.33	0	0.41	0.43	0.0039	0.41	0.36	0.009
Unspecified dementia	0.77	0.61	0.019	0.81	0.74	0.008	0.72	0.69	0.0038
Delirium due to known physiological condition	0.49	0.38	0.017	0.55	0.55	0	0.49	0.6	0.015
Other specified mental disorders due to known physiological condition	0.54	0.54	0.00095	0.56	0.53	0.0043	0.44	0.41	0.0049

Alcohol related disorders	3.24	3.22	0.0012	3.3	3.38	0.005	3.13	3.04	0.0055
Mental and behavioral disorders due to psychoactive substance use	19.93	20.14	0.005	20.45	20.77	0.008	19.73	19.74	4.00E-04
Opioid related disorders	1.94	1.91	0.0025	1.93	1.96	0.0023	1.82	1.89	0.0053
Cannabis related disorders	1.42	1.43	0.00059	1.46	1.43	0.0021	1.47	1.45	0.002
Cocaine related disorders	0.9	0.76	0.015	0.9	0.89	0.00068	0.83	0.81	0.0018
Other stimulant related disorders	0.56	0.55	0.00094	0.65	0.67	0.0031	0.63	0.64	0.001
Other psychoactive substance related disorders	1.52	1.43	0.0069	1.62	1.62	5.00E-04	1.55	1.68	0.01
Schizophrenia; schizotypal; delusional; and other non-mood psychotic disorders	2.01	2.05	0.0025	2.05	2.14	0.0062	1.8	1.85	0.0036
Manic episode	0.19	0.2	0.0016	0.2	0.2	0.0014	0.18	0.17	0.0019
Mood [affective] disorders	29.16	29.14	0.00031	30.82	30.89	0.0015	28.74	28.62	0.0028
Bipolar disorder	3.15	3.43	0.016	3.31	3.47	0.0088	3.24	3.46	0.012
Depressive episode	25.07	24.96	0.0026	26.74	26.55	0.0043	24.79	24.71	0.0018
Major depressive disorder; recurrent	7.55	7.52	0.0011	8.22	8.38	0.006	7.23	7.08	0.0062
Persistent mood [affective] disorders	3.24	3.29	0.0024	3.74	3.77	0.0013	3.09	3.09	0
Unspecified mood [affective] disorder	2.52	2.53	0.00089	2.7	2.72	0.00078	2.3	2.36	0.0037
Anxiety; dissociative; stress-related; somatoform and other nonpsychotic mental disorders	30.24	30.33	0.0021	31.82	31.7	0.0026	29.92	29.41	0.011
Eating disorders	0.7	0.66	0.0042	0.83	0.9	0.0076	0.7	0.68	0.0029
Behavioral syndromes associated with physiological disturbances and physical factors	6.1	6.12	0.00058	6.77	6.85	0.0033	6.17	6.1	0.003
Sleep disorders not due to a substance or known physiological condition	4.19	4.26	0.0035	4.62	4.55	0.003	4.29	4.3	0.00079
Disorders of adult personality and behavior	1.34	1.27	0.0061	1.44	1.48	0.0032	1.23	1.24	0.0014
Attention-deficit hyperactivity disorders	1.66	1.67	0.00054	1.72	1.67	0.0039	1.71	1.73	0.0018
Behavioral and emotional disorders with onset usually occurring in childhood and adolescence	2.3	2.33	0.0019	2.44	2.44	0.00041	2.44	2.46	0.0015
Diseases of the nervous system	63.19	63.4	0.0043	65.83	66.47	0.013	62.58	63.1	0.011
Encephalitis; myelitis and encephalomyelitis	0.16	0.19	0.0067	0.19	0.18	0.003	0.21	0.21	0.0017
Encephalitis; myelitis and encephalomyelitis in diseases classified elsewhere	0.07	0	0.037	0.064	0.064	0	0.08	0.08	0
Extrapyramidal and movement disorders	5.59	5.61	0.00091	6.15	6.54	0.016	5.41	5.58	0.0073

Alzheimer disease	0.22	0.14	0.02	0.29	0.31	0.0047	0.28	0.26	0.0046
Frontotemporal dementia	0.07	0.07	0	0.064	0.064	0	0.08	0.08	0
Senile degeneration of brain; not elsewhere classified	0.07	0.07	0	0.064	0.064	0	0.08	0.08	0
Degeneration of nervous system due to alcohol	0	0.07	0.037	0	0.064	0.036	0	0	NA
Neurocognitive disorder with Lewy bodies	0.07	0.07	0	0.064	0.064	0	0.08	0.08	0
Mild cognitive impairment of uncertain or unknown etiology	0.9	0.83	0.0068	0.99	1.02	0.0032	0.94	0.84	0.01
Epilepsy and recurrent seizures	1.76	1.6	0.012	1.82	1.77	0.0038	1.86	1.84	0.0012
Migraine	7.86	7.74	0.0044	8.28	8.33	0.0018	7.98	8.25	0.0099
Sleep disorders	36.85	37.13	0.0059	39.29	40.06	0.016	36.81	36.83	5.00E-04
Chronic pain; not elsewhere classified	24.39	24.9	0.012	26.59	26.66	0.0017	24.15	24.7	0.013
Encephalopathy; unspecified	1.46	1.43	0.0018	1.5	1.5	0	1.44	1.53	0.0072
Diseases of the eye and adnexa	32.15	32.14	0.00015	34.78	35.34	0.012	30.33	30.44	0.0024
CORTICOSTEROIDS FOR SYSTEMIC USE	58.73	58.64	0.0018	60.66	60.92	0.0053	57.11	57.17	0.0011
THYROID THERAPY	14.64	14.68	0.0012	15.14	15.05	0.0025	14.71	14.63	0.002
Disorders of optic nerve and visual pathways	1.54	1.59	0.0045	1.75	1.83	0.0062	1.62	1.53	0.007
Diseases of the ear and mastoid process	23.8	23.83	0.00082	25.88	25.99	0.0026	22.74	22.8	0.0015
Diseases of the circulatory system	85.02	84.9	0.0035	86.58	86.72	0.0039	83.99	83.98	0.00022
Hypertensive diseases	80.06	79.91	0.0037	81.89	82.12	0.006	79.02	79.09	0.0018
Hypertensive chronic kidney disease	8.86	8.8	0.002	10.17	10.52	0.012	8.69	8.37	0.011
Ischemic heart diseases	23.92	23.61	0.0074	25.2	25.21	0.00029	23.1	23.11	0.00038
Other forms of heart disease	32.51	32.24	0.0058	34.09	34.15	0.0011	31.19	31.35	0.0034
Cerebrovascular diseases	10.98	10.47	0.016	11.51	11.76	0.0079	10.62	10.65	0.001
Diseases of arteries; arterioles and capillaries	14.9	14.29	0.017	15.93	15.95	0.00052	14.29	14.54	0.007
ANTIINFECTIVES FOR SYSTEMIC USE	78.69	79.19	0.012	80.58	80.49	0.0022	77.47	77.12	0.0084
Diseases of the respiratory system	59.52	59.65	0.0027	61.53	61.27	0.0055	58.35	58.25	0.0021
Diseases of the digestive system	63.84	64.16	0.0067	66.39	66.11	0.0058	62.95	63.12	0.0036
Diseases of liver	17.19	17.31	0.0031	18.44	18.76	0.0083	17.63	17.24	0.01

Diseases of the skin and subcutaneous tissue	47.05	47.37	0.0066	49.9	50.08	0.0036	45.43	45.28	0.0029
ANTINEOPLASTIC AGENTS	10.54	10.79	0.0081	11.65	11.87	0.0069	10.2	10.37	0.0058
Estrogens	2.53	2.37	0.01	2.42	2.36	0.0042	2.59	2.28	0.02
Progestogens	2.64	2.57	0.0044	2.89	2.79	0.0061	2.66	2.65	5.00E-04
IMMUNOSUPPRESSANTS	5.13	5.09	0.0019	5.57	5.45	0.005	5.14	4.91	0.011
Psoriasis	2.94	3.03	0.0049	3.04	3.01	0.0019	2.65	2.58	0.0045
Married	31.11	31	0.0024	30.51	30.3	0.0044	31.56	31.88	0.007
Diseases of the musculoskeletal system and connective tissue	74.06	73.89	0.004	76.58	76.81	0.0054	72.87	73.33	0.01
ANTIINFLAMMATORY AND ANTIRHEUMATIC PRODUCTS	53.09	53.32	0.0047	54.89	54.85	0.00064	51.1	51.99	0.018
Rheumatoid arthritis with rheumatoid factor	0.48	0.47	0.001	0.55	0.61	0.0084	0.49	0.5	0.0011
Other rheumatoid arthritis	2.29	2.3	0.00046	2.37	2.49	0.0079	2.26	2.26	0
Systemic lupus erythematosus (SLE)	0.57	0.58	0.0018	0.61	0.59	0.0025	0.57	0.63	0.0072
Diseases of the genitourinary system	61.89	61.81	0.0016	64.34	64.72	0.008	61.02	60.8	0.0044
OPIOIDS	53.14	53.07	0.0015	55.27	55.2	0.0014	51.78	51.48	0.0061
OTHER ANALGESICS AND ANTIPYRETICS	68.24	68.41	0.0036	69.86	70.39	0.012	66.8	67.11	0.0064
ANTIMIGRAINE PREPARATIONS	7.96	7.51	0.017	8.11	8.07	0.0014	7.74	7.78	0.0018
ANTIEPILEPTICS	33.12	33.56	0.0093	34.85	35.37	0.011	31.98	32.29	0.0067
ANTI-PARKINSON DRUGS	4.87	4.91	0.0023	5.3	5.29	0.00028	4.69	4.76	0.003
ANTIPSYCHOTICS	15.34	15.69	0.0096	16.24	16.46	0.006	14.74	14.35	0.011
Lithium	0.11	0.12	0.004	0.12	0.12	0.0019	0.08	0.08	0
HYPNOTICS AND SEDATIVES	41.05	41.27	0.0047	42.92	42.8	0.0026	39.39	38.95	0.009
Non-selective monoamine reuptake inhibitors	7.22	7.36	0.0054	7.81	7.85	0.0014	6.68	6.84	0.0063
Selective serotonin reuptake inhibitors	23.05	23.16	0.0025	24	24.37	0.0086	22.49	22.3	0.0046
Monoamine oxidase inhibitors; non-selective	0.07	0.07	0	0.064	0	0.036	0.08	0.08	0
Other antidepressants	24.05	23.87	0.0041	25.54	25.55	0.00029	23.49	23.44	0.0013
PSYCHOSTIMULANTS; AGENTS USED FOR ADHD AND NOOTROPICS	6.09	6.09	0	6.79	6.76	0.0013	5.7	5.93	0.01
ANTI-DEMENTIA DRUGS	0.72	0.63	0.011	0.77	0.78	0.0014	0.71	0.65	0.0078

DRUGS USED IN ADDICTIVE DISORDERS	8.25	8.45	0.0073	8.77	8.91	0.0052	7.94	8.08	0.005
Acute kidney failure and chronic kidney disease	21.58	21.56	0.00068	23.76	24.12	0.0086	20.87	20.44	0.011
Diseases of male genital organs	18.27	18.47	0.005	19.11	19.32	0.0053	17.54	17.69	0.004
Noninflammatory disorders of female genital tract	19.4	19.18	0.0055	20.33	20.21	0.0028	19.23	19.33	0.0024
Pregnancy; childbirth and the puerperium	3.86	3.69	0.0088	3.81	3.62	0.0098	4	3.95	0.0024
ANTIPARASITIC PRODUCTS; INSECTICIDES AND REPELLENTS	14.89	14.71	0.0051	15.8	15.91	0.003	14.32	14.6	0.0079
ANTIHISTAMINES FOR SYSTEMIC USE	45.51	45.97	0.0091	48.02	48.1	0.0018	44.61	44.69	0.0014
Somnolence; stupor and coma	3.99	3.68	0.016	4.52	4.48	0.0021	3.9	3.71	0.01
Other symptoms and signs involving cognitive functions and awareness	8.32	7.66	0.025	8.63	8.63	0	7.85	7.71	0.0051
Age-related cognitive decline	0.07	0.11	0.014	0.089	0.095	0.0021	0.08	0.08	0
Suicidal ideations	1.45	1.57	0.0097	1.53	1.58	0.0046	1.37	1.47	0.0087
Nonsuicidal self-harm	0	0.07	0.037	0	0	NA	0	0	NA
Convulsions; not elsewhere classified	2.34	2.35	0.00092	2.44	2.5	0.0037	2.53	2.6	0.0045
Abnormal weight gain	4.32	4.17	0.0076	4.99	4.87	0.0053	4.55	4.23	0.016
Never Married	11.33	11.66	0.01	10.68	10.49	0.006	11.27	11.49	0.0068
Intracranial injury	2.09	1.96	0.0089	2.11	2.23	0.0079	2.04	1.93	0.008
Suicide attempt	0.2	0.21	0.0031	0.17	0.14	0.0065	0.15	0.16	0.002
COVID-19	4	3.94	0.0032	3.7	3.83	0.0067	4.01	4	0.00041
Post COVID-19 condition	0.07	0.07	0	0.064	0.064	0	0.08	0	0.04
Antidotes	18.39	18.25	0.0036	19.39	19.44	0.0011	17.9	17.72	0.0048
Widowed	4.71	4.52	0.009	4.8	4.79	3.00E-04	4.53	4.4	0.0066
Intentional self-harm	0.15	0.17	0.0052	0.16	0.16	0	0.14	0.16	0.0041
Contact with and (suspected) exposure to COVID-19	5	4.85	0.0068	5.03	5.12	0.0038	5.03	4.92	0.0048
Problems related to education and literacy	0.17	0.17	0	0.18	0.18	0	0.17	0.16	0.002
Persons with potential health hazards related to socioeconomic and psychosocial circumstances	3.93	3.95	0.00072	4.47	4.31	0.0075	3.77	3.77	0
Problems related to employment and unemployment	0.39	0.39	0	0.43	0.47	0.0067	0.37	0.38	0.0026
Problems related to housing and economic circumstances	1.07	1.08	0.00067	1.18	1.21	0.0023	1	0.92	0.0074

Problems related to social environment	0.35	0.33	0.0036	0.37	0.36	0.0011	0.34	0.35	0.0014
Problems related to upbringing	0.2	0.2	0.0016	0.21	0.18	0.0057	0.17	0.19	0.0057
Other problems related to primary support group; including family circumstances	1.12	1.16	0.0033	1.37	1.27	0.0089	1.2	1.19	0.0015
Problems related to certain psychosocial circumstances	0.077	0.09	0.0048	0.095	0.076	0.0065	0.1	0.1	0
Problems related to other psychosocial circumstances	0.96	0.98	0.0021	1.18	1.16	0.0018	0.95	0.92	0.0025
Dietary counseling and surveillance	6.08	6.12	0.002	7.04	7.01	0.0012	6.63	6.25	0.015
Alcohol abuse counseling and surveillance	0.27	0.21	0.013	0.28	0.29	0.0024	0.26	0.22	0.0081
Drug abuse counseling and surveillance	0.07	0.07	0	0.064	0.064	0	0.08	0.08	0
Exercise counseling	0.64	0.63	0.0018	0.59	0.6	0.0017	0.66	0.63	0.004
Problems related to lifestyle	9.2	9.36	0.0055	9.63	9.61	0.00086	8.86	9.02	0.0056
Lack of physical exercise	0.11	0.097	0.0043	0.13	0.12	0.0054	0.096	0.096	0
Inappropriate diet and eating habits	0.17	0.15	0.0035	0.23	0.26	0.0064	0.25	0.16	0.019
Problems related to sleep	0.76	0.69	0.0082	0.74	0.77	0.0037	0.63	0.69	0.0069
Long term (current) drug therapy	51.29	50.85	0.0088	54.22	54.34	0.0024	51.63	50.84	0.016
Long term (current) use of insulin	28.57	28.02	0.012	32.09	32.48	0.0083	29.01	29.21	0.0044
Long term (current) use of aspirin	13.31	12.99	0.0095	14.75	14.94	0.0052	13.58	13.36	0.0065
Long term (current) use of oral hypoglycemic drugs	20.5	20.67	0.0043	21.57	21.66	0.0022	20.19	19.93	0.0066
Family history of mental and behavioral disorders	0.58	0.58	0.00092	0.65	0.65	0	0.6	0.55	0.0074
Personal history of mental and behavioral disorders	0.95	0.94	0.00072	1.07	1.02	0.0044	0.92	0.92	0
Personal history of psychological trauma; not elsewhere classified	0.2	0.2	0.0016	0.2	0.18	0.0029	0.19	0.17	0.0057
Personal history of self-harm	0.34	0.33	0.0024	0.38	0.38	0	0.36	0.39	0.0052
Bariatric surgery status	2.07	1.9	0.012	2.09	2	0.0063	1.91	1.78	0.01
Other Race	4.04	3.94	0.0053	3.73	3.66	0.0037	4.09	4.13	0.002
aspirin	37.75	37.58	0.0034	39.56	40.11	0.011	36.01	36.38	0.0078
ibuprofen	25.8	25.56	0.0054	26.73	26.57	0.0036	24.57	25.42	0.02
glucagon	17.47	17.23	0.0062	18.99	19.02	0.00081	17.13	16.86	0.0072

d) Study results, pre-COVID-19 pandemic (between 1st December 2017 and 31st December 2018 + between 1st January 2019 and 31st December 2019) and in-COVID-19 pandemic (between 1st January 2020 and 31st May 2021)

	Outcome	Pre-COVID-19		In-COVID-19	
**Semaglutide *vs***		*HR*	*p-value*	*HR*	*p-value*
**Empagliflozin (SGLT2I)**	Encephalitis	1.01	0.9921041	1.50	0.65327463
	Parkinsonism	1.07	0.86966234	1.04	0.90325192
	Cognitive deficit	0.91	0.29068865	0.91	0.17020492
	Dementia	1.21	0.39720463	1.02	0.90560248
	Epilepsy/seizure	0.97	0.86309137	0.88	0.4367128
	Migraine	0.88	0.33293643	1.00	0.97720997
	Insomnia	0.92	0.39515084	0.93	0.31670084
	Nerve disorder	0.95	0.59114187	1.04	0.59504786
	MNJ/muscle disease	0.86	0.56630392	0.60	0.00933239
	ICH	1.18	0.57102847	0.99	0.95709834
	Ischaemic stroke	1.06	0.67852935	0.88	0.25102034
	Alcohol misuse	0.84	0.37887573	0.93	0.68115791
	Opioid misuse	1.12	0.60518947	0.72	0.07799214
	Cannabis misuse	0.96	0.86677552	0.93	0.71638464
	Stimulants misuse	0.78	0.47408581	0.60	0.1266815
	Nicotine misuse	0.78	0.03113653	0.75	0.00516701
	Psychosis	0.72	0.30669487	1.02	0.93997821
	Bipolar disorder	0.99	0.96705246	0.92	0.66409776
	Depression	1.00	0.97781745	0.93	0.31888365
	Anxiety disorder	0.95	0.45580086	1.01	0.80250966
	OCD	0.61	0.38500974	0.86	0.68819463
	Suicidality	1.24	0.43609687	0.99	0.9634979
	NCOs	1.00	0.9771674	1.14	0.02314393
**Sitagliptin (DPP4I)**	Encephalitis	0.39	0.23438986	0.28	0.07672599
	Parkinsonism	0.53	0.08332607	0.92	0.80202107
	Cognitive deficit	0.70	4.47E-05	0.75	6.79E-05
	Dementia	0.72	0.11065389	0.60	0.00427259
	Epilepsy/seizure	0.79	0.23984765	0.90	0.52106567
	Migraine	0.84	0.21669664	1.03	0.81530646
	Insomnia	0.90	0.2946494	0.95	0.45478202
	Nerve disorder	0.97	0.71419701	0.98	0.80890646
	MNJ/muscle disease	0.86	0.59327857	0.75	0.20528955
	ICH	0.93	0.79936155	0.92	0.72780758
	Ischaemic stroke	0.91	0.5337035	0.79	0.04419163
	Alcohol misuse	0.92	0.69637351	0.74	0.08401265
	Opioid misuse	0.85	0.47149488	0.90	0.59780061
	Cannabis misuse	0.71	0.19681634	0.66	0.05380358

	Stimulants misuse	0.64	0.23192403	0.60	0.10303125
	Nicotine misuse	0.90	0.3653627	0.79	0.02593567
	Psychosis	0.42	0.0059871	0.70	0.12622129
	Bipolar disorder	0.73	0.2071834	0.66	0.02765459
	Depression	0.80	0.00605711	0.91	0.17162095
	Anxiety disorder	0.89	0.11971434	0.87	0.01684904
	OCD	0.29	0.03871312	1.04	0.9119564
	Suicidality	1.28	0.41244128	0.75	0.25951217
	NCOs	0.93	0.28347245	1.08	0.17992518
**Glipizide (SU)**	Encephalitis	0.97	0.97771672	0.39	0.23068124
	Parkinsonism	0.76	0.49063828	0.56	0.08109933
	Cognitive deficit	0.80	0.01972565	0.72	8.79E-06
	Dementia	0.66	0.07347358	0.57	0.00224685
	Epilepsy/seizure	0.96	0.84261577	0.75	0.07539595
	Migraine	1.38	0.04626516	1.04	0.78722679
	Insomnia	0.89	0.26595156	0.99	0.91324132
	Nerve disorder	1.06	0.59189598	0.94	0.47327209
	MNJ/muscle disease	0.83	0.48768726	0.78	0.31777472
	ICH	1.22	0.52285371	0.93	0.78886973
	Ischaemic stroke	1.07	0.65424717	0.95	0.6845328
	Alcohol misuse	0.84	0.40276505	0.70	0.06709619
	Opioid misuse	1.05	0.84313715	0.95	0.78600569
	Cannabis misuse	0.59	0.03846858	0.74	0.16974816
	Stimulants misuse	0.79	0.50903744	0.59	0.09593487
	Nicotine misuse	0.67	0.00091064	0.75	0.00860696
	Psychosis	0.76	0.36309749	0.57	0.02039893
	Bipolar disorder	0.90	0.68158801	0.92	0.70385987
	Depression	1.05	0.57078782	0.81	0.00437026
	Anxiety disorder	0.99	0.85598605	0.89	0.06916508
	OCD	0.97	0.97262953	1.55	0.27059445
	Suicidality	0.62	0.07700527	0.61	0.05271118
	NCOs	0.97	0.68907144	1.00	0.97218734

e) Study results, pooled across all years (between 1st December 2017 and 31st December 2018; between 1st January 2019 and 31st December 2019; between 1st January 2020 and 31st May 2021)

	Outcome		
**Semaglutide *vs***		*HR*	*p-value*
**Empagliflozin (SGLT2I)**	Encephalitis	1.25	0.7375377
	Parkinsonism	1.05	0.84313248
	Cognitive deficit	0.91	0.08362855
	Dementia	1.09	0.54238554
	Epilepsy/seizure	0.92	0.47762618
	Migraine	0.94	0.53315508
	Insomnia	0.93	0.18697765
	Nerve disorder	1.00	0.9338614
	MNJ/muscle disease	0.69	0.01606836
	ICH	1.05	0.77406436
	Ischaemic stroke	0.94	0.52105247
	Alcohol misuse	0.89	0.37743788
	Opioid misuse	0.86	0.3102225
	Cannabis misuse	0.94	0.69780187
	Stimulants misuse	0.68	0.11084229
	Nicotine misuse	0.77	0.00042611
	Psychosis	0.90	0.58551224
	Bipolar disorder	0.94	0.70533045
	Depression	0.96	0.45346891
	Anxiety disorder	0.99	0.78564134
	OCD	0.77	0.41271447
	Suicidality	1.09	0.6415841
	NCOs	1.08	0.07664263
**Sitagliptin (DPP4I)**	Encephalitis	0.32	0.0351418
	Parkinsonism	0.71	0.17005295
	Cognitive deficit	0.73	1.75E-08
	Dementia	0.65	0.00140122
	Epilepsy/seizure	0.86	0.21632456
	Migraine	0.94	0.52224512
	Insomnia	0.93	0.22061725
	Nerve disorder	0.97	0.68852156
	MNJ/muscle disease	0.79	0.19054214
	ICH	0.93	0.66975047
	Ischaemic stroke	0.84	0.0507092
	Alcohol misuse	0.80	0.11083025
	Opioid misuse	0.88	0.38692865
	Cannabis misuse	0.68	0.02104417

	Stimulants misuse	0.62	0.04444554
	Nicotine misuse	0.84	0.02343868
	Psychosis	0.58	0.00402006
	Bipolar disorder	0.69	0.01190808
	Depression	0.86	0.00513396
	Anxiety disorder	0.88	0.00492821
	OCD	0.74	0.31680529
	Suicidality	0.94	0.74075955
	NCOs	1.02	0.72490198
**Glipizide (SU)**	Encephalitis	0.56	0.34192258
	Parkinsonism	0.63	0.07391256
	Cognitive deficit	0.75	8.15E-07
	Dementia	0.60	0.00047675
	Epilepsy/seizure	0.82	0.13015886
	Migraine	1.17	0.13015842
	Insomnia	0.95	0.44324654
	Nerve disorder	0.99	0.83324592
	MNJ/muscle disease	0.81	0.2313466
	ICH	1.04	0.82999701
	Ischaemic stroke	1.00	0.99499276
	Alcohol misuse	0.76	0.05610346
	Opioid misuse	0.99	0.94039849
	Cannabis misuse	0.67	0.01693203
	Stimulants misuse	0.67	0.09209631
	Nicotine misuse	0.72	3.18E-05
	Psychosis	0.64	0.01781894
	Bipolar disorder	0.91	0.58112304
	Depression	0.90	0.06622817
	Anxiety disorder	0.93	0.13043347
	OCD	1.42	0.32709696
	Suicidality	0.61	0.00884853
	NCOs	0.99	0.84234809

Supplementary Table S8. Secondary analysis, 2-year follow-up

	Semaglutide vs Sitagliptin		Semaglutide vs Empagliflozin		Semaglutide vs Glipizide	
**Outcome**	*HR*	*p*	*HR*	*p*	*HR*	*p*
Alcohol (any)	0.83 (0.72-0.95)	0.0066	0.85 (0.74-0.98)	0.021	0.80 (0.69-0.94)	0.0058
Alcohol (first)	0.84 (0.68-1.04)	0.12	0.90 (0.72-1.12)	0.35	0.88 (0.69-1.12)	0.28
All-cause mortality (first)	0.67 (0.61-0.74)	8.70E-16	0.93 (0.84-1.03)	0.15	0.65 (0.58-0.72)	1.30E-15
Anxiety disorder (any)	0.98 (0.94-1.02)	0.25	0.99 (0.96-1.03)	0.76	1.04 (1.00-1.08)	0.081
Anxiety disorder (first)	0.98 (0.91-1.05)	0.53	1.01 (0.94-1.08)	0.82	1.03 (0.95-1.11)	0.45
Any NCOs (any)	0.97 (0.93-1.02)	0.25	1.03 (0.98-1.08)	0.2	1.06 (1.01-1.12)	0.021
Any NCOs (first)	0.96 (0.90-1.02)	0.2	1.01 (0.95-1.08)	0.69	1.01 (0.94-1.09)	0.71
Bipolar disorder (any)	0.93 (0.83-1.04)	0.2	0.90 (0.80-1.02)	0.093	0.91 (0.80-1.03)	0.15
Bipolar disorder (first)	0.76 (0.60-0.95)	0.017	1.00 (0.77-1.30)	0.99	0.84 (0.65-1.09)	0.19
Cannabis (any)	0.74 (0.61-0.89)	0.0013	0.88 (0.73-1.07)	0.21	0.95 (0.78-1.17)	0.66
Cannabis (first)	0.75 (0.59-0.94)	0.012	0.91 (0.71-1.18)	0.49	0.88 (0.68-1.15)	0.35
Cognitive deficit (first)	0.80 (0.74-0.86)	1.60E-08	0.98 (0.90-1.06)	0.57	0.81 (0.74-0.88)	2.90E-06
Cognitive deficits (any)	0.85 (0.80-0.90)	4.40E-08	1.02 (0.96-1.08)	0.54	0.87 (0.81-0.93)	3.40E-05
Dementia (any)	0.72 (0.61-0.84)	3.20E-05	1.02 (0.87-1.19)	0.8	0.73 (0.61-0.87)	4.00E-04
Dementia (first)	0.58 (0.47-0.70)	3.80E-08	1.01 (0.82-1.24)	0.92	0.67 (0.53-0.84)	0.00044
Depressive disorder (any)	0.94 (0.90-0.97)	0.0011	1.01 (0.97-1.05)	0.71	1.01 (0.96-1.05)	0.73
Depressive disorder (first)	0.88 (0.81-0.95)	0.0016	0.92 (0.85-1.00)	0.063	0.95 (0.87-1.04)	0.23
Encephalitis (any)	0.44 (0.23-0.84)	0.0095	0.55 (0.26-1.14)	0.098	0.40 (0.18-0.86)	0.014
Encephalitis (first)	0.29 (0.13-0.68)	0.0019	0.53 (0.21-1.33)	0.16	0.24 (0.08-0.72)	0.0043
Epilepsy/seizure (any)	0.89 (0.79-1.00)	0.042	1.08 (0.95-1.21)	0.24	0.95 (0.84-1.08)	0.45
Epilepsy/seizure (first)	0.79 (0.66-0.95)	0.012	0.91 (0.75-1.10)	0.33	0.87 (0.71-1.08)	0.21
Insomnia (any)	0.87 (0.82-0.93)	4.80E-06	0.93 (0.88-0.99)	0.021	0.94 (0.88-1.00)	0.055
Insomnia (first)	0.91 (0.83-0.99)	0.035	0.94 (0.86-1.03)	0.18	1.01 (0.91-1.11)	0.91
Intracranial haemorrhage (any)	0.76 (0.61-0.94)	0.012	0.81 (0.65-1.03)	0.08	0.86 (0.67-1.11)	0.25
Intracranial haemorrhage (first)	0.80 (0.61-1.06)	0.12	0.79 (0.60-1.04)	0.097	0.87 (0.64-1.19)	0.38

Ischaemic stroke (any)	0.90 (0.83-0.99)	0.026	0.99 (0.91-1.09)	0.89	1.03 (0.93-1.14)	0.51
Ischaemic stroke (first)	0.81 (0.71-0.93)	0.0022	1.01 (0.88-1.16)	0.89	0.91 (0.78-1.07)	0.26
Migraine (any)	1.01 (0.93-1.10)	0.77	1.04 (0.96-1.12)	0.39	1.20 (1.10-1.32)	4.20E-05
Migraine (first)	1.03 (0.89-1.19)	0.7	1.01 (0.87-1.17)	0.88	1.19 (1.01-1.39)	0.038
Myoneural junction/muscle disorder (any)	1.01 (0.83-1.23)	0.89	0.79 (0.65-0.97)	0.021	0.89 (0.71-1.12)	0.31
Myoneural junction/muscle disorder (first)	0.90 (0.71-1.15)	0.41	0.82 (0.64-1.05)	0.12	0.92 (0.69-1.23)	0.59
Nerve/nerve root/plexus disorder (any)	1.03 (0.96-1.10)	0.45	1.03 (0.96-1.10)	0.38	1.04 (0.96-1.12)	0.32
Nerve/nerve root/plexus disorder (first)	0.96 (0.87-1.05)	0.35	1.00 (0.91-1.10)	0.94	1.00 (0.91-1.11)	0.95
Nicotine (any)	0.89 (0.84-0.94)	4.00E-05	0.88 (0.83-0.93)	9.70E-06	0.89 (0.84-0.95)	2.00E-04
Nicotine (first)	0.83 (0.74-0.94)	0.0021	0.82 (0.72-0.92)	0.0012	0.80 (0.70-0.91)	0.00085
Obsessive-compulsive disorder (any)	0.75 (0.55-1.01)	0.059	0.91 (0.66-1.25)	0.55	0.66 (0.47-0.95)	0.023
Obsessive-compulsive disorder (first)	0.68 (0.44-1.05)	0.08	1.05 (0.66-1.68)	0.83	0.91 (0.55-1.51)	0.71
Opioid (any)	0.99 (0.85-1.16)	0.92	0.97 (0.82-1.13)	0.67	0.90 (0.76-1.08)	0.26
Opioid (first)	0.93 (0.75-1.15)	0.48	1.06 (0.85-1.33)	0.59	0.86 (0.68-1.08)	0.2
Parkinsonism (any)	0.74 (0.58-0.94)	0.013	0.99 (0.77-1.26)	0.92	0.79 (0.61-1.03)	0.086
Parkinsonism (first)	0.63 (0.44-0.91)	0.012	1.01 (0.69-1.49)	0.94	0.71 (0.48-1.05)	0.083
Schizophrenia spectrum disorder (any)	0.81 (0.69-0.94)	0.0075	0.92 (0.78-1.08)	0.3	0.74 (0.63-0.88)	0.00075
Schizophrenia spectrum disorder (first)	0.60 (0.45-0.80)	0.00032	0.86 (0.64-1.17)	0.34	0.59 (0.43-0.80)	0.00056
Stimulants (any)	0.86 (0.69-1.07)	0.17	0.76 (0.61-0.96)	0.019	0.75 (0.60-0.95)	0.015
Stimulants (first)	0.75 (0.54-1.06)	0.11	0.67 (0.47-0.97)	0.032	0.72 (0.51-1.01)	0.058
Suicidal ideation (any)	0.82 (0.66-1.01)	0.059	1.15 (0.92-1.44)	0.21	0.70 (0.55-0.88)	0.002
Suicidal ideation (first)	0.85 (0.66-1.11)	0.24	1.15 (0.87-1.52)	0.33	0.77 (0.58-1.04)	0.091

Supplementary Table S9. Tests of the proportionality assumption (p-values)

Outcome	Semaglutide vs Sitagliptin	Semaglutide vs Empagliflozin	Semaglutide vs Glipizide
Encephalitis (first)	0.47	0.87	0.95
Parkinsonism (first)	0.79	0.45	0.58
Cognitive deficit (first)	0.03	0.09	0.42
Dementia (first)	0.80	0.07	0.42
Epilepsy/seizure (first)	0.15	0.77	0.07
Migraine (first)	0.18	0.55	0.61
Insomnia (first)	0.11	0.18	0.37
Nerve disorder (first)	0.14	0.49	0.22
MNJ/muscle disease (first)	0.79	0.11	0.65
ICH (first)	0.41	0.67	0.83
Ischaemic stroke (first)	0.26	0.98	0.45
Alcohol misuse (first)	0.41	0.33	0.13
Opioid misuse (first)	0.99	0.03	0.69
Cannabis misuse (first)	0.63	0.43	0.64
Stimulants misuse (first)	0.37	0.89	0.14
Nicotine misuse (first)	0.18	0.79	0.03
Psychosis (first)	0.18	0.40	0.91
Bipolar disorder (first)	0.57	0.38	0.39
Depression (first)	0.09	0.67	0.12
Anxiety disorder (first)	0.01	<0.01	<0.01
OCD (first)	0.72	0.85	0.89
Suicidality (first)	0.57	0.14	0.99
NCOs (first)	0.11	0.32	0.16
Encephalitis (any)	0.30	0.59	0.65
Cognitive deficit (any)	0.01	0.14	0.28
Epilepsy/seizure (any)	0.03	0.98	<0.01
Migraine (any)	<0.01	0.13	0.04
Insomnia (any)	0.45	0.12	0.82
Nerve disorder (any)	0.50	0.50	0.75
ICH (any)	0.31	0.97	0.20
Ischaemic stroke (any)	0.02	0.29	<0.01
Alcohol misuse (any)	0.57	0.36	0.61
Opioid misuse (any)	0.93	0.04	0.22
Cannabis misuse (any)	0.94	0.60	0.36
Stimulants misuse (any)	0.46	0.65	0.80
Nicotine misuse (any)	0.62	0.19	0.33
Psychosis (any)	0.62	0.37	0.20
Bipolar disorder (any)	0.53	0.70	0.25
Depression (any)	0.24	0.70	0.41

Anxiety disorder (any)	0.77	0.67	0.89
OCD (any)	0.98	0.61	0.79
Suicidality (any)	0.44	0.50	0.25
NCOs (any)	0.03	0.60	0.38
All-cause mortality	<0.01	<0.01	<0.01

Numbers represent p-values. ICH: intracranial haemorrhage, MNJ: myoneural junction, NCOs: negative control outcomes, OCD: obsessive-compulsive disorder.

SUPPLEMENTARY FIGURES

Supplementary Figure S1. Negative logarithm of the p-values for the risks of neurological, psychiatric, and negative control outcomes (any diagnosis) in the year after semaglutide compared to three other antidiabetic medications

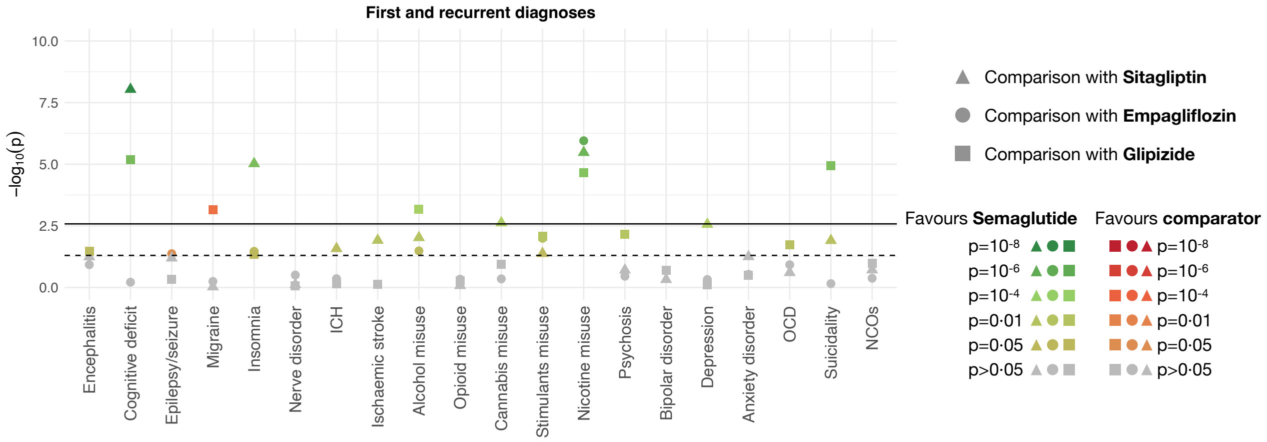

The horizontal solid line represents the threshold (p <0·0023) above which associations are statistically significant after Bonferroni correction. The dashed line represents the threshold above which associations are statistically significant at the nominal p <0·05 level. Colour shades encode the p-values with green shades favouring semaglutide and red shades favouring the comparator. ICH: intracranial haemorrhage; MNJ: myoneural junction; NCOs: composite negative control outcomes; OCD: obsessive-compulsive disorder.

Supplementary Figure S2. Negative logarithm of the p-values for the risks of neurological, psychiatric, and negative control outcomes (first diagnosis) for <65yo and ≥ 65yo in the year after semaglutide compared to three other antidiabetic medications

a) <65yo

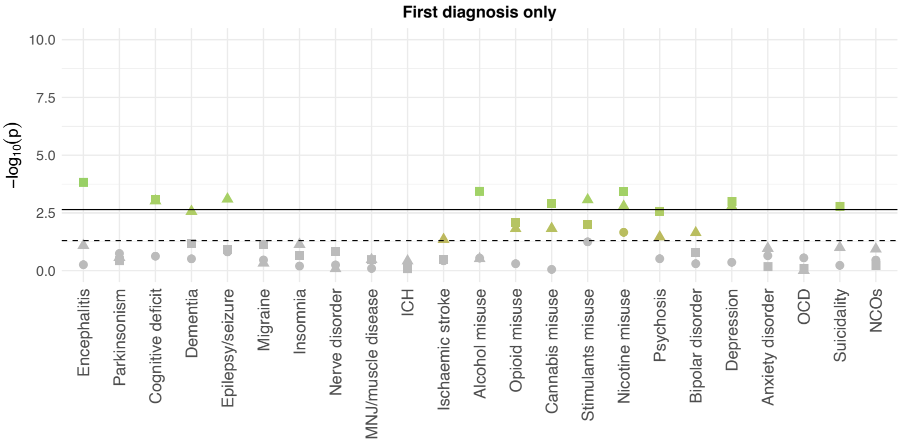

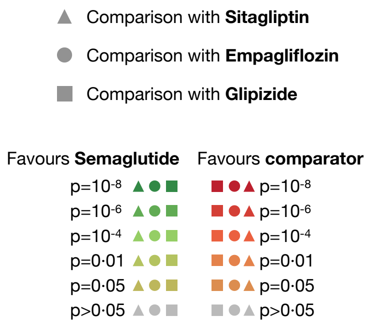

b) ≥65yo

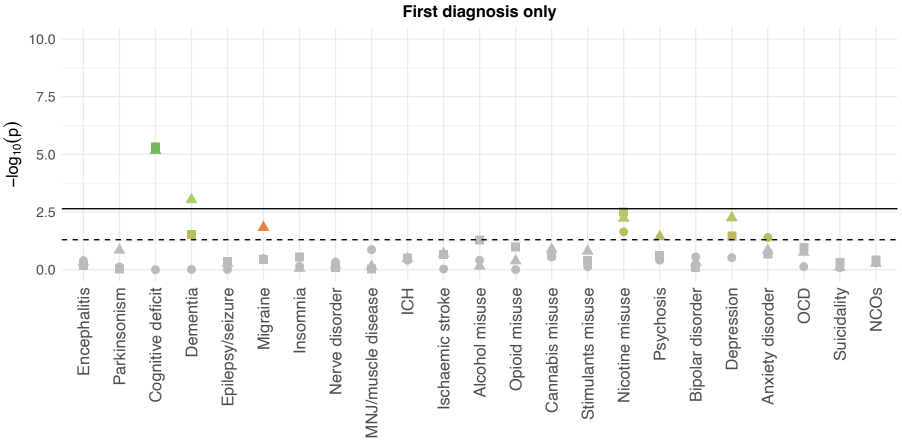

The horizontal solid line represents the threshold (p <0·0023) above which associations are statistically significant after Bonferroni correction. The dashed line represents the threshold above which associations are statistically significant at the nominal p <0·05 level. Colour shades encode the p-values with green shades favouring semaglutide and red shades favouring the comparator. Because sample sizes are smaller than in the primary analyses, the p-values should not be directly compared with those from the primary analyses. Baseline characteristics and details of all HRs and p-values can be found in Supplementary Tables S5. ICH: intracranial haemorrhage; MNJ: myoneural junction; NCOs: composite negative control outcomes; OCD: obsessive-compulsive disorder.

Supplementary Figure S3. Negative logarithm of the p-values for the composite risks of death in addition to neurological and psychiatric outcomes (first diagnosis) in the year after semaglutide compared to three other antidiabetic medications

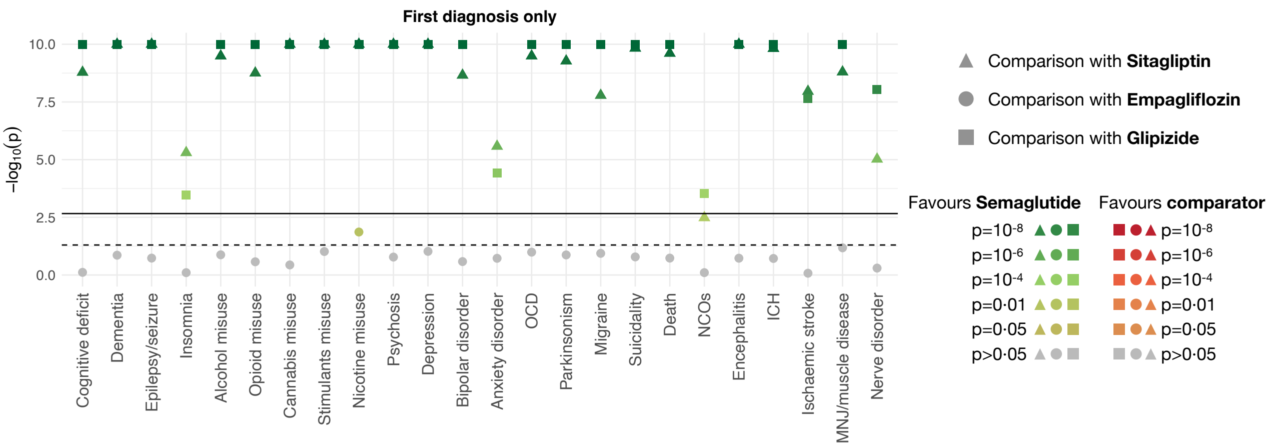

The horizontal solid line represents the threshold (p <0·0023) above which associations are statistically significant after Bonferroni correction. The dashed line represents the threshold above which associations are statistically significant at the nominal p <0·05 level. Colour shades encode the p-values with green shades favouring semaglutide and red shades favouring the comparator. All neurological and psychiatric outcomes are to be considered as composite in addition to death. Baseline characteristics are the same as the main cohorts (Supplementary Tables S1-S2), details of all HRs and p-values can be found in Supplementary Table S6. ICH: intracranial haemorrhage; MNJ: myoneural junction; NCOs: composite negative control outcomes; OCD: obsessive-compulsive disorder.

Supplementary Figure S4. Negative logarithm of the p-values for the risks of neurological, psychiatric, and negative control outcomes (first diagnosis) in the year after semaglutide compared to three other antidiabetic medications, adjusted for time of the index event (using pooling of results stratified in three time windows: between 1st December 2017 and 31st December 2018; between 1st January 2019 and 31st December 2019; between 1st January 2020 and 31st May 2021).

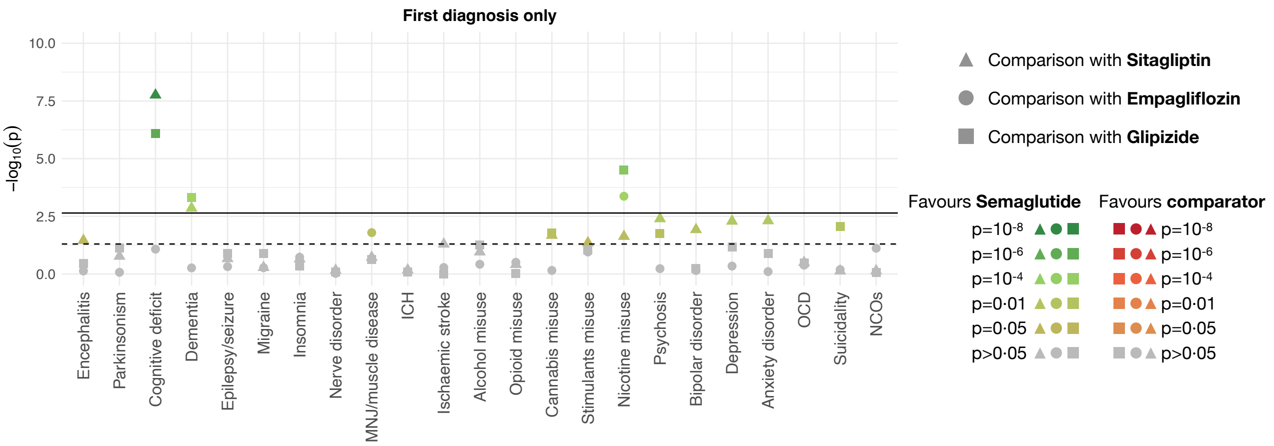

The horizontal solid line represents the threshold (p <0·0023) above which associations are statistically significant after Bonferroni correction. The dashed line represents the threshold above which associations are statistically significant at the nominal p <0·05 level. Colour shades encode the p-values with green shades favouring semaglutide and red shades favouring the comparator. Baseline characteristics and details of all HRs and p-values can be found in Supplementary Tables S7. ICH: intracranial haemorrhage; MNJ: myoneural junction; NCOs: composite negative control outcomes; OCD: obsessive-compulsive disorder.

Supplementary Figure S5. Negative logarithm of the p-values for the risks of neurological, psychiatric, and negative control outcomes (first diagnosis) *in the two years* after semaglutide compared to three other antidiabetic medications.

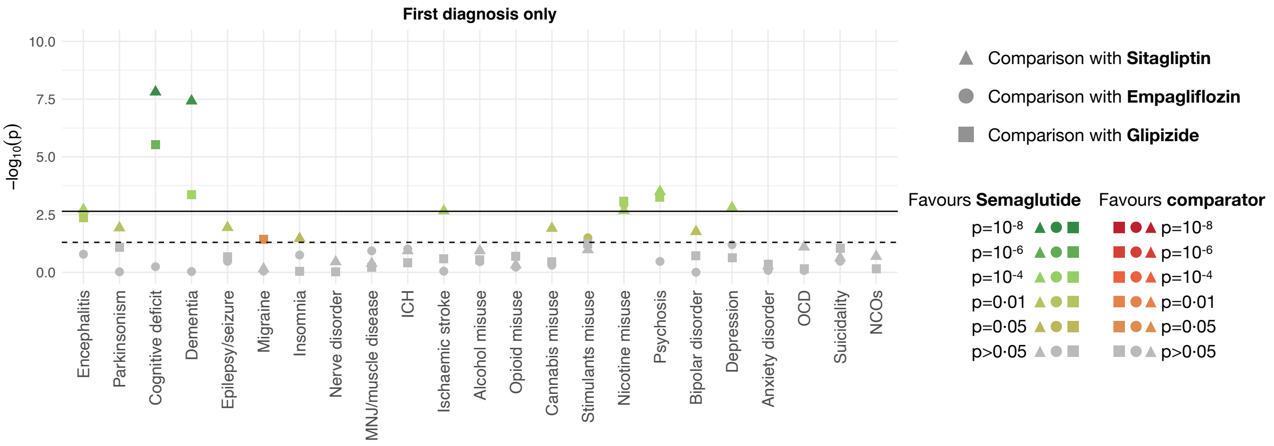

The horizontal solid line represents the threshold (p <0·0023) above which associations are statistically significant after Bonferroni correction. The dashed line represents the threshold above which associations are statistically significant at the nominal p <0·05 level. Colour shades encode the p-values with green shades favouring semaglutide and red shades favouring the comparator. Baseline characteristics are the same as the main cohorts (Supplementary Tables S1-S2), details of all HRs and p-values can be found in Supplementary Table S8. ICH: intracranial haemorrhage; MNJ: myoneural junction; NCOs: composite negative control outcomes; OCD: obsessive-compulsive disorder.

Supplementary Figure S6. Kaplan-Meier estimates for all outcomes across comparisons.

Shaded areas are 95% confidence intervals. For diagnostic subcategories of cognitive deficit, see the appendix, p 44. DPP4I: dipeptidyl peptidase 4 inhibitor, SGLT2I: sodium-glucose transporter 2 inhibitor, SU: sulphonylurea.

a) Semaglutide vs Sitagliptin

First diagnosis

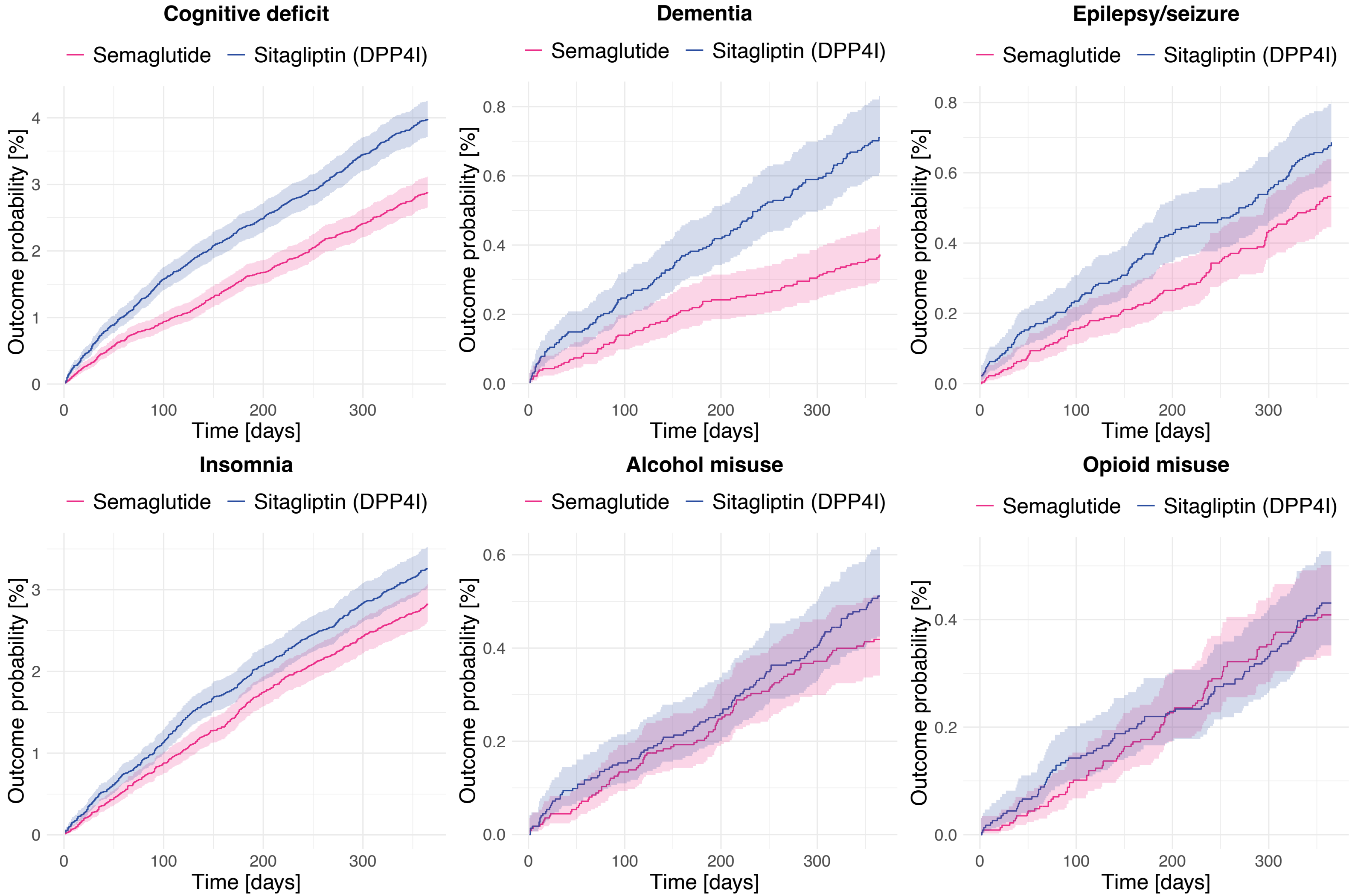

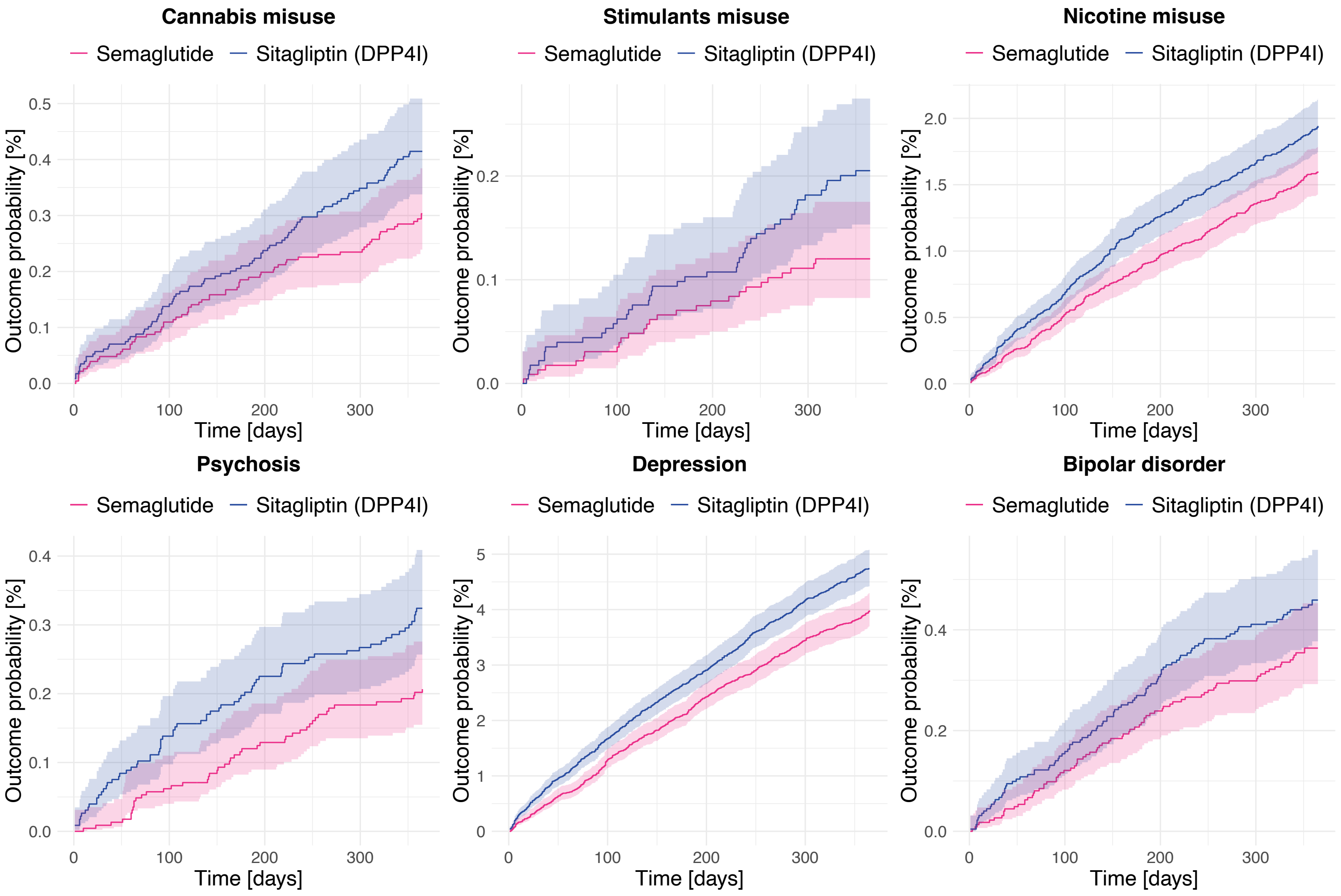

Cannabis misuse
Semaglutide
Sitagliptin (DPP4I)
Outcome probability [%]
0.0
0.1
0.2
0.3
0.4
0.5
0
100
200
300
Time [days]
Stimulants misuse
Semaglutide
Sitagliptin (DPP4I)
Outcome probability [%]
0.0
0.1
0.2
0
100
200
300
Time [days]
Nicotine misuse
Semaglutide
Sitagliptin (DPP4I)
Outcome probability [%]
0.0
0.5
1.0
1.5
2.0
0
100
200
300
Time [days]
Psychosis
Semaglutide
Sitagliptin (DPP4I)
Outcome probability [%]
0.0
0.1
0.2
0.3
0.4
0
100
200
300
Time [days]
Depression
Semaglutide
Sitagliptin (DPP4I)
Outcome probability [%]
0
1
2
3
4
5
0
100
200
300
Time [days]
Bipolar disorder
Semaglutide
Sitagliptin (DPP4I)
Outcome probability [%]
0.0
0.2
0.4
0
100
200
300
Time [days]

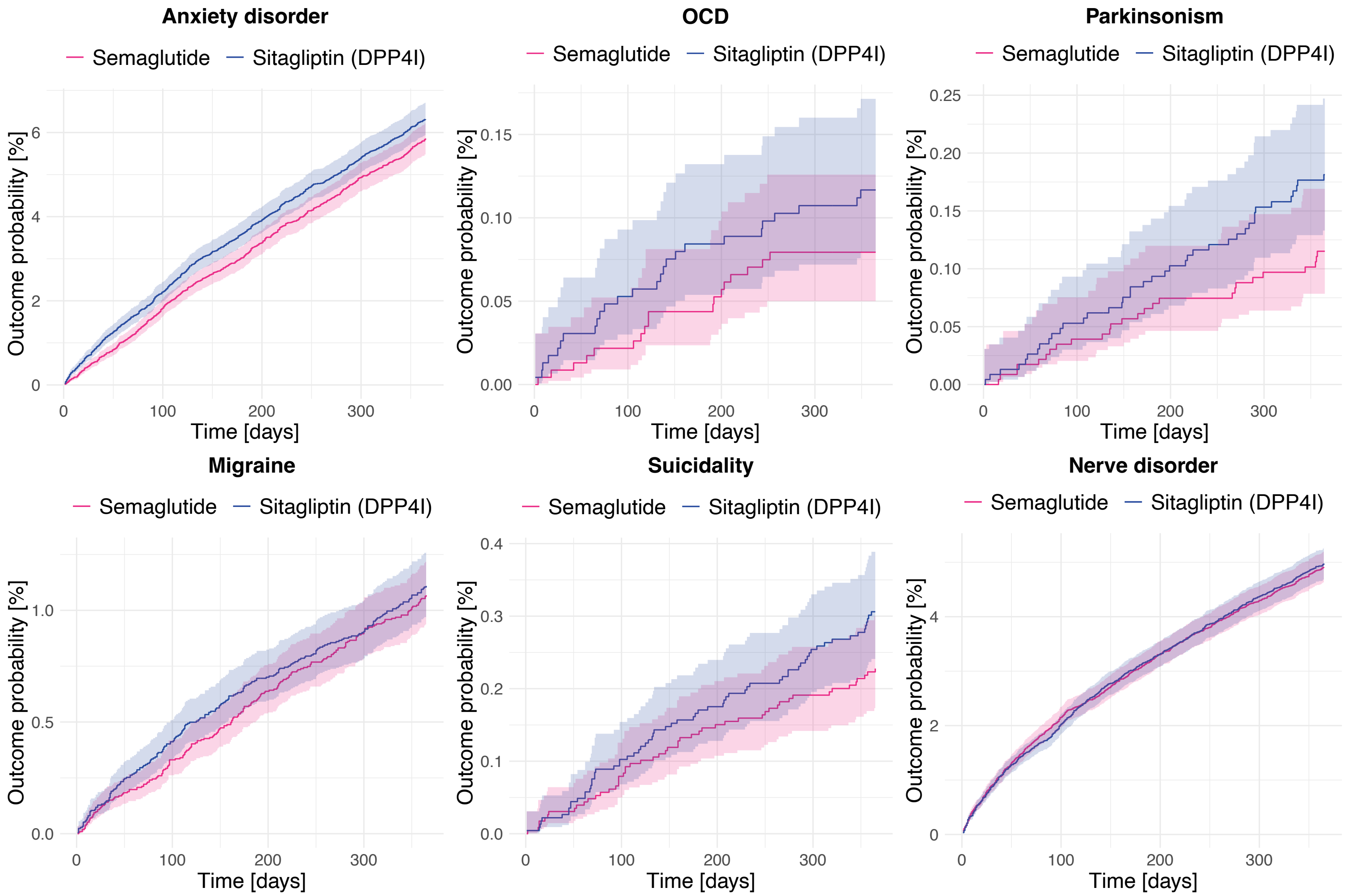

Anxiety disorder
Semaglutide
Sitagliptin (DPP4I)
Outcome probability [%]
Time [days]
OCD
Semaglutide
Sitagliptin (DPP4I)
Outcome probability [%]
Time [days]
Parkinsonism
Semaglutide
Sitagliptin (DPP4I)
Outcome probability [%]
Time [days]
Migraine
Semaglutide
Sitagliptin (DPP4I)
Outcome probability [%]
Time [days]
Suicidality
Semaglutide
Sitagliptin (DPP4I)
Outcome probability [%]
Time [days]
Nerve disorder
Semaglutide
Sitagliptin (DPP4I)
Outcome probability [%]
Time [days]

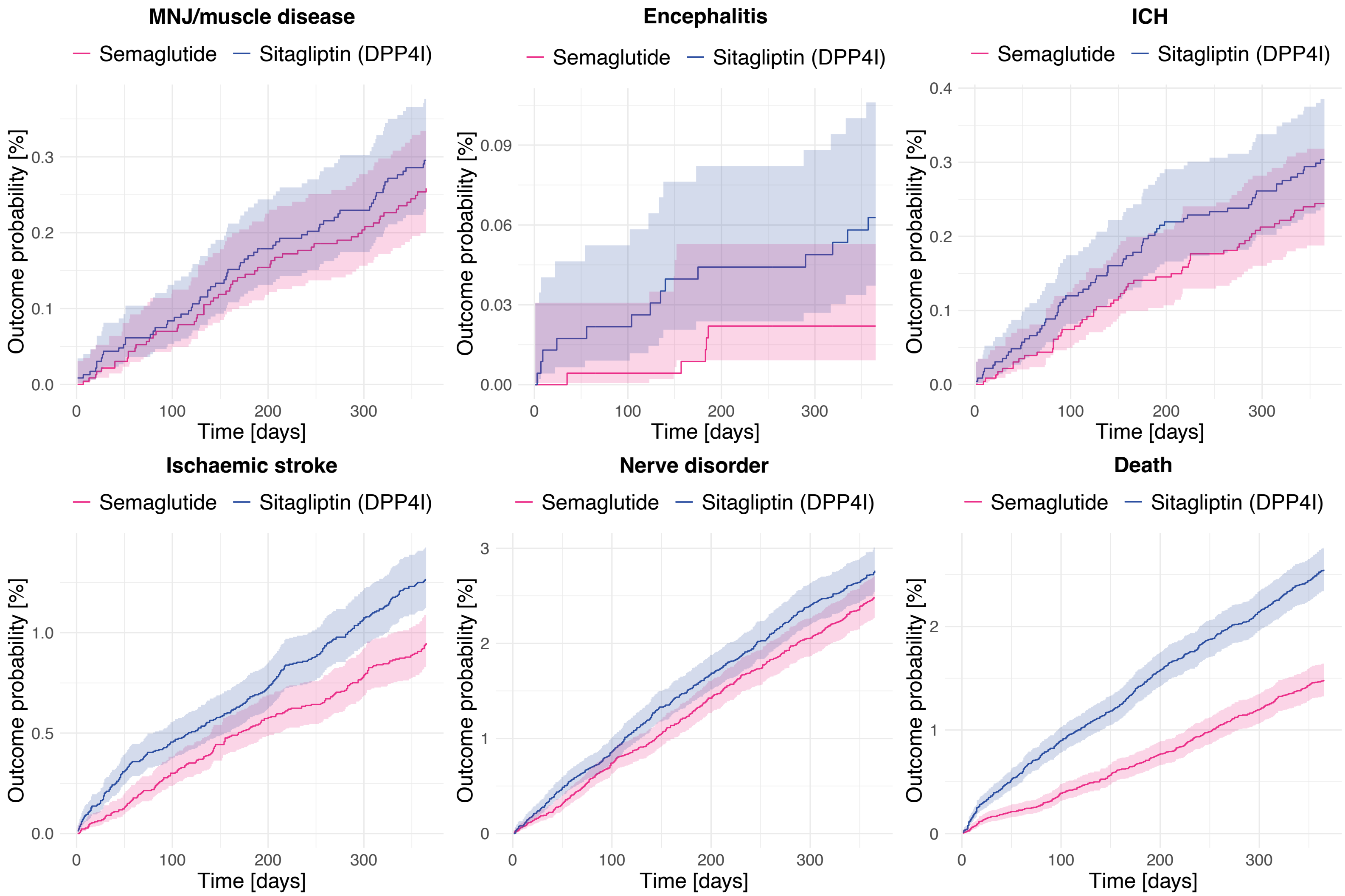

MNJ/muscle disease
Encephalitis
ICH
Ischaemic stroke
Nerve disorder
Death
Semaglutide
Sitagliptin (DPP4I)
Outcome probability [%]
Time [days]
0
100
200
300
0.0
0.1
0.2
0.3
0.00
0.03
0.06
0.09
0.4
0.5
1.0
1
2
3

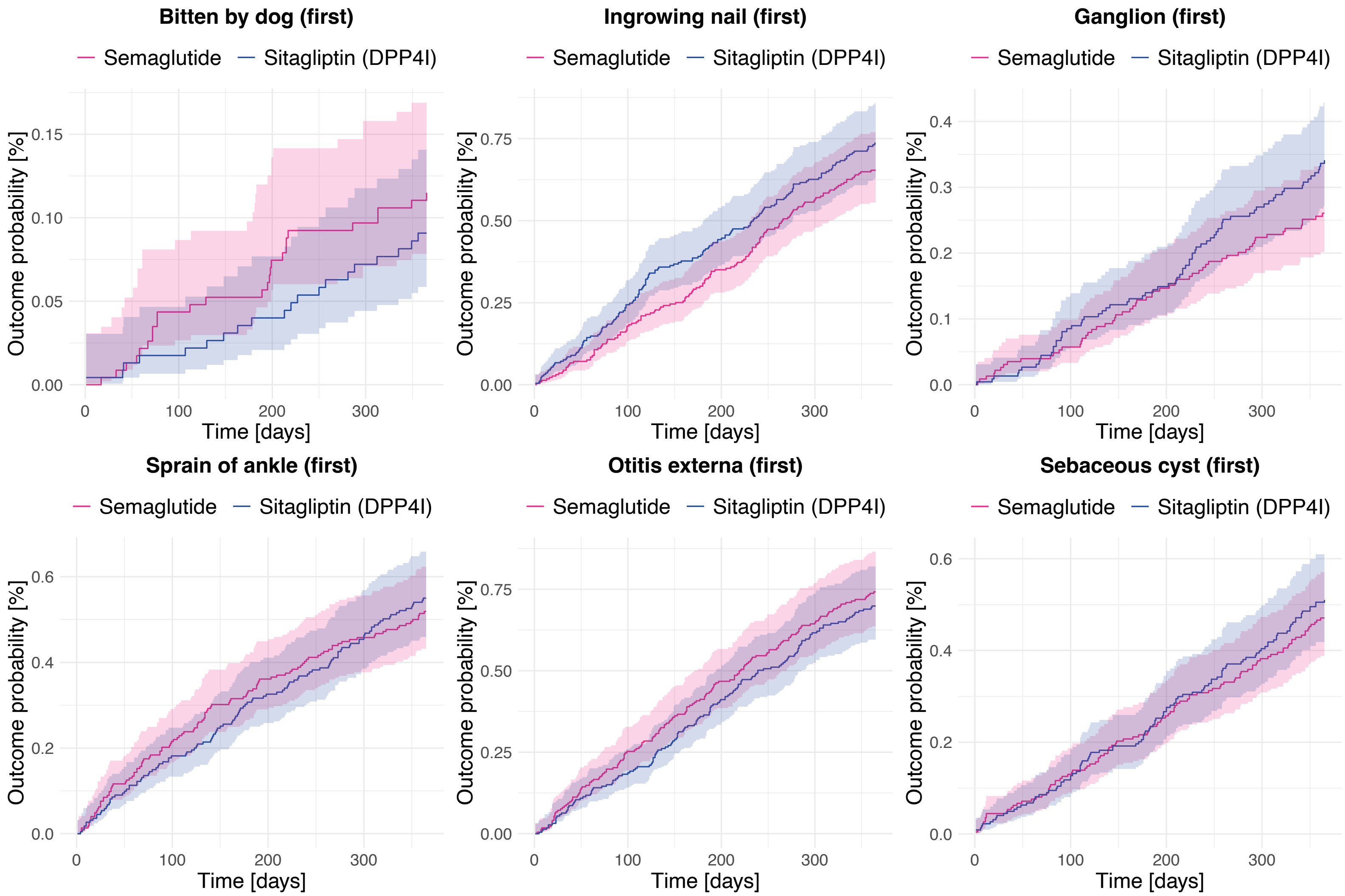
Bitten by dog (first)
Semaglutide
Sitagliptin (DPP4I)
Outcome probability [%]
0.15
0.10
0.05
0.00
0
100
200
300
Time [days]
Ingrowing nail (first)
Semaglutide
Sitagliptin (DPP4I)
Outcome probability [%]
0.75
0.50
0.25
0.00
0
100
200
300
Time [days]
Ganglion (first)
Semaglutide
Sitagliptin (DPP4I)
Outcome probability [%]
0.4
0.3
0.2
0.1
0.0
0
100
200
300
Time [days]
Sprain of ankle (first)
Semaglutide
Sitagliptin (DPP4I)
Outcome probability [%]
0.6
0.4
0.2
0.0
0
100
200
300
Time [days]
Otitis externa (first)
Semaglutide
Sitagliptin (DPP4I)
Outcome probability [%]
0.75
0.50
0.25
0.00
0
100
200
300
Time [days]
Sebaceous cyst (first)
Semaglutide
Sitagliptin (DPP4I)
Outcome probability [%]
0.6
0.4
0.2
0.0
0
100
200
300
Time [days]

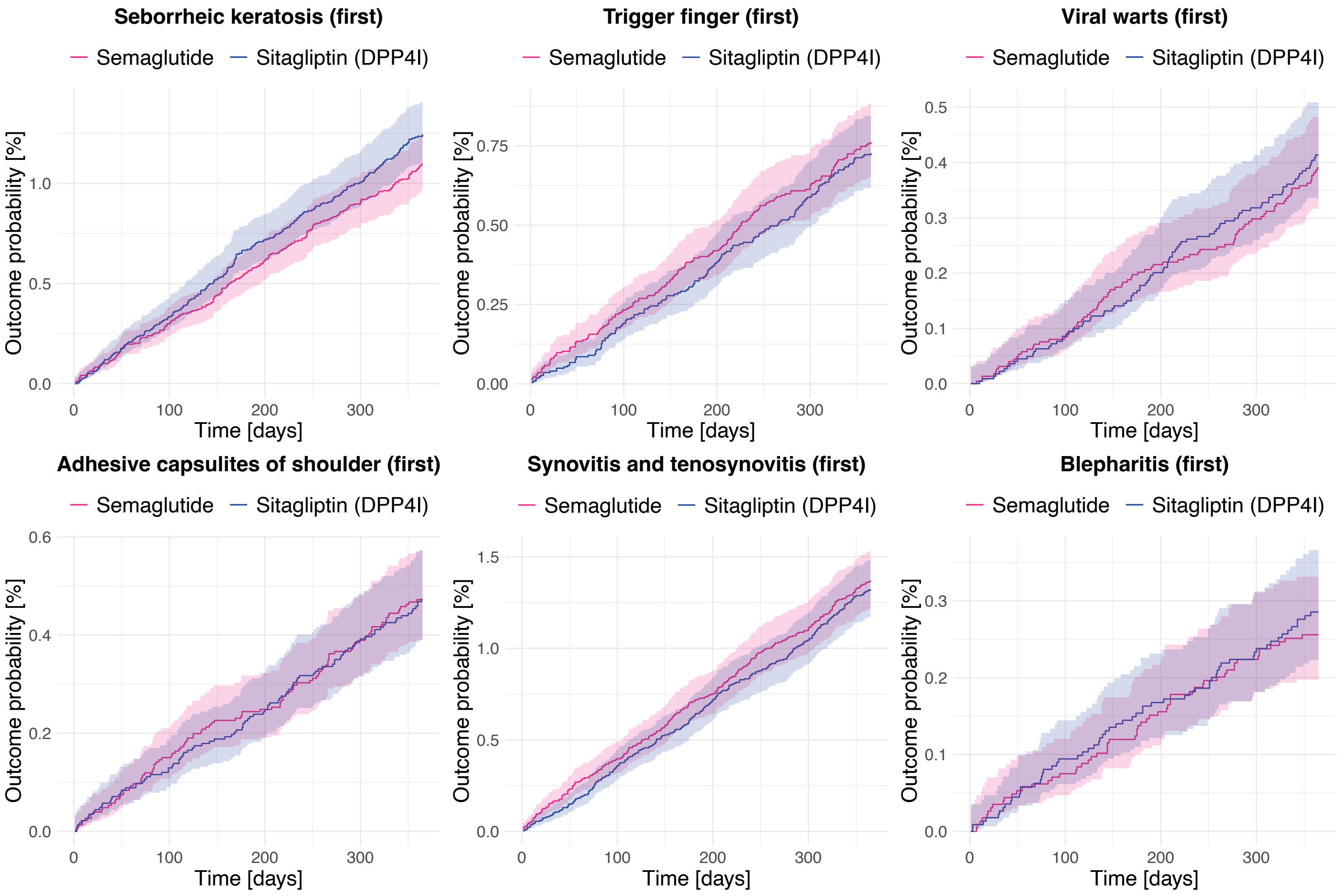
Seborrheic keratosis (first)
Semaglutide
Sitagliptin (DPP4I)
Outcome probability [%]
0.0
0.5
1.0
0
100
200
300
Time [days]
Trigger finger (first)
Semaglutide
Sitagliptin (DPP4I)
Outcome probability [%]
0.00
0.25
0.50
0.75
0
100
200
300
Time [days]
Viral warts (first)
Semaglutide
Sitagliptin (DPP4I)
Outcome probability [%]
0.0
0.1
0.2
0.3
0.4
0.5
0
100
200
300
Time [days]
Adhesive capsulites of shoulder (first)
Semaglutide
Sitagliptin (DPP4I)
Outcome probability [%]
0.0
0.2
0.4
0.6
0
100
200
300
Time [days]
Synovitis and tenosynovitis (first)
Semaglutide
Sitagliptin (DPP4I)
Outcome probability [%]
0.0
0.5
1.0
1.5
0
100
200
300
Time [days]
Blepharitis (first)
Semaglutide
Sitagliptin (DPP4I)
Outcome probability [%]
0.0
0.1
0.2
0.3
0
100
200
300
Time [days]

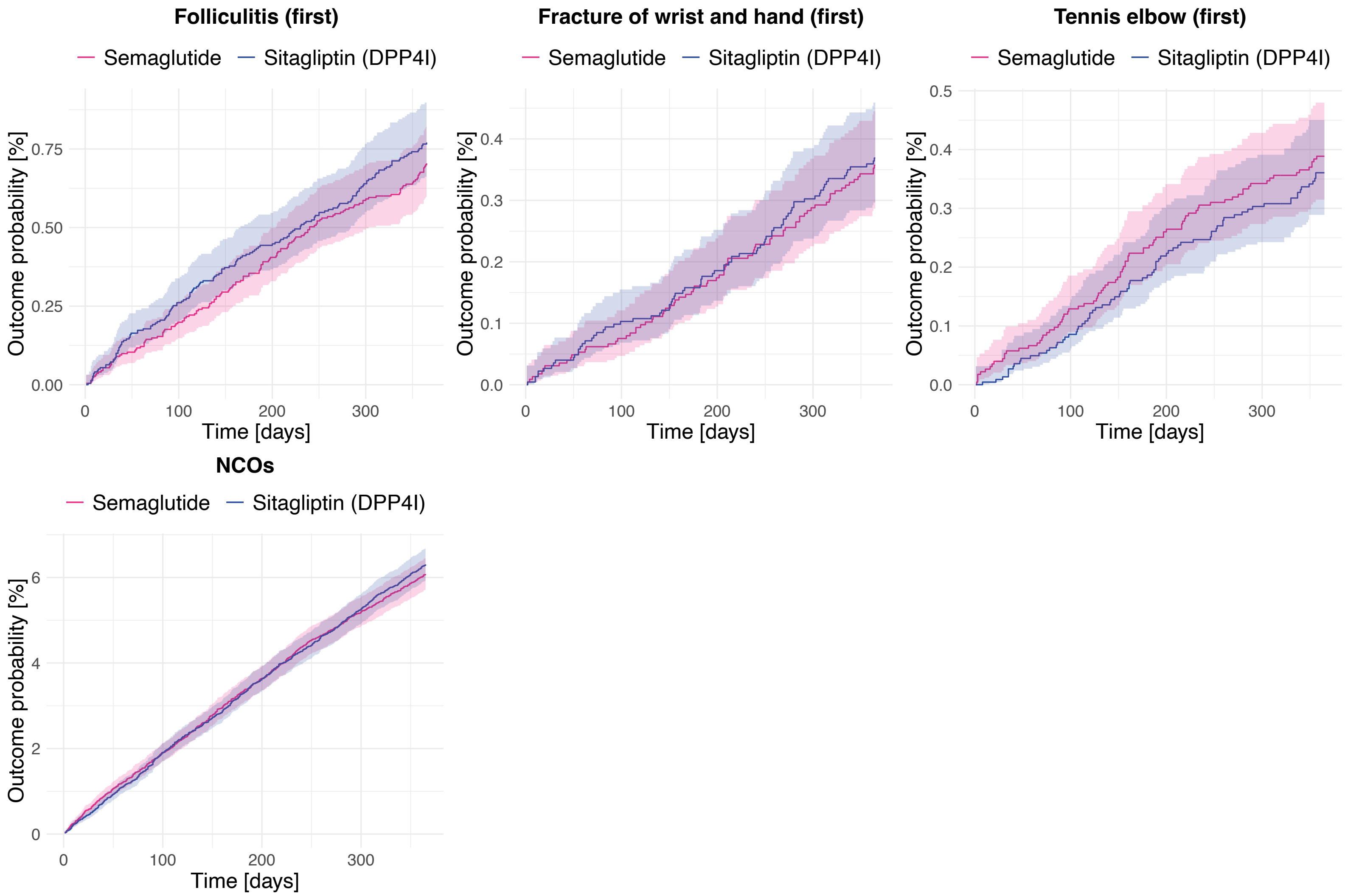

Any diagnosis

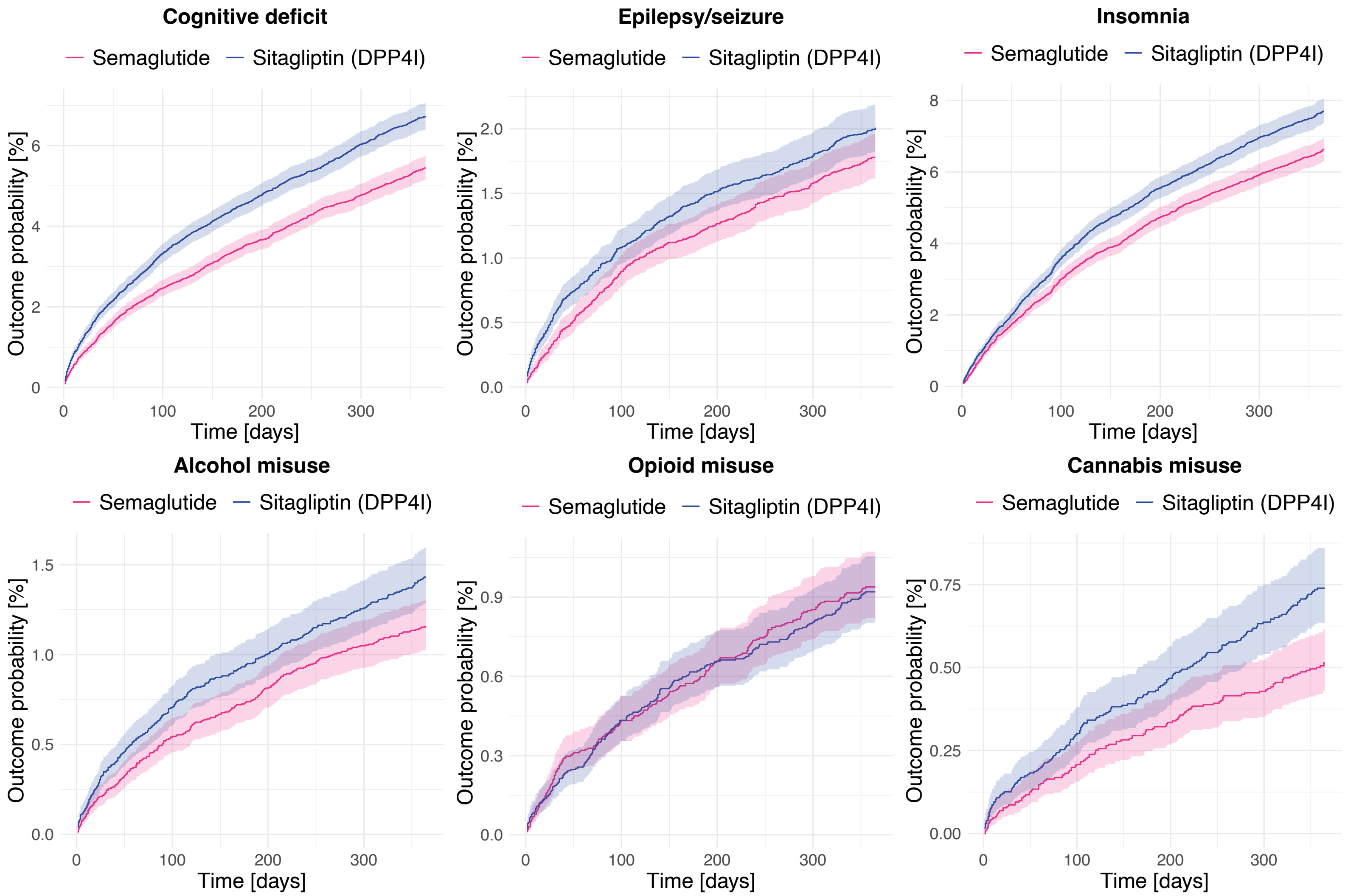
Cognitive deficit
Semaglutide
Sitagliptin (DPP4I)
Outcome probability [%]
0
2
4
6
0
100
200
300
Time [days]
Epilepsy/seizure
Semaglutide
Sitagliptin (DPP4I)
Outcome probability [%]
0.0
0.5
1.0
1.5
2.0
0
100
200
300
Time [days]
Insomnia
Semaglutide
Sitagliptin (DPP4I)
Outcome probability [%]
0
2
4
6
8
0
100
200
300
Time [days]
Alcohol misuse
Semaglutide
Sitagliptin (DPP4I)
Outcome probability [%]
0.0
0.5
1.0
1.5
0
100
200
300
Time [days]
Opioid misuse
Semaglutide
Sitagliptin (DPP4I)
Outcome probability [%]
0.0
0.3
0.6
0.9
0
100
200
300
Time [days]
Cannabis misuse
Semaglutide
Sitagliptin (DPP4I)
Outcome probability [%]
0.00
0.25
0.50
0.75
0
100
200
300
Time [days]

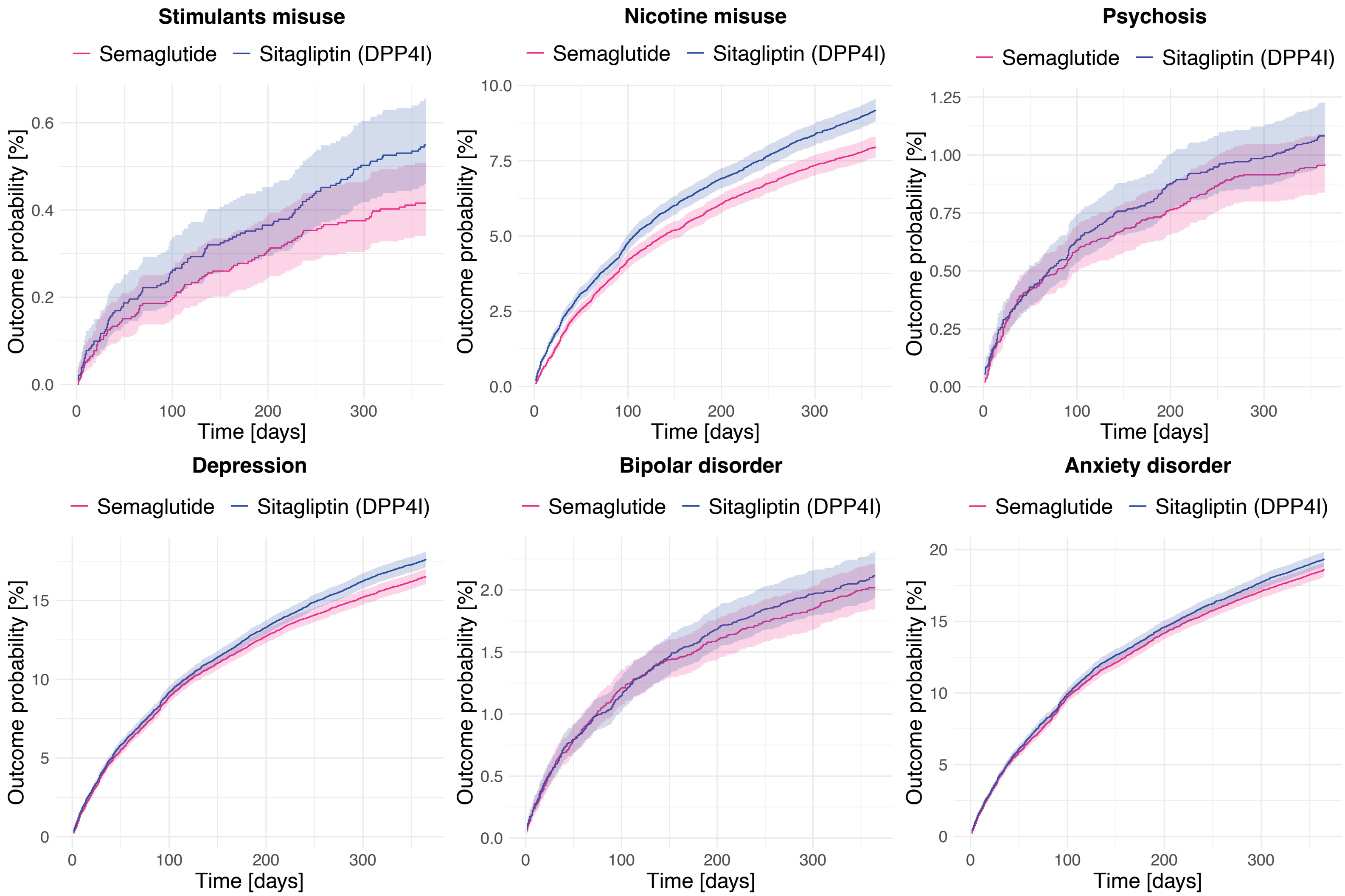

Stimulants misuse
Semaglutide
Sitagliptin (DPP4I)
Outcome probability [%]
0.0
0.2
0.4
0.6
0
100
200
300
Time [days]
Nicotine misuse
Semaglutide
Sitagliptin (DPP4I)
Outcome probability [%]
0.0
2.5
5.0
7.5
10.0
0
100
200
300
Time [days]
Psychosis
Semaglutide
Sitagliptin (DPP4I)
Outcome probability [%]
0.00
0.25
0.50
0.75
1.00
1.25
0
100
200
300
Time [days]
Depression
Semaglutide
Sitagliptin (DPP4I)
Outcome probability [%]
0
5
10
15
0
100
200
300
Time [days]
Bipolar disorder
Semaglutide
Sitagliptin (DPP4I)
Outcome probability [%]
0.0
0.5
1.0
1.5
2.0
0
100
200
300
Time [days]
Anxiety disorder
Semaglutide
Sitagliptin (DPP4I)
Outcome probability [%]
0
5
10
15
20
0
100
200
300
Time [days]

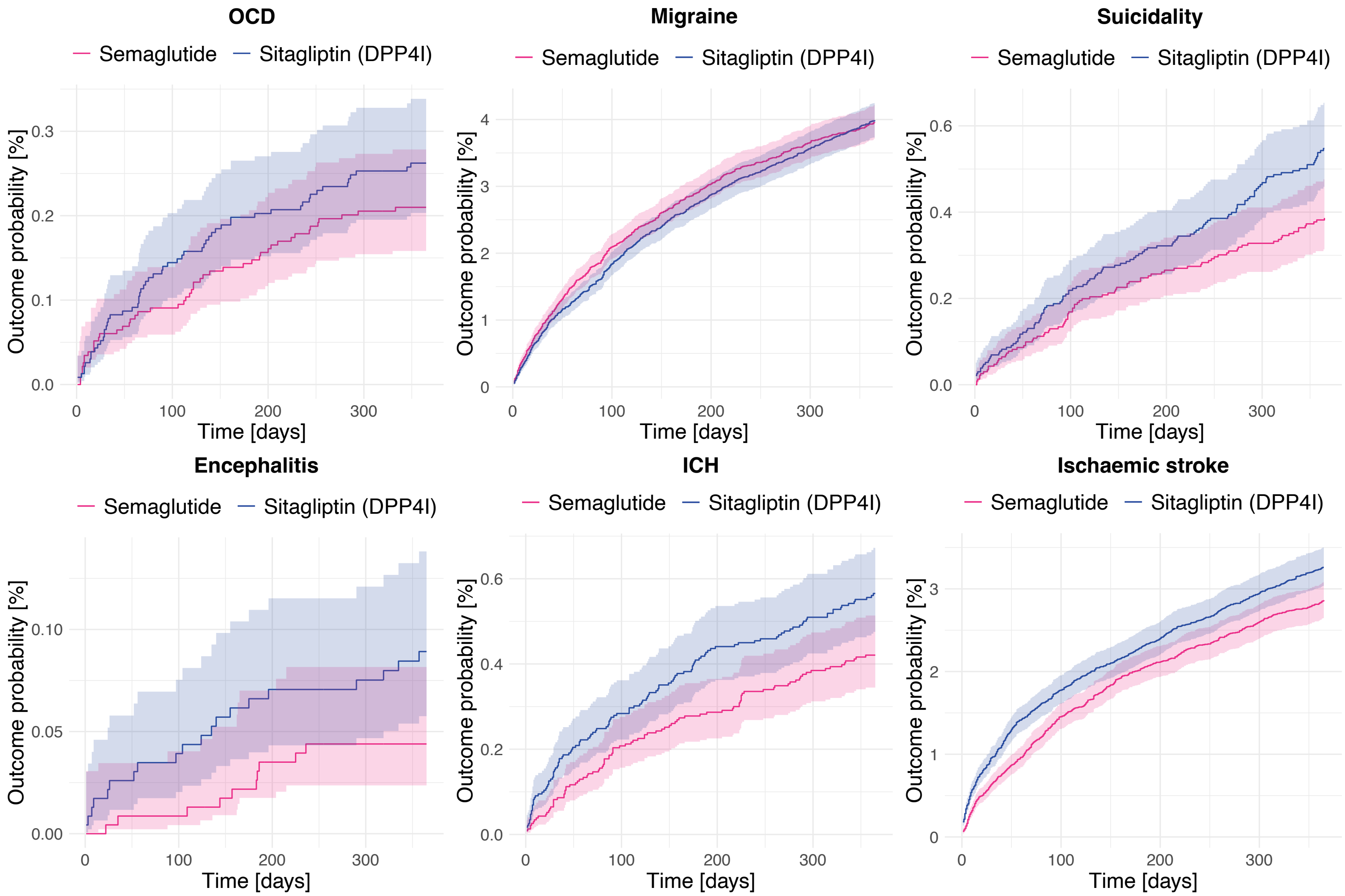
OCD
Semaglutide
Sitagliptin (DPP4I)
Outcome probability [%]
0.0
0.1
0.2
0.3
0
100
200
300
Time [days]
Migraine
Semaglutide
Sitagliptin (DPP4I)
Outcome probability [%]
0
1
2
3
4
0
100
200
300
Time [days]
Suicidality
Semaglutide
Sitagliptin (DPP4I)
Outcome probability [%]
0.0
0.2
0.4
0.6
0
100
200
300
Time [days]
Encephalitis
Semaglutide
Sitagliptin (DPP4I)
Outcome probability [%]
0.00
0.05
0.10
0
100
200
300
Time [days]
ICH
Semaglutide
Sitagliptin (DPP4I)
Outcome probability [%]
0.0
0.2
0.4
0.6
0
100
200
300
Time [days]
Ischaemic stroke
Semaglutide
Sitagliptin (DPP4I)
Outcome probability [%]
0
1
2
3
0
100
200
300
Time [days]

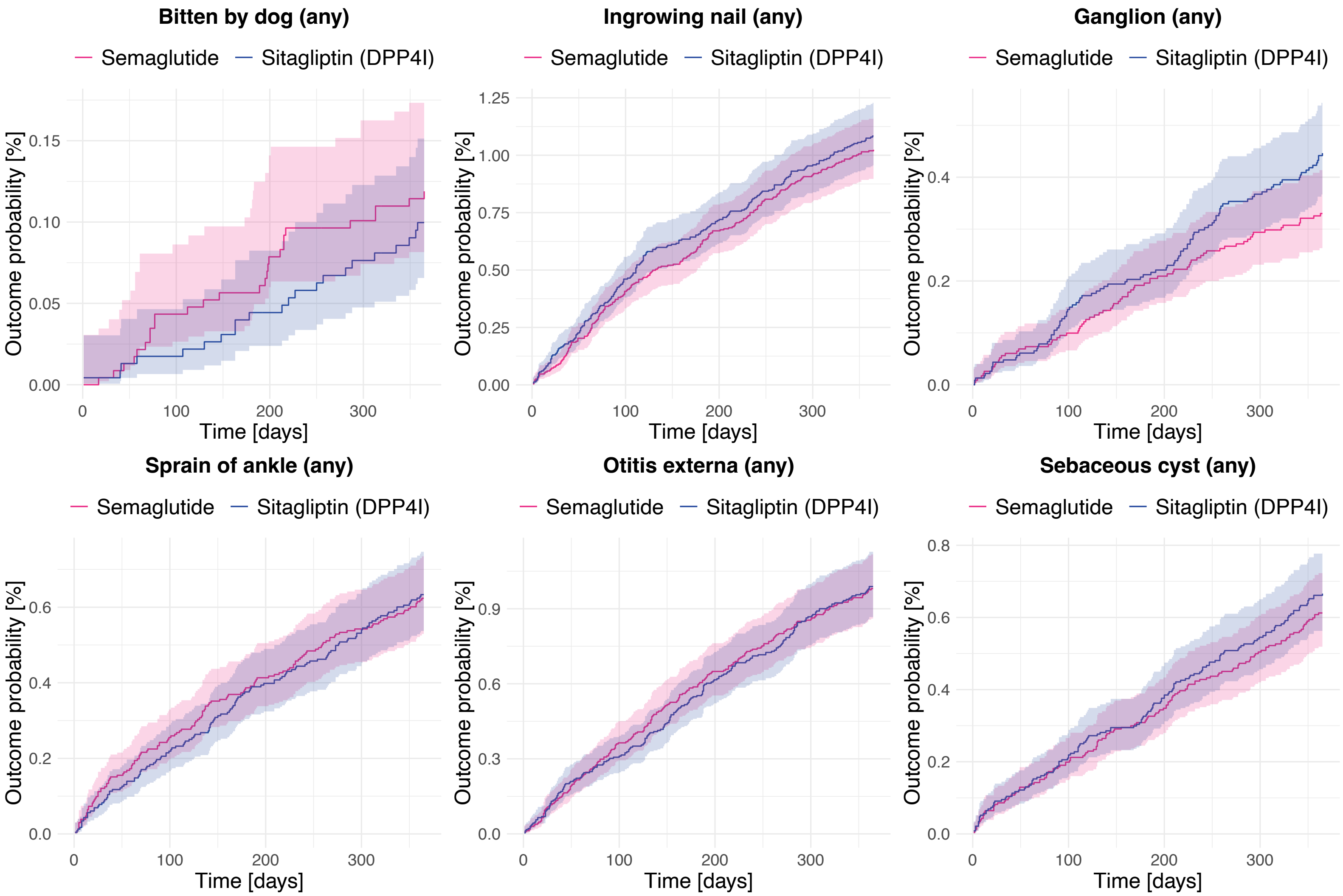

Bitten by dog (any)
Semaglutide
Sitagliptin (DPP4I)
Outcome probability [%]
0.00
0.05
0.10
0.15
Time [days]
0
100
200
300
Ingrowing nail (any)
Semaglutide
Sitagliptin (DPP4I)
Outcome probability [%]
0.00
0.25
0.50
0.75
1.00
1.25
Time [days]
0
100
200
300
Ganglion (any)
Semaglutide
Sitagliptin (DPP4I)
Outcome probability [%]
0.0
0.2
0.4
Time [days]
0
100
200
300
Sprain of ankle (any)
Semaglutide
Sitagliptin (DPP4I)
Outcome probability [%]
0.0
0.2
0.4
0.6
Time [days]
0
100
200
300
Otitis externa (any)
Semaglutide
Sitagliptin (DPP4I)
Outcome probability [%]
0.0
0.3
0.6
0.9
Time [days]
0
100
200
300
Sebaceous cyst (any)
Semaglutide
Sitagliptin (DPP4I)
Outcome probability [%]
0.0
0.2
0.4
0.6
0.8
Time [days]
0
100
200
300

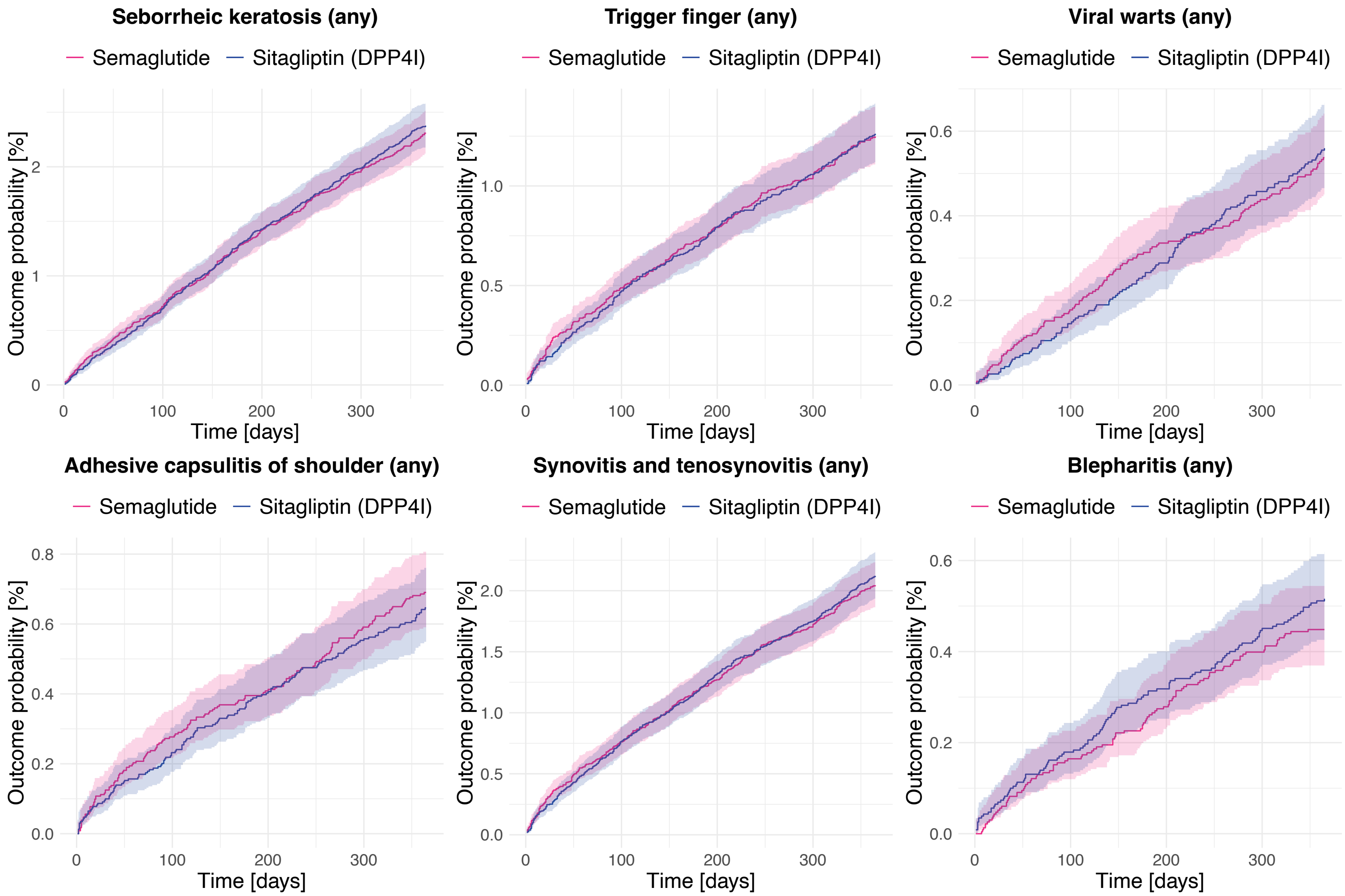
Seborrheic keratosis (any)
Semaglutide
Sitagliptin (DPP4I)
Outcome probability [%]
0
1
2
0
100
200
300
Time [days]
Trigger finger (any)
Semaglutide
Sitagliptin (DPP4I)
Outcome probability [%]
0.0
0.5
1.0
0
100
200
300
Time [days]
Viral warts (any)
Semaglutide
Sitagliptin (DPP4I)
Outcome probability [%]
0.0
0.2
0.4
0.6
0
100
200
300
Time [days]
Adhesive capsulitis of shoulder (any)
Semaglutide
Sitagliptin (DPP4I)
Outcome probability [%]
0.0
0.2
0.4
0.6
0.8
0
100
200
300
Time [days]
Synovitis and tenosynovitis (any)
Semaglutide
Sitagliptin (DPP4I)
Outcome probability [%]
0.0
0.5
1.0
1.5
2.0
0
100
200
300
Time [days]
Blepharitis (any)
Semaglutide
Sitagliptin (DPP4I)
Outcome probability [%]
0.0
0.2
0.4
0.6
0
100
200
300
Time [days]

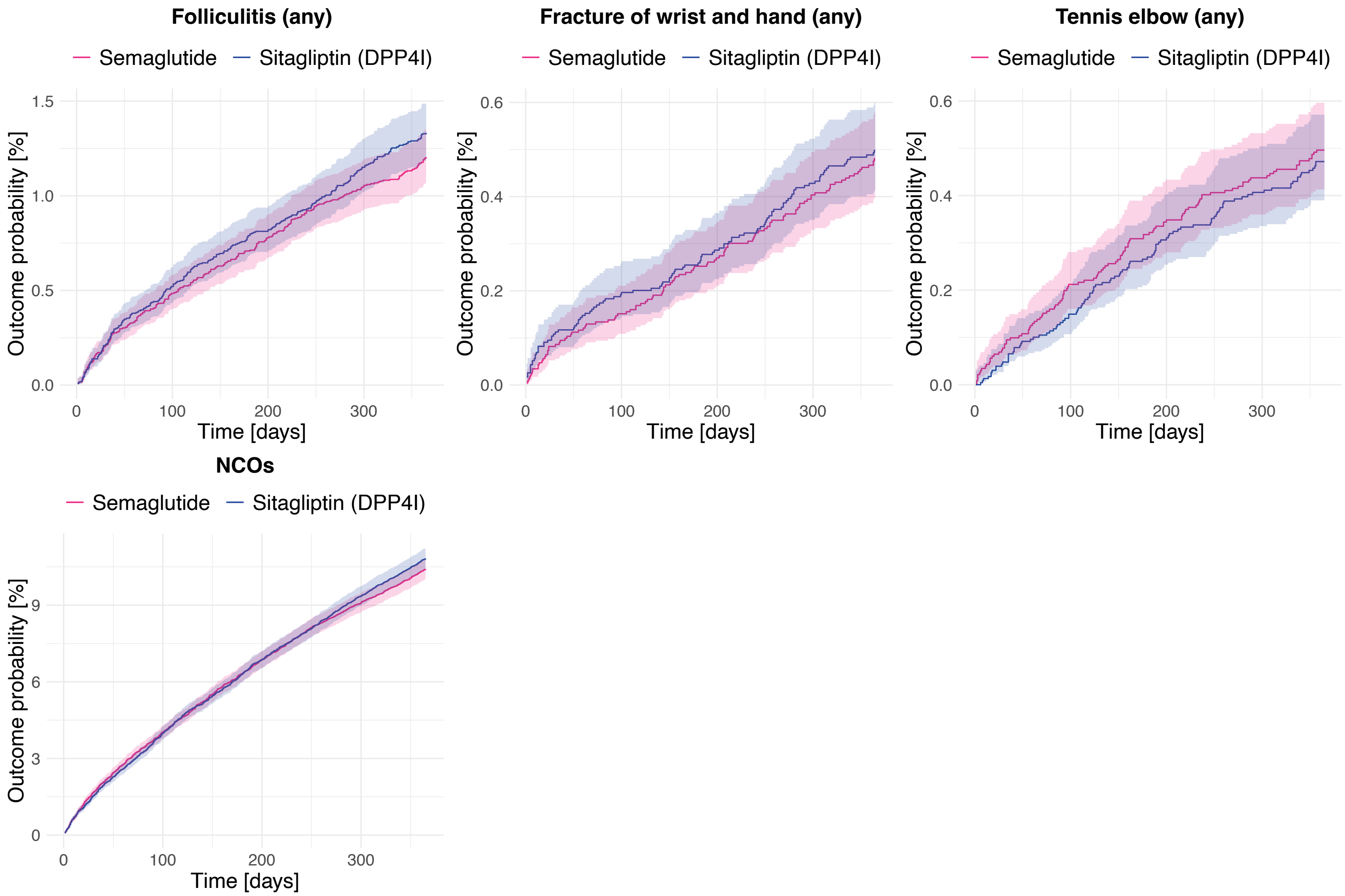
Folliculitis (any)
Semaglutide
Sitagliptin (DPP4I)
Outcome probability [%]
1.5
1.0
0.5
0.0
0
100
200
300
Time [days]
Fracture of wrist and hand (any)
Semaglutide
Sitagliptin (DPP4I)
Outcome probability [%]
0.6
0.4
0.2
0.0
0
100
200
300
Time [days]
Tennis elbow (any)
Semaglutide
Sitagliptin (DPP4I)
Outcome probability [%]
0.6
0.4
0.2
0.0
0
100
200
300
Time [days]
NCOs
Semaglutide
Sitagliptin (DPP4I)
Outcome probability [%]
9
6
3
0
0
100
200
300
Time [days]

b) Semaglutide vs Empagliflozin

First diagnosis

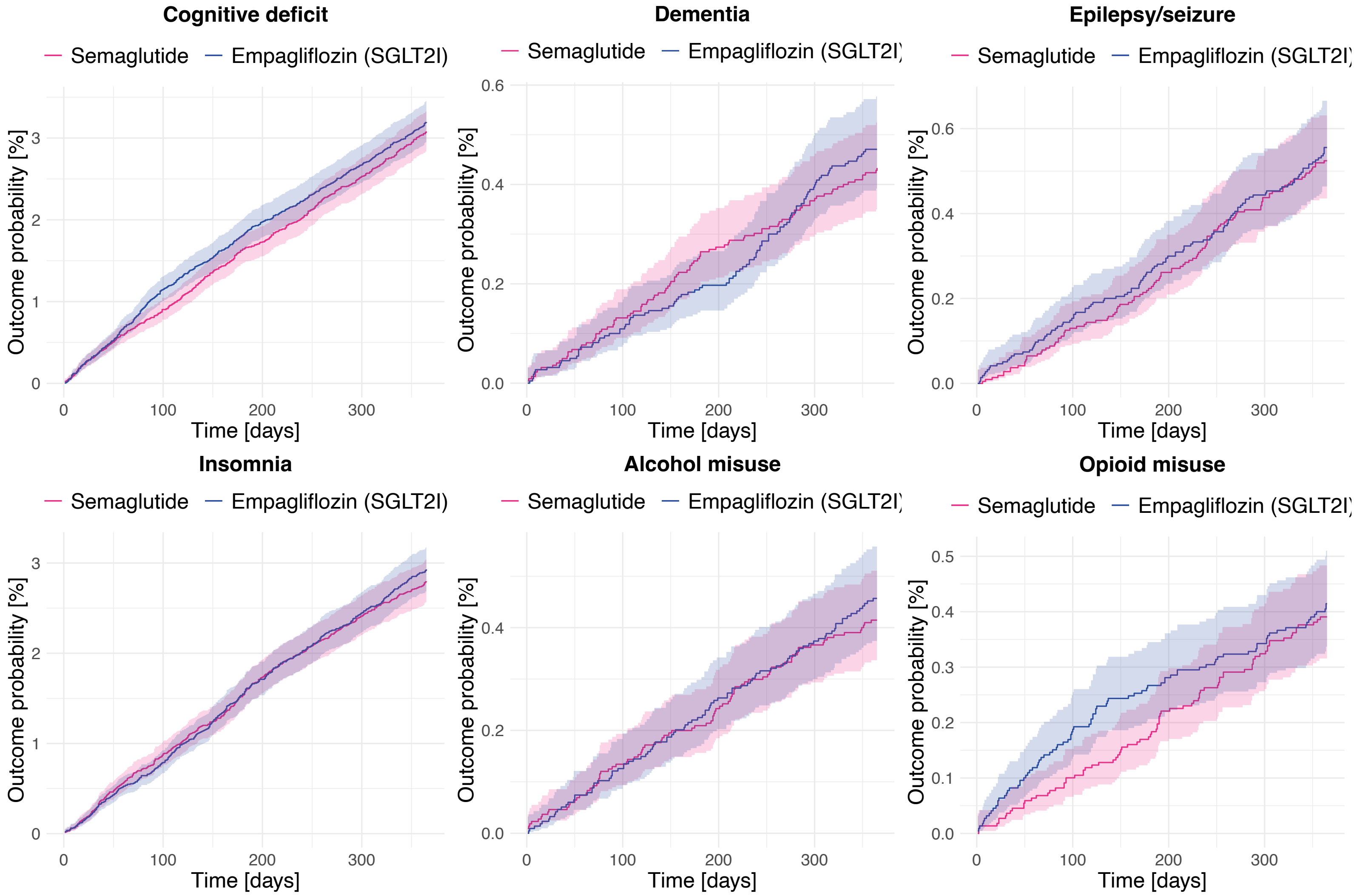

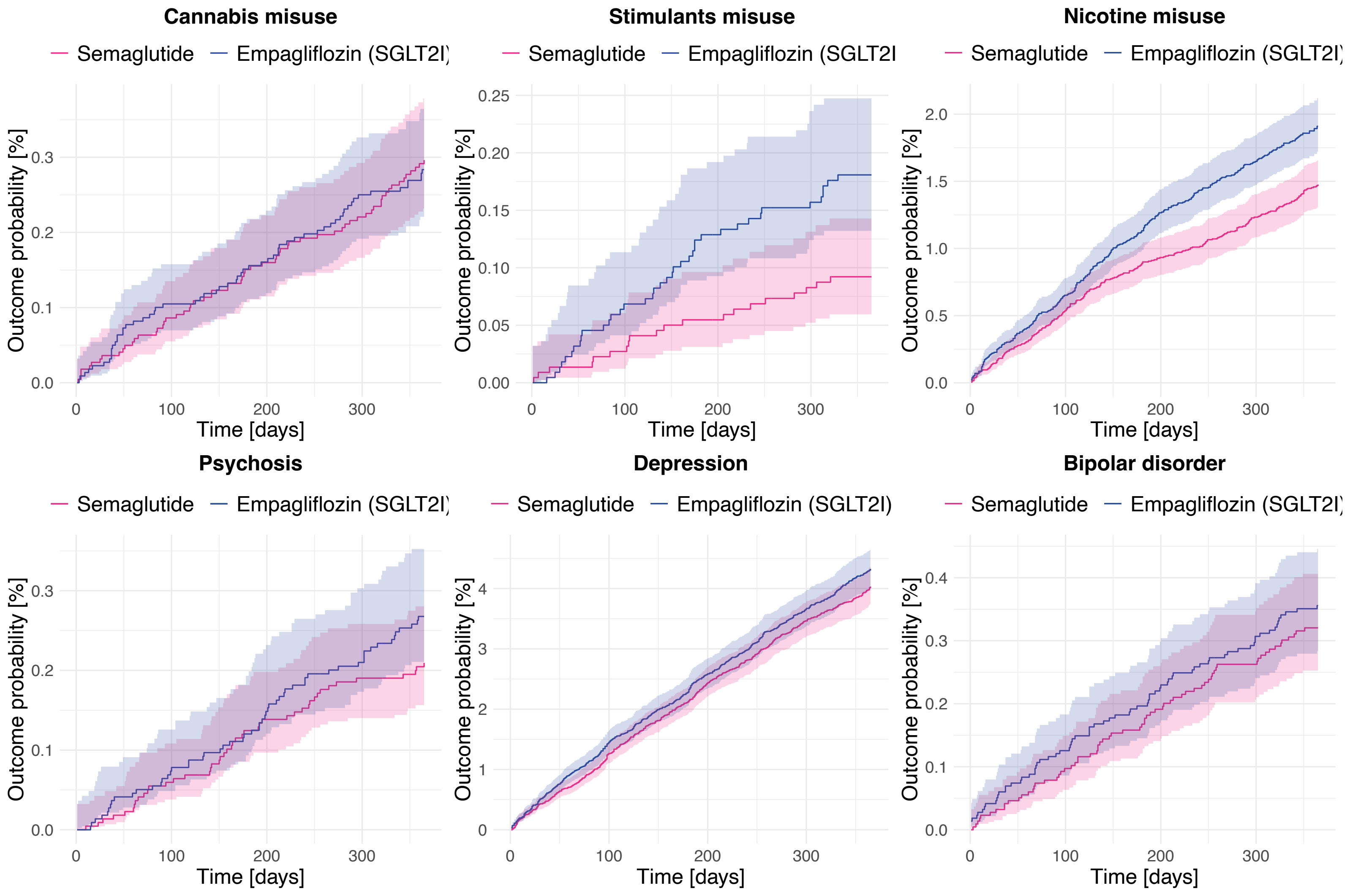

Cannabis misuse
Semaglutide
Empagliflozin (SGLT2I)
Outcome probability [%]
Time [days]
Stimulants misuse
Semaglutide
Empagliflozin (SGLT2I
Outcome probability [%]
Time [days]
Nicotine misuse
Semaglutide
Empagliflozin (SGLT2I)
Outcome probability [%]
Time [days]
Psychosis
Semaglutide
Empagliflozin (SGLT2I)
Outcome probability [%]
Time [days]
Depression
Semaglutide
Empagliflozin (SGLT2I)
Outcome probability [%]
Time [days]
Bipolar disorder
Semaglutide
Empagliflozin (SGLT2I)
Outcome probability [%]
Time [days]

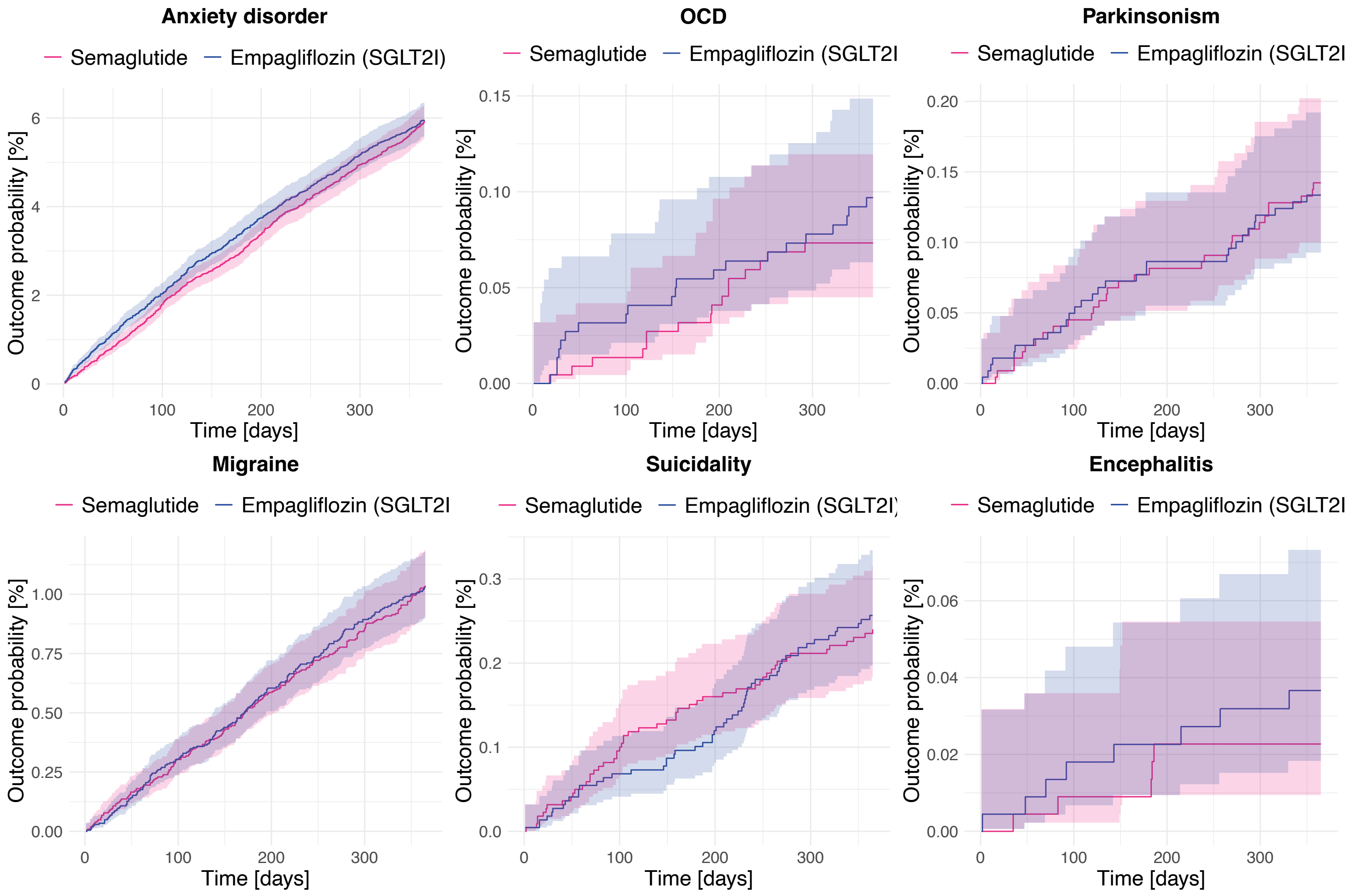
Anxiety disorder
Semaglutide
Empagliflozin (SGLT2I)
Outcome probability [%]
Time [days]
OCD
Semaglutide
Empagliflozin (SGLT2I
Outcome probability [%]
Time [days]
Parkinsonism
Semaglutide
Empagliflozin (SGLT2I
Outcome probability [%]
Time [days]
Migraine
Semaglutide
Empagliflozin (SGLT2I
Outcome probability [%]
Time [days]
Suicidality
Semaglutide
Empagliflozin (SGLT2I)
Outcome probability [%]
Time [days]
Encephalitis
Semaglutide
Empagliflozin (SGLT2I
Outcome probability [%]
Time [days]

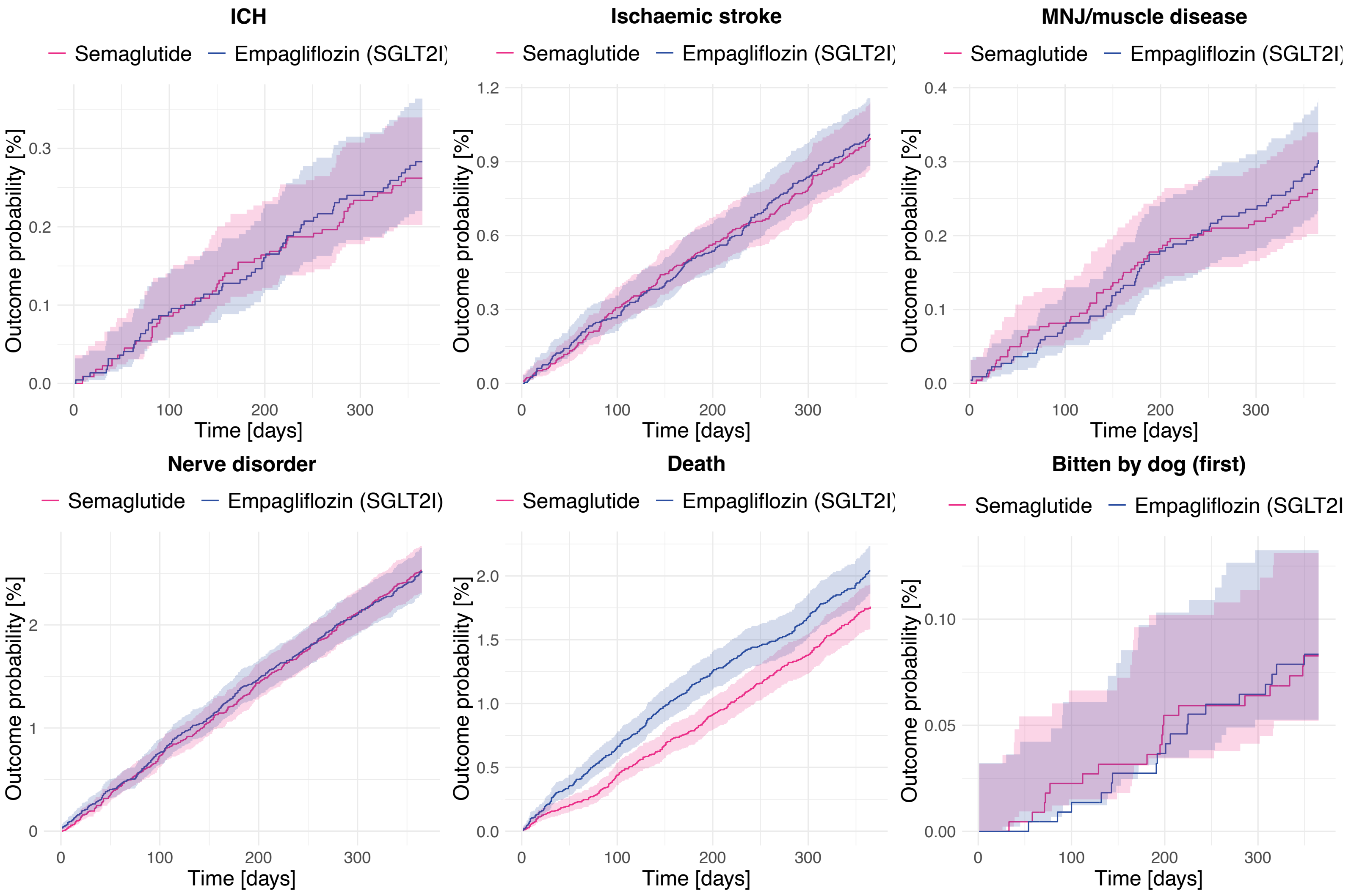
ICH
Semaglutide
Empagliflozin (SGLT2I)
Outcome probability [%]
Time [days]
Ischaemic stroke
MNJ/muscle disease
Nerve disorder
Death
Bitten by dog (first)

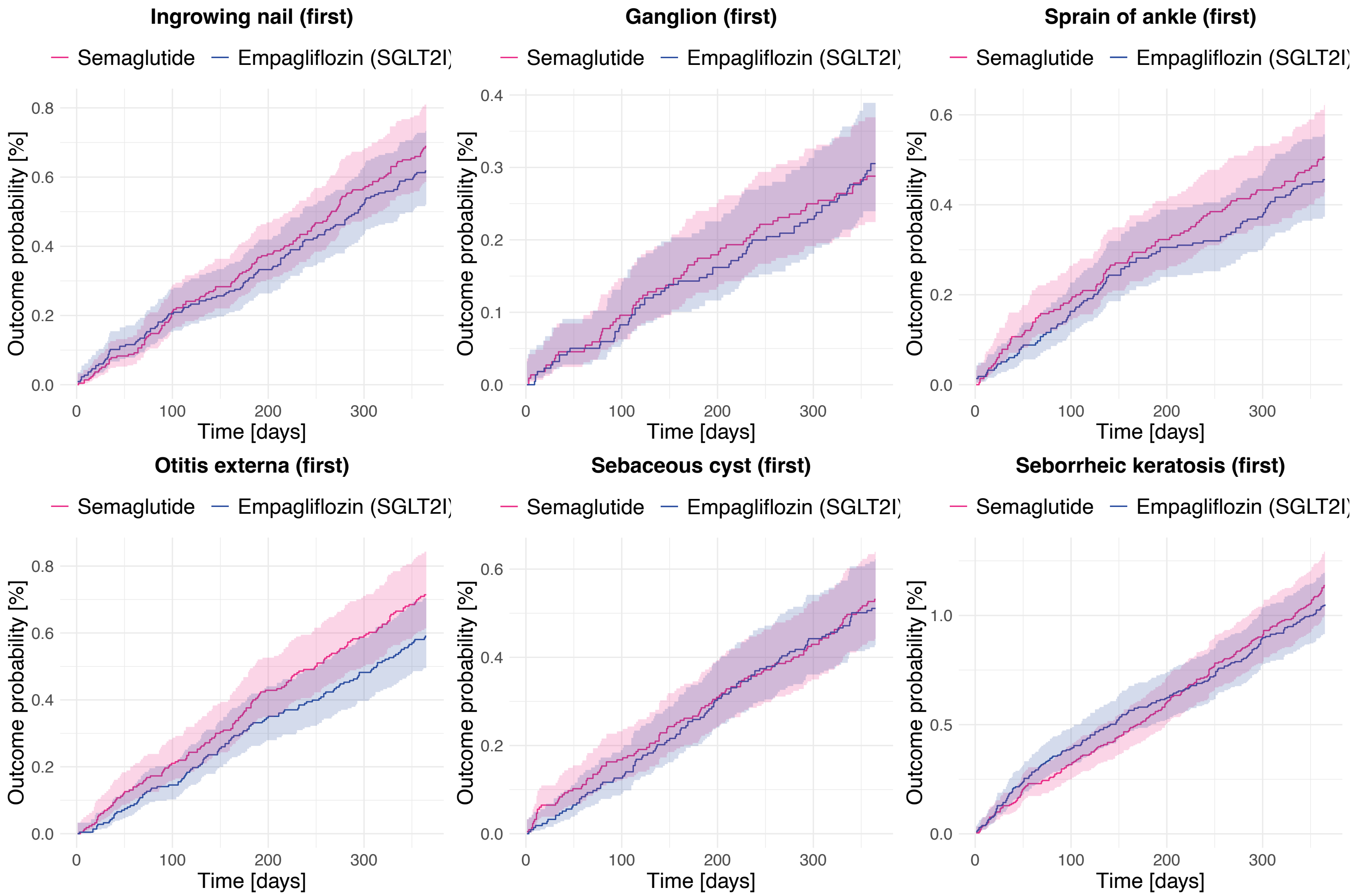
Ingrowing nail (first)
Semaglutide
Empagliflozin (SGLT2I)
Outcome probability [%]
Time [days]
Ganglion (first)
Sprain of ankle (first)
Otitis externa (first)
Sebaceous cyst (first)
Seborrheic keratosis (first)

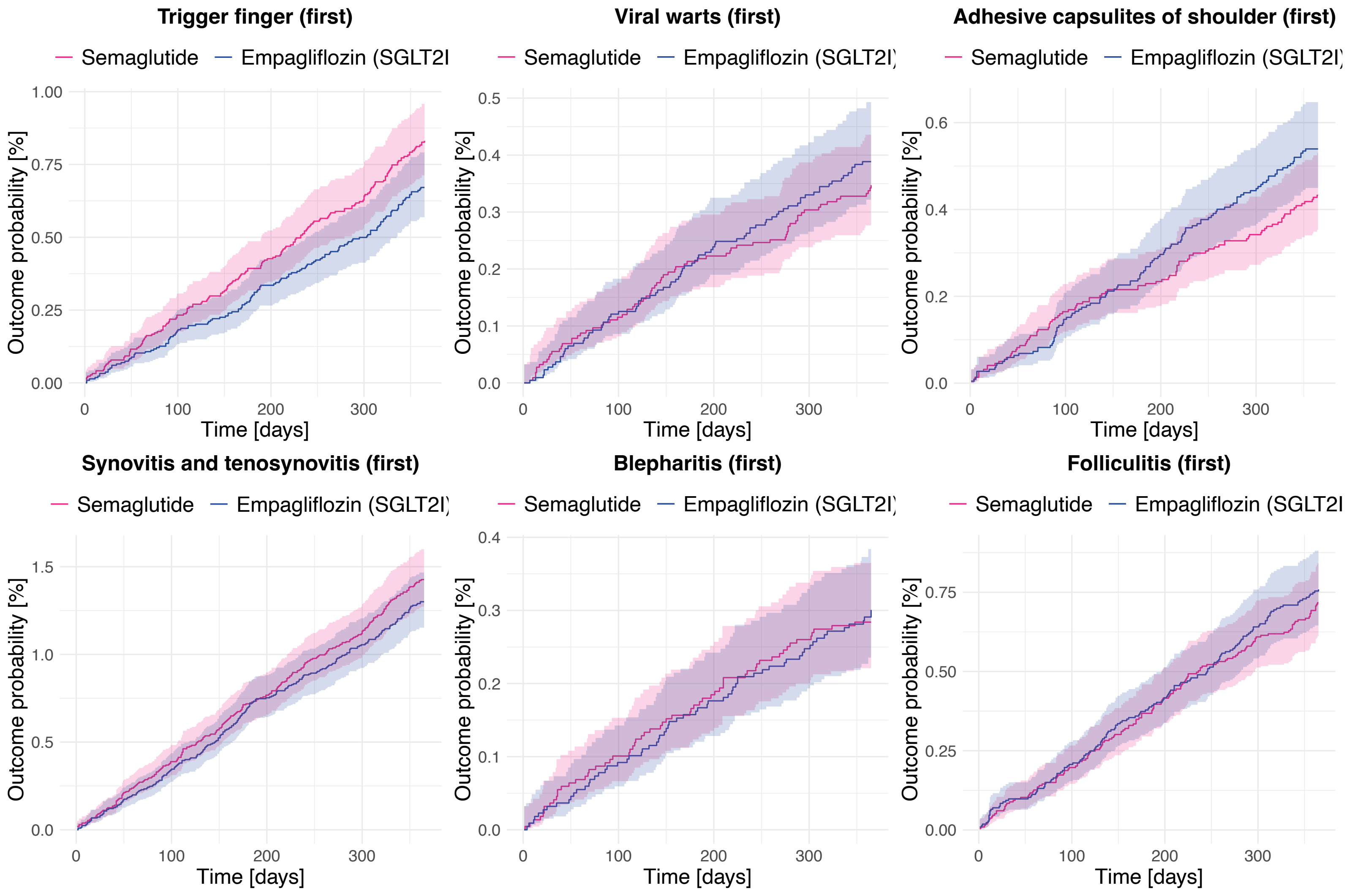
Trigger finger (first)
Semaglutide
Empagliflozin (SGLT2I
Outcome probability [%]
Time [days]
Viral warts (first)
Semaglutide
Empagliflozin (SGLT2I)
Outcome probability [%]
Time [days]
Adhesive capsulites of shoulder (first)
Semaglutide
Empagliflozin (SGLT2I)
Outcome probability [%]
Time [days]
Synovitis and tenosynovitis (first)
Semaglutide
Empagliflozin (SGLT2I)
Outcome probability [%]
Time [days]
Blepharitis (first)
Semaglutide
Empagliflozin (SGLT2I)
Outcome probability [%]
Time [days]
Folliculitis (first)
Semaglutide
Empagliflozin (SGLT2I
Outcome probability [%]
Time [days]

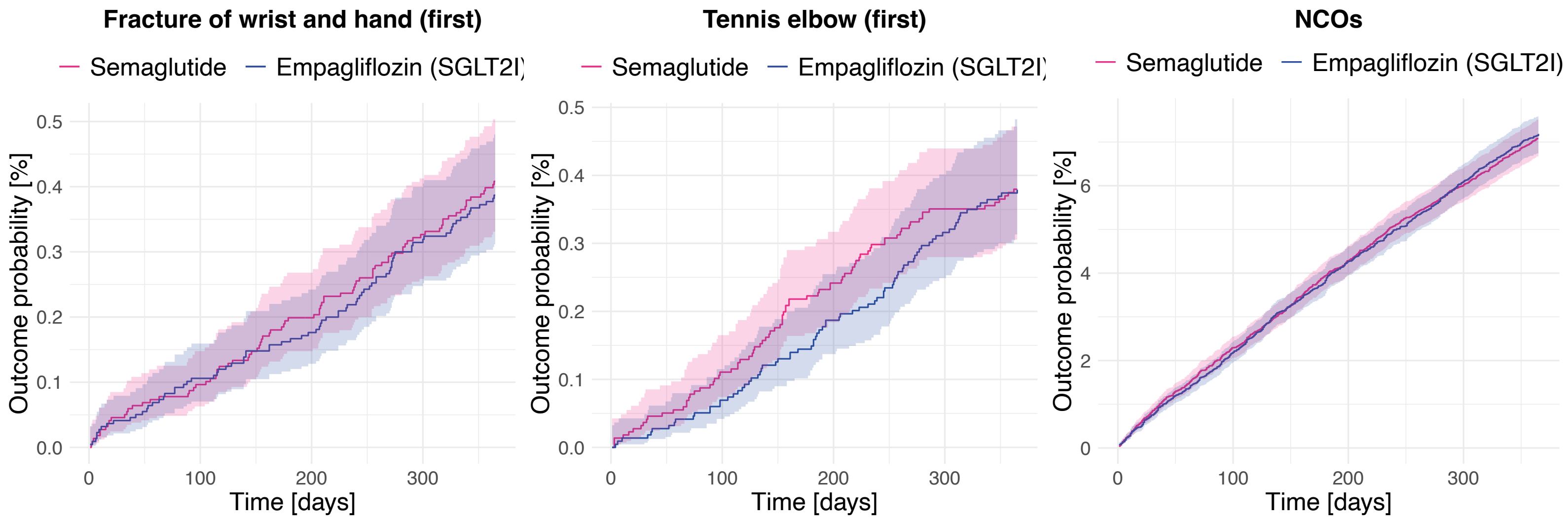

Fracture of wrist and hand (first)
Semaglutide
Empagliflozin (SGLT2I)
Outcome probability [%]
0.0
0.1
0.2
0.3
0.4
0.5
0
100
200
300
Time [days]
Tennis elbow (first)
Semaglutide
Empagliflozin (SGLT2I)
Outcome probability [%]
0.0
0.1
0.2
0.3
0.4
0.5
0
100
200
300
Time [days]
NCOs
Semaglutide
Empagliflozin (SGLT2I)
Outcome probability [%]
0
2
4
6
0
100
200
300
Time [days]

Any diagnosis

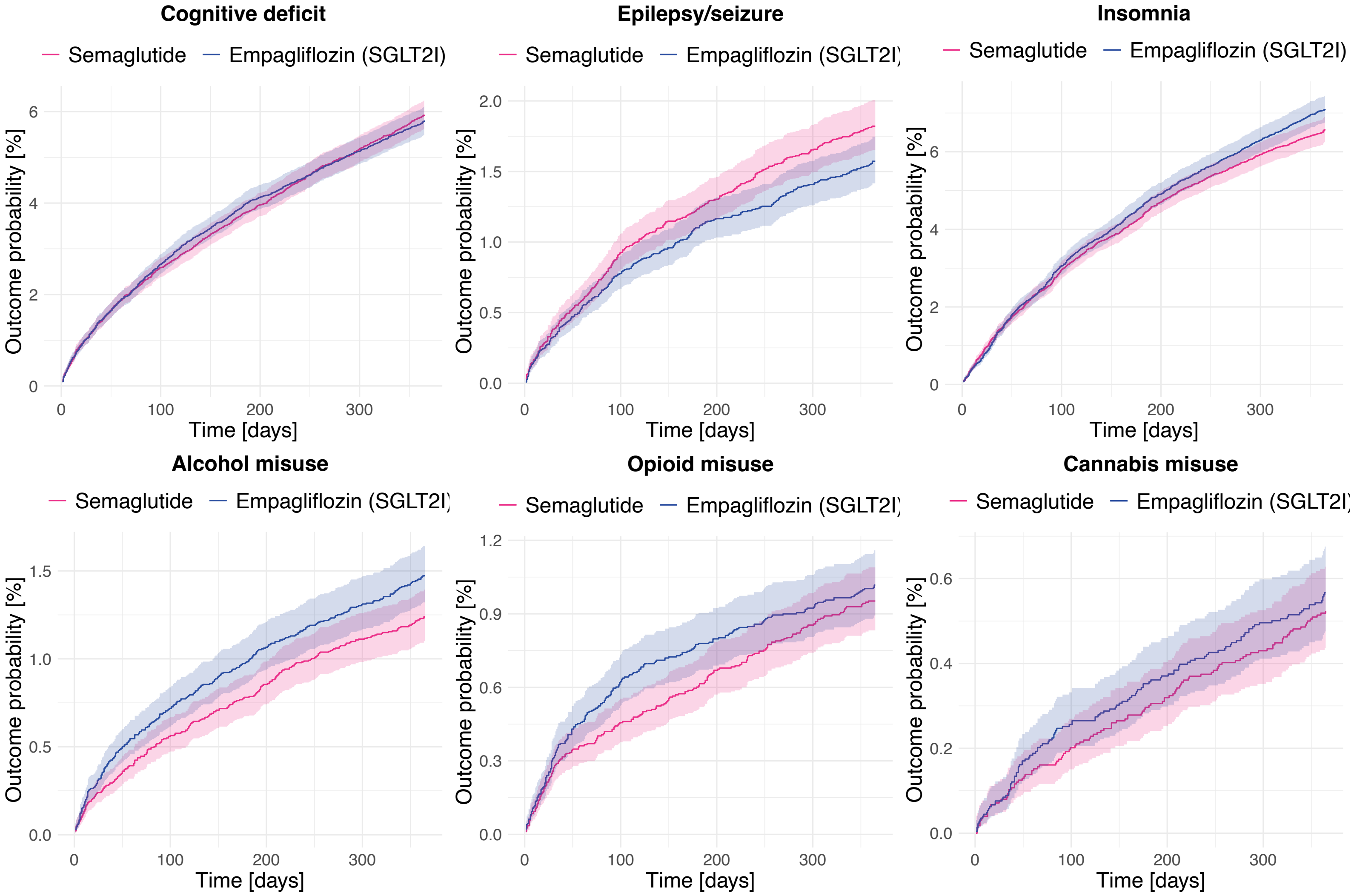

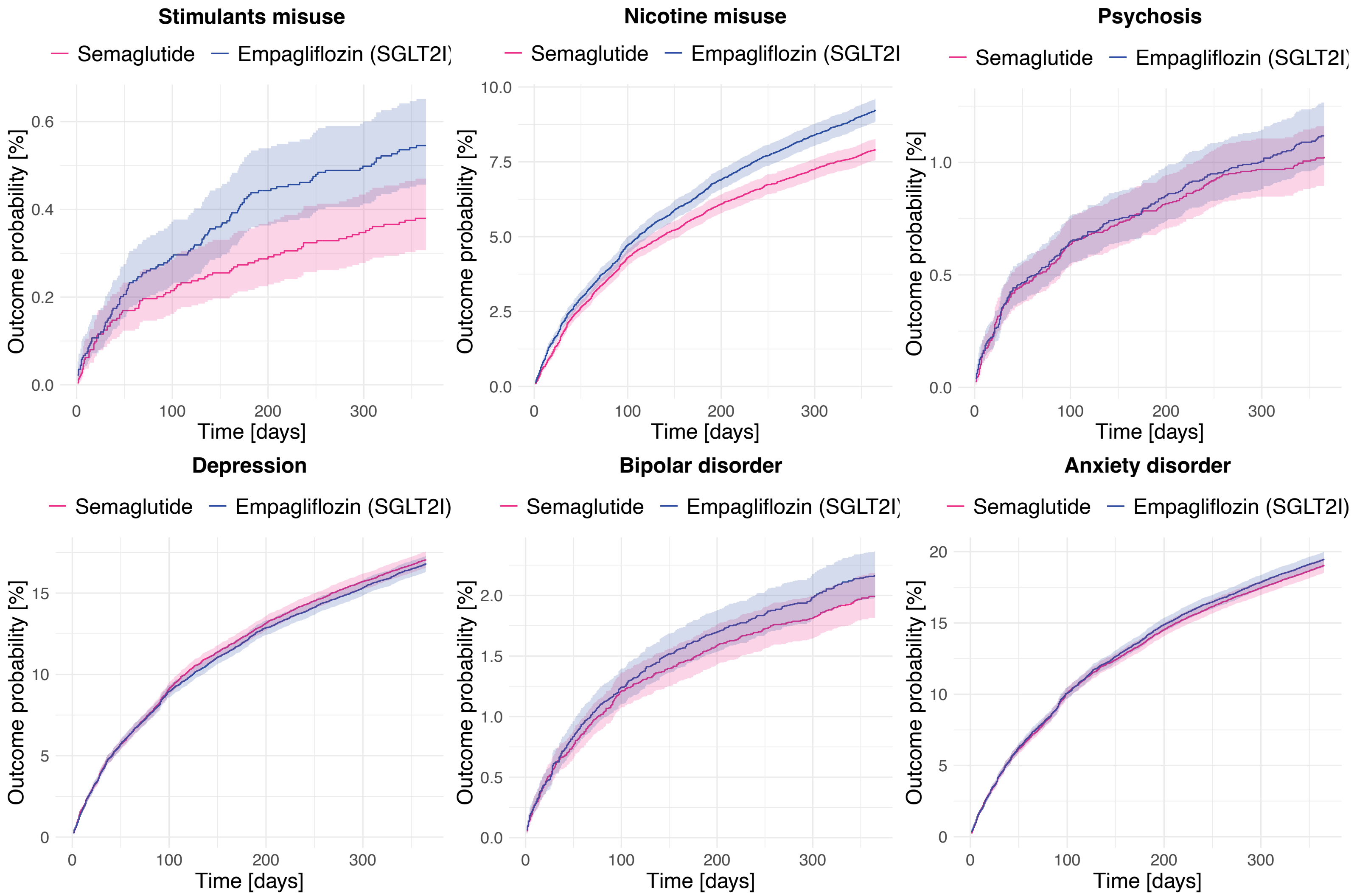
Stimulants misuse
Semaglutide
Empagliflozin (SGLT2I)
Outcome probability [%]
Time [days]
Nicotine misuse
Semaglutide
Empagliflozin (SGLT2I
Outcome probability [%]
Time [days]
Psychosis
Semaglutide
Empagliflozin (SGLT2I)
Outcome probability [%]
Time [days]
Depression
Semaglutide
Empagliflozin (SGLT2I)
Outcome probability [%]
Time [days]
Bipolar disorder
Semaglutide
Empagliflozin (SGLT2I)
Outcome probability [%]
Time [days]
Anxiety disorder
Semaglutide
Empagliflozin (SGLT2I)
Outcome probability [%]
Time [days]

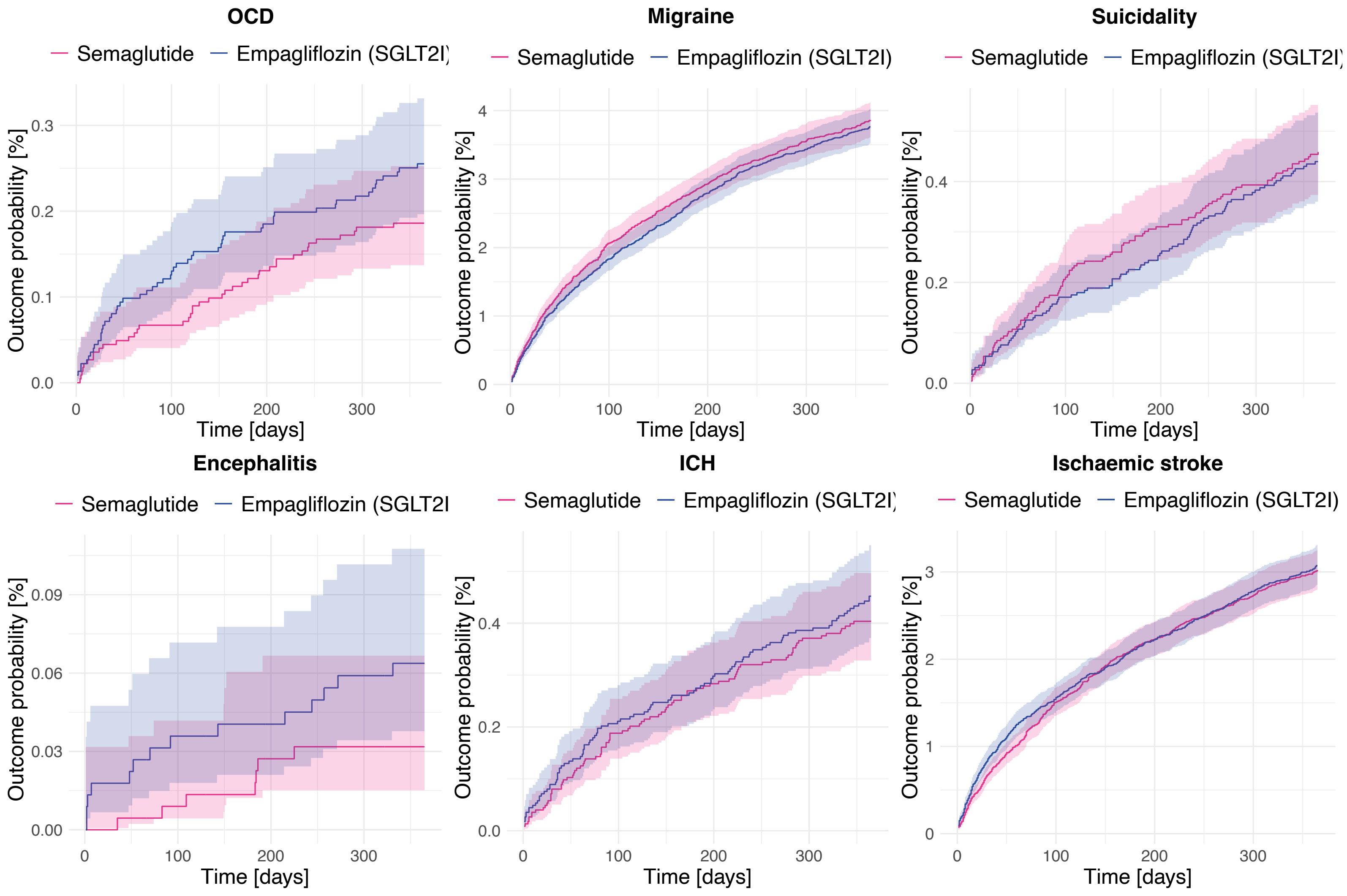

OCD
Semaglutide
Empagliflozin (SGLT2I)
Outcome probability [%]
Time [days]
Migraine
Semaglutide
Empagliflozin (SGLT2I)
Outcome probability [%]
Time [days]
Suicidality
Semaglutide
Empagliflozin (SGLT2I)
Outcome probability [%]
Time [days]
Encephalitis
Semaglutide
Empagliflozin (SGLT2I
Outcome probability [%]
Time [days]
ICH
Semaglutide
Empagliflozin (SGLT2I)
Outcome probability [%]
Time [days]
Ischaemic stroke
Semaglutide
Empagliflozin (SGLT2I)
Outcome probability [%]
Time [days]

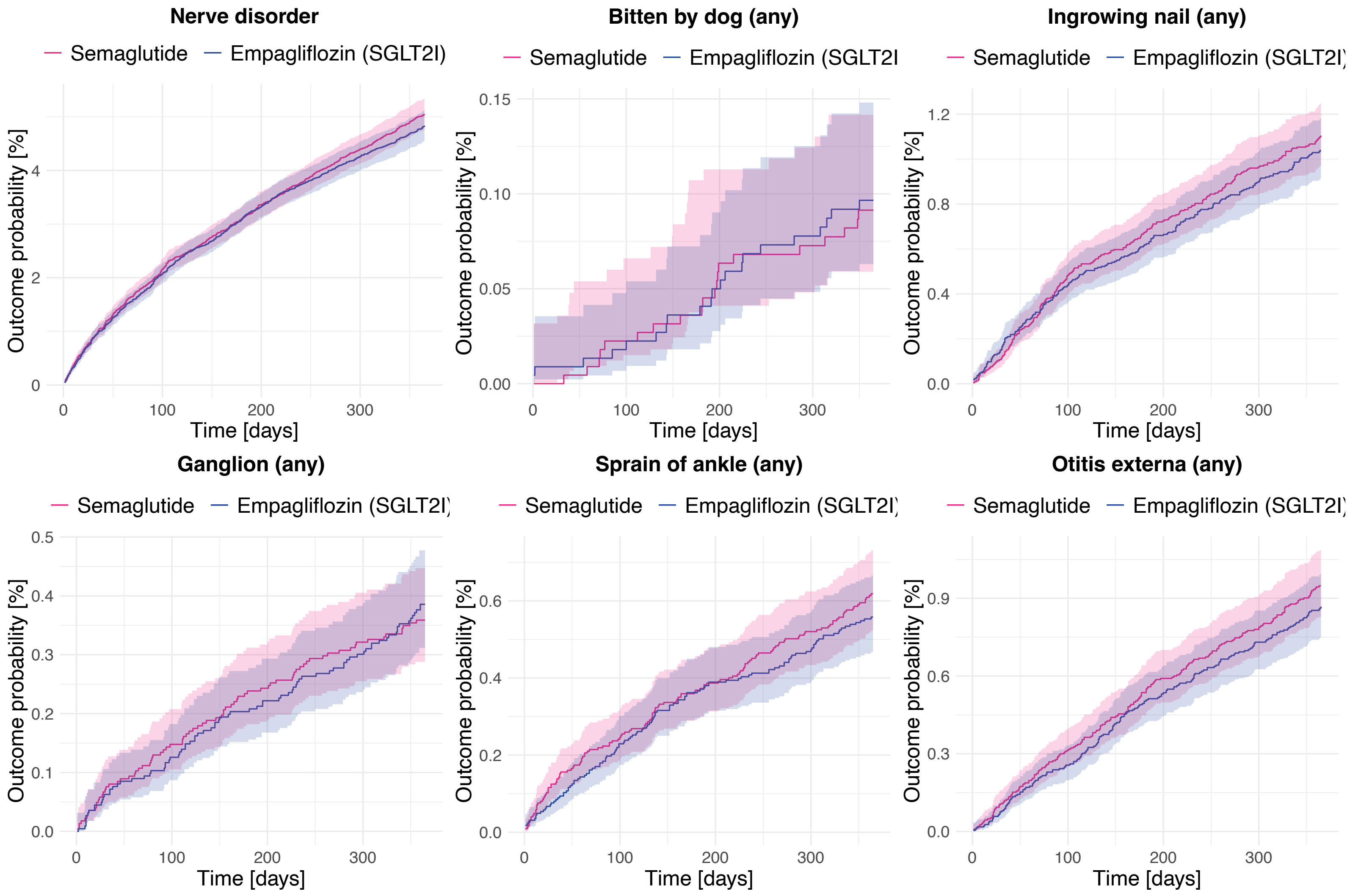

Nerve disorder
Semaglutide
Empagliflozin (SGLT2I)
Outcome probability [%]
Time [days]
Bitten by dog (any)
Semaglutide
Empagliflozin (SGLT2I
Outcome probability [%]
Time [days]
Ingrowing nail (any)
Semaglutide
Empagliflozin (SGLT2I
Outcome probability [%]
Time [days]
Ganglion (any)
Semaglutide
Empagliflozin (SGLT2I
Outcome probability [%]
Time [days]
Sprain of ankle (any)
Semaglutide
Empagliflozin (SGLT2I
Outcome probability [%]
Time [days]
Otitis externa (any)
Semaglutide
Empagliflozin (SGLT2I
Outcome probability [%]
Time [days]

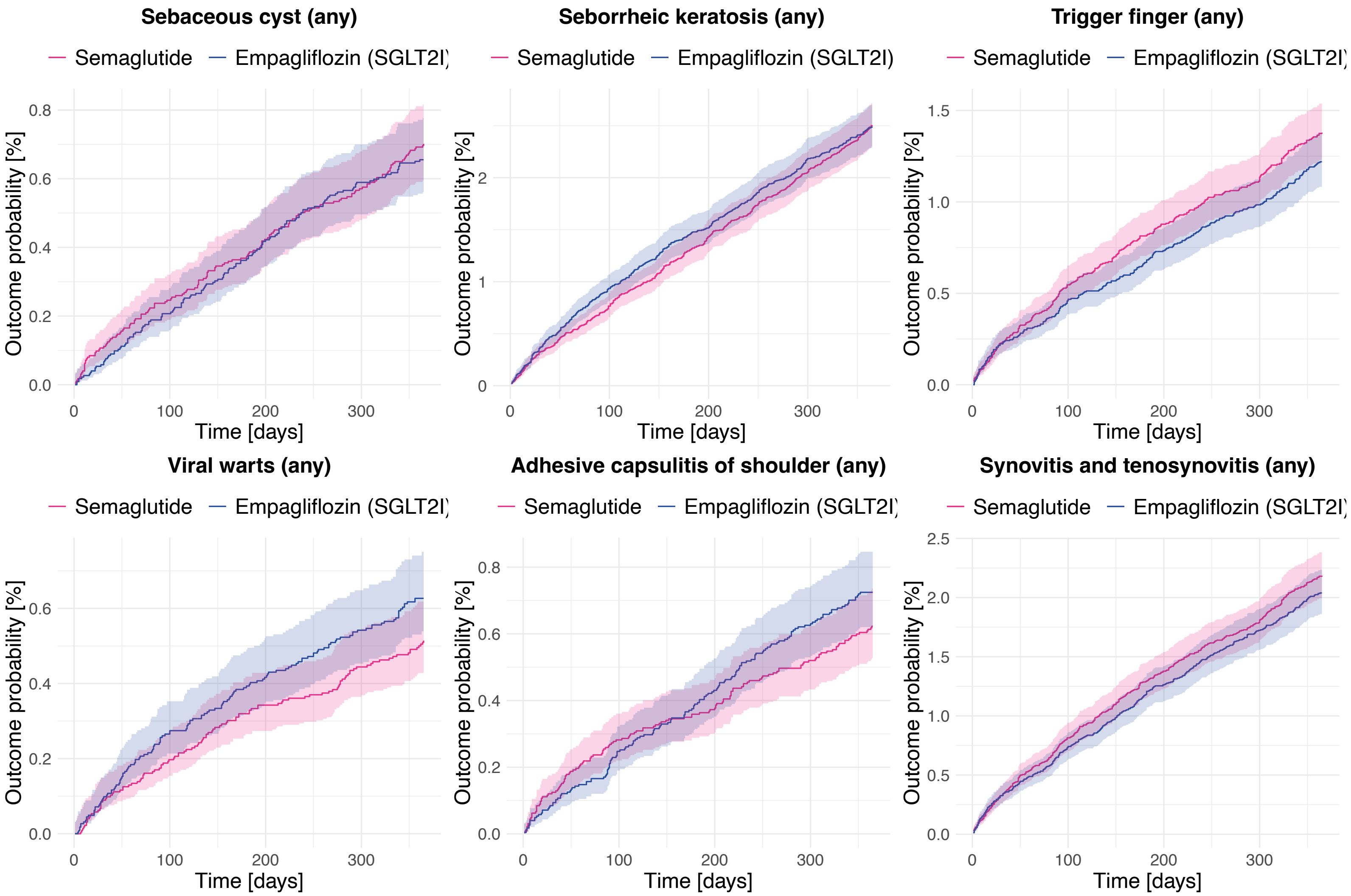
Sebaceous cyst (any)
Semaglutide
Empagliflozin (SGLT2I)
Outcome probability [%]
Time [days]
Seborrheic keratosis (any)
Semaglutide
Empagliflozin (SGLT2I)
Outcome probability [%]
Time [days]
Trigger finger (any)
Semaglutide
Empagliflozin (SGLT2I)
Outcome probability [%]
Time [days]
Viral warts (any)
Semaglutide
Empagliflozin (SGLT2I)
Outcome probability [%]
Time [days]
Adhesive capsulitis of shoulder (any)
Semaglutide
Empagliflozin (SGLT2I)
Outcome probability [%]
Time [days]
Synovitis and tenosynovitis (any)
Semaglutide
Empagliflozin (SGLT2I)
Outcome probability [%]
Time [days]

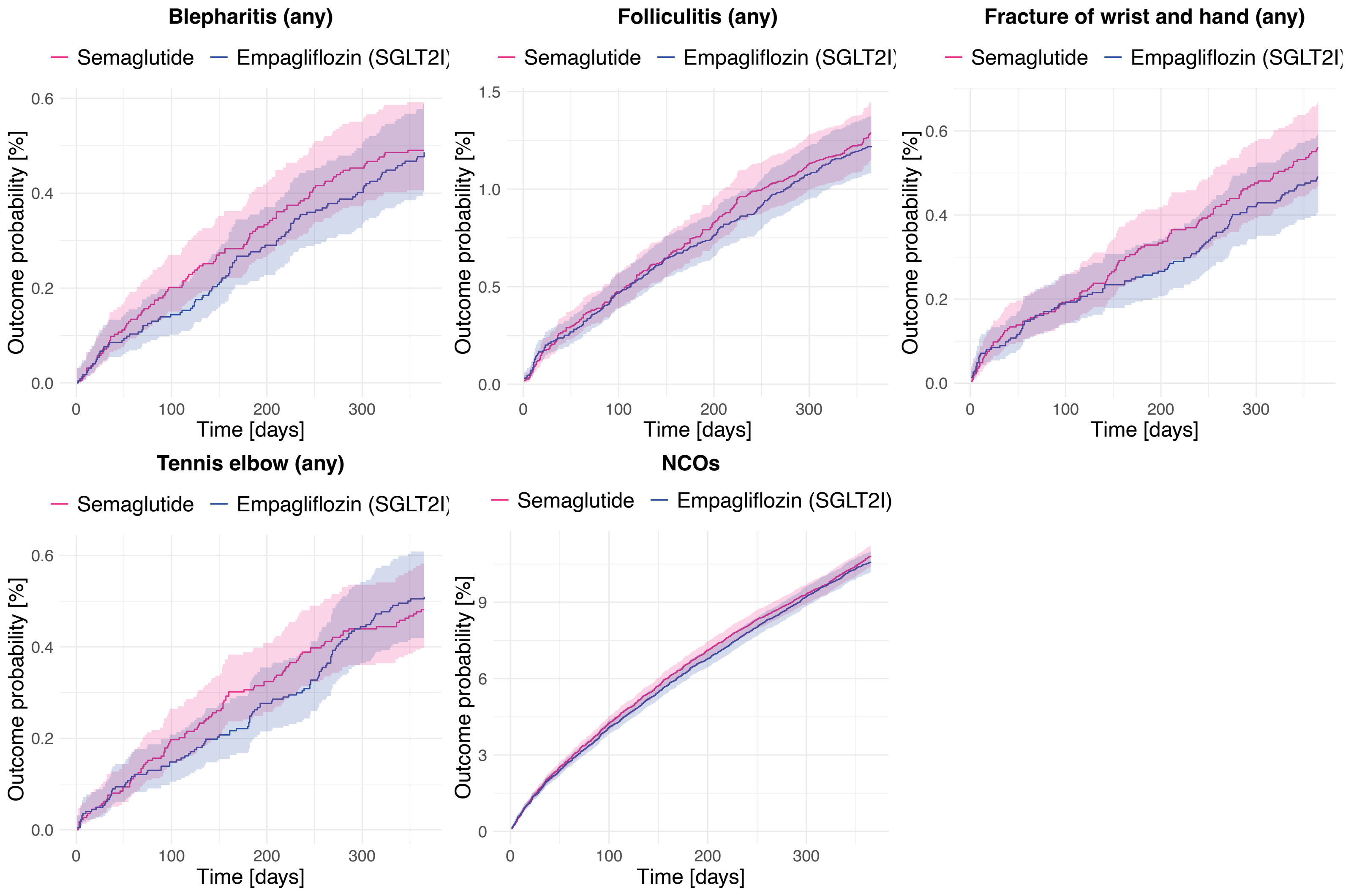

Blepharitis (any)
Semaglutide
Empagliflozin (SGLT2I)
Outcome probability [%]
Time [days]
Folliculitis (any)
Semaglutide
Empagliflozin (SGLT2I)
Outcome probability [%]
Time [days]
Fracture of wrist and hand (any)
Semaglutide
Empagliflozin (SGLT2I)
Outcome probability [%]
Time [days]
Tennis elbow (any)
Semaglutide
Empagliflozin (SGLT2I)
Outcome probability [%]
Time [days]
NCOs
Semaglutide
Empagliflozin (SGLT2I)
Outcome probability [%]
Time [days]

c) Semaglutide vs Glipizide

First diagnosis

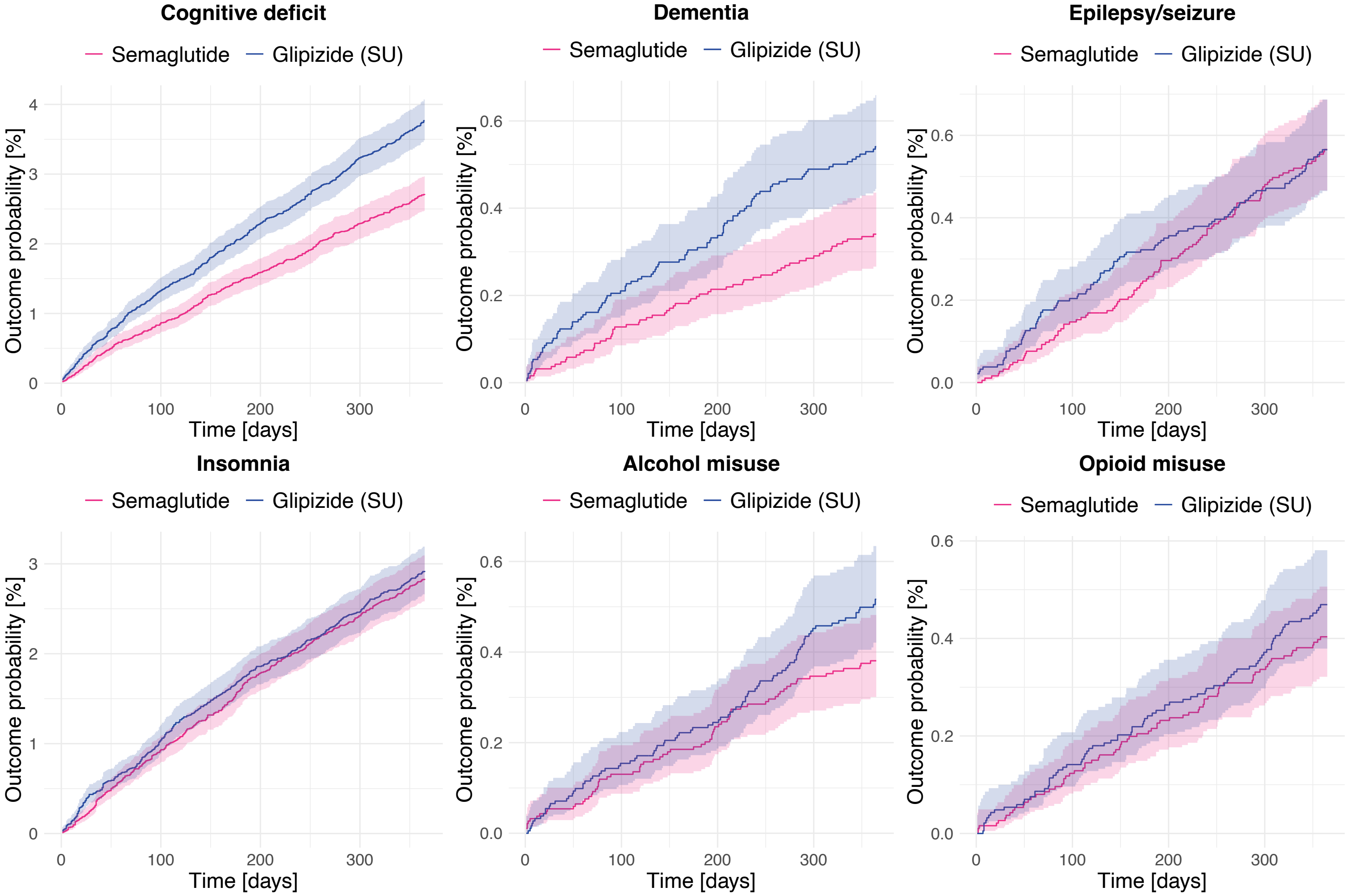

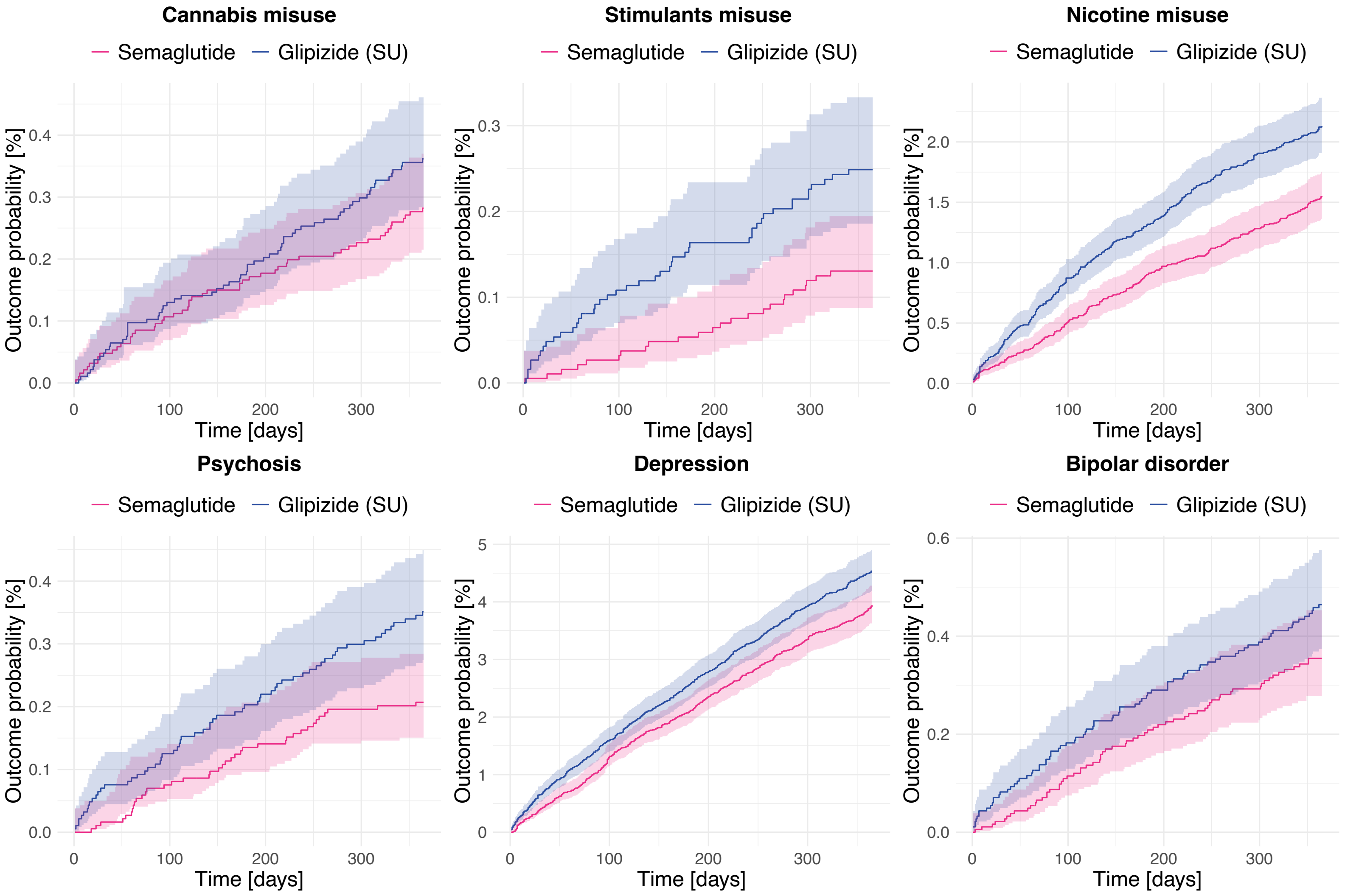
Cannabis misuse
Semaglutide
Glipizide (SU)
Outcome probability [%]
Time [days]
Stimulants misuse
Semaglutide
Glipizide (SU)
Outcome probability [%]
Time [days]
Nicotine misuse
Semaglutide
Glipizide (SU)
Outcome probability [%]
Time [days]
Psychosis
Semaglutide
Glipizide (SU)
Outcome probability [%]
Time [days]
Depression
Semaglutide
Glipizide (SU)
Outcome probability [%]
Time [days]
Bipolar disorder
Semaglutide
Glipizide (SU)
Outcome probability [%]
Time [days]

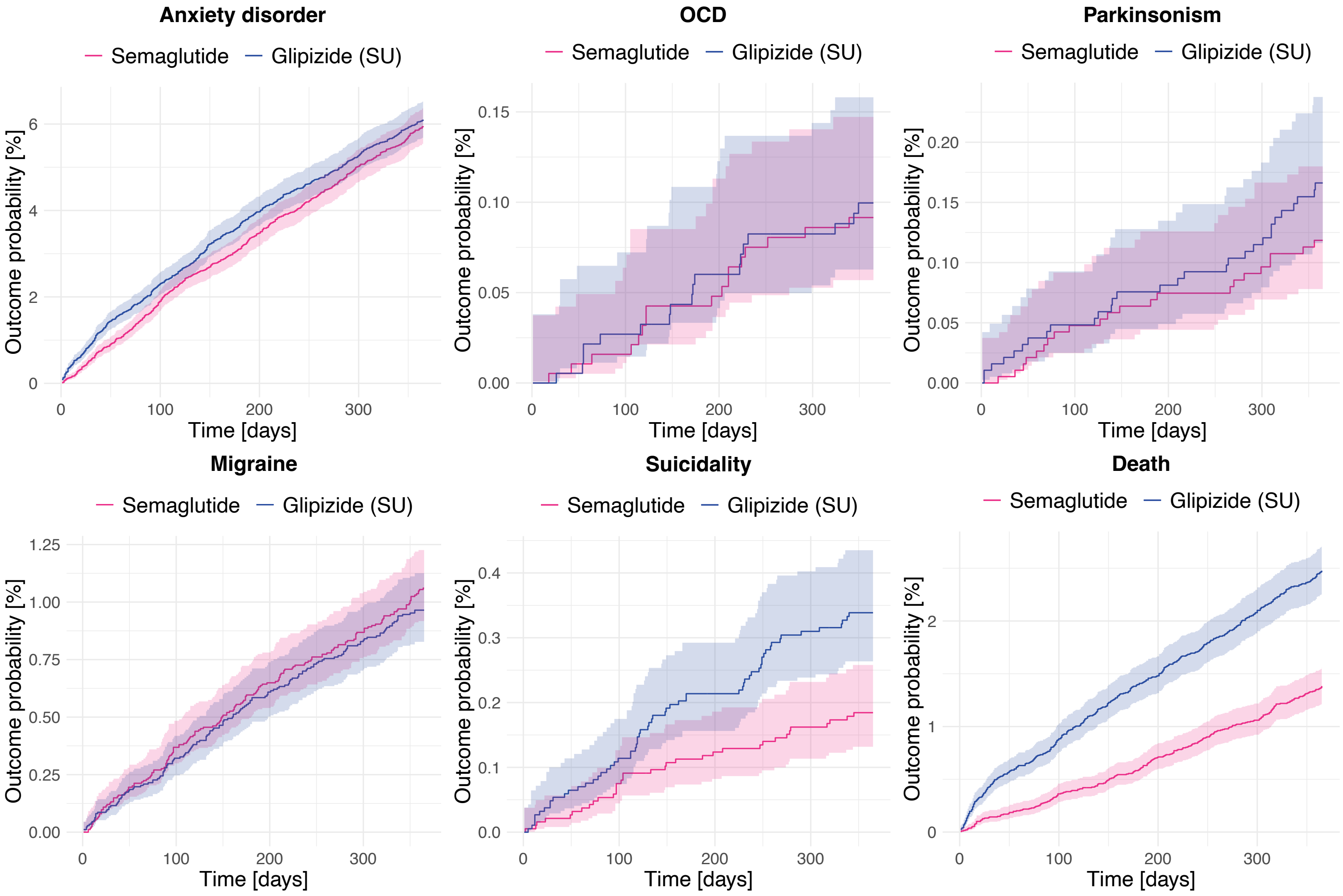
Anxiety disorder
Semaglutide
Glipizide (SU)
Outcome probability [%]
Time [days]
OCD
Semaglutide
Glipizide (SU)
Outcome probability [%]
Time [days]
Parkinsonism
Semaglutide
Glipizide (SU)
Outcome probability [%]
Time [days]
Migraine
Semaglutide
Glipizide (SU)
Outcome probability [%]
Time [days]
Suicidality
Semaglutide
Glipizide (SU)
Outcome probability [%]
Time [days]
Death
Semaglutide
Glipizide (SU)
Outcome probability [%]
Time [days]

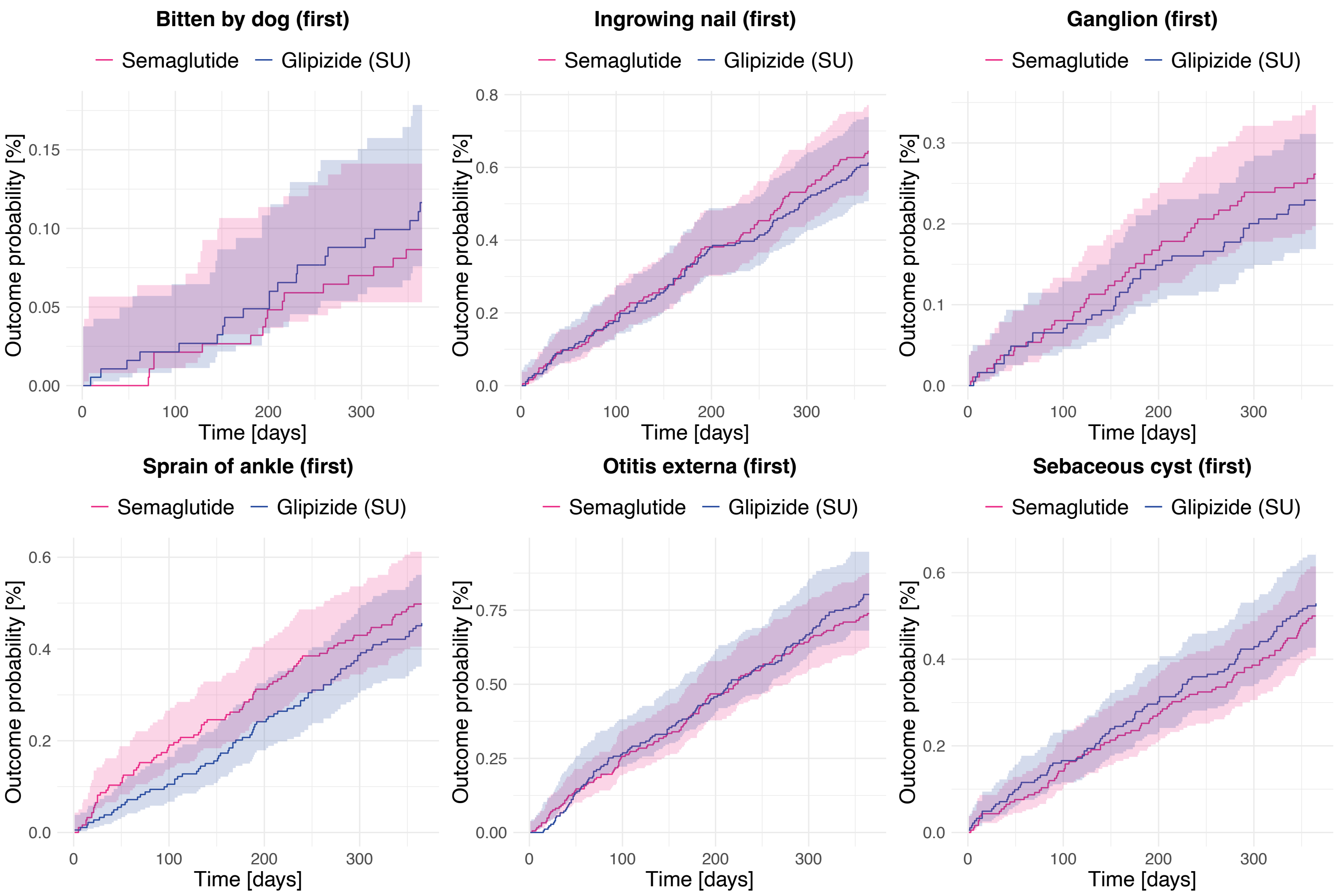

Bitten by dog (first)
Semaglutide
Glipizide (SU)
Outcome probability [%]
0.00
0.05
0.10
0.15
0
100
200
300
Time [days]
Ingrowing nail (first)
Semaglutide
Glipizide (SU)
Outcome probability [%]
0.0
0.2
0.4
0.6
0.8
0
100
200
300
Time [days]
Ganglion (first)
Semaglutide
Glipizide (SU)
Outcome probability [%]
0.0
0.1
0.2
0.3
0
100
200
300
Time [days]
Sprain of ankle (first)
Semaglutide
Glipizide (SU)
Outcome probability [%]
0.0
0.2
0.4
0.6
0
100
200
300
Time [days]
Otitis externa (first)
Semaglutide
Glipizide (SU)
Outcome probability [%]
0.00
0.25
0.50
0.75
0
100
200
300
Time [days]
Sebaceous cyst (first)
Semaglutide
Glipizide (SU)
Outcome probability [%]
0.0
0.2
0.4
0.6
0
100
200
300
Time [days]

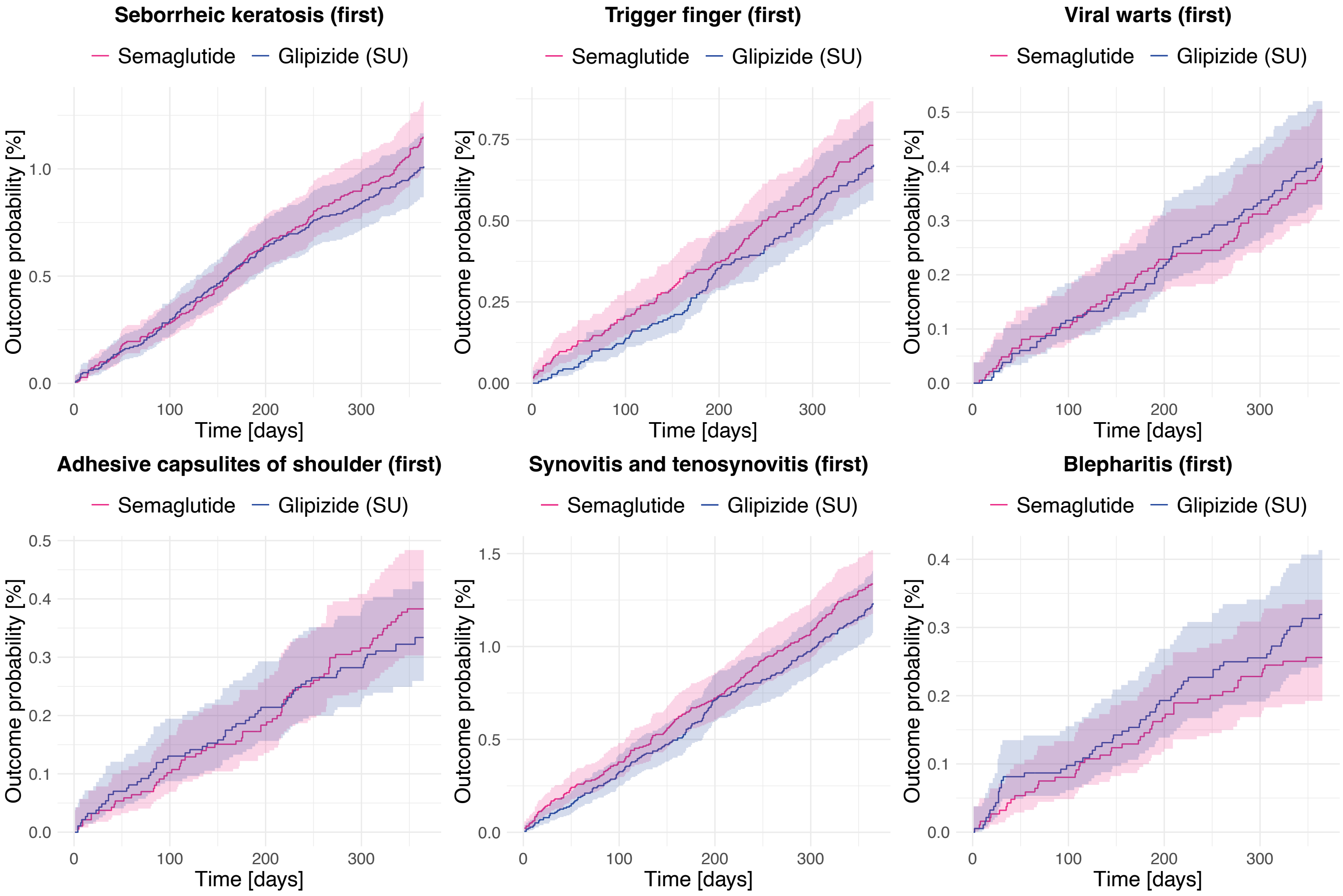
Seborrheic keratosis (first)
Trigger finger (first)
Viral warts (first)
Adhesive capsulites of shoulder (first)
Synovitis and tenosynovitis (first)
Blepharitis (first)
Semaglutide
Glipizide (SU)
Outcome probability [%]
Time [days]
0
100
200
300
0.0
0.5
1.0
1.5
0.00
0.25
0.50
0.75
0.1
0.2
0.3
0.4
0.5

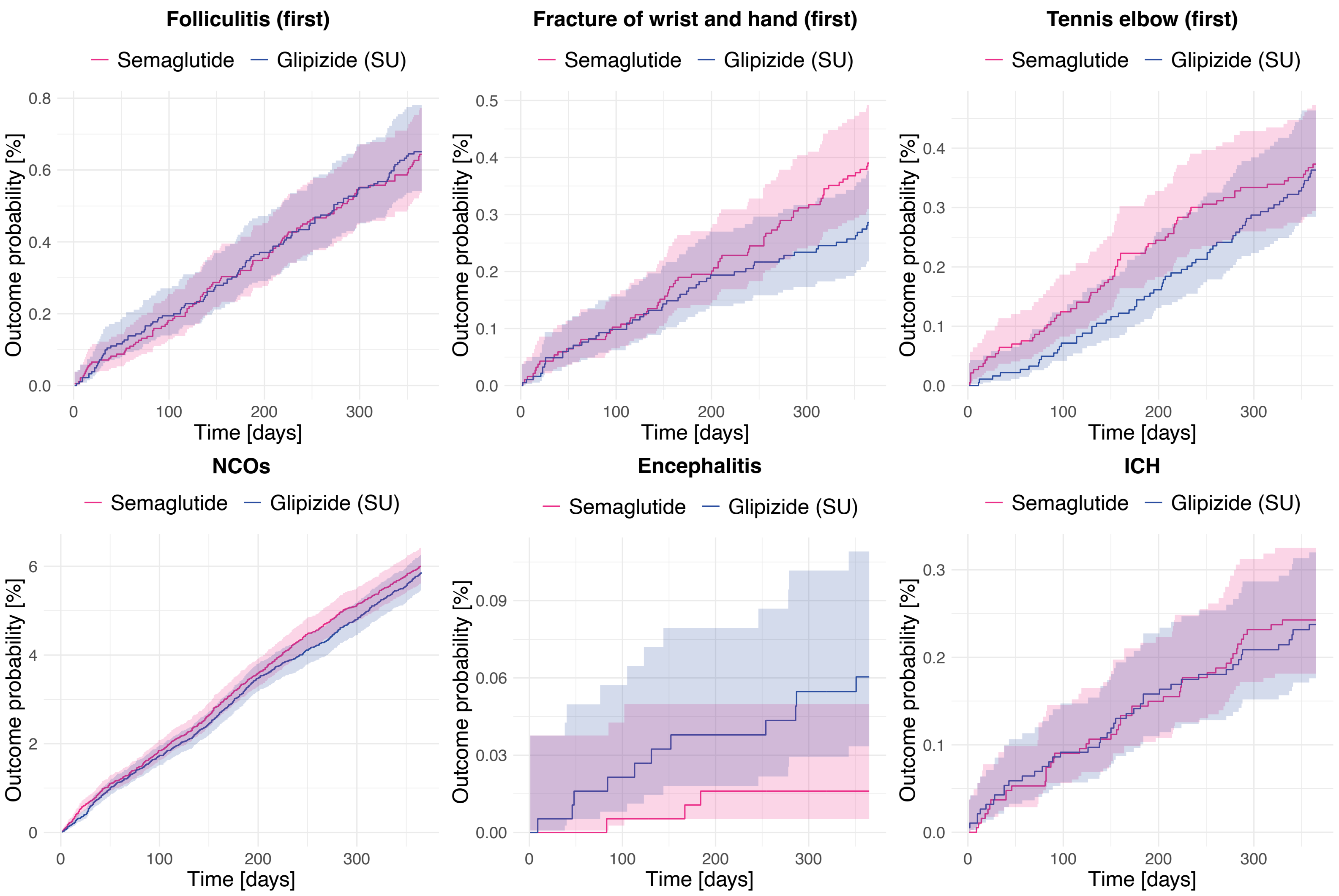
Folliculitis (first)
Semaglutide
Glipizide (SU)
Outcome probability [%]
0.0
0.2
0.4
0.6
0.8
0
100
200
300
Time [days]
Fracture of wrist and hand (first)
Semaglutide
Glipizide (SU)
Outcome probability [%]
0.0
0.1
0.2
0.3
0.4
0.5
0
100
200
300
Time [days]
Tennis elbow (first)
Semaglutide
Glipizide (SU)
Outcome probability [%]
0.0
0.1
0.2
0.3
0.4
0
100
200
300
Time [days]
NCOs
Semaglutide
Glipizide (SU)
Outcome probability [%]
0
2
4
6
0
100
200
300
Time [days]
Encephalitis
Semaglutide
Glipizide (SU)
Outcome probability [%]
0.00
0.03
0.06
0.09
0
100
200
300
Time [days]
ICH
Semaglutide
Glipizide (SU)
Outcome probability [%]
0.0
0.1
0.2
0.3
0
100
200
300
Time [days]

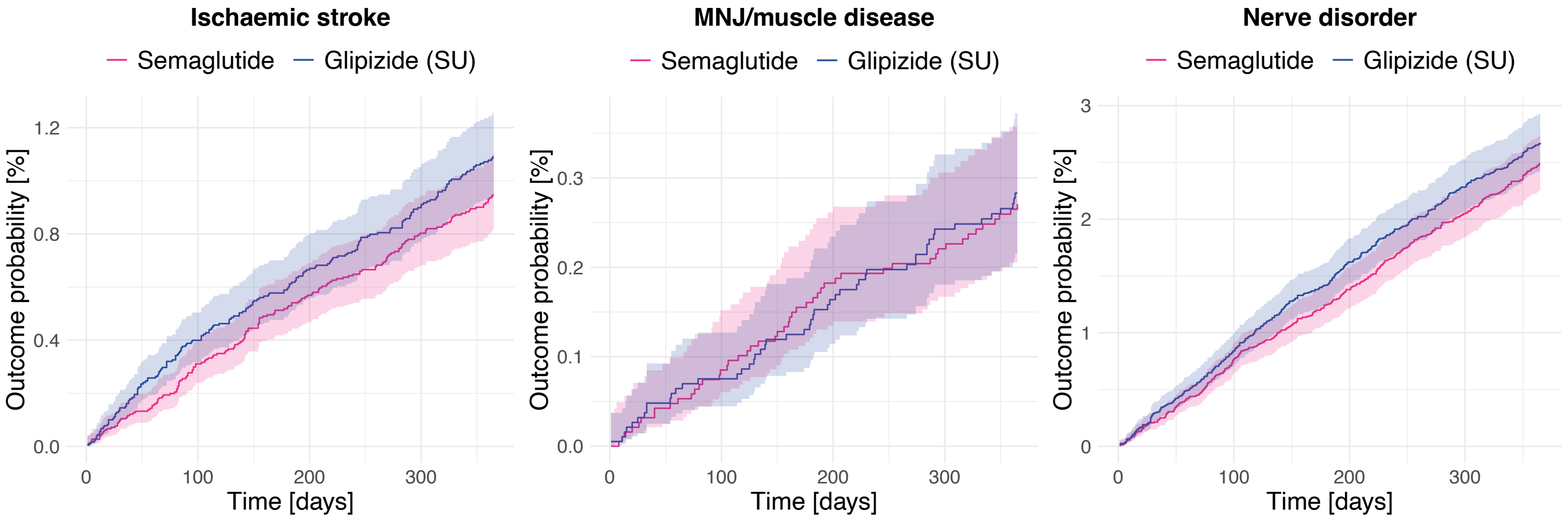

Ischaemic stroke
Semaglutide
Glipizide (SU)
Outcome probability [%]
0.0
0.4
0.8
1.2
0
100
200
300
Time [days]
MNJ/muscle disease
Semaglutide
Glipizide (SU)
Outcome probability [%]
0.0
0.1
0.2
0.3
0
100
200
300
Time [days]
Nerve disorder
Semaglutide
Glipizide (SU)
Outcome probability [%]
0
1
2
3
0
100
200
300
Time [days]

Any diagnosis

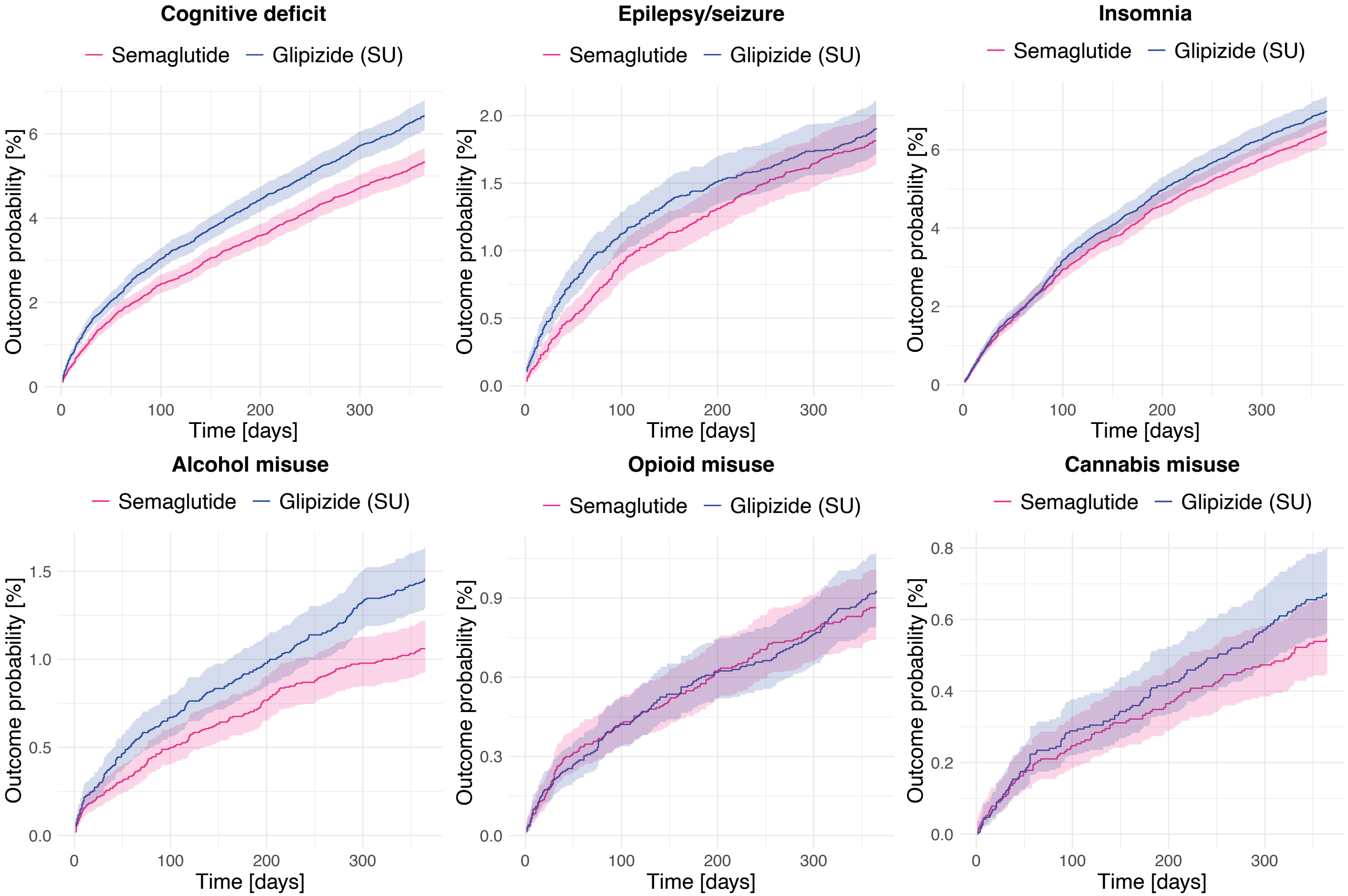

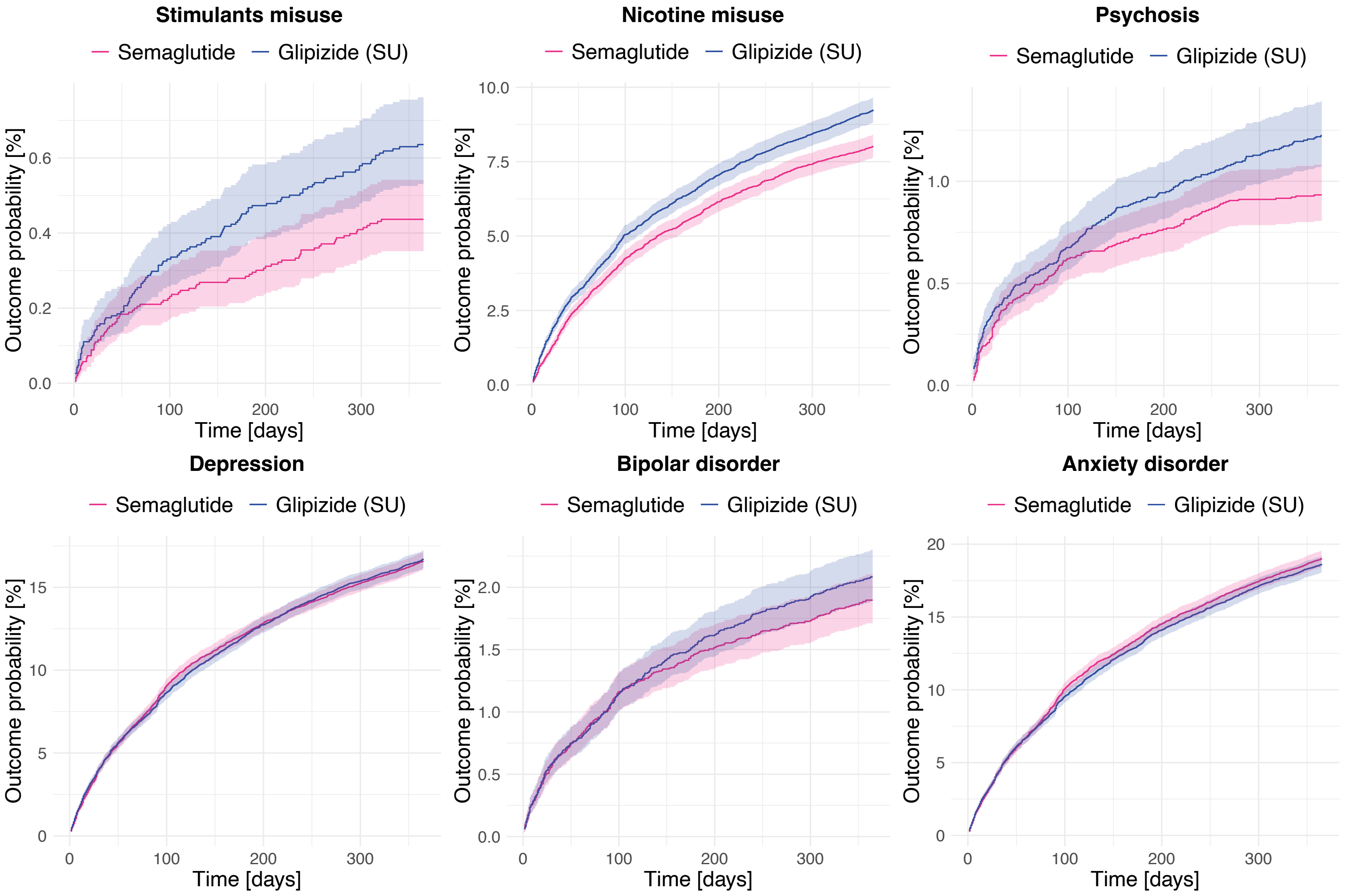
Stimulants misuse
Semaglutide
Glipizide (SU)
Outcome probability [%]
0.0
0.2
0.4
0.6
0
100
200
300
Time [days]
Nicotine misuse
Semaglutide
Glipizide (SU)
Outcome probability [%]
0.0
2.5
5.0
7.5
10.0
0
100
200
300
Time [days]
Psychosis
Semaglutide
Glipizide (SU)
Outcome probability [%]
0.0
0.5
1.0
0
100
200
300
Time [days]
Depression
Semaglutide
Glipizide (SU)
Outcome probability [%]
0
5
10
15
0
100
200
300
Time [days]
Bipolar disorder
Semaglutide
Glipizide (SU)
Outcome probability [%]
0.0
0.5
1.0
1.5
2.0
0
100
200
300
Time [days]
Anxiety disorder
Semaglutide
Glipizide (SU)
Outcome probability [%]
0
5
10
15
20
0
100
200
300
Time [days]

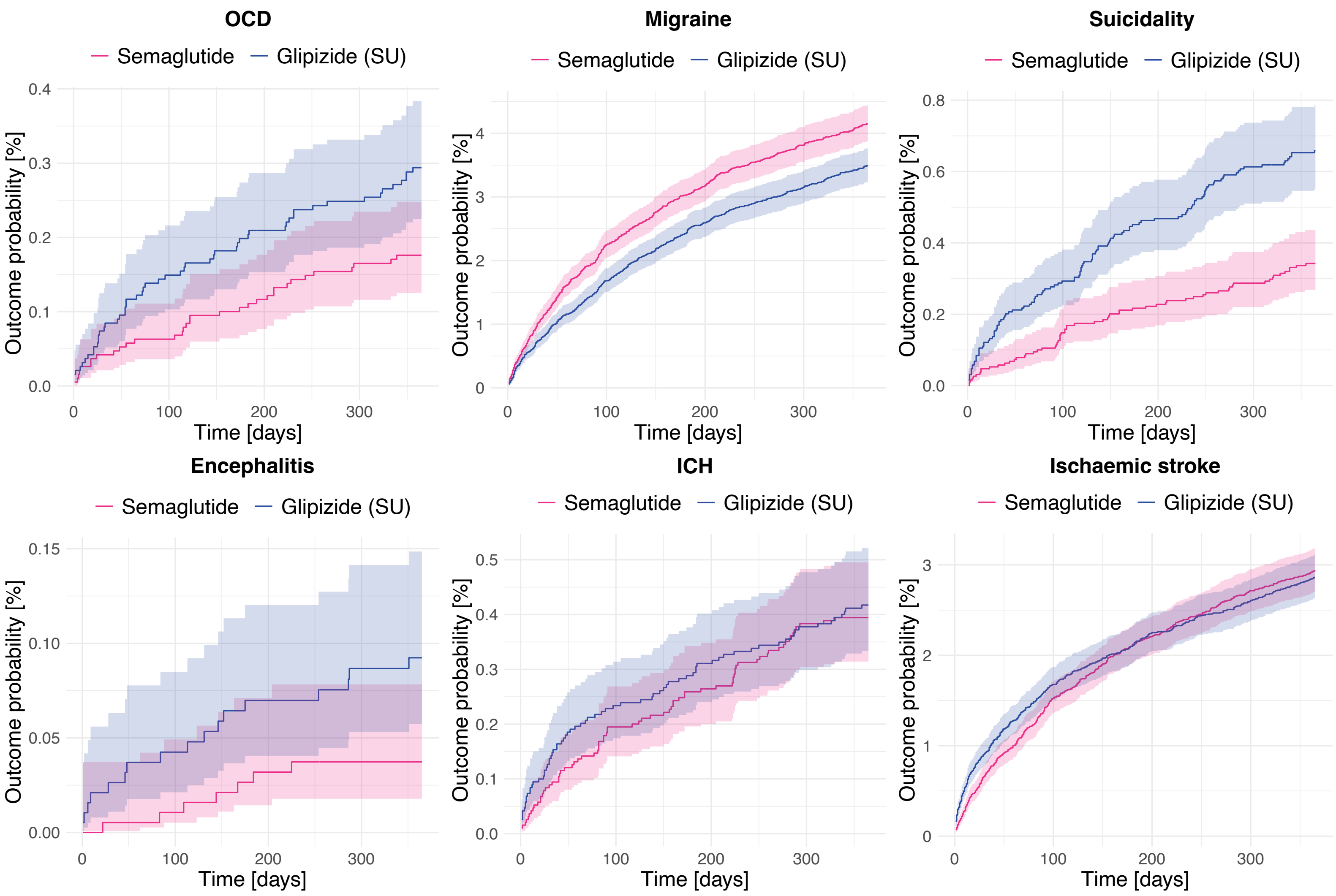
OCD
Semaglutide
Glipizide (SU)
Outcome probability [%]
0.0
0.1
0.2
0.3
0.4
0
100
200
300
Time [days]
Migraine
Semaglutide
Glipizide (SU)
Outcome probability [%]
0
1
2
3
4
0
100
200
300
Time [days]
Suicidality
Semaglutide
Glipizide (SU)
Outcome probability [%]
0.0
0.2
0.4
0.6
0.8
0
100
200
300
Time [days]
Encephalitis
Semaglutide
Glipizide (SU)
Outcome probability [%]
0.00
0.05
0.10
0.15
0
100
200
300
Time [days]
ICH
Semaglutide
Glipizide (SU)
Outcome probability [%]
0.0
0.1
0.2
0.3
0.4
0.5
0
100
200
300
Time [days]
Ischaemic stroke
Semaglutide
Glipizide (SU)
Outcome probability [%]
0
1
2
3
0
100
200
300
Time [days]

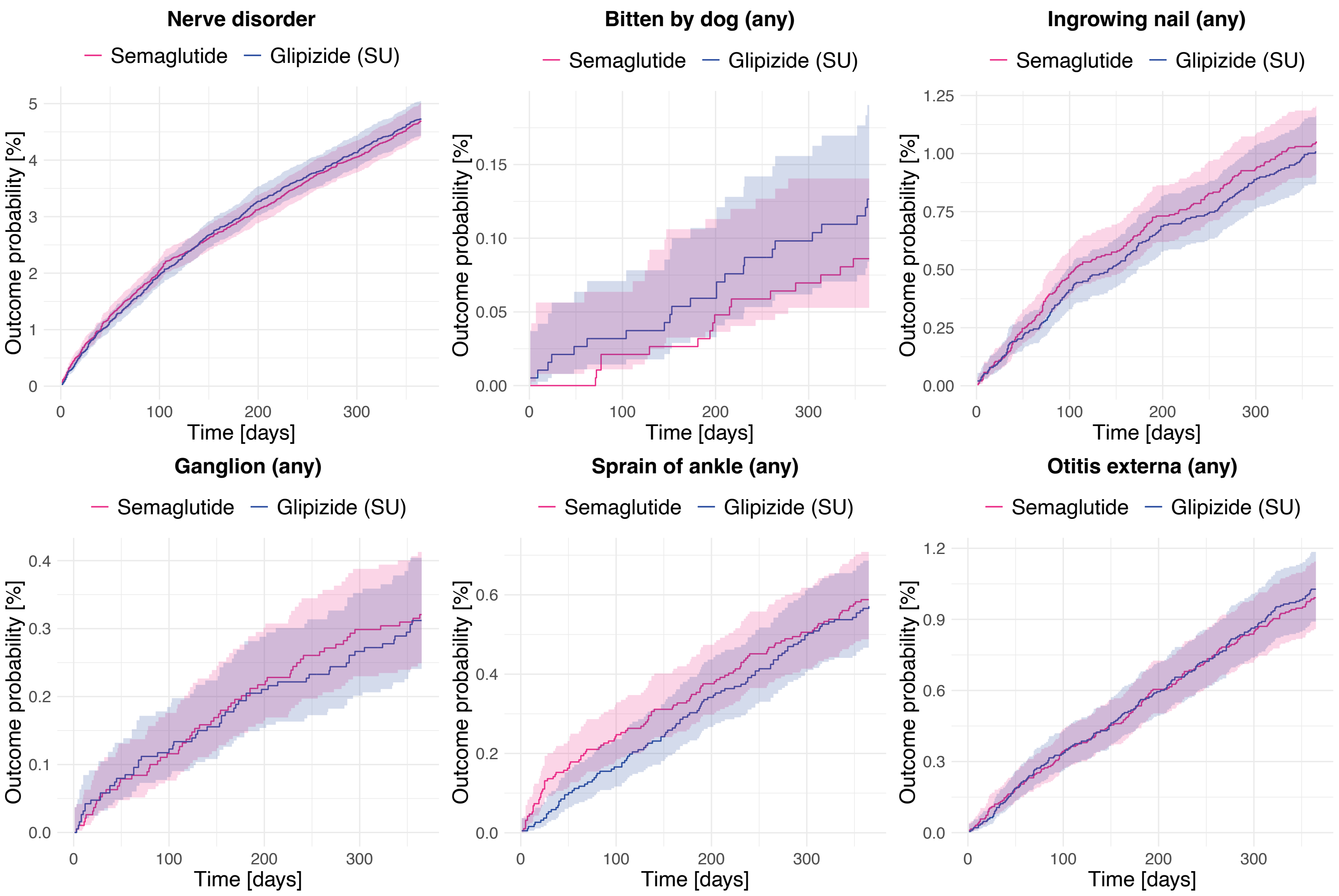
Nerve disorder
Semaglutide
Glipizide (SU)
Outcome probability [%]
0
1
2
3
4
5
0
100
200
300
Time [days]
Bitten by dog (any)
Semaglutide
Glipizide (SU)
Outcome probability [%]
0.00
0.05
0.10
0.15
0
100
200
300
Time [days]
Ingrowing nail (any)
Semaglutide
Glipizide (SU)
Outcome probability [%]
0.00
0.25
0.50
0.75
1.00
1.25
0
100
200
300
Time [days]
Ganglion (any)
Semaglutide
Glipizide (SU)
Outcome probability [%]
0.0
0.1
0.2
0.3
0.4
0
100
200
300
Time [days]
Sprain of ankle (any)
Semaglutide
Glipizide (SU)
Outcome probability [%]
0.0
0.2
0.4
0.6
0
100
200
300
Time [days]
Otitis externa (any)
Semaglutide
Glipizide (SU)
Outcome probability [%]
0.0
0.3
0.6
0.9
1.2
0
100
200
300
Time [days]

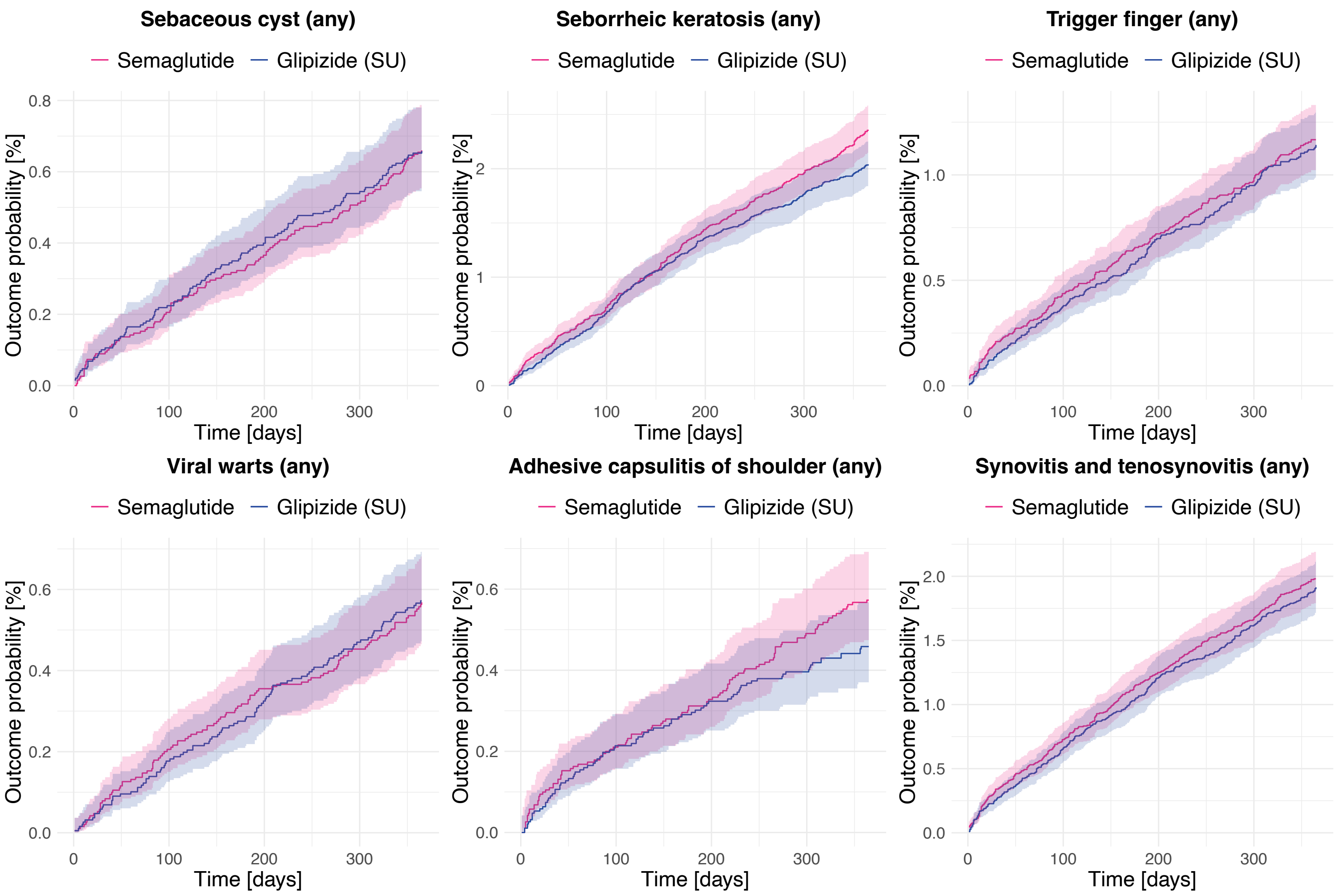
Sebaceous cyst (any)
Semaglutide
Glipizide (SU)
Outcome probability [%]
0.0
0.2
0.4
0.6
0.8
0
100
200
300
Time [days]
Seborrheic keratosis (any)
Semaglutide
Glipizide (SU)
Outcome probability [%]
0
1
2
0
100
200
300
Time [days]
Trigger finger (any)
Semaglutide
Glipizide (SU)
Outcome probability [%]
0.0
0.5
1.0
0
100
200
300
Time [days]
Viral warts (any)
Semaglutide
Glipizide (SU)
Outcome probability [%]
0.0
0.2
0.4
0.6
0
100
200
300
Time [days]
Adhesive capsulitis of shoulder (any)
Semaglutide
Glipizide (SU)
Outcome probability [%]
0.0
0.2
0.4
0.6
0
100
200
300
Time [days]
Synovitis and tenosynovitis (any)
Semaglutide
Glipizide (SU)
Outcome probability [%]
0.0
0.5
1.0
1.5
2.0
0
100
200
300
Time [days]

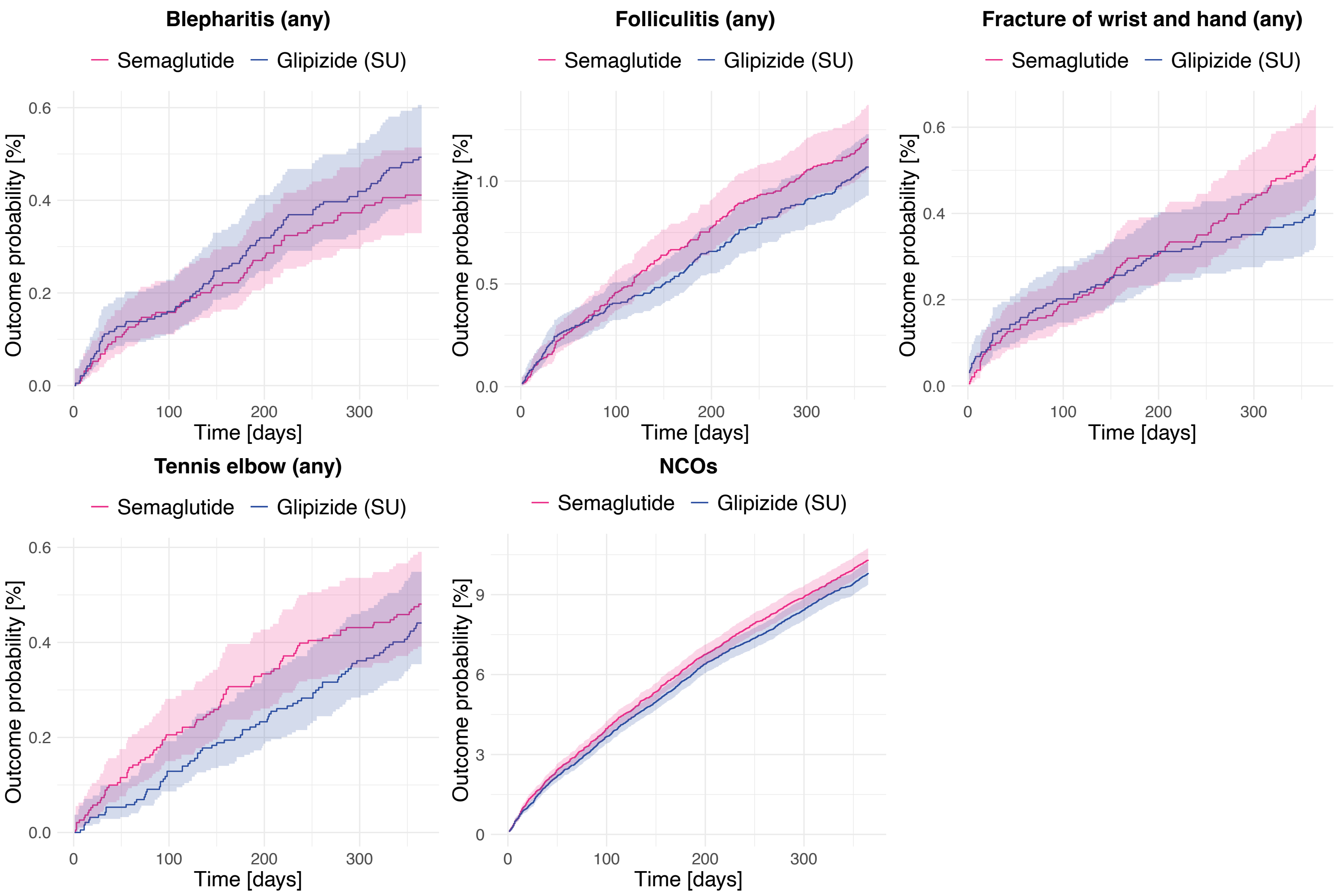
Blepharitis (any)
Semaglutide
Glipizide (SU)
Outcome probability [%]
0.0
0.2
0.4
0.6
0
100
200
300
Time [days]
Folliculitis (any)
Semaglutide
Glipizide (SU)
Outcome probability [%]
0.0
0.5
1.0
0
100
200
300
Time [days]
Fracture of wrist and hand (any)
Semaglutide
Glipizide (SU)
Outcome probability [%]
0.0
0.2
0.4
0.6
0
100
200
300
Time [days]
Tennis elbow (any)
Semaglutide
Glipizide (SU)
Outcome probability [%]
0.0
0.2
0.4
0.6
0
100
200
300
Time [days]
NCOs
Semaglutide
Glipizide (SU)
Outcome probability [%]
0
3
6
9
0
100
200
300
Time [days]

Supplementary Figure S6. Time-varying hazard ratios

a) Semaglutide vs Sitagliptin

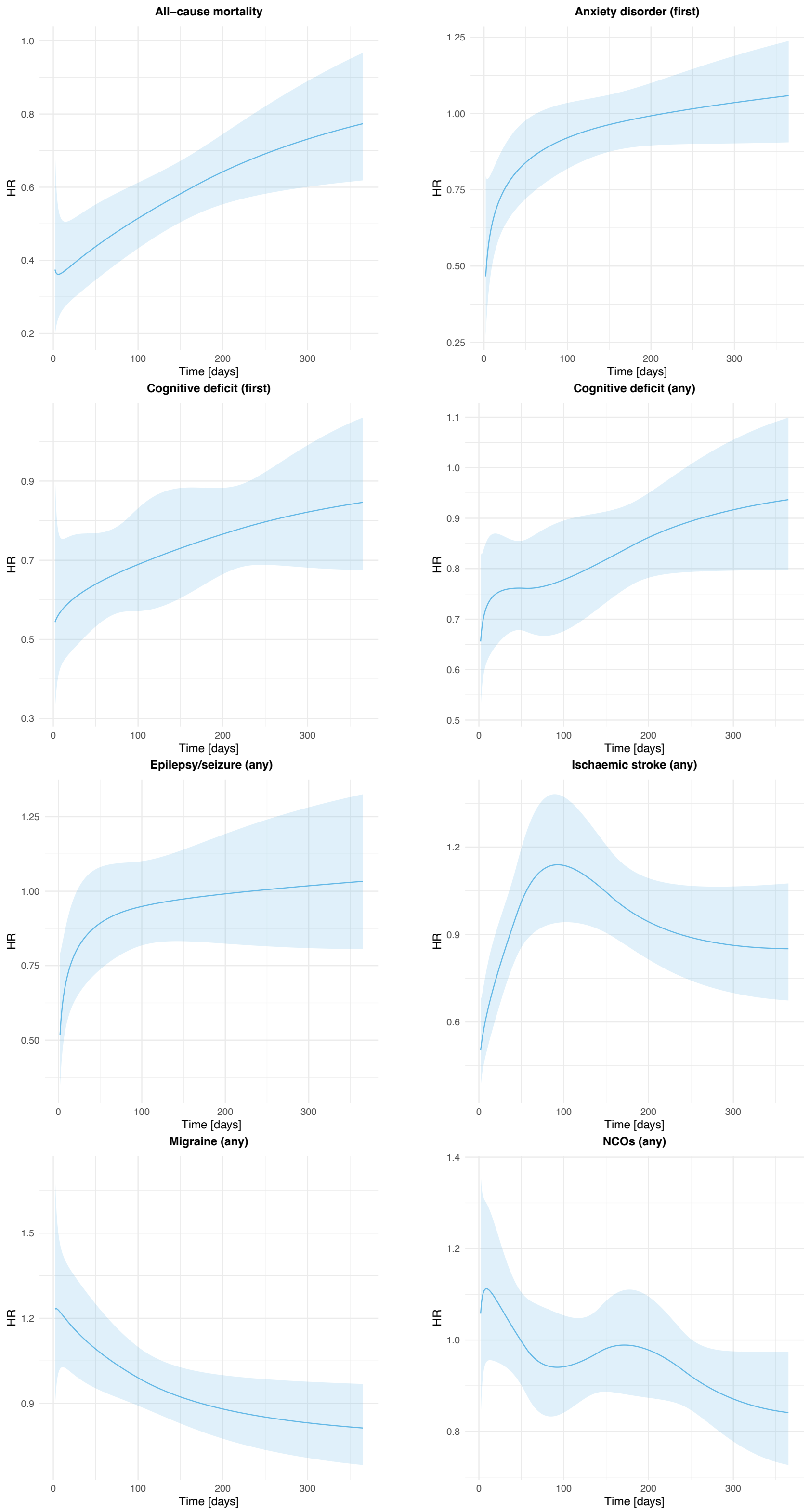

b) Semaglutide vs Empagliflozin

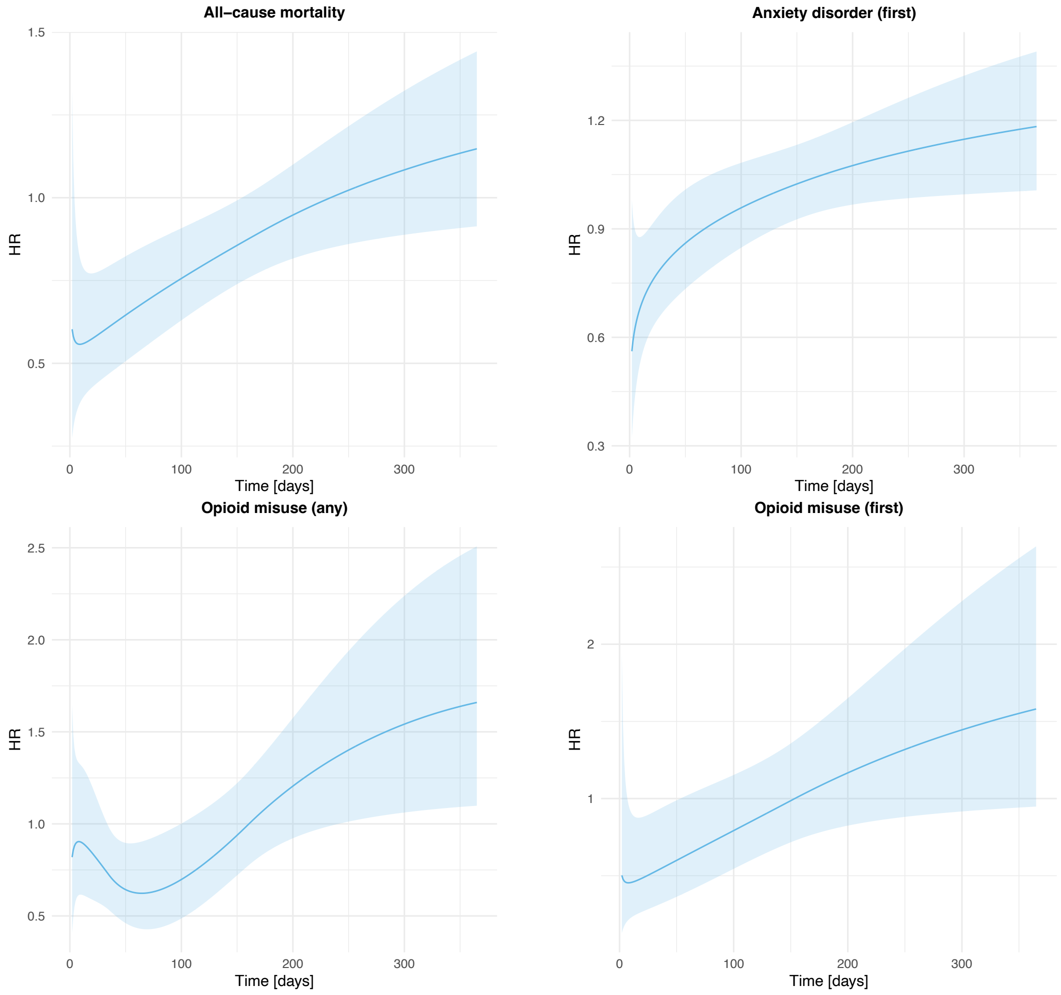

c) Semaglutide vs Glipizide

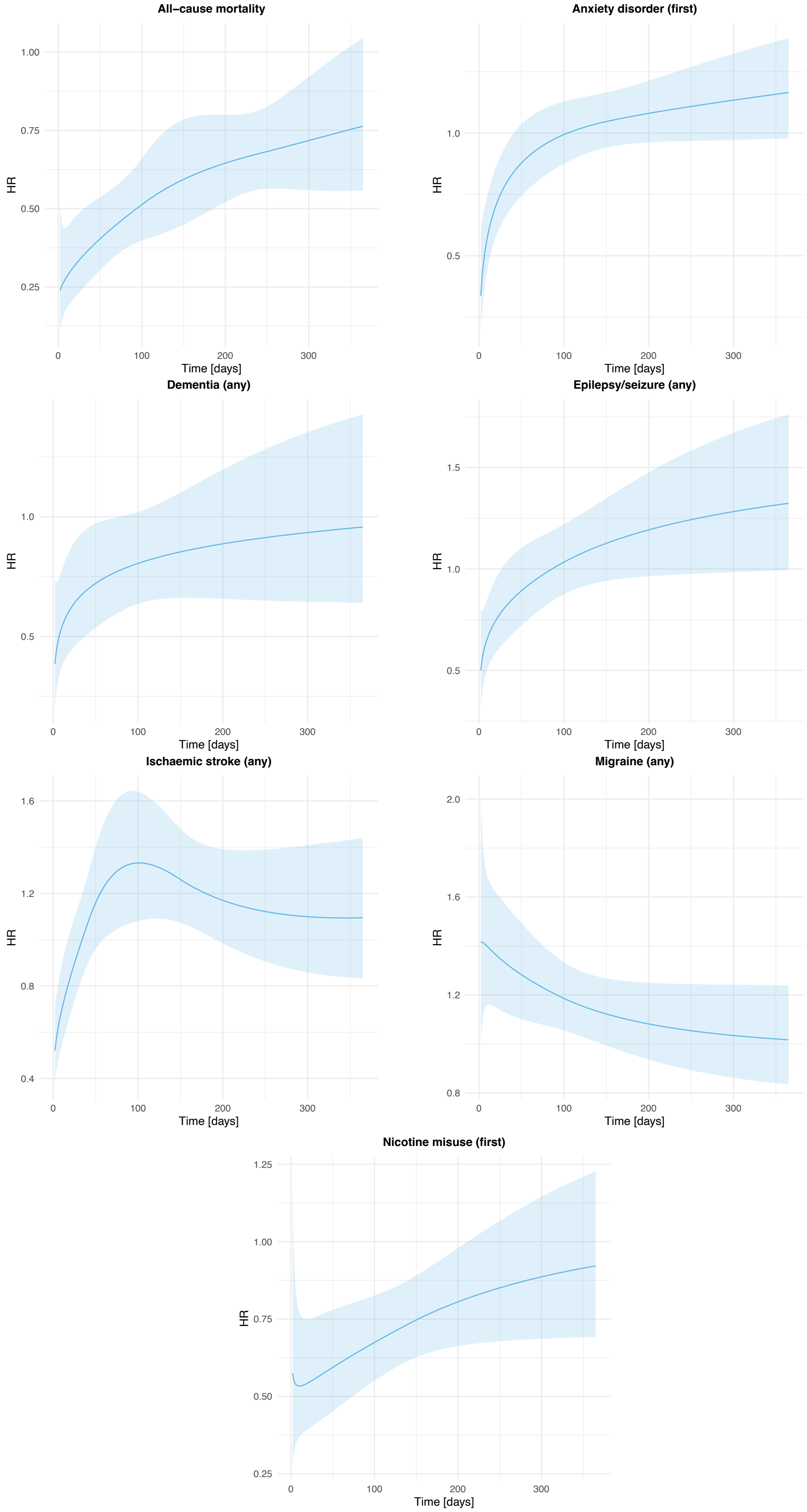

SUPPLEMENTARY REFERENCES

1. Taquet M, Geddes JR, Husain M, Luciano S, Harrison PJ. 6-month neurological and psychiatric outcomes in 236 379 survivors of COVID-19: a retrospective cohort study using electronic health records. *Lancet Psychiatry* 2021; **8**(5): 416-27.

2. Wang W, Volkow ND, Berger NA, Davis PB, Kaelber DC, Xu R. Association of semaglutide with risk of suicidal ideation in a real-world cohort. *Nat Med* 2024; **30**(1): 168-76.

3. Casey JA, Schwartz BS, Stewart WF, Adler NE. Using Electronic Health Records for Population Health Research: A Review of Methods and Applications. *Annu Rev Public Health* 2016; **37**: 61-81.

4. Cowie MR, Blomster JI, Curtis LH, et al. Electronic health records to facilitate clinical research. *Clin Res Cardiol* 2017; **106**(1): 1-9.

5. Jetley G, Zhang H. Electronic health records in IS research: Quality issues, essential thresholds and remedial actions. *Decision Support Systems* 2019; **126**: 113137.

6. de Cates AN, Harmer CJ, Harrison PJ, et al. Association between a selective 5-HT4 receptor agonist and incidence of major depressive disorder: an emulated target trial. *BJPsych* in press.

7. De Crescenzo F, Ciabattini M, D'Alò GL, et al. Comparative efficacy and acceptability of psychosocial interventions for individuals with cocaine and amphetamine addiction: A systematic review and network meta-analysis. *PLoS Med* 2018; **15**(12): e1002715.

8. Taquet M, Dercon Q, Luciano S, Geddes JR, Husain M, Harrison PJ. Incidence, co-occurrence, and evolution of long-COVID features: A 6-month retrospective cohort study of 273,618 survivors of COVID-19. *PLoS Med* 2021; **18**(9): e1003773.

9. Taquet M, Sillett R, Zhu L, et al. Neurological and psychiatric risk trajectories after SARS-CoV-2 infection: an analysis of 2-year retrospective cohort studies including 1 284 437 patients. *Lancet Psychiatry* 2022; **9**(10): 815-27.

10. Royston P, Parmar MK. Flexible parametric proportional-hazards and proportional-odds models for censored survival data, with application to prognostic modelling and estimation of treatment effects. *Stat Med* 2002; **21**(15): 2175-97.

11. Liu XR, Pawitan Y, Clements M. Parametric and penalized generalized survival models. *Stat Methods Med Res* 2018; **27**(5): 1531-46.
